# Efficacy of N,N‘bis-(2-mercaptoethyl) isophthalamide on mercury intoxication: a randomized controlled trial

**DOI:** 10.1186/s12940-018-0358-1

**Published:** 2018-02-14

**Authors:** Paul Schutzmeier, Augusto Focil Baquerizo, Wilson Castillo-Tandazo, Nicholas Focil, Stephan Bose-O’Reilly

**Affiliations:** 1 Institute and Outpatient Clinic for Occupational, Social and Environmental Medicine, WHO Collaborating Centre for Occupational Health, University Hospital Munich, Ziemssenstr. 1, D-80336 Munich, Germany; 2 FOMAT Medical Research, Oxnard, CA USA; 3 Institute of Public Health, Medical Decision Making and Health Technology Assessment, Department of Public Health, Health Services Research and Health Technology Assessment, UMIT (University for Health Sciences, Medical Informatics and Technology), A-6060 Hall i.T, Innsbruck, Austria

**Keywords:** Mercury, Chronic mercury intoxication, Gold mining, NBMI, Chelation therapy

## Abstract

**Background:**

Chronic mercury intoxication is a severe health issue and occurs especially in gold mining communities. Common chelators used for improving mercury elimination are not everywhere available and challenged by poor cell wall penetration. This study is part of a feasibility trial and the aim was to gather first information about the efficacy of the newly developed chelator N,N‘bis-(2-mercaptoethyl) isophthalamide (NBMI) on chronic mercury intoxication.

**Methods:**

In this three-armed, placebo-controlled randomized trial, 36 miners with mercury urine levels exceeding 15 μg/l were administered 100 mg NBMI, 300 mg NBMI or placebo for 14 days. Levels of mercury in urine [μg/l and μg/g creatinine] and plasma l were analyzed. Therapeutic effect was assessed using the medical intoxication score (MIS) and its single health outcomes (e.g. excessive salivation, sleeping problems), fatigue scores, a neuromotoric test battery (CATSYS) and a neurological outcome (Finger to nose test).

**Results:**

Physical fatigue was significantly decreased in the 300 mg NBMI group compared to the control. Mercury concentration in urine following 300 mg NBMI treatment was significantly lowered compared to control, however, this effect was less distinct with adjustment for creatinine.

**Conclusion:**

NBMI showed an effect on physical fatigue and there were indications to positive effects on other symptoms as well. More comprehensive studies are mandatory to verify the effects of NBMI as a novel tool for treating mercury intoxications.

**Trial registration:**

ClinicalTrials.gov Identifier: NCT02486289. Date of registration: June 24, 2015.

**Electronic supplementary material:**

The online version of this article (10.1186/s12940-018-0358-1) contains supplementary material, which is available to authorized users.

## Background

Mercury (Hg) is a highly toxic heavy metal; acute as well as chronic intoxication can occur. It accumulates in the body over time and can cause severe damage to kidneys and neural tissues [1–3]. A recent study determined the severity of chronic mercury intoxication in terms of disability weights and found the most severe case of intoxication to be more serious than severe depression and quadriplegia [4]. Common chronic intoxication symptoms are tremor, ataxia of gait, coordination problems, excessive salivation and sleeping problems [5, 6].

The risk of exposure is especially high in gold producing areas, since practices using elemental mercury are still very common in artisanal small-scale gold mining (ASGM) [7, 8]. The global number of people earning their living with ASGM is estimated to be 10 to 16 million in 70 countries [7, 9]. By mixing the pulverized gold ore with elemental mercury and burning the so formed amalgam without protection, the miners are directly exposed by inhalation of the toxic vapor [10, 11]. This results in increased mercury burden, which is detectable in urine, blood and hair. Moreover, the proximity of the processing to living spaces and lack of awareness for toxic waste disposal affects not only the miners themselves, but also their children and communities by food, air, water and soil pollution [12–14].

There are two common chelating agents in use for treating mercury intoxication at the moment: the British Anti-Lewisite derivates 2,3-dimercapto-1-propanesulfonate (DMPS) and dimercaptosuccinic acid (DMSA). DMSA is approved by the U.S. Food and Drug administration to treat patients with increased mercury levels by eliminating lead, but is also used as an off-label medication for Hg intoxication in North America. DMPS is approved in Germany and used across Europe for excreting Hg. However, unfortunately, in many countries where ASGM and the exposure to mercury are widespread, e.g. Ecuador and Indonesia, neither is available. As the Hg exposure mainly affects the poorer parts of the population, e.g. informal miners and their families, there is very little effort from the private sector to improve the situation. Nevertheless, as recognized by the Minamata Convention in 2013, along with prevention, the treatment of Hg intoxication is a paramount task and an efficacious and available medication much needed.

Several studies described the efficacy on mercury intoxication of both agents [15–18]. Additionally, a recent report suggests that DMPS and DMSA do not form true chelate complex with mercuric ions [19]. Unfavorable stereo-chemical properties allow only one thiol of the chelators to bind to the metal resulting in a weaker bond. Thus, there might be possibilities to further improve the clinical efficacy [19]. On top, both are charged molecules and, as a result, are not able to pass the blood brain barrier or effectively enter cells. Hence, they are not able to extract the heavy metal directly from brain tissue and within the cells [20], recent reports even postulates redistribution of Hg into neuronal tissue [21]. Consequently, novel approaches for metal elimination are highly desirable [18].

**Fig. 1:**
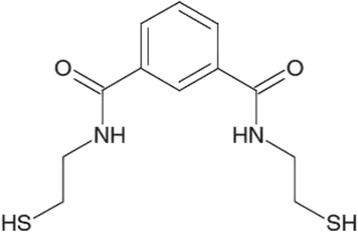
Chemical structure and basic facts

N,N‘bis-(2-mercaptoethyl) isophthalamide (NBMI) is a newly developed, lipophilic chelating agent specifically created for complexing inorganic mercury (see Fig. 1). This field study was embedded in an explorative trial to test the feasibility for an upcoming, larger phase-IIb clinical study. These smaller studies are needed to test outcomes, infrastructure, study parameters and local circumstances and to avoid problems in following bigger and more expensive studies. While its cytoprotective, anti-oxidative properties and its impact on acute Hg intoxication in rats are already proven [22–24], the aim of this pilot study was to gain first knowledge about the efficacy of NBMI on chronic mercury intoxication in humans.

## Methods

### Study design

The design of this trial was three-armed, randomized, double blinded and placebo controlled, consisting of 14 days of treatment and 1 month of follow-up, totaling to a length of 45 days. It was conceptualized as a pilot study to gain knowledge about dose and efficacy of NBMI in mercury-intoxicated individuals. Furthermore, the feasibility of a future phase-IIb study at the site was examined. The three arms consisted of two treatment groups, treated with 100 mg/d NBMI and 300 mg/d NBMI, respectively, and one placebo group. The subjects were randomized into the treatment arms in equal numbers to obtain a 1:1:1 ratio.

### Study population

**Fig. 2:**
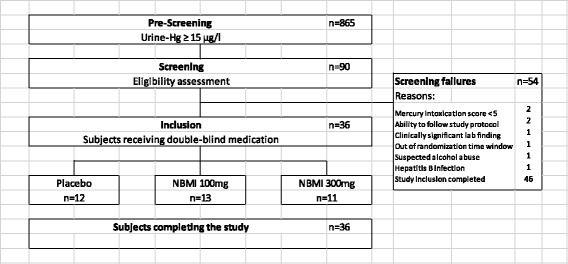
Subject Disposition

The study subjects were recruited from the local gold miner population, since the artisanal miners in the region of Zaruma/Portovelo, Ecuador, were occupationally exposed to mercury by handling it directly to amalgamate the gold dust or smelting amalgam to extract the gold. The miners were approached in collaboration with local mining organizations and through advertising in the local community. An informed consent to participate in the study was obtained from all participants. Urine samples of 865 interested miners were collected and analyzed for their mercury content. The purpose of this prescreening was to identify individuals with mercury in urine values > 15 μg/l for further screening. Of 90 persons with mercury urine levels exceeding the threshold 44 were examined whether they met all the inclusion criteria and none of the exclusion criteria until the sample size of 36 was reached (see Appendix and Fig. 2). For inclusion in the study, subjects had to fulfil the following criteria: an age between 18 and 65 years, urine-Hg ≥ 15 μg/l, a mercury intoxication medical score of ≥ 5 or ≥ 3 in combination with at least two of the additional symptoms social nervousness/withdrawal, irritability, memory loss, metallic taste, mental- and physical fatigue.

All the participants obtained an emergency card with information on the investigational product, the subject’s id, the investigator’s name, an emergency number as well as the name and address of the sponsor. The whole study was conducted at the Hospital Doctor Humberto Molina, Zaruma, Ecuador, where direct access to medical emergency care could be provided.

There were no formal sample size calculations for this study, since the main goal was to explore the feasibility for a more extensive study. The size of 36 subjects was chosen as a generally accepted number for a pilot study. 12, 11 and 13 subjects in the treatment groups meant that the 95% confidence intervals for the difference between mean values in a NMBI treated group and the placebo treated group resulted in a length of about 1.6 times the standard deviation.

The three treatment arms differ in participant numbers because after randomizing the first 19 individuals, the necessity to relabel some of the boxes with an extended expiry date occurred which caused an interruption of the process. The subjects corresponded to three blocks of six and the remaining subject was allocated as an incomplete block. With the arrival of the relabeled batch, the randomization was continued with new blocks and caused the inequality in group size. However, this way the subjects could be randomized and keeping the blind of the study.

The participants were analyzed according to the treatment arm they were assigned to, regardless of the treatment received. The analysis included all participants who took at least one dose of medication (NBMI or placebo) and at least one post baseline assessment of the MIS. This analysis set corresponds to a modified intention-to-treat (mITT) analysis set. The per protocol (PP) analysis set includes only participants without major protocol violations. This set was used as a sensitivity analysis for the efficacy endpoints.

As shown in Fig. 2, 36 subjects were selected for the study. 34 (94.4%) participants were administered the assigned daily dose of the study product on site for all 14 days of treatment. Two subjects took only one capsule for the first 2 and 3 days, respectively, based on an administrative error in the hospital. The following 12 and 11 days all doses were taken as planned by both subjects. One of those two subjects had been allocated to the placebo arm and missed no active dose.

### Treatment

For the 14 days of the intervention 100 mg NBMI, 300 mg NBMI or placebo were administered orally to all patients. The medication comprised of three capsules per administration for each group, two containing 50 mg and one containing 200 mg, either of the investigational product, placebo or a combination of both in the following regime:

300 dose arm: two 50 mg NBMI capsules and one 200 mg NBMI capsule,

100 mg dose arm: two 50 mg NBMI capsules and one 200 mg placebo capsule,

Placebo dose arm: two 50 mg placebo capsules and one 200 mg placebo capsule.

The use of placebo filled dummy capsules ensured the blinding of the patients. No restriction on medication intake in relation to meals was set. Other medication necessary for the patients’ well-being was allowed to be administered at the investigator’s discretion, but recorded appropriately. The subjects were asked to abstain from drinking energy drinks containing taurine or glucuronolactone and alcohol equivalent to more than two half-liter bottles per day 72 h prior to the medical assessment visits at day 1, 6 ± 1, 15 and 45 ± 1. Moreover, for the length of treatment, the subjects were to refrain from the intake of herbal medicine, grapefruit or grapefruit juice to avoid possible drug interactions. As the use of mercury in mining is banned in Ecuador no restriction to working in the mines were made.

#### Randomization and assignment to treatment groups

Upon enrolment, each participant was assigned a consecutive number. The subjects were allocated to the treatment arms (100 mg/d, 300 mg/d or placebo) at a ratio of 1:1:1. For this purpose, a randomization list was created with the subject numbers using the R system and the subjects were allocated to the three arms in blocks of six.

#### Blinding

At the start of the treatment, the participants were handed out numbered boxes with the capsules corresponding to their subject number. Each participant received the same number of blisters, either filled with NBMI, placebo capsules or both. The boxes and the blisters containing the investigational product and/or placebo were labelled with a subject number by an external packaging company to ensure the blinding of the investigators.

#### Treatment compliance

The treatment compliance was controlled and documented by the use of investigational product accountability logs. In those logs the date and quantities of study products received, dispensed to and used by each subject and products returned and destroyed at the end was recorded. All administration of the study drug was supervised, and each participant received the daily dose at the clinic. The recorded data was verified by the study monitor during the study’s process and at the end of the follow up, all investigational products used or returned were accounted for.

### Outcomes

The main aim of this study was to generate explorative and descriptive data as there is no consistent method for efficacy assessment or guideline for treatment of mercury intoxication. No formal primary efficacy endpoint was predetermined, and the outcomes were specified as differences in comparison to baseline values and the placebo treated subjects. As described in previous reports, the following characteristics were examined: the mercury intoxication medical score (MIS) and its individual components [5]. Moreover, a panel of neuro-motoric functions (CATSYS) [25], mental and physical fatigue score [17], tremor (finger-to-nose test) [6] and mercury values in plasma and urine were analyzed.

#### Biomonitoring

The samples for analyzing the mercury concentration in plasma and urine were taken at day 1 (pre-dose), 15 and 45 and sent to the qualified laboratory ALS Scandinavia AB in Luleå, Sweden for analyzing.

#### Medical intoxication score

The medical intoxication score (MIS) is a tool created for identifying mercury intoxication in patients. It is a ten-point score which is assessed through medical examination, neuromotoric tests and an anamnestic questionnaire [3, 5, 6]. The items consist of excessive salivation, tremor at work, sleeping problems, bluish discoloration of gingiva, ataxia, dysdiadochokinesia as much as a heel-to-shin test, a match box test, a pencil tapping test and additionally a dipstick test for Proteinuria. Each one can have an assigned value of 0 or 1, depending on whether the symptom is absent (0) or present (1) or whether the test result is negative (0) or positive (1). The mercury intoxication medical score is the sum of the items’ values.

In this study, a change in the score and the value of its individual items compared to baseline assessment was used as an efficacy parameter. Accordingly, the change value for the score ranges from − 10 (maximal improvement of symptoms) to 7 (maximal worsening of symptoms), since the least score for subject inclusion is 3. The change value for the individual items can be − 1 (for worsened condition), 0 (for unchanged condition) and 1 (for improved condition). The mercury intoxication medical score was assessed at screening, visit day 15 and visit day 45.

#### Mental and physical fatigue score

This score consists of 13 items of which 8 describe physical fatigue and 5 concern mental fatigue [26]. The items were assessed in five categories with score values from 0 to 3: “better or much better than usual” (0), “same as usual” (1), “worse than usual” (2) and “much worse than usual” (3). This means, the higher the score, the worse the fatigue. The achievable score values were 0–15 for mental fatigue and 0–24 for physical fatigue. Items are on a relative scale to baseline, which has by definition a value of 1 (“same as usual”) for each item, resulting in a mental fatigue score of 5 and a physical fatigue score of 8. The score was assessed at day 2–13, day 15 and 45 of follow up.

For statistical analysis, a fixed model for repeated measures was fitted to the endpoints mental fatigue score and physical fatigue score. All results from day 2 to 15 were included to estimate the change from usual for each day assessed. The comparison of the NBMI treatment groups with the placebo group is based on the change from baseline on day 15.

A sensitivity analysis was performed for the results of the statistical examination based on non-parametric tests (Wilcoxon–Mann–Whitney test), because diagnostic tests and scrutiny of the model residuals hinted at a violation of the assumption of normality.

#### Neuromotoric test battery CATSYS

To record neurological symptoms caused by mercury intoxication objectively, a common computerized test system for measuring motoric skills was used (CATSYS). It assesses hand coordination, reaction time, postural tremor and postural stability (www.catsys.dk) [27] and was already successfully used to measure the effects of chronic mercury exposure [25, 28, 29]. The tremor intensity was statistically analyzed for differences between the treatment arms.

A set of performance indices available for the CATSYS was calculated. The resulting index values were referenced and compared to the performance of a large sound population given by the company [27]. These indices evaluate the tests for tremor intensity and frequency, sway characteristics as much as the test for reaction time and the tests for hand coordination skills. The instructions for the CATSYS state, that an index value of 1 corresponds to the performance of a healthy population. Index results strongly deviating from 1 signify an abnormal performance.

Each participant received an individual instruction and demonstration of the tests by the investigator and was subsequently examined for about 15 min.

#### Finger-to-nose test

The finger-to-nose test detects tremor and can take on following values: Absent (0), slight (1) and moderate to severe (2) [30]. The baseline was assessed at day 1 and compared to the results assessed at day 15 and 45 of follow up.

### Statistical methods

Only descriptive statistics have been used to present all efficacy variables. Continuous variables were summarized with sample size, median, minimum and maximum value. Categorical data are shown in frequency tables with number of subjects, frequency and percentage of occurrence. Individual data were presented in subject listings. When applicable, point estimates, together with their 95% confidence intervals have been presented.

The results of the two NBMI treatment groups were separately compared with the placebo group on day 15. To test for differences between the groups, the ANCOVA method, adjusting for baseline level, was used for parametric distributed characteristics, while the Wilcoxon–Mann–Whitney test was used for non-parametric results. Using the mixed model repeated measures method, a model was fitted to the daily measures of fatigue scores, mental and physical fatigue, including all results of days 2 to 15 to estimate the mean change from usual of fatigue at each day. The comparison between the groups treated with NBMI and the one treated with placebo is based on the change from usual on day 15. This change was individually calculated as the difference of baseline values and the values on day 15 or day 45 and the groups analyzed for distinctions in the change values. The initial model was fitted using an unstructured covariance between repeated measures. If convergence failed, the following covariance structures were tested in the given order until convergence was reached: Unstructured, Heterogeneous Toeplitz, Heterogeneous autoregressive, Toeplitz, Autoregressive.

All assessed data were included in the statistical summary and missing data were not imputed. All statistical analyses were performed using the version 9.4 of the SAS® software.

### Safety assessment

Adverse events were identified by spontaneous reports from the subjects, observations by the investigators or the medical personnel or through elicitation based on non-leading questions by the study personnel. All adverse events were recorded in the case report files along with a diagnosis, when available, or signs and symptoms, start and stop date and time, intensity (mild, moderate or severe), a causal relationship with NBMI (probable, possible or not related). If applicable, the action taken to handle symptoms and its outcome were recorded as well. Blood samples were collected for the examination of clinical chemistry and hematology and sent to a laboratory at screening and on days 1,6,15 and 45. On the same days, a full physical examination was done to control the vital signs and the general condition as well as the pulmonic, the cardiovascular, the abdominal and the neurological state of the participants.

### Ethics

The study protocol and study informed consent forms (ICF) were submitted to the Institutional Review Board (IRB) of the Universidad de San Francisco de Quito, Diego de Robles y Vía Interoceánica, Quito, Ecuador, for review. After requested revisions, the study protocol and ICF were approved in writing on 26 May 2015. The Regulatory Authorities required revisions and protocol (amendment 2) and ICF were therefore re-submitted to the IRB and approved on 27 Jul 2015.

The study protocol was amended once during the study (amendment 3) to protocol. The amendment, including ICF was submitted to the IRB and approved on 30 September 2015.

The study was performed in accordance with the ethical principles that have their origin in the Declaration of Helsinki and that are consistent with International Conference on Harmonisation/Good Clinical Practice and applicable regulatory requirements on Bioethics.

## Results

### Participants

The demographics and baseline characteristics of the participants are shown in Table 1. They were all Hispanic males. The mean age was 39 years (range: 19–59 years). There was a difference in age distribution between the groups.

**Table 1:** Subject work exposure and fish consumption

Variable	Category	NBMI 100 mg (*n* = 13)	NBMI 300 mg (*n* = 11)	Placebo (*n* = 12)
Age (years)	Mean	43.6	38.5	33.3
	Min	22	26	19
	Max	56	59	47
BMI (kg/m^2^)	Mean	28.5	27.7	29.5
	Min	21.8	23.1	18.9
	Max	36.4	34.4	39.1
Living in the mining area [years]	Median	22	15	31
	Min	3	1.5	1
	Max	54	50	47
Working with mercury [years]	Median	15	5	12
	Min	1	1.5	0.5
	Max	20	30	40
Smelting amalgam to recover the gold (Yes/No)	Yes	13 (100.0%)	7 (63.6%)	9 (75.0%)
Handling mercury to extract the gold from the ore, but no smelting (Yes/No)	Yes	6 (46.2%)	4 (36.4%)	3 (25.0%)
Smelting gold / gold buyer (Yes/No)	Yes	0 (0%)	2 (18.2%)	2 (16.7%)
Another job or no job (Yes/No)	Yes	0 (0%)	2 (18.2%)	1 (8.3%)
Fish Consumption	At least once a week	9 (69.2%)	6 (54.5%)	8 (66.7%)
	Never or less than once a week	4 (30.8%)	5 (45.5%)	4 (33.3%)

It further summarizes the baseline data about the mercury exposure of the participants. It shows the time period of exposure through working and living in the study area, the kind of occupation in the last year and the frequency of eating fish. The exposure to Hg varied greatly in individuals.

Table 2 summarizes the median mercury in urine and plasma values. The groups apparently differed in Hg in urine baseline levels.

**Table 2:** Summary of the mercury in urine and plasma values at screening, day 15 and day 45

	NBMI 300 mg (*n* = 11)	NBMI 100 mg (*n* = 13)	Placebo (*n* = 12)
Mercury in urine [μg/l]^a^			
Screening	26.5 (4.22–321.0)	39.1 (12.6–160.0)	9.16 (1.7–230.0)
Day 15	18.5 (2.1–131)	37.7 (8.1–132.0)	13.9 (1.3–161.0)
Day 45	14.2 (1.8–226.0)	39.1 (2.6–96.1)	11.3 (1.3–275.0)
Mercury in urine [μg/g creatinine]^a^			
Screening	62.4 (7.6–773.2)	66.7 (12.4–200)	18.7 (2.6–488.5)
Day 15	65.2 (2.6–298.0)	64.4 (17.2–241.0)	29.75 (1.8–536.7)
Day 45	34.8 (2.9–1883.3)	53.3 (13.2–340.2)	9.86 (0.4–1797.4)
Mercury in plasma [μg/l]^a^			
Screening	5.8 (1.3–95.9)	6.4 (1.7–25.8)	6.1 (1.4–45.8)
Day 15	6.8 (1.7–64.4)	8.0 (2.6–33.6)	5.1 (1.5–42.9)
Day 45	6.4 (1.8–49.0)	9.3 (1.6–42.2)	5.9 (1.0–90.1)

^a^Shown values are medians. Range is shown in brackets

The different scores at screening, day 15 and day 45 are given in Table 3.

**Table 3:** Summary of median scores and ranges at screening, day 15 and day 45

	NBMI 300 mg (*n* = 11)	NBMI 100 mg (*n* = 13)	Placebo (*n* = 12)
Mercury intoxication score^a^			
Screening	6 (5–8)	6 (4–8)	5 (4–6)
Day 15	3 (1–5)	4 (1–7)	2.5 (0–5)
Day 45	3 (1–5)	4 (2–5)	2.5 (0–4)
Physical fatigue score^a^			
Screening	8 (8)	8 (8)	8 (8)
Day 15	3 (0–7)	4 (0–8)	6 (0–8)
Day 45	3 (0–8)	4 (0–9)	6 (0–8)
Mental fatigue score^a^			
Screening	5 (5)	5 (5)	5 (5)
Day 15	4 (0–5)	4 (0–5)	5 (0–5)
Day 45	4 (0–5)	5 (0–6)	4.5(0–5)
CATSYS Index^a^			
Screening	105 (80–139)	72 (62–129)	89 (59–148)
Day 15	98 (48–144)	95 (29–134)	113 (65–152)
Day 45	106 (61–152)	92 (48–127)	90 (70–15)

^a^Shown values are medians. Range is shown in brackets

### Mercury intoxication score

At baseline, the placebo group had a slightly lower average and median MIS than the other groups (see Table 3). At visit day 15, the majority of all participants experienced a decrease in MIS compared to the screening day. The mean change was − 3.2 points for the 300 mg NBMI arm, − 2.2 for the 100 mg NBMI arm and − 2.5 for the placebo arm, hinting an easing of symptoms in all treatment groups, which stayed constant till day 45. The differences between the treatment arms were assessed based on the ANCOVA methods, including the baseline score as a covariate in the model. The change from baseline is not significantly dependent on the baseline score, which means that the change values are similar, independently from the baseline scores.

The average differences in MIS change between the NBMI groups and the placebo group on visit day 15 amounts to − 0.69 (95%-CI: -1.80–0.43, not significant) for the 300 mg dose group and 0.19 (95%-CI: -0.90-1.28, not significant) for the 100 mg dose group. See the Appendix for the single score items.

A sensitivity analysis based on the PP analysis set resulted in similar values.

### Neuro-motoric test battery (CATSYS)

In the CATSYS test, the left and right hand were evaluated and analyzed separately. The tremor intensity was additionally statistically tested for differences between the treatment arms and the placebo arm.

#### Tremor intensity

The different treatment groups were compared for their results in tremor intensity. The Wilcoxon–Mann–Whitney test was used, because the assumption of normality was violated by some outliers. The examination of the left-hand tremor intensity indicated a decrease of tremor in the 100 mg NBMI group compared to placebo (two-sided *p*-value = 0.053), but the decrease was less distinct in the 300 mg NBMI group compared to placebo (not significant). The sensitivity analysis with the PP subset led to similar results. The assessment of the right-hand tremor intensity did not indicate any statistically evident differences between the treatment arms. This finding was supported by the sensitivity analysis with the per protocol subset.

#### CATSYS performance indices

The interpretation of the performance indices was inconclusive, since some of the tests showed results in contrast to the expectations. In referral to the CATSYS 200 user’s manual, the calculated indices for reaction time, the rhythmic test, the max frequency test and the median index for tremor would signify a performance above average. The CATSYS Index is the average of all performance indices and given in Table 3.

### Mental and physical fatigue scores

#### Physical fatigue score

**Fig. 3:**
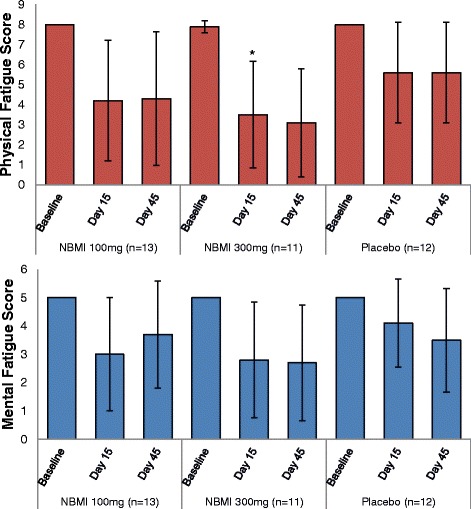
Overview of mean fatigue scores and change over follow up. The bars show the physical and mental fatigue scores at baseline, Day 15 and Day 45 stratified by treatment arm. The data is expressed as mean ± SD, the asterisk indicate statistical difference to the placebo group at *p* < 0.05

The physical fatigue score comprised of 8 items and therefore each subject had a value of 8 at the screening. The median scores are shown in Table 3 and means in Fig. 3. The underlying covariance use for the mixed model repeated measures for the physical fatigue score was a heterogeneous Toeplitz covariance structure. Table 4 shows the mean difference of the scores at day 15 and baseline after adjustment for the covariates of the mixed model of repeated measurements and on the right side the difference of the adjusted means of the treatment groups and placebo. At day 15, a mean drop of 56% in the 300 mg NBMI group, a decrease of 47% in the 100 mg NBMI group and a 30% drop in the placebo group. The mean difference in change in physical fatigue score between the NBMI 300 mg group and placebo was estimated to − 2.1 and statistically significant. The corresponding mean difference in change between the NBMI 100 mg group and placebo was estimated to − 1.4 (see Table 4). The sensitivity analysis with the non-parametric tests led to similar results as presented in this section.

**Table 4:** Statistical analysis of the fatigue scores – adjusted mean changes from baseline and difference from placebo on day 15

Adjusted change from baseline				Difference from Placebo		
Treatment	N	Adj.mean^a^	95%-CI^b^	Adj.mean^a^	95%-CI^b^	*p*-value
Physical fatigue score						
NBMI 100 mg	13	−3.769	[−5.343;-2.195]	−1.353	[−3.433; 0.728]	*.2019*
NBMI 300 mg	11	−4.454	[−6.049;-3.042]	−2.129	[−4.156;-0.102]	.0396*
Placebo	12	−2.417	[−3.777;-1.057]	–		
Mental fatigue score						
NBMI 100 mg	13	−2.000	[−3.048;-0.952]	−1.083	[− 2.432; 0.266]	*.1152*
NBMI 300 mg	11	−2.182	[−3.335;-1.029]	−1.265	[−2.698; 0.167]	*.0833*
Placebo	12	−0.917	[−1.767;-0.067]	–		

The asterisk indicates a statistical difference to the placebo group with *p* < 0.05

^a^Adjusted mean score reduction after fitting the daily measured fatigue scores in a Mixed Model of Repeated Measurements

^b^95%-Confidence interval

#### Mental fatigue score

All the participants had per definition a baseline value of 5. On day 15 it showed a decrease in the 300 mg treatment group to 2.8 ± 2.04 (a drop of 44%), to 3.0 ± 2 in the 100 mg treatment group (a decrease of 40%) and to 4.1 ± 1.56 in the placebo group (a decrease of 18%). The mixed model repeated measures that converged for this analysis was the model with a Toeplitz covariance structure. The model showed mean differences in change to be − 1.3 for 300 mg NBMI vs. placebo and − 1.1 for 100 mg vs. placebo (see Table 4). The sensitivity analysis with the non-parametric tests led to similar results.

### Change from baseline finger-to-nose test

At baseline assessment, tremor was found in 14 (39%) persons. Throughout the groups, the symptom levels did not change in the majority of persons on day 15 compared to baseline. The two treatment groups were tested against placebo using the Wilcoxon–Mann–Whitney test. Since the scale is discrete, an exact method based on the Monte Carlo simulation was used.

The sum of scores was marginally lower for the 300 mg NBMI group compared to the placebo group (0 vs. 1), however this seems to be accidental. In contrast, the sum of scores of the 100 mg NBMI group is slightly higher (2), but this also is not statistically significant.

### Individual change of mercury levels

#### Changes of urine-Hg levels adjusted for creatinine

**Fig. 4:**
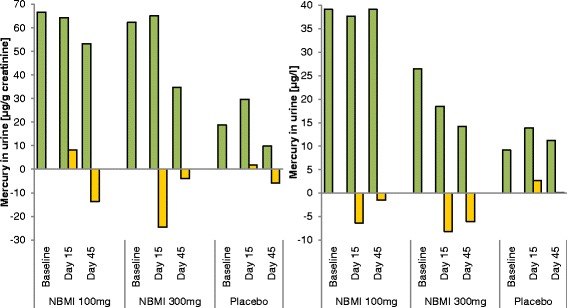
Changes in urine values after follow-up. The creatinine adjusted, and unadjusted urine values are shown stratified by treatment group and examination day. Green bars signify median urine values, yellow bars median changes from baseline values

The inter-individual variability was large for this characteristic (range: 2.63–773.2 μg/g creatinine, see Table 2). The median values and change values are shown in Fig. 4. The 300 mg NBMI group showed a higher average level for mercury in urine, 199.1 μg/g creatinine, at baseline compared to a level of 84.9 μg/g creatinine in the 100 mg NBMI group and 107.8 μg/g creatinine in the placebo group. The distribution of the adjusted mercury in urine values was asymmetrical, thus to compare the change from baseline between the groups the non-parametric Wilcoxon–Mann–Whitney test was used. It was applied for the differences between results of day 15 and day 45 and baseline. The medians of the measured values are shown in the summary in Table 2, while Table 5 shows the median of the individual differences between the values of day 15 and 45 and baseline. The results indicate a lowering of the adjusted mercury in urine levels for the 300 mg NBMI group, although this effect is not statistically. The result of the test of 100 mg NBMI vs. placebo does not show a difference.

**Table 5:** Summary of changes from baseline in urine values

Treatment	Visit	Median	*p*-value*
Mercury in urine [μg/l]			
NBMI 100 mg	Day 15	−6.4 (− 128.6–76.1)	*.892*
	Day 45	−1.5 (− 126.5–22.2)	*.812*
NBMI 300 mg	Day 15	−8.2 (− 240.9–0.8)	*.011*
	Day 45	−6.0 (− 246.9–6.8)	*.069*
Placebo	Day 15	2.7 (−71.9–45.9)	
	Day 45	0.1 (−74.7–49.3)	
Mercury in urine [μg/g creatinine]			
NBMI 100 mg	Day 15	8.2 (− 135.9–122.8)	*.849*
	Day 45	−13.8 (− 140.2–232.5)	*.894*
NBMI 300 mg	Day 15	−24.6 (− 475.5–79.0)	*.091*
	Day 45	−4.0 (− 463.3–1110.1)	*.722*
Placebo	Day 15	1.8 (−407.9–297.1)	
	Day 45	−5.9 (− 453.7–1557.8)	

Results give the median values measured at each time point. Change of baseline is the difference of the day 15/day 45 value and baseline value in each individual. The median of calculated differences is shown

*Wilcoxon-Mann-Whitney test for differences in change from baseline between treatment groups and placebo

#### Changes of urine-Hg unadjusted for creatinine

As for the adjusted urine-Hg, the distribution of the unadjusted urine-Hg was asymmetrical as well and the Wilcoxon–Mann–Whitney test was used for evaluation. The findings suggest a statistical significant difference in change at day 15 for 300 mg NBMI vs. placebo (*p* < 0.05). There is a change in value for the 100 mg group vs. placebo, however, not statistically significant.

#### Change of plasma-Hg

To obtain a normal distribution for the plasma Hg values, the values needed to be log-transformed. Then the test results from the ANCOVA were transformed back after the test. Based on the model, at day 15, there was an average reduction of 13% for the 300 mg NBMI group, for the 100 mg NBMI group there was a 7% decrease and for the placebo group a 9% reduction of mercury levels in plasma from baseline levels. The differences in change between the groups were not statistically significant. The sensitivity analysis of the differences in change of the plasma Hg was done with the Wilcoxon–Mann–Whitney test provided consistent results.

### Adverse events

No serious adverse events (SAE) occurred in this study and there was no discontinuation due to adverse events (AE). The majority of AE were headaches and gastrointestinal events evenly throughout all three groups, so that no event could be clearly related to NBMI. 47 AE in 19 subjects (52.8%) were reported over the treatment and follow-up period similar distributed between the treatment arms. All events are given in a table in the Appendix.

## Discussion

NBMI is a chelating agent, which binds to Hg molecules and renders them nontoxic. Nevertheless, it chemical properties are different from the two common agents, DMSA and DMPS, as it is lipophilic and able to enter cells. NBMI is postulated to access the Hg in deeper departments, while DMSA and DMPS bind to the free Hg and help to excrete it with urine. Thus, it is difficult to compare these compounds by the same standards. As NBMI is mainly excreted through feces, temporarily raised urine Hg values as well as a very fast excretion could not be expected. NBMI forms a very stable chelating complex with Hg and disables it from reacting within the body. A reduced Hg body burden would result in lowering urine levels and a relieving of symptoms.

This study was designed as a pilot to gather information about various outcomes and their value for measuring efficacy of NBMI. It is the first trial to assess the efficacy of this new compound. Despite the small sample size, a positive effect could be shown.

The most evident effect of NBMI compared to placebo could be seen on the subjects’ fatigue. In contrast to the 100 mg NBMI dose, the physical fatigue score was improved in the 300 mg NBMI group suggesting a dose-response relationship with a *p*-value < 0.05 for the difference to placebo group. As the sample size of 36 was chosen to show a ‘proof of concept’, these statistically significant results surpassed the expectations. Some of the fatigue score items improved quickly within the first days and the effects persisted to day 45.

Of the biomonitoring outcomes, mercury levels in urine and plasma, only the change in the unadjusted Hg in urine level for the 300 mg group was statistically significant (*p* < 0.05) in comparison to placebo. However, this effect was not as distinct after adjusting the values for creatinine (*p* < 0.1). Evaluation of spontaneous urine samples was challenging due to a broad inter-individual variability and an inter-group difference at baseline. Unfortunately, after randomizing the groups, the baseline urine values of the placebo group were lower than in the other groups. The difference occurred probably due to the small sample size and the broad individual variability. Hence further studies will have to include 24-h urine collection in order to underpin the data on Hg excretion as analyzed in this study. Chemical properties as such as lipophilicity of NBMI also suggest fecal analysis being a valuable tool for quantifying Hg excretion.

Plasma Hg levels did not differ between the analyzed groups. With a high quantity of Hg binding to albumin whole blood analysis would be mandatory in further studies. We waived this analysis as the analytic setup needed was not available at the site, and transportation was not feasible due to technical constraints. Whole blood samples might have been more conclusive.

There was also no evidence of a treatment effect on the MIS. The single MIS items proved to be challenging to compare, since differences between the groups were already apparent at screening. For example, in the 300 mg group 7 persons showed excessive salivation at screening, of which 4 showed an improvement, while there were none with this symptom in the placebo group. Additionally, the MIS was designed as a tool for a quick and easy diagnosis of Hg intoxications in the field and might not be ideal for efficacy assessment. Some of the neurological signs assessed in the MIS developed over years of exposure and could be irreversible. Furthermore*,* the binary coding of the items might be not sensitive enough to describe an improving of the symptoms and a different choice of scaling is advisable. For example: There was an improving in the matchbox test and the pencil tapping test throughout all groups and most participants reached results above the threshold levels at the second assessment. This improvement probably occurred due to a learning effect over repeating the test; therefore, narrower categories would be helpful to determine differences in improvement.

In comparison with another study in a similar setting and DMPS treatment by Drasch et al., the here obtained results are in the same order of magnitude [17]. In both studies, the greatest effect of the chelators showed in the improved fatigue scores. Additionally, in the Mt.Diwata population was a first improvement in neurological symptoms apparent; however, because of ethical restriction there was no placebo control possible. Assuming a similar effect size as in our study, these neurological improvements might also be attributable to a placebo or, with the pencil tapping test, a learning effect.

There was another coincidental observation made by the principle investigator during the study. Some of the men had problems with erectile dysfunction prior to the medication that seemed to improve with NBMI. This is based on oral reports and was not included in the original design; thus, it was not quantified, it will be, however, considered in the following studies.

Further two basic factors should be considered when evaluating the efficacy of the investigational product. As with DMPS, the treatment period of 14 days might be too short to see the improving of symptoms and it could not be expected, that neurological signs and damages developed over several years can be changed greatly in that period [17]. Furthermore, in future studies the dose could be adapted to body weight. The workers tend to be overweight with a mean BMI of 28 and thus might need a higher dose. The best effect on fatigue in the 300 mg group could be seen in participants who have been screened with a lower Hg urine level (less than 100 μg/g creatinine). The phase 1 study for NBMI showed that a daily dose of 600 mg is harmless in humans; therefore, a further dose escalation is possible and might result in a greater effect.

Although the use of mercury is legally prohibited in Ecuador since a couple of month prior to the study, one cannot rule out the possibility, that some of the miners and thus the participants are still using mercury informally, e.g. at their homes. This would result in a difference in ongoing exposure, as some miners are only exposed through old tailings while others handle mercury directly. This could explain the high outliers in urine values at day 45 and the newly occurred ataxia of gait in three participants. A possible solution for this problem would be the hospitalization of the participants for the length of the study to ensure controlled conditions. However, especially in the prospect of a more extensive study, hospital stays for all participants would exceed the local hospitals capacities and the magnify the costs for researching this orphan drug. A different and probably more feasible approach to keep the miners from processing gold ore would be a more restrictive protocol in combination with a payment for the miners’ participation. This would reduce exposure variance within and between the groups and cover the miners’ loss of income for that period, as some might depend on it for making a living.

This study is limited by the small sample size of 36, which needs to be increased to exhaustively prove the effects shown here. The reported improvement should be considered indications and need to be reconfirmed in the next study. Another limitation is the possible difference in ongoing exposure to mercury, which could have led to an underestimation of the effects. Additionally, as mentioned before, the study parameters like length of follow-up, the scale of efficacy variables need to be adjusted to describe any effects.

The strength of this study is in the extensive set of possible outcome variables. The task of proving the efficacy is rather difficult, since there are no standards for assessing it and no guidelines for treating Hg intoxications. It is important to test and define a set of parameters, which can show an improving condition through medication resp. Hg removal, e.g. signs of the neurological damage are probably irreversible and not viable for efficacy assessment. The insights of this study will be valuable in upcoming phases.

Moreover, there is a supply gap for treating Hg intoxication in many countries and there seems to be too little effort to close them. As in ASGM, the Hg exposure unfortunately mostly concerns the poorest [31], thus, the economic interest in improving this situation is probably too low. A chelating agent which is available in ASGM countries could be very helpful. Despite all difficulties, the goal of reducing the health effects of mercury exposure with prevention and treatment is important and much needed [11].

## Conclusion

Although this study was designed with a small sample size to test for feasibility, the gained results with 300 mg NBMI already showed an effect on physical fatigue with statistical significance and there were indications to positive effects on other symptoms, like sleeping problems. Further studies, with a greater sample size, longer follow-up and possibly higher doses or repetitive treatment periods are needed to verify the positive effects and their superiority against placebo. A further approach, including an analysis of feces, is necessary to quantify and prove the mobilization and excretion of mercury by NBMI. Therefore the outcome variables need to be improved to define a more suitable efficacy assessment.

### Additional file

Additional file 1: All analyzed results not provided in the article are given in the additional file. The tables include summaries of basline characteristics, biomonitoring values und scores. (PDF 1984 kb)

## Appendix

### Inclusion criteria

For inclusion in the study, subjects had to fulfil the following criteria:

1. Male or female subjects, age between 18 and 65 years, inclusive.

2. Urine-Hg ≥ 15 μg/l.

3. Mercury intoxication medical score sum ≥ 5 [5] or medical score sum ≥ 3 in combination with at least two of the following additional symptoms; social nervousness/withdrawal, irritability, memory loss, metallic taste, mental- and physical fatigue.

4. Has signed informed consent for participation.

5. Willingness and ability to comply with study procedures, visit schedules, and other instructions regarding the study.

### Exclusion criteria

Subjects could not be entered in the study if any of the following exclusion criteria were fulfilled:

1. A history of any clinically significant disease or disorder which, in the opinion of the investigator, may either put the subjects at risk because of participation in the study, or influence the results or the subject’s ability to participate in the study.
2. Known or a medical history of renal disorder, significant renal failure, or high risk of renal failure.
3. Any clinically significant abnormalities in clinical chemistry or hematology results at the time of screening as judged by the investigator.
4. Known or suspected neurodegenerative disorder including but not limited to stroke, polio, Parkinson’s and Alzheimer’s disease.
5. Known or suspected drug or alcohol abuse.
6. Positive pregnancy test in women.
7. Serious bacterial and chronic viral infection such as human immunodeficiency virus (HIV) or hepatitis virus.
8. History of severe allergy/hypersensitivity or on-going allergy/hypersensitivity, as judged by the investigator or history of hypersensitivity to drugs with a similar chemical structure or class to NBMI.
9. History of allergy/hypersensitivity to bisulphites (e.g. red/white wine).
10. Participation in any other clinical study that included drug treatment within 3 months of the first administration of investigational product.
11. Use of other therapies for mercury intoxication including metal chelators within 3 months.
12. Investigator considers subject unlikely to comply with study procedures, restrictions and requirements.

### Content of the capsules

NBMI capsules (50 and 200 mg) had the following content:

Active compound: NBMI.

Dosage form: Oral capsules.

Excipients: Microcrystalline cellulose, magnesium stearate.

Packaging: Blisters.

Placebo capsules (50 and 200 mg) had the following content:

Active compound: Placebo.

Dosage form: Oral capsules.

Excipients: Microcrystalline cellulose, magnesium stearate.

Packaging: Blisters

### Mercury intoxication score items

At screening, except of excessive salivation (33% of the study population), tremor at work (14%), bluish discoloration (33%) and proteinuria (3%), all symptoms were common (< 50%) among the workers. The distribution of symptoms in these Ecuadorian miners is described in detail in a previous paper [32].

#### Ataxia of gait

All in all, 26 participants (72%) suffered from ataxia of gait at screening. Only 4 showed an improvement on day 15, 2 with 300 mg NBMI and 2 with placebo. From the 10 persons without this sign at screening, 3 (30%) complained about slight or moderate ataxia of gait at day 15, 2 in the 100 mg treatment arm and 1 in 300 mg treatment arm.

#### Bluish discoloration of gingiva

Twelve (33%) subjects showed a discoloration of the gingiva at screening. 2 of those, 1 with 100 mg NBMI and 1 with placebo, showed improvement on day 15.

#### Dysdiadochokinesia

Thirty two (89%) miners showed dysdiadochokinesia at screening, 9 (82%) in the 300 mg group, 12 (92%) in the 100 mg group and 11 (92%) in the placebo group. At day 15, 3 (33%) persons showed improvement in the 300 mg treatment arm, 2 (17%) in the 100 mg treatment arm and 2 (18%) in the placebo arm. In both NBMI treatment groups, one person showed dysdiadochokinesia at day 15, but not at screening.

#### Excessive salivation

A total of 12 participants showed excessive salivation at screening, all of them were randomized into the NBMI treatment arms, 7 (63%) into the 300 mg dose group and 5 (38%) into the 100 mg dose group. At visit day 15, of the workers taking in 300 mg NBMI only 3 (27%) remained to show excessive salivation, which signifies a decrease of 57%, while in the 100 mg NBMI group 3 (23%) persons still showed this symptom, a decrease of 40%. Since no one in the placebo group suffered from excessive salivation at baseline, no comparison of the rate of improvement was possible.

#### Heel-to-shin test

Of all participants, 32 (89%) had trouble doing the heel-to-shin test. Similar proportions showed symptom improvement with 300 mg NBMI (4 persons; 44%) and placebo (5 persons; 45%), whereas the 100 mg group showed less improvement (2 persons; 17%)

#### Matchbox test

At screening 33 (92%) participants needed > 18 s for putting all the matches into the box. 58% of these could do the same test in ≤18 s at day 15. This progress was found equally in all groups, a marginally higher proportion improved in the placebo group.

#### Pencil tapping

In total 27 subjects (75%) had ≤45 taps in the pencil-tapping test at screening. Of these 89% showed an improvement to > 45 taps at day 15. This proportion is similar in both treatment groups and the placebo group.

#### Proteinuria

Only one subject showed proteinuria at screening, but did not anymore on day 15 after the 300 mg NBMI treatment.

#### Sleeping problems at night

In total, 33 (92%) subjects reported sleeping problems at screening. In the 300 mg group 9 (82%) participants complained about this symptom, while on day 15 only 2 (18%) remained suffering from it, a decrease of 78%. The 100 mg treatment group similarly showed a decrease of 77%: all 13 participants had sleeping problems at screening, while only 3 (23%) remained on day 15. In the placebo group, of 11 (91%) workers had sleeping problems at baseline and 6 (50%) on day 15, a 45% reduction.

#### Tremor at work

Five (14%) participants complained about tremor at work at baseline, all of them belonged to the treatment groups. In the 300 mg-treatment arm, at the screening 3 participants perceived tremor, while at visit day 15 none of them had any tremor at work. Of the 2 persons in the 100 mg NBMI group, one did not show his symptom at visit day 15. No comparison with the placebo group was possible.

**Table 6:** Variables of the laboratory safety assessments

Clinical chemistry	Hematology	Urine analysis
Serum (S)-Alanine aminotransferase (ALT)	Blood (B)-Hemoglobin (Hb)	Urine (U)-Bilirubin
S-Alkaline phosphatase (ALP)	Hematocrit	U-Creatinine
S-Aspartate aminotransferase (AST)	B-Leucocyte differential count (absolute)	U-Glucose
S-Albumin	B-Leucocyte count	U-Protein
S- Bilirubin	Mean cellular volume (MCV)	U-Hb/erythrocytes
S-Chloride	Mean corpuscular hemoglobin content (MCH)	U-Ketone
S-Cholesterol	Mean corpuscular hemoglobin concentration (MCHC)	U-Leucocytes
S-Creatinine	B-Platelet count	U-Nitrite
S-CRP	Reticulocytes	U- Urobilinogen
S-Gamma glutamyl transferase (GGT)		pH
S-Glucose		Specific gravity
S-LDH		U-Pregnancy test
S-Phosphate		U-Drug screen
S-Potassium		
S-Sodium		
S-TSH, T3, T4		
S- Ca^2+^, Mg^2+^, Zn^2+^, Cu^2+^, Fe^2+^ and Se^2+^*		
Urea (blood urea nitrogen)		

*Mercury binds to selenium with extraordinarily high affinity. Available evidence indicates that assessments of mercury exposure and tissue levels need to consider selenium intakes and tissue distributions to provide meaningful risk evaluations. Therefore, plasma Se^2+^ was analyzed to further characterize the study population and susceptibility to the mercury exposure

**Table 7:** Summary of subjects with any AE by preferred term (safety set)

	Number (%) of subjects			
Preferred term	NBMI 100 mg (*n* = 13)	NBMI 300 mg (*n* = 11)	Placebo (*n* = 12)	Total (*n* = 36)
Number of subjects with any AE*	6 (46.2%)	5 (45.5%)	8 (66.7%)	19 (52.8%)
Headache	5 (38.5%)	3 (27.3%)	4 (33.3%)	12 (33.3%)
Abdominal pain upper	1 (7.7%)	1 (9.1%)	2 (16.7%)	4 (11.1%)
Abdominal pain		1 (9.1%)	2 (16.7%)	3 (8.3%)
Diarrhoea	1 (7.7%)		1 (8.3%)	2 (5.6%)
Somnolence	1 (7.7%)		1 (8.3%)	2 (5.6%)
Animal bite		1 (9.1%)		1 (2.8%)
Arthralgia			1 (8.3%)	1 (2.8%)
Back pain			1 (8.3%)	1 (2.8%)
Ear pain	1 (7.7%)			1 (2.8%)
Gastroenteritis	1 (7.7%)			1 (2.8%)
Ingrowing nail	1 (7.7%)			1 (2.8%)
Insomnia			1 (8.3%)	1 (2.8%)
Lip dry			1 (8.3%)	1 (2.8%)
Oropharyngeal pain			1 (8.3%)	1 (2.8%)
Vertigo			1 (8.3%)	1 (2.8%)

## Abbreviations

AE: Adverse effect
ASGM: Artisanal small-scale gold mining
DMPS: 2,3-Dimercapto-1-propanesulfonate
DMSA: Dimercaptosuccinic acid
Hg: Mercury
ICF: Informed consent form
IRB: Institutional review board
MIS: Medical intoxication score
mITT: modified intention to treat analysis
NBMI: N,N‘bis-(2-mercaptoethyl)isophthalamide
PP: per protocol analysis
