# Supplementary material for: Efficacy of N,N‘bis-(2-mercaptoethyl) isophthalamide on mercury intoxication: a randomized controlled trial
Source: Environ Health. 2018 Feb 14;17:15. doi: 10.1186/s12940-018-0358-1 (PMC5813329; doi:10.1186/s12940-018-0358-1)
Supplement: Supplementary file 1 — All analyzed results not provided in the article are given in the additional file. The tables include summaries of basline characteristics, biomonitoring values und scores. (PDF 1984 kb) [file 12940_2018_358_MOESM1_ESM.pdf]

Table D1.1 Subject disposition (All randomised patients)

**Disposition**	**NBMI100mg (n=13)**	**NBMI300mg (n=11)**	**Placebo (n=12)**	**Total (n=36)**
**Subjects enrolled**				36
**Subjects randomised**	13 (100.0%)	11 (100.0%)	12 (100.0%)	36 (100.0%)
Subjects not randomised				0
**Patients randomised and treated**	13 (100.0%)	11 (100.0%)	12 (100.0%)	36 (100.0%)
**Patients who completed study**	13 (100.0%)	11 (100.0%)	12 (100.0%)	36 (100.0%)

Executed script: \\SDS-SERVER2\SAS$\SDS3\Projects\STATISTICS DRUG DEV\NBMI4011 - CTI002\Analyser\Script\SAS\T_ds001.sas, on: 23MAY2016:14:05, by: maria.lidsell
Version of source data: nbmi_end.sas7bdat, last modified: 24FEB16:23:13, status: Final

Table D2.1 Analysis set populations (All randomised patients)

**Population**	**NBMI100mg (n=13)**	**NBMI300mg (n=11)**	**Placebo (n=12)**	**Total (n=36)**
All subjects				36
All randomised patients	13 (100.0%)	11 (100.0%)	12 (100.0%)	36 (100.0%)
Safety set	13 (100.0%)	11 (100.0%)	12 (100.0%)	36 (100.0%)
Full analysis set	13 (100.0%)	11 (100.0%)	12 (100.0%)	36 (100.0%)
Per protocol population	13 (100.0%)	10 (90.9%)	9 (75.0%)	32 (88.9%)
PK analysis set	13 (100.0%)	10 (90.9%)		23 (63.9%)

Executed script: \\SDS-SERVER2\SAS$\SDS3\Projects\STATISTICS DRUG DEV\NBMI4011 - CTI002\Analyser\Script\SAS\T_ds002.sas, on: 23MAY2016:14:05, by: maria.lidsell
Version of source data: nbmi_end.sas7bdat, last modified: 24FEB16:23:13, status: Final

Table D3.1 Demographic characteristics (All randomised patients)

**Variable**	**Category**	**NBMI100mg (n=13)**	**NBMI300mg (n=11)**	**Placebo (n=12)**	**Total (n=36)**
Age (years)	n	13 (100.0%)	11 (100.0%)	12 (100.0%)	36 (100.0%)
	Mean	43.6	38.5	33.3	38.6
	SD	10.23	10.48	8.25	10.37
	Median	47	39	32.5	39.5
	Min	22	26	19	19
	Max	56	59	47	59
Sex	n	13 (100.0%)	11 (100.0%)	12 (100.0%)	36 (100.0%)
	MALE	13 (100.0%)	11 (100.0%)	12 (100.0%)	36 (100.0%)
Ethnic Origin	n	13 (100.0%)	11 (100.0%)	12 (100.0%)	36 (100.0%)
	HISPANIC	13 (100.0%)	11 (100.0%)	12 (100.0%)	36 (100.0%)

Executed script: \\SDS-SERVER2\SAS$\SDS3\Projects\STATISTICS DRUG DEV\NBMI4011 - CTI002\Analyser\Script\SAS\T_dm001.sas, on: 23MAY2016:14:05, by: maria.lidsell
Version of source data: nbmi_dem.sas7bdat, last modified: 24FEB16:23:13, status: Final

Table D4.1 Subject characteristics - work exposure and fish consumption (All randomised patients)

Variable	Category	NBMI100mg (n=13)	NBMI300mg (n=11)	Placebo (n=12)	Total (n=36)
Work Exposure, Living, years	n	13 (100.0%)	11 (100.0%)	12 (100.0%)	36 (100.0%)
	Mean	25.8	18.1	27.6	24.1
	SD	16.42	14.59	13.71	15.13
	Median	22	15	31	26
	Min	3	1.5	1	1
	Max	54	50	47	54
Work Exposure, Working with mercury, yea	n	13 (100.0%)	11 (100.0%)	12 (100.0%)	36 (100.0%)
	Mean	12.3	8.7	14	11.8
	SD	6.59	8.19	13.32	9.72
	Median	15	5	12	10
	Min	1	1.5	0.5	0.5
	Max	20	30	40	40
Work Exposure, Smelting amalgam to recover the gold, Yes-No	n	13 (100.0%)	11 (100.0%)	12 (100.0%)	36 (100.0%)
	Y	13 (100.0%)	7 (63.6%)	9 (75.0%)	29 (80.6%)
	N		4 (36.4%)	3 (25.0%)	7 (19.4%)
Work Exposure, Handling mercury to extract the gold from the ore, but no smelting, Yes-No	n	13 (100.0%)	11 (100.0%)	12 (100.0%)	36 (100.0%)
	Y	6 (46.2%)	4 (36.4%)	3 (25.0%)	13 (36.1%)
	N	7 (53.8%)	7 (63.6%)	9 (75.0%)	23 (63.9%)
Work Exposure, Smelting gold / gold buyer, Yes-No	n	13 (100.0%)	11 (100.0%)	12 (100.0%)	36 (100.0%)
	Y		2 (18.2%)	2 (16.7%)	4 (11.1%)
	N	13 (100.0%)	9 (81.8%)	10 (83.3%)	32 (88.9%)
Work Exposure, Another job or no job, Yes-No	n	13 (100.0%)	11 (100.0%)	12 (100.0%)	36 (100.0%)
	Y		2 (18.2%)	1 (8.3%)	3 (8.3%)
	N	13 (100.0%)	9 (81.8%)	11 (91.7%)	33 (91.7%)
Fish Consumption	n	13 (100.0%)	11 (100.0%)	12 (100.0%)	36 (100.0%)
	AT LEAST ONCE A WEEK	9 (69.2%)	6 (54.5%)	8 (66.7%)	23 (63.9%)
	NEVER OR LESS THAN ONCE A WEEK	4 (30.8%)	5 (45.5%)	4 (33.3%)	13 (36.1%)

Executed script: \\SDS-SERVER2\SAS$\SDS3\Projects\STATISTICS DRUG DEV\NBMI4011 - CTI002\Analyser\Script\SAS\T_dm002.sas, on: 23MAY2016:14:06, by: maria.lidsell
Version of source data: nbmi_dem.sas7bdat, last modified: 24FEB16:23:13, status: Final

Table D5.1 Summary of past medical history (All randomised patients)

	Number (%) of patients			
**Preferred term**	**NBMI100mg (n=13)**	**NBMI300mg (n=11)**	**Placebo (n=12)**	**Total (n=36)**
Number of patients with any past medical history	6 (46.2%)	4 (36.4%)	5 (41.7%)	15 (41.7%)
GASTRITIS	1 (7.7%)	1 (9.1%)	1 (8.3%)	3 (8.3%)
APPENDICECTOMY	1 (7.7%)	1 (9.1%)		2 (5.6%)
NEPHROLITHIASIS	1 (7.7%)		1 (8.3%)	2 (5.6%)
PNEUMONIA	1 (7.7%)	1 (9.1%)		2 (5.6%)
ACCIDENT	1 (7.7%)			1 (2.8%)
ALCOHOL POISONING	1 (7.7%)			1 (2.8%)
CHOLECYSTECTOMY		1 (9.1%)		1 (2.8%)
CRANIOCEREBRAL INJURY			1 (8.3%)	1 (2.8%)
DENGUE FEVER	1 (7.7%)			1 (2.8%)
GASTRIC ULCER			1 (8.3%)	1 (2.8%)
GASTROENTERITIS			1 (8.3%)	1 (2.8%)
HEPATITIS			1 (8.3%)	1 (2.8%)
HEPATITIS A	1 (7.7%)			1 (2.8%)
LOWER LIMB FRACTURE		1 (9.1%)		1 (2.8%)
RECTAL PROLAPSE		1 (9.1%)		1 (2.8%)
THROMBOCYTOPENIA			1 (8.3%)	1 (2.8%)
YELLOW FEVER	1 (7.7%)			1 (2.8%)

Executed script: \\SDS-SERVER2\SAS$\SDS3\Projects\STATISTICS DRUG DEV\NBMI4011 - CTI002\Analyser\Script\SAS\T_mh001.sas, on: 23MAY2016:14:06, by: maria.lidsell
Version of source data: nbmi_ma1.sas7bdat, last modified: 24FEB16:23:13, status: Final
Dictionary version: MedDRA 18.1

Table R1.1 Summary of urine Hg at Screening (All randomised patients)

Variable	Category	NBMI100mg (n=13)	NBMI300mg (n=11)	Placebo (n=12)	Total (n=36)
Biomonitoring Hg Urine, Result	n	13 (100.0%)	11 (100.0%)	12 (100.0%)	36 (100.0%)
	Mean	54.2	60.7	43.9	52.8
	SD	31.91	52.13	39.21	40.7
	Median	46	48	32	35.5
	Min	25	15	16	15
	Max	138	163	134	163

Executed script: \\SDS-SERVER2\SAS$\SDS3\Projects\STATISTICS DRUG DEV\NBMI4011 - CTI002\Analyser\Script\SAS\T_hgu001.sas, on: 23MAY2016:14:04, by: maria.lidsell
Version of source data: nbmi_bi2.sas7bdat, last modified: 24FEB16:23:13, status: Final

Table R2.1 Summary of chronic mercury intoxication symptoms at Screening (All randomised patients)

Variable	Category*	NBMI100mg (n=13)	NBMI300mg (n=11)	Placebo (n=12)	Total (n=36)
1. Metallic taste	n	9 (69.2%)	7 (63.6%)	8 (66.7%)	24 (66.7%)
	N	5 (38.5%)	6 (54.5%)	6 (50.0%)	17 (47.2%)
	YES	4 (30.8%)	1 (9.1%)	2 (16.7%)	7 (19.4%)
2. Irritability	n	9 (69.2%)	7 (63.6%)	8 (66.7%)	24 (66.7%)
	ABSENT	2 (15.4%)	1 (9.1%)		3 (8.3%)
	SLIGHT	6 (46.2%)	3 (27.3%)	6 (50.0%)	15 (41.7%)
	MODERATE	1 (7.7%)	3 (27.3%)	2 (16.7%)	6 (16.7%)
3. Social nervousness and/or withdrawal	n	9 (69.2%)	7 (63.6%)	8 (66.7%)	24 (66.7%)
	ABSENT	4 (30.8%)	3 (27.3%)	4 (33.3%)	11 (30.6%)
	SLIGHT	4 (30.8%)	2 (18.2%)	4 (33.3%)	10 (27.8%)
	MODERATE	1 (7.7%)	2 (18.2%)		3 (8.3%)
4. Memory loss	n	9 (69.2%)	7 (63.6%)	8 (66.7%)	24 (66.7%)
	ABSENT	5 (38.5%)	3 (27.3%)	4 (33.3%)	12 (33.3%)
	SLIGHT	4 (30.8%)	2 (18.2%)	3 (25.0%)	9 (25.0%)
	MODERATE		2 (18.2%)	1 (8.3%)	3 (8.3%)
5. Mental fatigue	n	9 (69.2%)	7 (63.6%)	8 (66.7%)	24 (66.7%)
	ABSENT	4 (30.8%)	2 (18.2%)	3 (25.0%)	9 (25.0%)
	SLIGHT	3 (23.1%)	2 (18.2%)	3 (25.0%)	8 (22.2%)
	MODERATE	2 (15.4%)	3 (27.3%)	2 (16.7%)	7 (19.4%)
6. Physical fatigue	n	9 (69.2%)	7 (63.6%)	8 (66.7%)	24 (66.7%)
	ABSENT	4 (30.8%)	2 (18.2%)	3 (25.0%)	9 (25.0%)
	SLIGHT	2 (15.4%)	1 (9.1%)	1 (8.3%)	4 (11.1%)
	MODERATE	3 (23.1%)	4 (36.4%)	4 (33.3%)	11 (30.6%)
Chronic mercury intoxication symptoms, sum	n	9 (69.2%)	7 (63.6%)	8 (66.7%)	24 (66.7%)
	Mean	3.3	3.6	3.5	3.5
	SD	1.73	1.27	1.31	1.41

Variable	Category*	NBMI100mg (n=13)	NBMI300mg (n=11)	Placebo (n=12)	Total (n=36)
	Median	3	4	3.5	3.5
	Min	1	1	1	1
	Max	6	5	5	6

Executed script: \\SDS-SERVER2\SAS$\SDS3\Projects\STATISTICS DRUG DEV\NBMI4011 - CTI002\Analyser\Script\SAS\T_mi001.sas, on: 23MAY2016:14:04, by: maria.lidsell
Version of source data: nbmi_ch2.sas7bdat, last modified: 24FEB16:23:13, status: Final
*) Screening scores are counted according to: No=0, Yes=1, Absent=0, Slight=1, Moderate=1.

Table R3.1.1 Summary of physical and mental fatigue scores at Screening - Total scores (All randomised patients)

Variable*	Category	NBMI100mg (n=13)	NBMI300mg (n=11)	Placebo (n=12)	Total (n=36)
Physical Fatigue Score, Total	n	9 (69.2%)	7 (63.6%)	8 (66.7%)	24 (66.7%)
	Mean	3.4	3	3.6	3.4
	SD	3.61	1.83	2.72	2.78
	Median	3	3	4	3.5
	Min	0	0	0	0
	Max	11	5	7	11
Mental Fatigue Score, Total	n	9 (69.2%)	7 (63.6%)	8 (66.7%)	24 (66.7%)
	Mean	1.7	1.7	2.4	1.9
	SD	1.8	1.6	1.41	1.59
	Median	1	1	2.5	1.5
	Min	0	0	1	0
	Max	5	5	5	5

Executed script: \\SDS-SERVER2\SAS$\SDS3\Projects\STATISTICS DRUG DEV\NBMI4011 - CTI002\Analyser\Script\SAS\T_fs001.sas, on: 23MAY2016:14:04, by: maria.lidsell
Version of source data: nbmi_ph4.sas7bdat, last modified: 24FEB16:23:13, status: Final
*) Total fatigue score sum is not calculated for Screening.

Table R3.2.1 Summary of physical and mental fatigue scores at Screening - Items (All randomised patients)

Variable	Category*	NBMI100mg (n=13)	NBMI300mg (n=11)	Placebo (n=12)	Total (n=36)
Physical Fatigue Score, tired	n	9 (69.2%)	7 (63.6%)	8 (66.7%)	24 (66.7%)
	same as usual	3 (23.1%)	1 (9.1%)	2 (16.7%)	6 (16.7%)
	worse than usual	5 (38.5%)	6 (54.5%)	5 (41.7%)	16 (44.4%)
	much worse than usual	1 (7.7%)		1 (8.3%)	2 (5.6%)
Physical Fatigue Score, need to rest	n	9 (69.2%)	7 (63.6%)	8 (66.7%)	24 (66.7%)
	same as usual	5 (38.5%)	5 (45.5%)	3 (25.0%)	13 (36.1%)
	worse than usual	3 (23.1%)	2 (18.2%)	5 (41.7%)	10 (27.8%)
	much worse than usual	1 (7.7%)			1 (2.8%)
Physical Fatigue Score, feel sleepy or drowsy	n	9 (69.2%)	7 (63.6%)	8 (66.7%)	24 (66.7%)
	same as usual	5 (38.5%)	4 (36.4%)	4 (33.3%)	13 (36.1%)
	worse than usual	3 (23.1%)	3 (27.3%)	4 (33.3%)	10 (27.8%)
	much worse than usual	1 (7.7%)			1 (2.8%)
Physical Fatigue Score, no longer start anything	n	9 (69.2%)	7 (63.6%)	8 (66.7%)	24 (66.7%)
	same as usual	8 (61.5%)	5 (45.5%)	6 (50.0%)	19 (52.8%)
	worse than usual	1 (7.7%)	2 (18.2%)	2 (16.7%)	5 (13.9%)
Physical Fatigue Score, always lack energy	n	9 (69.2%)	7 (63.6%)	8 (66.7%)	24 (66.7%)
	same as usual	6 (46.2%)	4 (36.4%)	3 (25.0%)	13 (36.1%)
	worse than usual	2 (15.4%)	3 (27.3%)	5 (41.7%)	10 (27.8%)
	much worse than usual	1 (7.7%)			1 (2.8%)
Physical Fatigue Score, have less strength in your muscles	n	9 (69.2%)	7 (63.6%)	8 (66.7%)	24 (66.7%)
	same as usual	6 (46.2%)	4 (36.4%)	6 (50.0%)	16 (44.4%)
	worse than usual	3 (23.1%)	3 (27.3%)	2 (16.7%)	8 (22.2%)
Physical Fatigue Score, feel weak	n	9 (69.2%)	7 (63.6%)	8 (66.7%)	24 (66.7%)
	same as usual	5 (38.5%)	5 (45.5%)	6 (50.0%)	16 (44.4%)
	worse than usual	3 (23.1%)	2 (18.2%)	2 (16.7%)	7 (19.4%)
	much worse than usual	1 (7.7%)			1 (2.8%)
Physical Fatigue Score, start things without difficulties, but get weak as you go on	n	9 (69.2%)	7 (63.6%)	8 (66.7%)	24 (66.7%)
	same as usual	8 (61.5%)	7 (63.6%)	6 (50.0%)	21 (58.3%)
	worse than usual	1 (7.7%)		2 (16.7%)	3 (8.3%)
Mental Fatigue Score, problems concentrating	n	9 (69.2%)	7 (63.6%)	8 (66.7%)	24 (66.7%)
	same as usual	7 (53.8%)	6 (54.5%)	4 (33.3%)	17 (47.2%)
	worse than usual	2 (15.4%)	1 (9.1%)	4 (33.3%)	7 (19.4%)
Mental Fatigue Score, problems thinking clearly	n	9 (69.2%)	7 (63.6%)	8 (66.7%)	24 (66.7%)
	same as usual	8 (61.5%)	6 (54.5%)	5 (41.7%)	19 (52.8%)
	worse than usual	1 (7.7%)	1 (9.1%)	3 (25.0%)	5 (13.9%)
Mental Fatigue Score, problems to find correct words when you speak	n	9 (69.2%)	7 (63.6%)	8 (66.7%)	24 (66.7%)
	same as usual	5 (38.5%)	4 (36.4%)	4 (33.3%)	13 (36.1%)

Variable	Category*	NBMI100mg (n=13)	NBMI300mg (n=11)	Placebo (n=12)	Total (n=36)
	worse than usual	4 (30.8%)	3 (27.3%)	4 (33.3%)	11 (30.6%)
Mental Fatigue Score, problems with eyestrain	n	9 (69.2%)	7 (63.6%)	8 (66.7%)	24 (66.7%)
	same as usual	5 (38.5%)	3 (27.3%)	5 (41.7%)	13 (36.1%)
	worse than usual	4 (30.8%)	4 (36.4%)	3 (25.0%)	11 (30.6%)
Mental Fatigue Score, problems with memory	n	9 (69.2%)	7 (63.6%)	8 (66.7%)	24 (66.7%)
	same as usual	6 (46.2%)	4 (36.4%)	3 (25.0%)	13 (36.1%)
	worse than usual	2 (15.4%)	3 (27.3%)	5 (41.7%)	10 (27.8%)
	much worse than usual	1 (7.7%)			1 (2.8%)

Executed script: \\SDS-SERVER2\SAS$\SDS3\Projects\STATISTICS DRUG DEV\NBMI4011 - CTI002\Analyser\Script\SAS\T_fs001.sas, on: 23MAY2016:14:04, by: maria.lidsell
Version of source data: nbmi_ph4.sas7bdat, last modified: 24FEB16:23:13, status: Final
*) Screening scores are counted according to: same as usual=0, worse than usual=1, much worse than usual=2.

Table R4.1 Summary of abnormal physical examination findings at Screening (All randomised patients)

	Number (%) of patients			
**Area of the examination**	**NBMI100mg (n=13)**	**NBMI300mg (n=11)**	**Placebo (n=12)**	**Total (n=36)**
Number of patients with any abnormal finding	10 (76.9%)	9 (81.8%)	10 (83.3%)	29 (80.6%)
Neurological examination	10 (76.9%)	8 (72.7%)	9 (75.0%)	27 (75.0%)
General Condition	7 (53.8%)	3 (27.3%)	4 (33.3%)	14 (38.9%)
Abdomen	6 (46.2%)	4 (36.4%)	1 (8.3%)	11 (30.6%)
Other	2 (15.4%)	1 (9.1%)	3 (25.0%)	6 (16.7%)
Cardiovascular system	1 (7.7%)	1 (9.1%)		2 (5.6%)
Lungs	1 (7.7%)			1 (2.8%)

Executed script: \\SDS-SERVER2\SAS$\SDS3\Projects\STATISTICS DRUG DEV\NBMI4011 - CTI002\Analyser\Script\SAS\T_pe001.sas, on: 23MAY2016:14:04, by: maria.lidsell
Version of source data: nbmi_ph2.sas7bdat, last modified: 24FEB16:23:13, status: Final

Table R5.1 Summary of vital signs at Screening (All randomised patients)

Variable	Category	NBMI100mg (n=13)	NBMI300mg (n=11)	Placebo (n=12)	Total (n=36)
Diastolic BP (mmHg)	n	13 (100.0%)	11 (100.0%)	12 (100.0%)	36 (100.0%)
	Mean	71.5	72.2	70.1	71.2
	SD	8.76	10.69	11.53	10.08
	Median	70	73	70	70
	Min	58	57	50	50
	Max	84	90	86	90
Systolic BP (mmHg)	n	13 (100.0%)	11 (100.0%)	12 (100.0%)	36 (100.0%)
	Mean	116.5	113.5	113.7	114.6
	SD	7.8	18.97	14.61	13.88
	Median	120	110	112.5	111
	Min	105	90	90	90
	Max	130	160	135	160
Heart rate (bpm)	n	13 (100.0%)	11 (100.0%)	12 (100.0%)	36 (100.0%)
	Mean	75.6	78.9	70.5	74.9
	SD	4.68	10.23	5.71	7.72
	Median	76	76	68	74.5
	Min	66	64	64	64
	Max	82	99	82	99
Body weight (kg)	n	13 (100.0%)	11 (100.0%)	12 (100.0%)	36 (100.0%)
	Mean	75.1	79.8	82.8	79.1
	SD	14.96	13.69	10.7	13.3
	Median	72	78	81.25	78.5
	Min	54.5	63	62	54.5
	Max	103	107	104	107
Height (cm)	n	13 (100.0%)	11 (100.0%)	12 (100.0%)	36 (100.0%)
	Mean	162.2	169.3	167.9	166.3
	SD	6.12	9.06	6.43	7.7
	Median	164	167	168	166
	Min	151	161	157	151
	Max	174	194	181	194
Body mass index (kg/m2) from height at s	n	13 (100.0%)	11 (100.0%)	12 (100.0%)	36 (100.0%)
	Mean	28.5	27.7	29.5	28.6
	SD	4.78	3.23	4.56	4.23
	Median	29.38	28.43	29.985	29.31
	Min	21.77	23.14	18.92	18.92
	Max	36.36	34.42	39.14	39.14

Executed script: \\SDS-SERVER2\SAS$\SDS3\Projects\STATISTICS DRUG DEV\NBMI4011 - CTI002\Analyser\Script\SAS\T_vs001.sas, on: 23MAY2016:14:04, by: maria.lidsell
Version of source data: nbmi_vi1.sas7bdat, last modified: 24FEB16:23:15, status: Final

Table R6.1 Summary of medical history ongoing at Screening (All randomised patients)

	Number (%) of patients			
**Preferred term**	**NBMI100mg (n=13)**	**NBMI300mg (n=11)**	**Placebo (n=12)**	**Total (n=36)**
Number of patients with any medical history ongoing at Screening	13 (100.0%)	11 (100.0%)	12 (100.0%)	36 (100.0%)
CEREBELLAR SYNDROME	12 (92.3%)	8 (72.7%)	10 (83.3%)	30 (83.3%)
SLEEP DISORDER	13 (100.0%)	6 (54.5%)	11 (91.7%)	30 (83.3%)
ATAXIA	9 (69.2%)	7 (63.6%)	9 (75.0%)	25 (69.4%)
IRRITABILITY	4 (30.8%)	4 (36.4%)	5 (41.7%)	13 (36.1%)
FATIGUE	3 (23.1%)	4 (36.4%)	4 (33.3%)	11 (30.6%)
GINGIVAL DISCOLOURATION	5 (38.5%)	4 (36.4%)	2 (16.7%)	11 (30.6%)
OBESITY	5 (38.5%)	2 (18.2%)	3 (25.0%)	10 (27.8%)
SALIVARY HYPERSECRETION	5 (38.5%)	5 (45.5%)		10 (27.8%)
AMNESIA	3 (23.1%)	3 (27.3%)	3 (25.0%)	9 (25.0%)
MENTAL FATIGUE	1 (7.7%)	4 (36.4%)	4 (33.3%)	9 (25.0%)
HEADACHE	5 (38.5%)		2 (16.7%)	7 (19.4%)
SOCIAL AVOIDANT BEHAVIOUR	2 (15.4%)	2 (18.2%)	2 (16.7%)	6 (16.7%)
DYSGEUSIA	2 (15.4%)	1 (9.1%)	1 (8.3%)	4 (11.1%)
TREMOR	2 (15.4%)	2 (18.2%)		4 (11.1%)
BACK PAIN	1 (7.7%)	1 (9.1%)		2 (5.6%)
HEPATIC STEATOSIS	1 (7.7%)	1 (9.1%)		2 (5.6%)
WEIGHT INCREASED	1 (7.7%)		1 (8.3%)	2 (5.6%)
ABDOMINAL HERNIA	1 (7.7%)			1 (2.8%)
ARTHRALGIA		1 (9.1%)		1 (2.8%)
CHRONIC OBSTRUCTIVE PULMONARY DISEASE	1 (7.7%)			1 (2.8%)
DIABETES MELLITUS	1 (7.7%)			1 (2.8%)
ELECTROCARDIOGRAM ST SEGMENT ELEVATION			1 (8.3%)	1 (2.8%)
GASTRITIS		1 (9.1%)		1 (2.8%)
HYPERTENSION		1 (9.1%)		1 (2.8%)
LACTOSE INTOLERANCE			1 (8.3%)	1 (2.8%)
MIGRAINE	1 (7.7%)			1 (2.8%)
PROSTATITIS		1 (9.1%)		1 (2.8%)
RHINITIS		1 (9.1%)		1 (2.8%)
SINUSITIS	1 (7.7%)			1 (2.8%)
UMBILICAL HERNIA		1 (9.1%)		1 (2.8%)
VENTRICULAR EXTRASYSTOLES	1 (7.7%)			1 (2.8%)

Executed script: \\SDS-SERVER2\SAS$\SDS3\Projects\STATISTICS DRUG DEV\NBMI4011 - CTI002\Analyser\Script\SAS\T_mh002.sas, on: 23MAY2016:14:04, by: maria.lidsell
Version of source data: nbmi_ma1.sas7bdat, last modified: 24FEB16:23:13, status: Final
Dictionary version: MedDRA 18.1

Table DE1.1.1 Change from baseline of Mercury intoxication Medical Score, Medical score sum (calculated) (Full analysis set)

		Result									Change from baseline (Screening visit)								
**Treatment**	**Visit**	**n**	**nmiss**	**Mean**	**SD**	**Q1**	**Median**	**Q3**	**Min**	**Max**	**n**	**nmiss**	**Mean**	**SD**	**Q1**	**Median**	**Q3**	**Min**	**Max**
Total (n=36)	Screening	36 (100%)	0 (0%)	5.9	1.08	5	6	7	4	8									
	Follow-up day 15	36 (100%)	0 (0%)	3.3	1.61	2	3	4.5	0	7	36 (100%)	0 (0%)	-2.6	1.22	-3	-2.5	-2	-5	0
	Follow-up day 45	36 (100%)	0 (0%)	3	1.28	2	3	4	0	5	36 (100%)	0 (0%)	-2.9	1.37	-3.5	-3	-2	-6	0
NBMI100mg (n=13)	Screening	13 (100%)	0 (0%)	6.3	1.18	6	6	7	4	8									
	Follow-up day 15	13 (100%)	0 (0%)	4	1.63	3	4	5	1	7	13 (100%)	0 (0%)	-2.3	1.11	-3	-2	-2	-5	0
	Follow-up day 45	13 (100%)	0 (0%)	3.7	0.95	3	4	4	2	5	13 (100%)	0 (0%)	-2.6	1.39	-3	-3	-2	-5	0
NBMI300mg (n=11)	Screening	11 (100%)	0 (0%)	6.2	0.98	5	6	7	5	8									
	Follow-up day 15	11 (100%)	0 (0%)	3	1.34	2	3	4	1	5	11 (100%)	0 (0%)	-3.2	1.08	-4	-3	-2	-5	-2
	Follow-up day 45	11 (100%)	0 (0%)	3	1.18	2	3	4	1	5	11 (100%)	0 (0%)	-3.2	1.33	-4	-3	-2	-6	-2
Placebo (n=12)	Screening	12 (100%)	0 (0%)	5.3	0.75	5	5	6	4	6									
	Follow-up day 15	12 (100%)	0 (0%)	2.8	1.66	1.5	2.5	4	0	5	12 (100%)	0 (0%)	-2.5	1.38	-3.5	-2.5	-1	-5	-1
	Follow-up day 45	12 (100%)	0 (0%)	2.3	1.37	1	2.5	3.5	0	4	12 (100%)	0 (0%)	-2.9	1.44	-3.5	-3	-2.5	-5	0

Executed script: \\SDS-SERVER2\SAS$\SDS3\Projects\STATISTICS DRUG DEV\NBMI4011 - CTI002\Analyser\Script\SAS\T_ms001.sas, on: 23MAY2016:14:00, by: maria.lidsell
Version of source data: nbmi_ma2.sas7bdat, last modified: 24FEB16:23:13, status: Final

Table DE1.2.1 Change from baseline of Mercury intoxication Medical Score, Match box test, sec (Full analysis set)

		Result									Change from baseline (Screening visit)								
**Treatment**	**Visit**	**n**	**nmiss**	**Mean**	**SD**	**Q1**	**Median**	**Q3**	**Min**	**Max**	**n**	**nmiss**	**Mean**	**SD**	**Q1**	**Median**	**Q3**	**Min**	**Max**
Total (n=36)	Screening	36 (100%)	0 (0%)	22.8	3.83	20	22	25	15	32									
	Follow-up day 15	36 (100%)	0 (0%)	18.3	3.36	16	18	20	13	28	36 (100%)	0 (0%)	-4.5	3.97	-7.5	-4	-2	-14	4
	Follow-up day 45	36 (100%)	0 (0%)	16.8	3.32	14	17	19	11	25	36 (100%)	0 (0%)	-6	3.96	-9	-5	-3	-14	2
NBMI100mg (n=13)	Screening	13 (100%)	0 (0%)	22.2	3.54	20	21	24	15	28									
	Follow-up day 15	13 (100%)	0 (0%)	18.6	2.57	18	19	20	14	23	13 (100%)	0 (0%)	-3.6	3.69	-5	-2	-2	-10	2
	Follow-up day 45	13 (100%)	0 (0%)	16.8	2.39	15	17	19	13	20	13 (100%)	0 (0%)	-5.5	3.6	-7	-5	-3	-14	-1
NBMI300mg (n=11)	Screening	11 (100%)	0 (0%)	22.5	3.62	21	22	25	15	28									
	Follow-up day 15	11 (100%)	0 (0%)	18.7	4.43	15	17	21	14	28	11 (100%)	0 (0%)	-3.8	3.68	-6	-4	-1	-9	4
	Follow-up day 45	11 (100%)	0 (0%)	17.5	4.25	14	17	20	11	25	11 (100%)	0 (0%)	-5.1	3.33	-8	-5	-3	-11	1
Placebo (n=12)	Screening	12 (100%)	0 (0%)	23.5	4.5	19.5	23	26	18	32									
	Follow-up day 15	12 (100%)	0 (0%)	17.4	3.12	15.5	17	18.5	13	25	12 (100%)	0 (0%)	-6.1	4.34	-9.5	-6	-2.5	-14	0
	Follow-up day 45	12 (100%)	0 (0%)	16.1	3.37	13	16	19	11	21	12 (100%)	0 (0%)	-7.4	4.72	-11	-9.5	-4.5	-13	2

Executed script: \\SDS-SERVER2\SAS$\SDS3\Projects\STATISTICS DRUG DEV\NBMI4011 - CTI002\Analyser\Script\SAS\T_ms001.sas, on: 23MAY2016:14:00, by: maria.lidsell
Version of source data: nbmi_ma2.sas7bdat, last modified: 24FEB16:23:13, status: Final

Table DE1.3.1 Change from baseline of Mercury intoxication Medical Score, Pencil tapping test, taps (Full analysis set)

		Result									Change from baseline (Screening visit)								
**Treatment**	**Visit**	**n**	**nmiss**	**Mean**	**SD**	**Q1**	**Median**	**Q3**	**Min**	**Max**	**n**	**nmiss**	**Mean**	**SD**	**Q1**	**Median**	**Q3**	**Min**	**Max**
Total (n=36)	Screening	36 (100%)	0 (0%)	45.1	12.43	40.5	43	46	30	90									
	Follow-up day 15	36 (100%)	0 (0%)	56.5	7.41	52.5	57	60	40	73	36 (100%)	0 (0%)	11.4	13.77	8.5	13	18	-40	30
	Follow-up day 45	36 (100%)	0 (0%)	59.9	7.47	55	60	65.5	44	77	36 (100%)	0 (0%)	14.8	13.91	11	17	23	-34	32
NBMI100mg (n=13)	Screening	13 (100%)	0 (0%)	42.6	15.25	36	41	42	30	90									
	Follow-up day 15	13 (100%)	0 (0%)	54.7	5.47	55	56	58	42	60	13 (100%)	0 (0%)	12.1	15.56	10	16	18	-34	30
	Follow-up day 45	13 (100%)	0 (0%)	58	6.86	56	58	62	44	70	13 (100%)	0 (0%)	15.4	15.62	15	20	23	-32	32
NBMI300mg (n=11)	Screening	11 (100%)	0 (0%)	48.8	14.21	42	43	48	41	90									
	Follow-up day 15	11 (100%)	0 (0%)	56.1	7.19	53	56	61	40	68	11 (100%)	0 (0%)	7.3	16.68	8	13	16	-40	20
	Follow-up day 45	11 (100%)	0 (0%)	60.2	9.69	52	58	67	46	77	11 (100%)	0 (0%)	11.4	17.24	7	14	22	-34	32
Placebo (n=12)	Screening	12 (100%)	0 (0%)	44.4	5.7	40.5	43.5	47.5	37	58									
	Follow-up day 15	12 (100%)	0 (0%)	58.9	9.2	51	59	67	45	73	12 (100%)	0 (0%)	14.5	7.65	7	13	20.5	6	28
	Follow-up day 45	12 (100%)	0 (0%)	61.7	5.76	56.5	61	66.5	53	70	12 (100%)	0 (0%)	17.3	7.79	12.5	17	24	4	29

Executed script: \\SDS-SERVER2\SAS$\SDS3\Projects\STATISTICS DRUG DEV\NBMI4011 - CTI002\Analyser\Script\SAS\T_ms001.sas, on: 23MAY2016:14:00, by: maria.lidsell
Version of source data: nbmi_ma2.sas7bdat, last modified: 24FEB16:23:13, status: Final

Table DE2.1.1 Categorical summary of change from baseline of Mercury intoxication medical score sum - Item 1. Excessive salivation (Full analysis set)

		**Result***			**Change** from baseline (Screening visit)**			
**Treatment**	**Visit**	**n**	**0**	**1**	**n**	**-1**	**0**	**1**
Total (n=36)	Screening	36 (100%)	24 (66.67%)	12 (33.33%)				
	Follow-up day 15	36 (100%)	30 (83.33%)	6 (16.67%)	36 (100%)	6 (16.67%)	30 (83.33%)	
	Follow-up day 45	36 (100%)	31 (86.11%)	5 (13.89%)	36 (100%)	7 (19.44%)	29 (80.56%)	
NBMI100mg (n=13)	Screening	13 (100%)	8 (61.54%)	5 (38.46%)				
	Follow-up day 15	13 (100%)	10 (76.92%)	3 (23.08%)	13 (100%)	2 (15.38%)	11 (84.62%)	
	Follow-up day 45	13 (100%)	11 (84.62%)	2 (15.38%)	13 (100%)	3 (23.08%)	10 (76.92%)	
NBMI300mg (n=11)	Screening	11 (100%)	4 (36.36%)	7 (63.64%)				
	Follow-up day 15	11 (100%)	8 (72.73%)	3 (27.27%)	11 (100%)	4 (36.36%)	7 (63.64%)	
	Follow-up day 45	11 (100%)	8 (72.73%)	3 (27.27%)	11 (100%)	4 (36.36%)	7 (63.64%)	
Placebo (n=12)	Screening	12 (100%)	12 (100%)					
	Follow-up day 15	12 (100%)	12 (100%)		12 (100%)		12 (100%)	
	Follow-up day 45	12 (100%)	12 (100%)		12 (100%)		12 (100%)	

Executed script: \\SDS-SERVER2\SAS$\SDS3\Projects\STATISTICS DRUG DEV\NBMI4011 - CTI002\Analyser\Script\SAS\T_ms002.sas, on: 23MAY2016:14:00, by: maria.lidsell
Version of source data: nbmi_ma2.sas7bdat, last modified: 24FEB16:23:13, status: Final
*) Scores are 0 or 1, where 1 contributes to the Medical Score Sum.
**) Change can be interpreted as -1=better, 0=unchanged, 1=worse.

Table DE2.2.1 Categorical summary of change from baseline of Mercury intoxication medical score sum - Item 2. Tremor at work (Full analysis set)

		Result*			Change** from baseline (Screening visit)			
**Treatment**	**Visit**	**n**	**0**	**1**	**n**	**-1**	**0**	**1**
Total (n=36)	Screening	36 (100%)	31 (86.11%)	5 (13.89%)				
	Follow-up day 15	36 (100%)	35 (97.22%)	1 (2.78%)	36 (100%)	4 (11.11%)	32 (88.89%)	
	Follow-up day 45	36 (100%)	34 (94.44%)	2 (5.56%)	36 (100%)	3 (8.33%)	33 (91.67%)	
NBMI100mg (n=13)	Screening	13 (100%)	11 (84.62%)	2 (15.38%)				
	Follow-up day 15	13 (100%)	12 (92.31%)	1 (7.69%)	13 (100%)	1 (7.69%)	12 (92.31%)	
	Follow-up day 45	13 (100%)	12 (92.31%)	1 (7.69%)	13 (100%)	1 (7.69%)	12 (92.31%)	
NBMI300mg (n=11)	Screening	11 (100%)	8 (72.73%)	3 (27.27%)				
	Follow-up day 15	11 (100%)	11 (100%)		11 (100%)	3 (27.27%)	8 (72.73%)	
	Follow-up day 45	11 (100%)	10 (90.91%)	1 (9.09%)	11 (100%)	2 (18.18%)	9 (81.82%)	
Placebo (n=12)	Screening	12 (100%)	12 (100%)					
	Follow-up day 15	12 (100%)	12 (100%)		12 (100%)		12 (100%)	
	Follow-up day 45	12 (100%)	12 (100%)		12 (100%)		12 (100%)	

Executed script: \\SDS-SERVER2\SAS$\SDS3\Projects\STATISTICS DRUG DEV\NBMI4011 - CTI002\Analyser\Script\SAS\T_ms002.sas, on: 23MAY2016:14:00, by: maria.lidsell
Version of source data: nbmi_ma2.sas7bdat, last modified: 24FEB16:23:13, status: Final
*) Scores are 0 or 1, where 1 contributes to the Medical Score Sum.
**) Change can be interpreted as -1=better, 0=unchanged, 1=worse.

Table DE2.3.1 Categorical summary of change from baseline of Mercury intoxication medical score sum - Item 3. Sleeping problems at night (Full analysis set)

		Result*			Change** from baseline (Screening visit)			
**Treatment**	**Visit**	**n**	**0**	**1**	**n**	**-1**	**0**	**1**
Total (n=36)	Screening	36 (100%)	3 (8.33%)	33 (91.67%)				
	Follow-up day 15	36 (100%)	25 (69.44%)	11 (30.56%)	36 (100%)	22 (61.11%)	14 (38.89%)	
	Follow-up day 45	36 (100%)	27 (75%)	9 (25%)	36 (100%)	24 (66.67%)	12 (33.33%)	
NBMI100mg (n=13)	Screening	13 (100%)		13 (100%)				
	Follow-up day 15	13 (100%)	10 (76.92%)	3 (23.08%)	13 (100%)	10 (76.92%)	3 (23.08%)	
	Follow-up day 45	13 (100%)	8 (61.54%)	5 (38.46%)	13 (100%)	8 (61.54%)	5 (38.46%)	
NBMI300mg (n=11)	Screening	11 (100%)	2 (18.18%)	9 (81.82%)				
	Follow-up day 15	11 (100%)	9 (81.82%)	2 (18.18%)	11 (100%)	7 (63.64%)	4 (36.36%)	
	Follow-up day 45	11 (100%)	9 (81.82%)	2 (18.18%)	11 (100%)	7 (63.64%)	4 (36.36%)	
Placebo (n=12)	Screening	12 (100%)	1 (8.33%)	11 (91.67%)				
	Follow-up day 15	12 (100%)	6 (50%)	6 (50%)	12 (100%)	5 (41.67%)	7 (58.33%)	
	Follow-up day 45	12 (100%)	10 (83.33%)	2 (16.67%)	12 (100%)	9 (75%)	3 (25%)	

Executed script: \\SDS-SERVER2\SAS$\SDS3\Projects\STATISTICS DRUG DEV\NBMI4011 - CTI002\Analyser\Script\SAS\T_ms002.sas, on: 23MAY2016:14:00, by: maria.lidsell
Version of source data: nbmi_ma2.sas7bdat, last modified: 24FEB16:23:13, status: Final
*) Scores are 0 or 1, where 1 contributes to the Medical Score Sum.
**) Change can be interpreted as -1=better, 0=unchanged, 1=worse.

Table DE2.4.1 Categorical summary of change from baseline of Mercury intoxication medical score sum - Item 4. Bluish discoloration of gingiva (Full analysis set)

		**Result***			**Change** from baseline (Screening visit)**			
**Treatment**	**Visit**	**n**	**0**	**1**	**n**	**-1**	**0**	**1**
Total (n=36)	Screening	36 (100%)	24 (66.67%)	12 (33.33%)				
	Follow-up day 15	36 (100%)	26 (72.22%)	10 (27.78%)	36 (100%)	2 (5.56%)	34 (94.44%)	
	Follow-up day 45	36 (100%)	24 (66.67%)	12 (33.33%)	36 (100%)		36 (100%)	
NBMI100mg (n=13)	Screening	13 (100%)	7 (53.85%)	6 (46.15%)				
	Follow-up day 15	13 (100%)	8 (61.54%)	5 (38.46%)	13 (100%)	1 (7.69%)	12 (92.31%)	
	Follow-up day 45	13 (100%)	7 (53.85%)	6 (46.15%)	13 (100%)		13 (100%)	
NBMI300mg (n=11)	Screening	11 (100%)	7 (63.64%)	4 (36.36%)				
	Follow-up day 15	11 (100%)	7 (63.64%)	4 (36.36%)	11 (100%)		11 (100%)	
	Follow-up day 45	11 (100%)	7 (63.64%)	4 (36.36%)	11 (100%)		11 (100%)	
Placebo (n=12)	Screening	12 (100%)	10 (83.33%)	2 (16.67%)				
	Follow-up day 15	12 (100%)	11 (91.67%)	1 (8.33%)	12 (100%)	1 (8.33%)	11 (91.67%)	
	Follow-up day 45	12 (100%)	10 (83.33%)	2 (16.67%)	12 (100%)		12 (100%)	

Executed script: \\SDS-SERVER2\SAS$\SDS3\Projects\STATISTICS DRUG DEV\NBMI4011 - CTI002\Analyser\Script\SAS\T_ms002.sas, on: 23MAY2016:14:00, by: maria.lidsell
Version of source data: nbmi_ma2.sas7bdat, last modified: 24FEB16:23:13, status: Final
*) Scores are 0 or 1, where 1 contributes to the Medical Score Sum.
**) Change can be interpreted as -1=better, 0=unchanged, 1=worse.

Table DE2.5.1 Categorical summary of change from baseline of Mercury intoxication medical score sum - Item 5. Ataxia of gait (Full analysis set)

		**Result***			**Change** from baseline (Screening visit)**			
**Treatment**	**Visit**	**n**	**0**	**1**	**n**	**-1**	**0**	**1**
Total (n=36)	Screening	36 (100%)	10 (27.78%)	26 (72.22%)				
	Follow-up day 15	36 (100%)	11 (30.56%)	25 (69.44%)	36 (100%)	4 (11.11%)	29 (80.56%)	3 (8.33%)
	Follow-up day 45	36 (100%)	14 (38.89%)	22 (61.11%)	36 (100%)	8 (22.22%)	24 (66.67%)	4 (11.11%)
NBMI100mg (n=13)	Screening	13 (100%)	4 (30.77%)	9 (69.23%)				
	Follow-up day 15	13 (100%)	2 (15.38%)	11 (84.62%)	13 (100%)		11 (84.62%)	2 (15.38%)
	Follow-up day 45	13 (100%)	4 (30.77%)	9 (69.23%)	13 (100%)	1 (7.69%)	11 (84.62%)	1 (7.69%)
NBMI300mg (n=11)	Screening	11 (100%)	3 (27.27%)	8 (72.73%)				
	Follow-up day 15	11 (100%)	4 (36.36%)	7 (63.64%)	11 (100%)	2 (18.18%)	8 (72.73%)	1 (9.09%)
	Follow-up day 45	11 (100%)	4 (36.36%)	7 (63.64%)	11 (100%)	3 (27.27%)	6 (54.55%)	2 (18.18%)
Placebo (n=12)	Screening	12 (100%)	3 (25%)	9 (75%)				
	Follow-up day 15	12 (100%)	5 (41.67%)	7 (58.33%)	12 (100%)	2 (16.67%)	10 (83.33%)	
	Follow-up day 45	12 (100%)	6 (50%)	6 (50%)	12 (100%)	4 (33.33%)	7 (58.33%)	1 (8.33%)

Executed script: \\SDS-SERVER2\SAS$\SDS3\Projects\STATISTICS DRUG DEV\NBMI4011 - CTI002\Analyser\Script\SAS\T_ms002.sas, on: 23MAY2016:14:00, by: maria.lidsell
Version of source data: nbmi_ma2.sas7bdat, last modified: 24FEB16:23:13, status: Final
*) Scores are 0 or 1, where 1 contributes to the Medical Score Sum.
**) Change can be interpreted as -1=better, 0=unchanged, 1=worse.

Table DE2.6.1 Categorical summary of change from baseline of Mercury intoxication medical score sum - Item 6. Dysdiadochokinesia (Full analysis set)

		Result*			Change** from baseline (Screening visit)			
**Treatment**	**Visit**	**n**	**0**	**1**	**n**	**-1**	**0**	**1**
Total (n=36)	Screening	36 (100%)	4 (11.11%)	32 (88.89%)				
	Follow-up day 15	36 (100%)	9 (25%)	27 (75%)	36 (100%)	7 (19.44%)	27 (75%)	2 (5.56%)
	Follow-up day 45	36 (100%)	8 (22.22%)	28 (77.78%)	36 (100%)	7 (19.44%)	26 (72.22%)	3 (8.33%)
NBMI100mg (n=13)	Screening	13 (100%)	1 (7.69%)	12 (92.31%)				
	Follow-up day 15	13 (100%)	2 (15.38%)	11 (84.62%)	13 (100%)	2 (15.38%)	10 (76.92%)	1 (7.69%)
	Follow-up day 45	13 (100%)	1 (7.69%)	12 (92.31%)	13 (100%)	1 (7.69%)	11 (84.62%)	1 (7.69%)
NBMI300mg (n=11)	Screening	11 (100%)	2 (18.18%)	9 (81.82%)				
	Follow-up day 15	11 (100%)	4 (36.36%)	7 (63.64%)	11 (100%)	3 (27.27%)	7 (63.64%)	1 (9.09%)
	Follow-up day 45	11 (100%)	3 (27.27%)	8 (72.73%)	11 (100%)	3 (27.27%)	6 (54.55%)	2 (18.18%)
Placebo (n=12)	Screening	12 (100%)	1 (8.33%)	11 (91.67%)				
	Follow-up day 15	12 (100%)	3 (25%)	9 (75%)	12 (100%)	2 (16.67%)	10 (83.33%)	
	Follow-up day 45	12 (100%)	4 (33.33%)	8 (66.67%)	12 (100%)	3 (25%)	9 (75%)	

Executed script: \\SDS-SERVER2\SAS$\SDS3\Projects\STATISTICS DRUG DEV\NBMI4011 - CTI002\Analyser\Script\SAS\T_ms002.sas, on: 23MAY2016:14:00, by: maria.lidsell
Version of source data: nbmi_ma2.sas7bdat, last modified: 24FEB16:23:13, status: Final
*) Scores are 0 or 1, where 1 contributes to the Medical Score Sum.
**) Change can be interpreted as -1=better, 0=unchanged, 1=worse.

Table DE2.7.1 Categorical summary of change from baseline of Mercury intoxication medical score sum - Item 7. Heel-to-chin test (Full analysis set)

		**Result***			**Change** from baseline (Screening visit)**			
**Treatment**	**Visit**	**n**	**0**	**1**	**n**	**-1**	**0**	**1**
Total (n=36)	Screening	36 (100%)	4 (11.11%)	32 (88.89%)				
	Follow-up day 15	36 (100%)	15 (41.67%)	21 (58.33%)	36 (100%)	11 (30.56%)	25 (69.44%)	
	Follow-up day 45	36 (100%)	19 (52.78%)	17 (47.22%)	36 (100%)	16 (44.44%)	19 (52.78%)	1 (2.78%)
NBMI100mg (n=13)	Screening	13 (100%)	1 (7.69%)	12 (92.31%)				
	Follow-up day 15	13 (100%)	3 (23.08%)	10 (76.92%)	13 (100%)	2 (15.38%)	11 (84.62%)	
	Follow-up day 45	13 (100%)	5 (38.46%)	8 (61.54%)	13 (100%)	5 (38.46%)	7 (53.85%)	1 (7.69%)
NBMI300mg (n=11)	Screening	11 (100%)	2 (18.18%)	9 (81.82%)				
	Follow-up day 15	11 (100%)	6 (54.55%)	5 (45.45%)	11 (100%)	4 (36.36%)	7 (63.64%)	
	Follow-up day 45	11 (100%)	8 (72.73%)	3 (27.27%)	11 (100%)	6 (54.55%)	5 (45.45%)	
Placebo (n=12)	Screening	12 (100%)	1 (8.33%)	11 (91.67%)				
	Follow-up day 15	12 (100%)	6 (50%)	6 (50%)	12 (100%)	5 (41.67%)	7 (58.33%)	
	Follow-up day 45	12 (100%)	6 (50%)	6 (50%)	12 (100%)	5 (41.67%)	7 (58.33%)	

Executed script: \\SDS-SERVER2\SAS$\SDS3\Projects\STATISTICS DRUG DEV\NBMI4011 - CTI002\Analyser\Script\SAS\T_ms002.sas, on: 23MAY2016:14:00, by: maria.lidsell
Version of source data: nbmi_ma2.sas7bdat, last modified: 24FEB16:23:13, status: Final
*) Scores are 0 or 1, where 1 contributes to the Medical Score Sum.
**) Change can be interpreted as -1=better, 0=unchanged, 1=worse.

Table DE2.8.1 Categorical summary of change from baseline of Mercury intoxication medical score sum - Item 8. Proteinuria (Full analysis set)

		**Result***			**Change** from baseline (Screening visit)**			
**Treatment**	**Visit**	**n**	**0**	**1**	**n**	**-1**	**0**	**1**
Total (n=36)	Screening	36 (100%)	35 (97.22%)	1 (2.78%)				
	Follow-up day 15	36 (100%)	36 (100%)		36 (100%)	1 (2.78%)	35 (97.22%)	
	Follow-up day 45	36 (100%)	36 (100%)		36 (100%)	1 (2.78%)	35 (97.22%)	
NBMI100mg (n=13)	Screening	13 (100%)	13 (100%)					
	Follow-up day 15	13 (100%)	13 (100%)		13 (100%)		13 (100%)	
	Follow-up day 45	13 (100%)	13 (100%)		13 (100%)		13 (100%)	
NBMI300mg (n=11)	Screening	11 (100%)	10 (90.91%)	1 (9.09%)				
	Follow-up day 15	11 (100%)	11 (100%)		11 (100%)	1 (9.09%)	10 (90.91%)	
	Follow-up day 45	11 (100%)	11 (100%)		11 (100%)	1 (9.09%)	10 (90.91%)	
Placebo (n=12)	Screening	12 (100%)	12 (100%)					
	Follow-up day 15	12 (100%)	12 (100%)		12 (100%)		12 (100%)	
	Follow-up day 45	12 (100%)	12 (100%)		12 (100%)		12 (100%)	

Executed script: \\SDS-SERVER2\SAS$\SDS3\Projects\STATISTICS DRUG DEV\NBMI4011 - CTI002\Analyser\Script\SAS\T_ms002.sas, on: 23MAY2016:14:00, by: maria.lidsell
Version of source data: nbmi_ma2.sas7bdat, last modified: 24FEB16:23:13, status: Final
*) Scores are 0 or 1, where 1 contributes to the Medical Score Sum.
**) Change can be interpreted as -1=better, 0=unchanged, 1=worse.

Table DE2.9.1 Categorical summary of change from baseline of Mercury intoxication medical score sum - Item 9. Match box test (Full analysis set)

		Result*			Change** from baseline (Screening visit)			
**Treatment**	**Visit**	**n**	**0**	**1**	**n**	**-1**	**0**	**1**
Total (n=36)	Screening	36 (100%)	3 (8.33%)	33 (91.67%)				
	Follow-up day 15	36 (100%)	22 (61.11%)	14 (38.89%)	36 (100%)	19 (52.78%)	17 (47.22%)	
	Follow-up day 45	36 (100%)	23 (63.89%)	13 (36.11%)	36 (100%)	21 (58.33%)	14 (38.89%)	1 (2.78%)
NBMI100mg (n=13)	Screening	13 (100%)	1 (7.69%)	12 (92.31%)				
	Follow-up day 15	13 (100%)	6 (46.15%)	7 (53.85%)	13 (100%)	5 (38.46%)	8 (61.54%)	
	Follow-up day 45	13 (100%)	9 (69.23%)	4 (30.77%)	13 (100%)	8 (61.54%)	5 (38.46%)	
NBMI300mg (n=11)	Screening	11 (100%)	1 (9.09%)	10 (90.91%)				
	Follow-up day 15	11 (100%)	7 (63.64%)	4 (36.36%)	11 (100%)	6 (54.55%)	5 (45.45%)	
	Follow-up day 45	11 (100%)	6 (54.55%)	5 (45.45%)	11 (100%)	5 (45.45%)	6 (54.55%)	
Placebo (n=12)	Screening	12 (100%)	1 (8.33%)	11 (91.67%)				
	Follow-up day 15	12 (100%)	9 (75%)	3 (25%)	12 (100%)	8 (66.67%)	4 (33.33%)	
	Follow-up day 45	12 (100%)	8 (66.67%)	4 (33.33%)	12 (100%)	8 (66.67%)	3 (25%)	1 (8.33%)

Executed script: \\SDS-SERVER2\SAS$\SDS3\Projects\STATISTICS DRUG DEV\NBMI4011 - CTI002\Analyser\Script\SAS\T_ms002.sas, on: 23MAY2016:14:00, by: maria.lidsell
Version of source data: nbmi_ma2.sas7bdat, last modified: 24FEB16:23:13, status: Final
*) Scores are 0 or 1, where 1 contributes to the Medical Score Sum.
**) Change can be interpreted as -1=better, 0=unchanged, 1=worse.

Table DE2.10.1 Categorical summary of change from baseline of Mercury intoxication medical score sum - Item 10. Pencil tapping test (Full analysis set)

		**Result***			**Change** from baseline (Screening visit)**			
**Treatment**	**Visit**	**n**	**0**	**1**	**n**	**-1**	**0**	**1**
Total (n=36)	Screening	36 (100%)	9 (25%)	27 (75%)				
	Follow-up day 15	36 (100%)	33 (91.67%)	3 (8.33%)	36 (100%)	24 (66.67%)	12 (33.33%)	
	Follow-up day 45	36 (100%)	35 (97.22%)	1 (2.78%)	36 (100%)	26 (72.22%)	10 (27.78%)	
NBMI100mg (n=13)	Screening	13 (100%)	2 (15.38%)	11 (84.62%)				
	Follow-up day 15	13 (100%)	12 (92.31%)	1 (7.69%)	13 (100%)	10 (76.92%)	3 (23.08%)	
	Follow-up day 45	13 (100%)	12 (92.31%)	1 (7.69%)	13 (100%)	10 (76.92%)	3 (23.08%)	
NBMI300mg (n=11)	Screening	11 (100%)	3 (27.27%)	8 (72.73%)				
	Follow-up day 15	11 (100%)	10 (90.91%)	1 (9.09%)	11 (100%)	7 (63.64%)	4 (36.36%)	
	Follow-up day 45	11 (100%)	11 (100%)		11 (100%)	8 (72.73%)	3 (27.27%)	
Placebo (n=12)	Screening	12 (100%)	4 (33.33%)	8 (66.67%)				
	Follow-up day 15	12 (100%)	11 (91.67%)	1 (8.33%)	12 (100%)	7 (58.33%)	5 (41.67%)	
	Follow-up day 45	12 (100%)	12 (100%)		12 (100%)	8 (66.67%)	4 (33.33%)	

Executed script: \\SDS-SERVER2\SAS$\SDS3\Projects\STATISTICS DRUG DEV\NBMI4011 - CTI002\Analyser\Script\SAS\T_ms002.sas, on: 23MAY2016:14:00, by: maria.lidsell
Version of source data: nbmi_ma2.sas7bdat, last modified: 24FEB16:23:13, status: Final
*) Scores are 0 or 1, where 1 contributes to the Medical Score Sum.
**) Change can be interpreted as -1=better, 0=unchanged, 1=worse.

Table DE3.1.1 Shift table of baseline vs. Post baseline of Mercury intoxication medical score sum - Item 1. Excessive salivation (Full analysis set)

			Post baseline score*		
**Visit**	**Treatment**	**Baseline score* (Screening visit)**	**0**	**1**	**Total**
Follow-up day 15	Total (n=36)	0	24 (100%)		24 (66.67%)
		1	6 (50%)	6 (50%)	12 (33.33%)
		Total	30 (83.33%)	6 (16.67%)	36 (100%)
	NBMI100mg (n=13)	0	8 (100%)		8 (61.54%)
		1	2 (40%)	3 (60%)	5 (38.46%)
		Total	10 (76.92%)	3 (23.08%)	13 (100%)
	NBMI300mg (n=11)	0	4 (100%)		4 (36.36%)
		1	4 (57.14%)	3 (42.86%)	7 (63.64%)
		Total	8 (72.73%)	3 (27.27%)	11 (100%)
	Placebo (n=12)	0	12 (100%)		12 (100%)
		Total	12 (100%)		12 (100%)
Follow-up day 45	Total (n=36)	0	24 (100%)		24 (66.67%)
		1	7 (58.33%)	5 (41.67%)	12 (33.33%)
		Total	31 (86.11%)	5 (13.89%)	36 (100%)
	NBMI100mg (n=13)	0	8 (100%)		8 (61.54%)
		1	3 (60%)	2 (40%)	5 (38.46%)
		Total	11 (84.62%)	2 (15.38%)	13 (100%)
	NBMI300mg (n=11)	0	4 (100%)		4 (36.36%)
		1	4 (57.14%)	3 (42.86%)	7 (63.64%)
		Total	8 (72.73%)	3 (27.27%)	11 (100%)
	Placebo (n=12)	0	12 (100%)		12 (100%)
		Total	12 (100%)		12 (100%)

Executed script: \\SDS-SERVER2\SAS$\SDS3\Projects\STATISTICS DRUG DEV\NBMI4011 - CTI002\Analyser\Script\SAS\T_ms003.sas, on: 23MAY2016:14:00, by: maria.lidsell
Version of source data: nbmi_ma2.sas7bdat, last modified: 24FEB16:23:13, status: Final
*) Scores are 0 or 1, where 1 contributes to the Medical Score Sum.

Table DE3.2.1 Shift table of baseline vs. Post baseline of Mercury intoxication medical score sum - Item 2. Tremor at work (Full analysis set)

			Post baseline score*		
**Visit**	**Treatment**	**Baseline score* (Screening visit)**	**0**	**1**	**Total**
Follow-up day 15	Total (n=36)	0	31 (100%)		31 (86.11%)
		1	4 (80%)	1 (20%)	5 (13.89%)
		Total	35 (97.22%)	1 (2.78%)	36 (100%)
	NBMI100mg (n=13)	0	11 (100%)		11 (84.62%)
		1	1 (50%)	1 (50%)	2 (15.38%)
		Total	12 (92.31%)	1 (7.69%)	13 (100%)
	NBMI300mg (n=11)	0	8 (100%)		8 (72.73%)
		1	3 (100%)		3 (27.27%)
		Total	11 (100%)		11 (100%)
	Placebo (n=12)	0	12 (100%)		12 (100%)
		Total	12 (100%)		12 (100%)
Follow-up day 45	Total (n=36)	0	31 (100%)		31 (86.11%)
		1	3 (60%)	2 (40%)	5 (13.89%)
		Total	34 (94.44%)	2 (5.56%)	36 (100%)
	NBMI100mg (n=13)	0	11 (100%)		11 (84.62%)
		1	1 (50%)	1 (50%)	2 (15.38%)
		Total	12 (92.31%)	1 (7.69%)	13 (100%)
	NBMI300mg (n=11)	0	8 (100%)		8 (72.73%)
		1	2 (66.67%)	1 (33.33%)	3 (27.27%)
		Total	10 (90.91%)	1 (9.09%)	11 (100%)
	Placebo (n=12)	0	12 (100%)		12 (100%)
		Total	12 (100%)		12 (100%)

Executed script: \\SDS-SERVER2\SAS$\SDS3\Projects\STATISTICS DRUG DEV\NBMI4011 - CTI002\Analyser\Script\SAS\T_ms003.sas, on: 23MAY2016:14:00, by: maria.lidsell
Version of source data: nbmi_ma2.sas7bdat, last modified: 24FEB16:23:13, status: Final
*) Scores are 0 or 1, where 1 contributes to the Medical Score Sum.

Table DE3.3.1 Shift table of baseline vs. Post baseline of Mercury intoxication medical score sum - Item 3. Sleeping problems at night (Full analysis set)

			**Post baseline score***		
**Visit**	**Treatment**	**Baseline score*** **(Screening visit)**	**0**	**1**	**Total**
Follow-up day 15	Total (n=36)	0	3 (100%)		3 (8.33%)
		1	22 (66.67%)	11 (33.33%)	33 (91.67%)
		Total	25 (69.44%)	11 (30.56%)	36 (100%)
	NBMI100mg (n=13)	1	10 (76.92%)	3 (23.08%)	13 (100%)
		Total	10 (76.92%)	3 (23.08%)	13 (100%)
	NBMI300mg (n=11)	0	2 (100%)		2 (18.18%)
		1	7 (77.78%)	2 (22.22%)	9 (81.82%)
		Total	9 (81.82%)	2 (18.18%)	11 (100%)
	Placebo (n=12)	0	1 (100%)		1 (8.33%)
		1	5 (45.45%)	6 (54.55%)	11 (91.67%)
		Total	6 (50%)	6 (50%)	12 (100%)
Follow-up day 45	Total (n=36)	0	3 (100%)		3 (8.33%)
		1	24 (72.73%)	9 (27.27%)	33 (91.67%)
		Total	27 (75%)	9 (25%)	36 (100%)
	NBMI100mg (n=13)	1	8 (61.54%)	5 (38.46%)	13 (100%)
		Total	8 (61.54%)	5 (38.46%)	13 (100%)
	NBMI300mg (n=11)	0	2 (100%)		2 (18.18%)
		1	7 (77.78%)	2 (22.22%)	9 (81.82%)
		Total	9 (81.82%)	2 (18.18%)	11 (100%)
	Placebo (n=12)	0	1 (100%)		1 (8.33%)
		1	9 (81.82%)	2 (18.18%)	11 (91.67%)
		Total	10 (83.33%)	2 (16.67%)	12 (100%)

Executed script: \\SDS-SERVER2\SAS$\SDS3\Projects\STATISTICS DRUG DEV\NBMI4011 - CTI002\Analyser\Script\SAS\T_ms003.sas, on: 23MAY2016:14:00, by: maria.lidsell
Version of source data: nbmi_ma2.sas7bdat, last modified: 24FEB16:23:13, status: Final
*) Scores are 0 or 1, where 1 contributes to the Medical Score Sum.

Table DE3.4.1 Shift table of baseline vs. Post baseline of Mercury intoxication medical score sum - Item 4. Bluish discoloration of gingiva (Full analysis set)

			Post baseline score*		
**Visit**	**Treatment**	**Baseline score* (Screening visit)**	**0**	**1**	**Total**
Follow-up day 15	Total (n=36)	0	24 (100%)		24 (66.67%)
		1	2 (16.67%)	10 (83.33%)	12 (33.33%)
		Total	26 (72.22%)	10 (27.78%)	36 (100%)
	NBMI100mg (n=13)	0	7 (100%)		7 (53.85%)
		1	1 (16.67%)	5 (83.33%)	6 (46.15%)
		Total	8 (61.54%)	5 (38.46%)	13 (100%)
	NBMI300mg (n=11)	0	7 (100%)		7 (63.64%)
		1		4 (100%)	4 (36.36%)
		Total	7 (63.64%)	4 (36.36%)	11 (100%)
	Placebo (n=12)	0	10 (100%)		10 (83.33%)
		1	1 (50%)	1 (50%)	2 (16.67%)
		Total	11 (91.67%)	1 (8.33%)	12 (100%)
Follow-up day 45	Total (n=36)	0	24 (100%)		24 (66.67%)
		1		12 (100%)	12 (33.33%)
		Total	24 (66.67%)	12 (33.33%)	36 (100%)
	NBMI100mg (n=13)	0	7 (100%)		7 (53.85%)
		1		6 (100%)	6 (46.15%)
		Total	7 (53.85%)	6 (46.15%)	13 (100%)
	NBMI300mg (n=11)	0	7 (100%)		7 (63.64%)
		1		4 (100%)	4 (36.36%)
		Total	7 (63.64%)	4 (36.36%)	11 (100%)
	Placebo (n=12)	0	10 (100%)		10 (83.33%)
		1		2 (100%)	2 (16.67%)
		Total	10 (83.33%)	2 (16.67%)	12 (100%)

Executed script: \\SDS-SERVER2\SAS$\SDS3\Projects\STATISTICS DRUG DEV\NBMI4011 - CTI002\Analyser\Script\SAS\T_ms003.sas, on: 23MAY2016:14:00, by: maria.lidsell
Version of source data: nbmi_ma2.sas7bdat, last modified: 24FEB16:23:13, status: Final
*) Scores are 0 or 1, where 1 contributes to the Medical Score Sum.

Table DE3.5.1 Shift table of baseline vs. Post baseline of Mercury intoxication medical score sum - Item 5. Ataxia of gait (Full analysis set)

			Post baseline score*		
**Visit**	**Treatment**	**Baseline score* (Screening visit)**	**0**	**1**	**Total**
Follow-up day 15	Total (n=36)	0	7 (70%)	3 (30%)	10 (27.78%)
		1	4 (15.38%)	22 (84.62%)	26 (72.22%)
		Total	11 (30.56%)	25 (69.44%)	36 (100%)
	NBMI100mg (n=13)	0	2 (50%)	2 (50%)	4 (30.77%)
		1		9 (100%)	9 (69.23%)
		Total	2 (15.38%)	11 (84.62%)	13 (100%)
	NBMI300mg (n=11)	0	2 (66.67%)	1 (33.33%)	3 (27.27%)
		1	2 (25%)	6 (75%)	8 (72.73%)
		Total	4 (36.36%)	7 (63.64%)	11 (100%)
	Placebo (n=12)	0	3 (100%)		3 (25%)
		1	2 (22.22%)	7 (77.78%)	9 (75%)
		Total	5 (41.67%)	7 (58.33%)	12 (100%)
Follow-up day 45	Total (n=36)	0	6 (60%)	4 (40%)	10 (27.78%)
		1	8 (30.77%)	18 (69.23%)	26 (72.22%)
		Total	14 (38.89%)	22 (61.11%)	36 (100%)
	NBMI100mg (n=13)	0	3 (75%)	1 (25%)	4 (30.77%)
		1	1 (11.11%)	8 (88.89%)	9 (69.23%)
		Total	4 (30.77%)	9 (69.23%)	13 (100%)
	NBMI300mg (n=11)	0	1 (33.33%)	2 (66.67%)	3 (27.27%)
		1	3 (37.5%)	5 (62.5%)	8 (72.73%)
		Total	4 (36.36%)	7 (63.64%)	11 (100%)
	Placebo (n=12)	0	2 (66.67%)	1 (33.33%)	3 (25%)
		1	4 (44.44%)	5 (55.56%)	9 (75%)
		Total	6 (50%)	6 (50%)	12 (100%)

Executed script: \\SDS-SERVER2\SAS$\SDS3\Projects\STATISTICS DRUG DEV\NBMI4011 - CTI002\Analyser\Script\SAS\T_ms003.sas, on: 23MAY2016:14:00, by: maria.lidsell
Version of source data: nbmi_ma2.sas7bdat, last modified: 24FEB16:23:13, status: Final
*) Scores are 0 or 1, where 1 contributes to the Medical Score Sum.

Table DE3.6.1 Shift table of baseline vs. Post baseline of Mercury intoxication medical score sum - Item 6. Dysdiadochokinesia (Full analysis set)

			Post baseline score*		
**Visit**	**Treatment**	**Baseline score* (Screening visit)**	**0**	**1**	**Total**
Follow-up day 15	Total (n=36)	0	2 (50%)	2 (50%)	4 (11.11%)
		1	7 (21.88%)	25 (78.13%)	32 (88.89%)
		Total	9 (25%)	27 (75%)	36 (100%)
	NBMI100mg (n=13)	0		1 (100%)	1 (7.69%)
		1	2 (16.67%)	10 (83.33%)	12 (92.31%)
		Total	2 (15.38%)	11 (84.62%)	13 (100%)
	NBMI300mg (n=11)	0	1 (50%)	1 (50%)	2 (18.18%)
		1	3 (33.33%)	6 (66.67%)	9 (81.82%)
		Total	4 (36.36%)	7 (63.64%)	11 (100%)
	Placebo (n=12)	0	1 (100%)		1 (8.33%)
		1	2 (18.18%)	9 (81.82%)	11 (91.67%)
		Total	3 (25%)	9 (75%)	12 (100%)
Follow-up day 45	Total (n=36)	0	1 (25%)	3 (75%)	4 (11.11%)
		1	7 (21.88%)	25 (78.13%)	32 (88.89%)
		Total	8 (22.22%)	28 (77.78%)	36 (100%)
	NBMI100mg (n=13)	0		1 (100%)	1 (7.69%)
		1	1 (8.33%)	11 (91.67%)	12 (92.31%)
		Total	1 (7.69%)	12 (92.31%)	13 (100%)
	NBMI300mg (n=11)	0		2 (100%)	2 (18.18%)
		1	3 (33.33%)	6 (66.67%)	9 (81.82%)
		Total	3 (27.27%)	8 (72.73%)	11 (100%)
	Placebo (n=12)	0	1 (100%)		1 (8.33%)
		1	3 (27.27%)	8 (72.73%)	11 (91.67%)
		Total	4 (33.33%)	8 (66.67%)	12 (100%)

Executed script: \\SDS-SERVER2\SAS$\SDS3\Projects\STATISTICS DRUG DEV\NBMI4011 - CTI002\Analyser\Script\SAS\T_ms003.sas, on: 23MAY2016:14:00, by: maria.lidsell
Version of source data: nbmi_ma2.sas7bdat, last modified: 24FEB16:23:13, status: Final
*) Scores are 0 or 1, where 1 contributes to the Medical Score Sum.

Table DE3.7.1 Shift table of baseline vs. Post baseline of Mercury intoxication medical score sum - Item 7. Heel-to-chin test (Full analysis set)

			Post baseline score*		
**Visit**	**Treatment**	**Baseline score* (Screening visit)**	**0**	**1**	**Total**
Follow-up day 15	Total (n=36)	0	4 (100%)		4 (11.11%)
		1	11 (34.38%)	21 (65.63%)	32 (88.89%)
		Total	15 (41.67%)	21 (58.33%)	36 (100%)
	NBMI100mg (n=13)	0	1 (100%)		1 (7.69%)
		1	2 (16.67%)	10 (83.33%)	12 (92.31%)
		Total	3 (23.08%)	10 (76.92%)	13 (100%)
	NBMI300mg (n=11)	0	2 (100%)		2 (18.18%)
		1	4 (44.44%)	5 (55.56%)	9 (81.82%)
		Total	6 (54.55%)	5 (45.45%)	11 (100%)
	Placebo (n=12)	0	1 (100%)		1 (8.33%)
		1	5 (45.45%)	6 (54.55%)	11 (91.67%)
		Total	6 (50%)	6 (50%)	12 (100%)
Follow-up day 45	Total (n=36)	0	3 (75%)	1 (25%)	4 (11.11%)
		1	16 (50%)	16 (50%)	32 (88.89%)
		Total	19 (52.78%)	17 (47.22%)	36 (100%)
	NBMI100mg (n=13)	0		1 (100%)	1 (7.69%)
		1	5 (41.67%)	7 (58.33%)	12 (92.31%)
		Total	5 (38.46%)	8 (61.54%)	13 (100%)
	NBMI300mg (n=11)	0	2 (100%)		2 (18.18%)
		1	6 (66.67%)	3 (33.33%)	9 (81.82%)
		Total	8 (72.73%)	3 (27.27%)	11 (100%)
	Placebo (n=12)	0	1 (100%)		1 (8.33%)
		1	5 (45.45%)	6 (54.55%)	11 (91.67%)
		Total	6 (50%)	6 (50%)	12 (100%)

Executed script: \\SDS-SERVER2\SAS$\SDS3\Projects\STATISTICS DRUG DEV\NBMI4011 - CTI002\Analyser\Script\SAS\T_ms003.sas, on: 23MAY2016:14:00, by: maria.lidsell
Version of source data: nbmi_ma2.sas7bdat, last modified: 24FEB16:23:13, status: Final
*) Scores are 0 or 1, where 1 contributes to the Medical Score Sum.

Table DE3.8.1 Shift table of baseline vs. Post baseline of Mercury intoxication medical score sum - Item 8. Proteinuria (Full analysis set)

			Post baseline score*		
**Visit**	**Treatment**	**Baseline score* (Screening visit)**	**0**	**1**	**Total**
Follow-up day 15	Total (n=36)	0	35 (100%)		35 (97.22%)
		1	1 (100%)		1 (2.78%)
		Total	36 (100%)		36 (100%)
	NBMI100mg (n=13)	0	13 (100%)		13 (100%)
		Total	13 (100%)		13 (100%)
	NBMI300mg (n=11)	0	10 (100%)		10 (90.91%)
		1	1 (100%)		1 (9.09%)
		Total	11 (100%)		11 (100%)
	Placebo (n=12)	0	12 (100%)		12 (100%)
		Total	12 (100%)		12 (100%)
Follow-up day 45	Total (n=36)	0	35 (100%)		35 (97.22%)
		1	1 (100%)		1 (2.78%)
		Total	36 (100%)		36 (100%)
	NBMI100mg (n=13)	0	13 (100%)		13 (100%)
		Total	13 (100%)		13 (100%)
	NBMI300mg (n=11)	0	10 (100%)		10 (90.91%)
		1	1 (100%)		1 (9.09%)
		Total	11 (100%)		11 (100%)
	Placebo (n=12)	0	12 (100%)		12 (100%)
		Total	12 (100%)		12 (100%)

Executed script: \\SDS-SERVER2\SAS$\SDS3\Projects\STATISTICS DRUG DEV\NBMI4011 - CTI002\Analyser\Script\SAS\T_ms003.sas, on: 23MAY2016:14:00, by: maria.lidsell
Version of source data: nbmi_ma2.sas7bdat, last modified: 24FEB16:23:13, status: Final
*) Scores are 0 or 1, where 1 contributes to the Medical Score Sum.

Table DE3.9.1 Shift table of baseline vs. Post baseline of Mercury intoxication medical score sum - Item 9. Match box test (Full analysis set)

			Post baseline score*		
**Visit**	**Treatment**	**Baseline score* (Screening visit)**	**0**	**1**	**Total**
Follow-up day 15	Total (n=36)	0	3 (100%)		3 (8.33%)
		1	19 (57.58%)	14 (42.42%)	33 (91.67%)
		Total	22 (61.11%)	14 (38.89%)	36 (100%)
	NBMI100mg (n=13)	0	1 (100%)		1 (7.69%)
		1	5 (41.67%)	7 (58.33%)	12 (92.31%)
		Total	6 (46.15%)	7 (53.85%)	13 (100%)
	NBMI300mg (n=11)	0	1 (100%)		1 (9.09%)
		1	6 (60%)	4 (40%)	10 (90.91%)
		Total	7 (63.64%)	4 (36.36%)	11 (100%)
	Placebo (n=12)	0	1 (100%)		1 (8.33%)
		1	8 (72.73%)	3 (27.27%)	11 (91.67%)
		Total	9 (75%)	3 (25%)	12 (100%)
Follow-up day 45	Total (n=36)	0	2 (66.67%)	1 (33.33%)	3 (8.33%)
		1	21 (63.64%)	12 (36.36%)	33 (91.67%)
		Total	23 (63.89%)	13 (36.11%)	36 (100%)
	NBMI100mg (n=13)	0	1 (100%)		1 (7.69%)
		1	8 (66.67%)	4 (33.33%)	12 (92.31%)
		Total	9 (69.23%)	4 (30.77%)	13 (100%)
	NBMI300mg (n=11)	0	1 (100%)		1 (9.09%)
		1	5 (50%)	5 (50%)	10 (90.91%)
		Total	6 (54.55%)	5 (45.45%)	11 (100%)
	Placebo (n=12)	0		1 (100%)	1 (8.33%)
		1	8 (72.73%)	3 (27.27%)	11 (91.67%)
		Total	8 (66.67%)	4 (33.33%)	12 (100%)

Executed script: \\SDS-SERVER2\SAS$\SDS3\Projects\STATISTICS DRUG DEV\NBMI4011 - CTI002\Analyser\Script\SAS\T_ms003.sas, on: 23MAY2016:14:00, by: maria.lidsell
Version of source data: nbmi_ma2.sas7bdat, last modified: 24FEB16:23:13, status: Final
*) Scores are 0 or 1, where 1 contributes to the Medical Score Sum.

Table DE3.10.1 Shift table of baseline vs. Post baseline of Mercury intoxication medical score sum - Item 10. Pencil tapping test (Full analysis set)

			**Post baseline score***		
**Visit**	**Treatment**	**Baseline score* (Screening visit)**	**0**	**1**	**Total**
Follow-up day 15	Total (n=36)	0	9 (100%)		9 (25%)
		1	24 (88.89%)	3 (11.11%)	27 (75%)
		Total	33 (91.67%)	3 (8.33%)	36 (100%)
	NBMI100mg (n=13)	0	2 (100%)		2 (15.38%)
		1	10 (90.91%)	1 (9.09%)	11 (84.62%)
		Total	12 (92.31%)	1 (7.69%)	13 (100%)
	NBMI300mg (n=11)	0	3 (100%)		3 (27.27%)
		1	7 (87.5%)	1 (12.5%)	8 (72.73%)
		Total	10 (90.91%)	1 (9.09%)	11 (100%)
	Placebo (n=12)	0	4 (100%)		4 (33.33%)
		1	7 (87.5%)	1 (12.5%)	8 (66.67%)
		Total	11 (91.67%)	1 (8.33%)	12 (100%)
Follow-up day 45	Total (n=36)	0	9 (100%)		9 (25%)
		1	26 (96.3%)	1 (3.7%)	27 (75%)
		Total	35 (97.22%)	1 (2.78%)	36 (100%)
	NBMI100mg (n=13)	0	2 (100%)		2 (15.38%)
		1	10 (90.91%)	1 (9.09%)	11 (84.62%)
		Total	12 (92.31%)	1 (7.69%)	13 (100%)
	NBMI300mg (n=11)	0	3 (100%)		3 (27.27%)
		1	8 (100%)		8 (72.73%)
		Total	11 (100%)		11 (100%)
	Placebo (n=12)	0	4 (100%)		4 (33.33%)
		1	8 (100%)		8 (66.67%)
		Total	12 (100%)		12 (100%)

Executed script: \\SDS-SERVER2\SAS$\SDS3\Projects\STATISTICS DRUG DEV\NBMI4011 - CTI002\Analyser\Script\SAS\T_ms003.sas, on: 23MAY2016:14:00, by: maria.lidsell
Version of source data: nbmi_ma2.sas7bdat, last modified: 24FEB16:23:13, status: Final
*) Scores are 0 or 1, where 1 contributes to the Medical Score Sum.

Table DE4.1.1 Categorical summary of change from usual of Chronic mercury intoxication symptoms - Item 1. Metallic taste (Full analysis set)

		**Result***						**Change from usual****				
**Treatment**	**Visit**	**n**	**NA*****	**much better than usual**	**better than usual**	**same as usual**	**worse than usual**	**n**	**nmiss**	**-1**	**0**	**1**
Total (n=36)	Follow-up day 15	17 (47.22%)	11 (30.56%)	1 (2.78%)	1 (2.78%)	4 (11.11%)		6 (16.67%)	11 (30.56%)	2 (5.56%)	4 (11.11%)	
	Follow-up day 45	17 (47.22%)	13 (36.11%)	2 (5.56%)	2 (5.56%)			4 (11.11%)	13 (36.11%)	4 (11.11%)		
NBMI100mg (n=13)	Follow-up day 15	6 (46.15%)	3 (23.08%)			3 (23.08%)		3 (23.08%)	3 (23.08%)		3 (23.08%)	
	Follow-up day 45	6 (46.15%)	5 (38.46%)		1 (7.69%)			1 (7.69%)	5 (38.46%)	1 (7.69%)		
NBMI300mg (n=11)	Follow-up day 15	5 (45.45%)	4 (36.36%)			1 (9.09%)		1 (9.09%)	4 (36.36%)		1 (9.09%)	
	Follow-up day 45	5 (45.45%)	4 (36.36%)		1 (9.09%)			1 (9.09%)	4 (36.36%)	1 (9.09%)		
Placebo (n=12)	Follow-up day 15	6 (50%)	4 (33.33%)	1 (8.33%)	1 (8.33%)			2 (16.67%)	4 (33.33%)	2 (16.67%)		
	Follow-up day 45	6 (50%)	4 (33.33%)	2 (16.67%)				2 (16.67%)	4 (33.33%)	2 (16.67%)		

Executed script: \\SDS-SERVER2\SAS$\SDS3\Projects\STATISTICS DRUG DEV\NBMI4011 - CTI002\Analyser\Script\SAS\T_mi002.sas, on: 23MAY2016:14:00, by: maria.lidsell
Version of source data: nbmi_ch1.sas7bdat, last modified: 24FEB16:23:13, status: Final
*) Scores are counted according to: much better than usual=0, better than usual=0, same as usual=1, worse than usual=2, much worse than usual=3.
**) Change for chronic mercury intoxication symptoms is defined as difference from 1=same as usual.
***) Missing results registered in database as 'NA'

Table DE4.2.1 Categorical summary of change from usual of Chronic mercury intoxication symptoms - Item 2. Irritability (Full analysis set)

		**Result***						**Change from usual****				
**Treatment**	**Visit**	**n**	**NA*****	**much better than usual**	**better than usual**	**same as usual**	**worse than usual**	**n**	**nmiss**	**-1**	**0**	**1**
Total (n=36)	Follow-up day 15	17 (47.22%)	2 (5.56%)	3 (8.33%)	11 (30.56%)	1 (2.78%)		15 (41.67%)	2 (5.56%)	14 (38.89%)	1 (2.78%)	
	Follow-up day 45	17 (47.22%)	2 (5.56%)	4 (11.11%)	9 (25%)	1 (2.78%)	1 (2.78%)	15 (41.67%)	2 (5.56%)	13 (36.11%)	1 (2.78%)	1 (2.78%)
NBMI100mg (n=13)	Follow-up day 15	6 (46.15%)	1 (7.69%)		4 (30.77%)	1 (7.69%)		5 (38.46%)	1 (7.69%)	4 (30.77%)	1 (7.69%)	
	Follow-up day 45	6 (46.15%)	1 (7.69%)	1 (7.69%)	3 (23.08%)		1 (7.69%)	5 (38.46%)	1 (7.69%)	4 (30.77%)		1 (7.69%)
NBMI300mg (n=11)	Follow-up day 15	5 (45.45%)	1 (9.09%)	2 (18.18%)	2 (18.18%)			4 (36.36%)	1 (9.09%)	4 (36.36%)		
	Follow-up day 45	5 (45.45%)	1 (9.09%)	2 (18.18%)	2 (18.18%)			4 (36.36%)	1 (9.09%)	4 (36.36%)		
Placebo (n=12)	Follow-up day 15	6 (50%)		1 (8.33%)	5 (41.67%)			6 (50%)		6 (50%)		
	Follow-up day 45	6 (50%)		1 (8.33%)	4 (33.33%)	1 (8.33%)		6 (50%)		5 (41.67%)	1 (8.33%)	

Executed script: \\SDS-SERVER2\SAS$\SDS3\Projects\STATISTICS DRUG DEV\NBMI4011 - CTI002\Analyser\Script\SAS\T_mi002.sas, on: 23MAY2016:14:00, by: maria.lidsell
Version of source data: nbmi_ch1.sas7bdat, last modified: 24FEB16:23:13, status: Final
*) Scores are counted according to: much better than usual=0, better than usual=0, same as usual=1, worse than usual=2, much worse than usual=3.
**) Change for chronic mercury intoxication symptoms is defined as difference from 1=same as usual.
***) Missing results registered in database as 'NA'

Table DE4.3.1 Categorical summary of change from usual of Chronic mercury intoxication symptoms - Item 3. Social nervousness and/or withdrawal (Full analysis set)

		**Result***						**Change from usual****				
**Treatment**	**Visit**	**n**	**NA*****	**much better than usual**	**better than usual**	**same as usual**	**worse than usual**	**n**	**nmiss**	**-1**	**0**	**1**
Total (n=36)	Follow-up day 15	17 (47.22%)	10 (27.78%)		4 (11.11%)	3 (8.33%)		7 (19.44%)	10 (27.78%)	4 (11.11%)	3 (8.33%)	
	Follow-up day 45	17 (47.22%)	10 (27.78%)		4 (11.11%)	3 (8.33%)		7 (19.44%)	10 (27.78%)	4 (11.11%)	3 (8.33%)	
NBMI100mg (n=13)	Follow-up day 15	6 (46.15%)	4 (30.77%)		1 (7.69%)	1 (7.69%)		2 (15.38%)	4 (30.77%)	1 (7.69%)	1 (7.69%)	
	Follow-up day 45	6 (46.15%)	4 (30.77%)		2 (15.38%)			2 (15.38%)	4 (30.77%)	2 (15.38%)		
NBMI300mg (n=11)	Follow-up day 15	5 (45.45%)	3 (27.27%)		2 (18.18%)			2 (18.18%)	3 (27.27%)	2 (18.18%)		
	Follow-up day 45	5 (45.45%)	3 (27.27%)		2 (18.18%)			2 (18.18%)	3 (27.27%)	2 (18.18%)		
Placebo (n=12)	Follow-up day 15	6 (50%)	3 (25%)		1 (8.33%)	2 (16.67%)		3 (25%)	3 (25%)	1 (8.33%)	2 (16.67%)	
	Follow-up day 45	6 (50%)	3 (25%)			3 (25%)		3 (25%)	3 (25%)		3 (25%)	

Executed script: \\SDS-SERVER2\SAS$\SDS3\Projects\STATISTICS DRUG DEV\NBMI4011 - CTI002\Analyser\Script\SAS\T_mi002.sas, on: 23MAY2016:14:00, by: maria.lidsell
Version of source data: nbmi_ch1.sas7bdat, last modified: 24FEB16:23:13, status: Final
*) Scores are counted according to: much better than usual=0, better than usual=0, same as usual=1, worse than usual=2, much worse than usual=3.
**) Change for chronic mercury intoxication symptoms is defined as difference from 1=same as usual.
***) Missing results registered in database as 'NA'

Table DE4.4.1 Categorical summary of change from usual of Chronic mercury intoxication symptoms - Item 4. Memory loss (Full analysis set)

		**Result***						**Change from usual****				
**Treatment**	**Visit**	**n**	**NA*****	**much better than usual**	**better than usual**	**same as usual**	**worse than usual**	**n**	**nmiss**	**-1**	**0**	**1**
Total (n=36)	Follow-up day 15	17 (47.22%)	7 (19.44%)		7 (19.44%)	3 (8.33%)		10 (27.78%)	7 (19.44%)	7 (19.44%)	3 (8.33%)	
	Follow-up day 45	17 (47.22%)	6 (16.67%)		6 (16.67%)	5 (13.89%)		11 (30.56%)	6 (16.67%)	6 (16.67%)	5 (13.89%)	
NBMI100mg (n=13)	Follow-up day 15	6 (46.15%)	3 (23.08%)		2 (15.38%)	1 (7.69%)		3 (23.08%)	3 (23.08%)	2 (15.38%)	1 (7.69%)	
	Follow-up day 45	6 (46.15%)	2 (15.38%)			4 (30.77%)		4 (30.77%)	2 (15.38%)		4 (30.77%)	
NBMI300mg (n=11)	Follow-up day 15	5 (45.45%)	2 (18.18%)		2 (18.18%)	1 (9.09%)		3 (27.27%)	2 (18.18%)	2 (18.18%)	1 (9.09%)	
	Follow-up day 45	5 (45.45%)	2 (18.18%)		2 (18.18%)	1 (9.09%)		3 (27.27%)	2 (18.18%)	2 (18.18%)	1 (9.09%)	
Placebo (n=12)	Follow-up day 15	6 (50%)	2 (16.67%)		3 (25%)	1 (8.33%)		4 (33.33%)	2 (16.67%)	3 (25%)	1 (8.33%)	
	Follow-up day 45	6 (50%)	2 (16.67%)		4 (33.33%)			4 (33.33%)	2 (16.67%)	4 (33.33%)		

Executed script: \\SDS-SERVER2\SAS$\SDS3\Projects\STATISTICS DRUG DEV\NBMI4011 - CTI002\Analyser\Script\SAS\T_mi002.sas, on: 23MAY2016:14:00, by: maria.lidsell
Version of source data: nbmi_ch1.sas7bdat, last modified: 24FEB16:23:13, status: Final
*) Scores are counted according to: much better than usual=0, better than usual=0, same as usual=1, worse than usual=2, much worse than usual=3.
**) Change for chronic mercury intoxication symptoms is defined as difference from 1=same as usual.
***) Missing results registered in database as 'NA'

Table DE4.5.1 Categorical summary of change from usual of Chronic mercury intoxication symptoms - Item 5. Mental fatigue (Full analysis set)

		Result*						Change from usual**				
**Treatment**	**Visit**	**n**	**NA*** **	**much better than usual**	**better than usual**	**same as usual**	**worse than usual**	**n**	**nmiss**	**-1**	**0**	**1**
Total (n=36)	Follow-up day 15	17 (47.22%)	6 (16.67%)		8 (22.22%)	3 (8.33%)		11 (30.56%)	6 (16.67%)	8 (22.22%)	3 (8.33%)	
	Follow-up day 45	17 (47.22%)	7 (19.44%)	3 (8.33%)	4 (11.11%)	3 (8.33%)		10 (27.78%)	7 (19.44%)	7 (19.44%)	3 (8.33%)	
NBMI100mg (n=13)	Follow-up day 15	6 (46.15%)	4 (30.77%)		1 (7.69%)	1 (7.69%)		2 (15.38%)	4 (30.77%)	1 (7.69%)	1 (7.69%)	
	Follow-up day 45	6 (46.15%)	5 (38.46%)		1 (7.69%)			1 (7.69%)	5 (38.46%)	1 (7.69%)		
NBMI300mg (n=11)	Follow-up day 15	5 (45.45%)	1 (9.09%)		3 (27.27%)	1 (9.09%)		4 (36.36%)	1 (9.09%)	3 (27.27%)	1 (9.09%)	
	Follow-up day 45	5 (45.45%)	1 (9.09%)	2 (18.18%)	2 (18.18%)			4 (36.36%)	1 (9.09%)	4 (36.36%)		
Placebo (n=12)	Follow-up day 15	6 (50%)	1 (8.33%)		4 (33.33%)	1 (8.33%)		5 (41.67%)	1 (8.33%)	4 (33.33%)	1 (8.33%)	
	Follow-up day 45	6 (50%)	1 (8.33%)	1 (8.33%)	1 (8.33%)	3 (25%)		5 (41.67%)	1 (8.33%)	2 (16.67%)	3 (25%)	

Executed script: \\SDS-SERVER2\SAS$\SDS3\Projects\STATISTICS DRUG DEV\NBMI4011 - CTI002\Analyser\Script\SAS\T_mi002.sas, on: 23MAY2016:14:00, by: maria.lidsell
Version of source data: nbmi_ch1.sas7bdat, last modified: 24FEB16:23:13, status: Final
*) Scores are counted according to: much better than usual=0, better than usual=0, same as usual=1, worse than usual=2, much worse than usual=3.
**) Change for chronic mercury intoxication symptoms is defined as difference from 1=same as usual.
***) Missing results registered in database as 'NA'

Table DE4.6.1 Categorical summary of change from usual of Chronic mercury intoxication symptoms - Item 6. Physical fatigue (Full analysis set)

		Result*						Change from usual**				
**Treatment**	**Visit**	**n**	**NA*** **	**much better than usual**	**better than usual**	**same as usual**	**worse than usual**	**n**	**nmiss**	**-1**	**0**	**1**
Total (n=36)	Follow-up day 15	17 (47.22%)	6 (16.67%)	1 (2.78%)	9 (25%)	1 (2.78%)		11 (30.56%)	6 (16.67%)	10 (27.78%)	1 (2.78%)	
	Follow-up day 45	17 (47.22%)	7 (19.44%)	3 (8.33%)	6 (16.67%)	1 (2.78%)		10 (27.78%)	7 (19.44%)	9 (25%)	1 (2.78%)	
NBMI100mg (n=13)	Follow-up day 15	6 (46.15%)	3 (23.08%)		3 (23.08%)			3 (23.08%)	3 (23.08%)	3 (23.08%)		
	Follow-up day 45	6 (46.15%)	4 (30.77%)		2 (15.38%)			2 (15.38%)	4 (30.77%)	2 (15.38%)		
NBMI300mg (n=11)	Follow-up day 15	5 (45.45%)	1 (9.09%)	1 (9.09%)	2 (18.18%)	1 (9.09%)		4 (36.36%)	1 (9.09%)	3 (27.27%)	1 (9.09%)	
	Follow-up day 45	5 (45.45%)	1 (9.09%)	2 (18.18%)	2 (18.18%)			4 (36.36%)	1 (9.09%)	4 (36.36%)		
Placebo (n=12)	Follow-up day 15	6 (50%)	2 (16.67%)		4 (33.33%)			4 (33.33%)	2 (16.67%)	4 (33.33%)		
	Follow-up day 45	6 (50%)	2 (16.67%)	1 (8.33%)	2 (16.67%)	1 (8.33%)		4 (33.33%)	2 (16.67%)	3 (25%)	1 (8.33%)	

Executed script: \\SDS-SERVER2\SAS$\SDS3\Projects\STATISTICS DRUG DEV\NBMI4011 - CTI002\Analyser\Script\SAS\T_mi002.sas, on: 23MAY2016:14:00, by: maria.lidsell
Version of source data: nbmi_ch1.sas7bdat, last modified: 24FEB16:23:13, status: Final
*) Scores are counted according to: much better than usual=0, better than usual=0, same as usual=1, worse than usual=2, much worse than usual=3.
**) Change for chronic mercury intoxication symptoms is defined as difference from 1=same as usual.
***) Missing results registered in database as 'NA'

Table DE5.1.1 Change from baseline of NMB, Index, Tremor Index (Full analysis set)

		Result									Change from baseline (Visit day 1)								
**Treatment**	**Visit**	**n**	**nmiss**	**Mean**	**SD**	**Q1**	**Median**	**Q3**	**Min**	**Max**	**n**	**nmiss**	**Mean**	**SD**	**Q1**	**Median**	**Q3**	**Min**	**Max**
Total (n=36)	Visit day 1	36 (100%)	0 (0%)	41.7	10.86	34	43	48	15	61									
	Follow-up day 15	36 (100%)	0 (0%)	39	13.25	27.5	38	48.5	10	62	36 (100%)	0 (0%)	-2.6	15.81	-11	-2.5	7	-36	30
	Follow-up day 45	36 (100%)	0 (0%)	36.3	16.24	27	41	50	4	60	36 (100%)	0 (0%)	-5.4	19.89	-18.5	-1	7	-41	45
NBMI100mg (n=13)	Visit day 1	13 (100%)	0 (0%)	43.3	9.05	40	44	48	24	57									
	Follow-up day 15	13 (100%)	0 (0%)	44	12.64	31	46	56	26	61	13 (100%)	0 (0%)	0.7	14.29	-11	-1	7	-20	30
	Follow-up day 45	13 (100%)	0 (0%)	39.2	16.34	33	42	51	5	58	13 (100%)	0 (0%)	-4.2	20.77	-15	-2	8	-41	27
NBMI300mg (n=11)	Visit day 1	11 (100%)	0 (0%)	37.1	13.6	27	36	44	15	61									
	Follow-up day 15	11 (100%)	0 (0%)	39.2	11.01	32	39	45	22	62	11 (100%)	0 (0%)	2.1	13.58	-6	0	14	-26	22
	Follow-up day 45	11 (100%)	0 (0%)	33.2	16.93	22	30	46	8	60	11 (100%)	0 (0%)	-3.9	20.7	-19	-8	7	-31	45
Placebo (n=12)	Visit day 1	12 (100%)	0 (0%)	44.1	9.35	36	46	50.5	31	58									
	Follow-up day 15	12 (100%)	0 (0%)	33.5	14.54	25	31	42	10	60	12 (100%)	0 (0%)	-10.6	17.35	-26.5	-8.5	-1.5	-36	23
	Follow-up day 45	12 (100%)	0 (0%)	36	16.37	29	39.5	48	4	54	12 (100%)	0 (0%)	-8.1	19.67	-26	1	7	-38	13

Executed script: \\SDS-SERVER2\SAS$\SDS3\Projects\STATISTICS DRUG DEV\NBMI4011 - CTI002\Analyser\Script\SAS\T_cat001.sas, on: 23MAY2016:14:00, by: maria.lidsell
Version of source data: nbmi_neu.sas7bdat, last modified: 24FEB16:23:13, status: Final

Table DE5.2.1 Change from baseline of NMB, Index, Sway Index (Full analysis set)

		Result									Change from baseline (Visit day 1)								
**Treatment**	**Visit**	**n**	**nmiss**	**Mean**	**SD**	**Q1**	**Median**	**Q3**	**Min**	**Max**	**n**	**nmiss**	**Mean**	**SD**	**Q1**	**Median**	**Q3**	**Min**	**Max**
Total (n=36)	Visit day 1	36 (100%)	0 (0%)	175.6	12.39	170.5	179.5	182.5	143	195									
	Follow-up day 15	36 (100%)	0 (0%)	172.1	22.82	167	179	186	96	195	36 (100%)	0 (0%)	-3.5	24.63	-11	-1	6.5	-99	43
	Follow-up day 45	36 (100%)	0 (0%)	164.6	34.93	166.5	172	182.5	12	195	36 (100%)	0 (0%)	-11	31.91	-13	-4	4.5	-149	25
NBMI100mg (n=13)	Visit day 1	13 (100%)	0 (0%)	176.5	10.63	177	180	182	154	188									
	Follow-up day 15	13 (100%)	0 (0%)	178.6	15.37	177	184	188	144	195	13 (100%)	0 (0%)	2.2	13.02	-2	0	8	-25	31
	Follow-up day 45	13 (100%)	0 (0%)	158.8	51.57	165	179	186	12	195	13 (100%)	0 (0%)	-17.7	44.23	-14	-1	4	-149	17
NBMI300mg (n=11)	Visit day 1	11 (100%)	0 (0%)	175.9	10.85	170	175	183	152	195									
	Follow-up day 15	11 (100%)	0 (0%)	162.7	30.94	157	171	182	96	194	11 (100%)	0 (0%)	-13.2	36.84	-17	-1	5	-99	28
	Follow-up day 45	11 (100%)	0 (0%)	169.8	27.49	168	177	183	90	191	11 (100%)	0 (0%)	-6.1	28.57	-11	-3	8	-85	25
Placebo (n=12)	Visit day 1	12 (100%)	0 (0%)	174.5	16	164	180	184	143	195									
	Follow-up day 15	12 (100%)	0 (0%)	173.8	19.71	170	178	183.5	121	194	12 (100%)	0 (0%)	-0.8	19.27	-14	-3	8.5	-25	43
	Follow-up day 45	12 (100%)	0 (0%)	166.2	14.95	159.5	168	173.5	139	193	12 (100%)	0 (0%)	-8.3	16.74	-19.5	-7	-1	-37	24

Executed script: \\SDS-SERVER2\SAS$\SDS3\Projects\STATISTICS DRUG DEV\NBMI4011 - CTI002\Analyser\Script\SAS\T_cat001.sas, on: 23MAY2016:14:00, by: maria.lidsell
Version of source data: nbmi_neu.sas7bdat, last modified: 24FEB16:23:13, status: Final

Table DE5.3.1 Change from baseline of NMB, Index, Catsys Index (Full analysis set)

		Result									Change from baseline (Visit day 1)								
**Treatment**	**Visit**	**n**	**nmiss**	**Mean**	**SD**	**Q1**	**Median**	**Q3**	**Min**	**Max**	**n**	**nmiss**	**Mean**	**SD**	**Q1**	**Median**	**Q3**	**Min**	**Max**
Total (n=36)	Visit day 1	36 (100%)	0 (0%)	93	23.91	72	87.5	107	59	148									
	Follow-up day 15	36 (100%)	0 (0%)	99.5	28.44	78	100.5	121.5	29	152	36 (100%)	0 (0%)	6.5	23.46	-10	7.5	23	-57	44
	Follow-up day 45	36 (100%)	0 (0%)	99.8	27.73	76	99	122	48	158	36 (100%)	0 (0%)	6.8	29.45	-11	3.5	21	-55	71
NBMI100mg (n=13)	Visit day 1	13 (100%)	0 (0%)	80.2	19.4	64	72	88	62	129									
	Follow-up day 15	13 (100%)	0 (0%)	92.2	27.46	76	95	108	29	134	13 (100%)	0 (0%)	12	20.66	5	12	23	-34	44
	Follow-up day 45	13 (100%)	0 (0%)	92.5	26.68	68	92	113	48	127	13 (100%)	0 (0%)	12.4	30.26	-14	6	26	-33	64
NBMI300mg (n=11)	Visit day 1	11 (100%)	0 (0%)	105	18.05	87	105	116	80	139									
	Follow-up day 15	11 (100%)	0 (0%)	101	31.17	80	98	129	48	144	11 (100%)	0 (0%)	-4	27.31	-24	5	17	-57	33
	Follow-up day 45	11 (100%)	0 (0%)	105.6	28.49	77	106	128	61	152	11 (100%)	0 (0%)	0.6	31.11	-29	-3	20	-44	71
Placebo (n=12)	Visit day 1	12 (100%)	0 (0%)	96	27.64	77	89	109	59	148									
	Follow-up day 15	12 (100%)	0 (0%)	106.1	27.48	81	112.5	127	65	152	12 (100%)	0 (0%)	10.1	21.16	-3	13.5	24	-33	40
	Follow-up day 45	12 (100%)	0 (0%)	102.4	28.79	80.5	90	128.5	70	158	12 (100%)	0 (0%)	6.4	28.36	-5.5	7	17.5	-55	53

Executed script: \\SDS-SERVER2\SAS$\SDS3\Projects\STATISTICS DRUG DEV\NBMI4011 - CTI002\Analyser\Script\SAS\T_cat001.sas, on: 23MAY2016:14:00, by: maria.lidsell
Version of source data: nbmi_neu.sas7bdat, last modified: 24FEB16:23:13, status: Final

Table DE5.4.1 Change from baseline of NMB, Tremor test, Right hand, Tremor Intensity (Full analysis set)

		Result									Change from baseline (Visit day 1)								
**Treatment**	**Visit**	**n**	**nmiss**	**Mean**	**SD**	**Q1**	**Median**	**Q3**	**Min**	**Max**	**n**	**nmiss**	**Mean**	**SD**	**Q1**	**Median**	**Q3**	**Min**	**Max**
Total (n=36)	Visit day 1	36 (100%)	0 (0%)	0.1	0.07	0.07	0.09	0.1	0.04	0.48									
	Follow-up day 15	36 (100%)	0 (0%)	0.2	0.5	0.07	0.09	0.12	0.04	2.96	36 (100%)	0 (0%)	0.1	0.51	-0.01	0.01	0.03	-0.25	2.87
	Follow-up day 45	36 (100%)	0 (0%)	0.6	1.37	0.08	0.1	0.18	0.05	5.91	36 (100%)	0 (0%)	0.5	1.38	0	0.02	0.09	-0.36	5.85
NBMI100mg (n=13)	Visit day 1	13 (100%)	0 (0%)	0.1	0.02	0.08	0.08	0.09	0.06	0.13									
	Follow-up day 15	13 (100%)	0 (0%)	0.1	0.02	0.07	0.09	0.1	0.05	0.14	13 (100%)	0 (0%)	0	0.02	-0.01	0	0.02	-0.03	0.06
	Follow-up day 45	13 (100%)	0 (0%)	0.3	0.71	0.09	0.1	0.16	0.08	2.66	13 (100%)	0 (0%)	0.3	0.71	0.01	0.02	0.08	-0.03	2.58
NBMI300mg (n=11)	Visit day 1	11 (100%)	0 (0%)	0.1	0.12	0.06	0.09	0.09	0.05	0.48									
	Follow-up day 15	11 (100%)	0 (0%)	0.1	0.06	0.06	0.09	0.12	0.06	0.23	11 (100%)	0 (0%)	0	0.09	-0.01	0.01	0.04	-0.25	0.12
	Follow-up day 45	11 (100%)	0 (0%)	1.1	1.86	0.07	0.1	2.25	0.05	5.91	11 (100%)	0 (0%)	0.9	1.89	-0.02	0.01	2.19	-0.36	5.85
Placebo (n=12)	Visit day 1	12 (100%)	0 (0%)	0.1	0.04	0.08	0.09	0.11	0.04	0.19									
	Follow-up day 15	12 (100%)	0 (0%)	0.4	0.85	0.07	0.105	0.18	0.04	2.96	12 (100%)	0 (0%)	0.3	0.85	-0.005	0.01	0.06	-0.07	2.87
	Follow-up day 45	12 (100%)	0 (0%)	0.6	1.4	0.09	0.115	0.17	0.05	4.99	12 (100%)	0 (0%)	0.5	1.4	-0.005	0.035	0.09	-0.07	4.9

Executed script: \\SDS-SERVER2\SAS$\SDS3\Projects\STATISTICS DRUG DEV\NBMI4011 - CTI002\Analyser\Script\SAS\T_cat001.sas, on: 23MAY2016:14:00, by: maria.lidsell
Version of source data: nbmi_neu.sas7bdat, last modified: 24FEB16:23:13, status: Final

Table DE5.5.1 Change from baseline of NMB, Tremor test, Right hand, Centre Frequency (Full analysis set)

		Result									Change from baseline (Visit day 1)								
**Treatment**	**Visit**	**n**	**nmiss**	**Mean**	**SD**	**Q1**	**Median**	**Q3**	**Min**	**Max**	**n**	**nmiss**	**Mean**	**SD**	**Q1**	**Median**	**Q3**	**Min**	**Max**
Total (n=36)	Visit day 1	36 (100%)	0 (0%)	8.9	8.92	6.1	7.7	9.15	4	60									
	Follow-up day 15	36 (100%)	0 (0%)	6.9	2.12	5.95	7.2	8.2	0.9	11.4	36 (100%)	0 (0%)	-2	9.03	-1.6	-0.5	0.5	-53.5	4.9
	Follow-up day 45	36 (100%)	0 (0%)	6.3	2.79	4.8	7.1	8.3	0.9	11.8	36 (100%)	0 (0%)	-2.7	9.21	-2.3	-0.95	0.2	-53.5	6.1
NBMI100mg (n=13)	Visit day 1	13 (100%)	0 (0%)	7.4	1.26	7.2	7.7	7.9	4.5	9.4									
	Follow-up day 15	13 (100%)	0 (0%)	7.7	1.65	7	7.4	8.2	5.5	11.4	13 (100%)	0 (0%)	0.3	1.85	-0.5	-0.3	0.5	-3	4.9
	Follow-up day 45	13 (100%)	0 (0%)	7.5	2.33	7	7.7	8.4	1.3	11.8	13 (100%)	0 (0%)	0.1	2.12	-0.7	-0.2	0.2	-3.2	6.1
NBMI300mg (n=11)	Visit day 1	11 (100%)	0 (0%)	7.2	1.81	6	7.4	8.9	4	9.6									
	Follow-up day 15	11 (100%)	0 (0%)	6.8	2.02	4.8	6.7	8.4	4	10.4	11 (100%)	0 (0%)	-0.4	1.49	-1.7	-0.5	0.8	-2.9	1.9
	Follow-up day 45	11 (100%)	0 (0%)	5.3	3.11	1.1	6.7	8.2	0.9	8.7	11 (100%)	0 (0%)	-1.9	3.2	-3.9	-1	1	-8.5	1.7
Placebo (n=12)	Visit day 1	12 (100%)	0 (0%)	12.1	15.22	5.7	8.4	10	5.2	60									
	Follow-up day 15	12 (100%)	0 (0%)	6.3	2.55	5.4	6.85	8.05	0.9	8.9	12 (100%)	0 (0%)	-5.8	15.14	-3.6	-1.5	-0.45	-53.5	2.5
	Follow-up day 45	12 (100%)	0 (0%)	5.8	2.66	4.65	5.85	7.9	1.1	9.4	12 (100%)	0 (0%)	-6.3	15.22	-4.5	-2.1	-0.7	-53.5	4.2

Executed script: \\SDS-SERVER2\SAS$\SDS3\Projects\STATISTICS DRUG DEV\NBMI4011 - CTI002\Analyser\Script\SAS\T_cat001.sas, on: 23MAY2016:14:00, by: maria.lidsell
Version of source data: nbmi_neu.sas7bdat, last modified: 24FEB16:23:13, status: Final

Table DE5.6.1 Change from baseline of NMB, Tremor test, Right hand, Standard Deviation (Full analysis set)

		Result									Change from baseline (Visit day 1)								
**Treatment**	**Visit**	**n**	**nmiss**	**Mean**	**SD**	**Q1**	**Median**	**Q3**	**Min**	**Max**	**n**	**nmiss**	**Mean**	**SD**	**Q1**	**Median**	**Q3**	**Min**	**Max**
Total (n=36)	Visit day 1	26 (72.22%)	10 (27.78%)	3.1	0.82	2.7	2.9	3.4	1	5.1									
	Follow-up day 15	23 (63.89%)	13 (36.11%)	4.2	5.07	2.9	3.2	3.9	0.2	27	22 (61.11%)	14 (38.89%)	1.3	5.17	-0.3	0.25	0.9	-2.2	24.1
	Follow-up day 45	4 (11.11%)	32 (88.89%)	3.4	0.46	3.05	3.45	3.8	2.9	3.9	4 (11.11%)	32 (88.89%)	0.2	0.72	-0.35	0.4	0.8	-0.7	0.8
NBMI100mg (n=13)	Visit day 1	10 (76.92%)	3 (23.08%)	2.9	0.72	2.4	2.7	3.2	2.2	4.6									
	Follow-up day 15	9 (69.23%)	4 (30.77%)	2.9	0.68	2.7	2.9	2.9	2	4.4	9 (69.23%)	4 (30.77%)	0	0.52	-0.3	0	0.3	-1	0.7
	Follow-up day 45	3 (23.08%)	10 (76.92%)	3.6	0.36	3.2	3.7	3.9	3.2	3.9	3 (23.08%)	10 (76.92%)	0.3	0.87	-0.7	0.8	0.8	-0.7	0.8
NBMI300mg (n=11)	Visit day 1	7 (63.64%)	4 (36.36%)	3.2	1.28	2.7	3.2	4.2	1	5.1									
	Follow-up day 15	7 (63.64%)	4 (36.36%)	3.5	0.81	3.2	3.4	3.9	2	4.6	7 (63.64%)	4 (36.36%)	0.3	1.38	-0.5	0.5	1.2	-2.2	2.2
	Follow-up day 45	0 (0%)	11 (100%)								0 (0%)	11 (100%)							
Placebo (n=12)	Visit day 1	9 (75%)	3 (25%)	3.2	0.43	2.9	3.2	3.4	2.4	3.7									
	Follow-up day 15	7 (58.33%)	5 (41.67%)	6.6	9.1	2.9	3.9	4.6	0.2	27	6 (50%)	6 (50%)	4.6	9.57	0	1.2	1.5	-0.3	24.1
	Follow-up day 45	1 (8.33%)	11 (91.67%)	2.9		2.9	2.9	2.9	2.9	2.9	1 (8.33%)	11 (91.67%)	0		0	0	0	0	0

Executed script: \\SDS-SERVER2\SAS$\SDS3\Projects\STATISTICS DRUG DEV\NBMI4011 - CTI002\Analyser\Script\SAS\T_cat001.sas, on: 23MAY2016:14:00, by: maria.lidsell
Version of source data: nbmi_neu.sas7bdat, last modified: 24FEB16:23:13, status: Final

Table DE5.7.1 Change from baseline of NMB, Tremor test, Right hand, Harmonic Index (Full analysis set)

		Result									Change from baseline (Visit day 1)								
**Treatment**	**Visit**	**n**	**nmiss**	**Mean**	**SD**	**Q1**	**Median**	**Q3**	**Min**	**Max**	**n**	**nmiss**	**Mean**	**SD**	**Q1**	**Median**	**Q3**	**Min**	**Max**
Total (n=36)	Visit day 1	36 (100%)	0 (0%)	0.9	0.06	0.81	0.875	0.905	0.73	0.95									
	Follow-up day 15	36 (100%)	0 (0%)	0.9	0.06	0.82	0.87	0.905	0.69	0.97	36 (100%)	0 (0%)	0	0.08	-0.04	-0.005	0.05	-0.22	0.16
	Follow-up day 45	36 (100%)	0 (0%)	0.9	0.07	0.825	0.885	0.925	0.7	0.97	36 (100%)	0 (0%)	0	0.07	-0.05	0	0.065	-0.1	0.18
NBMI100mg (n=13)	Visit day 1	13 (100%)	0 (0%)	0.9	0.05	0.88	0.9	0.92	0.79	0.95									
	Follow-up day 15	13 (100%)	0 (0%)	0.9	0.06	0.84	0.88	0.91	0.77	0.95	13 (100%)	0 (0%)	0	0.07	-0.04	-0.01	0	-0.13	0.12
	Follow-up day 45	13 (100%)	0 (0%)	0.9	0.07	0.83	0.88	0.91	0.7	0.96	13 (100%)	0 (0%)	0	0.05	-0.06	-0.03	0	-0.09	0.08
NBMI300mg (n=11)	Visit day 1	11 (100%)	0 (0%)	0.8	0.07	0.77	0.84	0.89	0.73	0.93									
	Follow-up day 15	11 (100%)	0 (0%)	0.8	0.06	0.78	0.85	0.88	0.75	0.94	11 (100%)	0 (0%)	0	0.06	-0.04	0.01	0.05	-0.07	0.1
	Follow-up day 45	11 (100%)	0 (0%)	0.9	0.09	0.78	0.86	0.95	0.74	0.97	11 (100%)	0 (0%)	0	0.08	-0.07	0.05	0.09	-0.1	0.17
Placebo (n=12)	Visit day 1	12 (100%)	0 (0%)	0.8	0.05	0.805	0.845	0.88	0.74	0.91									
	Follow-up day 15	12 (100%)	0 (0%)	0.9	0.08	0.825	0.86	0.915	0.69	0.97	12 (100%)	0 (0%)	0	0.1	-0.03	0.03	0.09	-0.22	0.16
	Follow-up day 45	12 (100%)	0 (0%)	0.9	0.06	0.845	0.89	0.925	0.78	0.97	12 (100%)	0 (0%)	0	0.07	-0.005	0.025	0.095	-0.06	0.18

Executed script: \\SDS-SERVER2\SAS$\SDS3\Projects\STATISTICS DRUG DEV\NBMI4011 - CTI002\Analyser\Script\SAS\T_cat001.sas, on: 23MAY2016:14:00, by: maria.lidsell
Version of source data: nbmi_neu.sas7bdat, last modified: 24FEB16:23:13, status: Final

Table DE5.8.1 Change from baseline of NMB, Tremor test, Right hand, Tremor Index (Full analysis set)

		Result									Change from baseline (Visit day 1)								
**Treatment**	**Visit**	**n**	**nmiss**	**Mean**	**SD**	**Q1**	**Median**	**Q3**	**Min**	**Max**	**n**	**nmiss**	**Mean**	**SD**	**Q1**	**Median**	**Q3**	**Min**	**Max**
Total (n=36)	Visit day 1	36 (100%)	0 (0%)	42.3	14.63	30	44.5	53.5	11	71									
	Follow-up day 15	36 (100%)	0 (0%)	39.9	17.39	28	40	53.5	5	77	36 (100%)	0 (0%)	-2.4	19.37	-12	1	15	-48	24
	Follow-up day 45	36 (100%)	0 (0%)	36.8	20.23	24.5	34.5	54.5	5	72	36 (100%)	0 (0%)	-5.5	23	-23	-1.5	10	-53	38
NBMI100mg (n=13)	Visit day 1	13 (100%)	0 (0%)	45.2	13.39	38	45	54	21	71									
	Follow-up day 15	13 (100%)	0 (0%)	45.7	19.26	29	52	56	10	77	13 (100%)	0 (0%)	0.5	19.97	-12	2	17	-31	24
	Follow-up day 45	13 (100%)	0 (0%)	41.5	18.91	28	41	55	7	72	13 (100%)	0 (0%)	-3.8	22.76	-17	-10	11	-38	35
NBMI300mg (n=11)	Visit day 1	11 (100%)	0 (0%)	35.2	16.28	24	30	49	11	60									
	Follow-up day 15	11 (100%)	0 (0%)	40.2	12.82	32	40	50	17	61	11 (100%)	0 (0%)	5	12.85	1	5	10	-24	24
	Follow-up day 45	11 (100%)	0 (0%)	29.5	19.01	7	27	49	5	57	11 (100%)	0 (0%)	-5.7	25.31	-30	4	8	-53	38
Placebo (n=12)	Visit day 1	12 (100%)	0 (0%)	45.8	13.07	36	49	54.5	25	68									
	Follow-up day 15	12 (100%)	0 (0%)	33.5	17.99	26	30.5	43	5	70	12 (100%)	0 (0%)	-12.3	20.93	-31	-7.5	0	-48	19
	Follow-up day 45	12 (100%)	0 (0%)	38.5	22.37	25.5	31	61.5	6	70	12 (100%)	0 (0%)	-7.3	23	-28	-3	11.5	-42	30

Executed script: \\SDS-SERVER2\SAS$\SDS3\Projects\STATISTICS DRUG DEV\NBMI4011 - CTI002\Analyser\Script\SAS\T_cat001.sas, on: 23MAY2016:14:00, by: maria.lidsell
Version of source data: nbmi_neu.sas7bdat, last modified: 24FEB16:23:13, status: Final

Table DE5.9.1 Change from baseline of NMB, Tremor test, Left hand, Tremor Intensity (Full analysis set)

		Result									Change from baseline (Visit day 1)								
**Treatment**	**Visit**	**n**	**nmiss**	**Mean**	**SD**	**Q1**	**Median**	**Q3**	**Min**	**Max**	**n**	**nmiss**	**Mean**	**SD**	**Q1**	**Median**	**Q3**	**Min**	**Max**
Total (n=36)	Visit day 1	36 (100%)	0 (0%)	0.1	0.07	0.07	0.085	0.11	0.05	0.5									
	Follow-up day 15	36 (100%)	0 (0%)	0.2	0.49	0.07	0.09	0.12	0.06	3.03	36 (100%)	0 (0%)	0.1	0.49	-0.01	0.005	0.02	-0.22	2.92
	Follow-up day 45	36 (100%)	0 (0%)	0.4	0.66	0.08	0.12	0.175	0.05	2.83	36 (100%)	0 (0%)	0.3	0.66	0	0.02	0.085	-0.37	2.71
NBMI100mg (n=13)	Visit day 1	13 (100%)	0 (0%)	0.1	0.03	0.07	0.08	0.11	0.05	0.15									
	Follow-up day 15	13 (100%)	0 (0%)	0.1	0.02	0.07	0.07	0.09	0.06	0.13	13 (100%)	0 (0%)	0	0.03	-0.03	-0.01	0.01	-0.06	0.05
	Follow-up day 45	13 (100%)	0 (0%)	0.4	0.77	0.08	0.09	0.13	0.05	2.83	13 (100%)	0 (0%)	0.3	0.76	0	0.01	0.04	-0.03	2.71
NBMI300mg (n=11)	Visit day 1	11 (100%)	0 (0%)	0.1	0.13	0.07	0.08	0.11	0.06	0.5									
	Follow-up day 15	11 (100%)	0 (0%)	0.1	0.07	0.08	0.09	0.11	0.06	0.28	11 (100%)	0 (0%)	0	0.07	-0.01	0.01	0.02	-0.22	0.06
	Follow-up day 45	11 (100%)	0 (0%)	0.3	0.36	0.08	0.13	0.25	0.08	1.13	11 (100%)	0 (0%)	0.2	0.4	0	0.02	0.17	-0.37	1.07
Placebo (n=12)	Visit day 1	12 (100%)	0 (0%)	0.1	0.03	0.075	0.09	0.125	0.06	0.15									
	Follow-up day 15	12 (100%)	0 (0%)	0.4	0.84	0.08	0.12	0.165	0.06	3.03	12 (100%)	0 (0%)	0.3	0.84	0	0.01	0.04	-0.01	2.92
	Follow-up day 45	12 (100%)	0 (0%)	0.4	0.77	0.09	0.13	0.185	0.06	2.7	12 (100%)	0 (0%)	0.3	0.76	-0.005	0.045	0.085	-0.04	2.59

Executed script: \\SDS-SERVER2\SAS$\SDS3\Projects\STATISTICS DRUG DEV\NBMI4011 - CTI002\Analyser\Script\SAS\T_cat001.sas, on: 23MAY2016:14:00, by: maria.lidsell
Version of source data: nbmi_neu.sas7bdat, last modified: 24FEB16:23:13, status: Final

Table DE5.10.1 Change from baseline of NMB, Tremor test, Left hand, Centre Frequency (Full analysis set)

		Result									Change from baseline (Visit day 1)								
**Treatment**	**Visit**	**n**	**nmiss**	**Mean**	**SD**	**Q1**	**Median**	**Q3**	**Min**	**Max**	**n**	**nmiss**	**Mean**	**SD**	**Q1**	**Median**	**Q3**	**Min**	**Max**
Total (n=36)	Visit day 1	36 (100%)	0 (0%)	7.4	1.61	6	7.7	8.55	4.5	10.6									
	Follow-up day 15	36 (100%)	0 (0%)	7	1.96	6	7.2	8.3	1.8	10.9	36 (100%)	0 (0%)	-0.4	2.05	-1.7	0	1.1	-6.6	3.2
	Follow-up day 45	36 (100%)	0 (0%)	6.7	2.79	5	7	8.8	0.9	12.3	36 (100%)	0 (0%)	-0.8	3.04	-2.3	-0.4	1.1	-7.5	6.1
NBMI100mg (n=13)	Visit day 1	13 (100%)	0 (0%)	7.2	1.24	6	7.2	7.9	5.5	9.6									
	Follow-up day 15	13 (100%)	0 (0%)	7.4	1.33	7.2	7.4	8.2	4.3	9.4	13 (100%)	0 (0%)	0.2	1.88	-0.5	0.2	1.4	-3.6	3.2
	Follow-up day 45	13 (100%)	0 (0%)	6.6	2.77	6	6.7	7.4	0.9	12.3	13 (100%)	0 (0%)	-0.5	3.06	-1.7	-0.3	1.2	-5.6	6.1
NBMI300mg (n=11)	Visit day 1	11 (100%)	0 (0%)	7.5	1.64	5.7	7.9	8.4	5	10.1									
	Follow-up day 15	11 (100%)	0 (0%)	7.4	1.71	6.2	7.4	8.4	4.5	10.4	11 (100%)	0 (0%)	-0.2	1.28	-1.3	-0.2	0.8	-2	2
	Follow-up day 45	11 (100%)	0 (0%)	6.6	2.67	4.3	7	8.9	2.6	9.9	11 (100%)	0 (0%)	-1	2.63	-2.6	-1.2	1	-6.1	3.9
Placebo (n=12)	Visit day 1	12 (100%)	0 (0%)	7.6	2.01	6	7.45	9.15	4.5	10.6									
	Follow-up day 15	12 (100%)	0 (0%)	6.4	2.62	4.8	6.35	7.95	1.8	10.9	12 (100%)	0 (0%)	-1.2	2.61	-3.15	-0.85	0.75	-6.6	2.2
	Follow-up day 45	12 (100%)	0 (0%)	6.8	3.14	4.9	7.2	9.25	0.9	11.8	12 (100%)	0 (0%)	-0.8	3.58	-3.3	0.25	1.05	-7.5	5.3

Executed script: \\SDS-SERVER2\SAS$\SDS3\Projects\STATISTICS DRUG DEV\NBMI4011 - CTI002\Analyser\Script\SAS\T_cat001.sas, on: 23MAY2016:14:00, by: maria.lidsell
Version of source data: nbmi_neu.sas7bdat, last modified: 24FEB16:23:13, status: Final

Table DE5.11.1 Change from baseline of NMB, Tremor test, Left hand, Standard Deviation (Full analysis set)

		Result									Change from baseline (Visit day 1)								
**Treatment**	**Visit**	**n**	**nmiss**	**Mean**	**SD**	**Q1**	**Median**	**Q3**	**Min**	**Max**	**n**	**nmiss**	**Mean**	**SD**	**Q1**	**Median**	**Q3**	**Min**	**Max**
Total (n=36)	Visit day 1	26 (72.22%)	10 (27.78%)	3.3	0.96	2.7	3.4	3.9	0.7	4.9									
	Follow-up day 15	23 (63.89%)	13 (36.11%)	3.7	0.94	3.2	3.4	4.2	1.5	5.6	22 (61.11%)	14 (38.89%)	0.3	1.27	-0.5	0.3	0.8	-2.9	2.5
	Follow-up day 45	4 (11.11%)	32 (88.89%)	3.6	0.48	3.2	3.45	3.95	3.2	4.2	4 (11.11%)	32 (88.89%)	1	0.89	0.3	1.15	1.65	-0.2	1.8
NBMI100mg (n=13)	Visit day 1	10 (76.92%)	3 (23.08%)	3.3	0.98	2.4	3.55	3.9	1.7	4.9									
	Follow-up day 15	9 (69.23%)	4 (30.77%)	4.1	0.59	3.4	4.2	4.4	3.2	4.9	9 (69.23%)	4 (30.77%)	0.8	1.28	0.4	0.8	1.8	-1.5	2.5
	Follow-up day 45	3 (23.08%)	10 (76.92%)	3.4	0.29	3.2	3.2	3.7	3.2	3.7	3 (23.08%)	10 (76.92%)	0.7	0.85	-0.2	0.8	1.5	-0.2	1.5
NBMI300mg (n=11)	Visit day 1	7 (63.64%)	4 (36.36%)	3.3	1.28	2.9	3.4	4.2	0.7	4.6									
	Follow-up day 15	7 (63.64%)	4 (36.36%)	3.7	1.24	3.2	3.7	4.2	1.5	5.6	7 (63.64%)	4 (36.36%)	0.4	0.96	-0.4	0	0.8	-0.5	2.2
	Follow-up day 45	0 (0%)	11 (100%)								0 (0%)	11 (100%)							
Placebo (n=12)	Visit day 1	9 (75%)	3 (25%)	3.4	0.76	2.7	3.4	3.9	2.4	4.6									
	Follow-up day 15	7 (58.33%)	5 (41.67%)	3.1	0.8	2.9	3.2	3.4	1.7	4.4	6 (50%)	6 (50%)	-0.6	1.28	-1	-0.35	0.2	-2.9	0.7
	Follow-up day 45	1 (8.33%)	11 (91.67%)	4.2		4.2	4.2	4.2	4.2	4.2	1 (8.33%)	11 (91.67%)	1.8		1.8	1.8	1.8	1.8	1.8

Executed script: \\SDS-SERVER2\SAS$\SDS3\Projects\STATISTICS DRUG DEV\NBMI4011 - CTI002\Analyser\Script\SAS\T_cat001.sas, on: 23MAY2016:14:00, by: maria.lidsell
Version of source data: nbmi_neu.sas7bdat, last modified: 24FEB16:23:13, status: Final

Table DE5.12.1 Change from baseline of NMB, Tremor test, Left hand, Harmonic Index (Full analysis set)

		Result									Change from baseline (Visit day 1)								
**Treatment**	**Visit**	**n**	**nmiss**	**Mean**	**SD**	**Q1**	**Median**	**Q3**	**Min**	**Max**	**n**	**nmiss**	**Mean**	**SD**	**Q1**	**Median**	**Q3**	**Min**	**Max**
Total (n=36)	Visit day 1	36 (100%)	0 (0%)	0.8	0.05	0.79	0.83	0.86	0.75	0.97									
	Follow-up day 15	36 (100%)	0 (0%)	0.8	0.06	0.77	0.82	0.87	0.68	0.95	36 (100%)	0 (0%)	0	0.08	-0.07	-0.005	0.05	-0.16	0.17
	Follow-up day 45	36 (100%)	0 (0%)	0.9	0.06	0.815	0.855	0.89	0.74	0.98	36 (100%)	0 (0%)	0	0.09	-0.035	0.035	0.09	-0.14	0.22
NBMI100mg (n=13)	Visit day 1	13 (100%)	0 (0%)	0.8	0.05	0.78	0.81	0.86	0.77	0.91									
	Follow-up day 15	13 (100%)	0 (0%)	0.8	0.06	0.77	0.81	0.86	0.71	0.92	13 (100%)	0 (0%)	0	0.08	-0.08	0	0.03	-0.13	0.11
	Follow-up day 45	13 (100%)	0 (0%)	0.9	0.06	0.81	0.84	0.88	0.77	0.98	13 (100%)	0 (0%)	0	0.09	-0.04	0.04	0.1	-0.11	0.15
NBMI300mg (n=11)	Visit day 1	11 (100%)	0 (0%)	0.8	0.05	0.79	0.83	0.87	0.78	0.97									
	Follow-up day 15	11 (100%)	0 (0%)	0.8	0.05	0.8	0.82	0.87	0.76	0.91	11 (100%)	0 (0%)	0	0.06	-0.06	-0.03	0.03	-0.08	0.1
	Follow-up day 45	11 (100%)	0 (0%)	0.9	0.06	0.82	0.85	0.89	0.76	0.94	11 (100%)	0 (0%)	0	0.08	-0.03	0.01	0.08	-0.11	0.15
Placebo (n=12)	Visit day 1	12 (100%)	0 (0%)	0.8	0.04	0.815	0.84	0.855	0.75	0.9									
	Follow-up day 15	12 (100%)	0 (0%)	0.8	0.08	0.77	0.84	0.895	0.68	0.95	12 (100%)	0 (0%)	0	0.11	-0.08	0.01	0.075	-0.16	0.17
	Follow-up day 45	12 (100%)	0 (0%)	0.9	0.07	0.815	0.875	0.91	0.74	0.97	12 (100%)	0 (0%)	0	0.1	-0.04	0.045	0.09	-0.14	0.22

Executed script: \\SDS-SERVER2\SAS$\SDS3\Projects\STATISTICS DRUG DEV\NBMI4011 - CTI002\Analyser\Script\SAS\T_cat001.sas, on: 23MAY2016:14:00, by: maria.lidsell
Version of source data: nbmi_neu.sas7bdat, last modified: 24FEB16:23:13, status: Final

Table DE5.13.1 Change from baseline of NMB, Tremor test, Left hand, Tremor Index (Full analysis set)

		Result									Change from baseline (Visit day 1)								
**Treatment**	**Visit**	**n**	**nmiss**	**Mean**	**SD**	**Q1**	**Median**	**Q3**	**Min**	**Max**	**n**	**nmiss**	**Mean**	**SD**	**Q1**	**Median**	**Q3**	**Min**	**Max**
Total (n=36)	Visit day 1	36 (100%)	0 (0%)	41	13.47	32.5	40	51	11	65									
	Follow-up day 15	36 (100%)	0 (0%)	37.8	13.7	28.5	36	47	11	65	36 (100%)	0 (0%)	-3.2	18.34	-15	-5.5	4	-33	44
	Follow-up day 45	36 (100%)	0 (0%)	35.7	17.34	27	41	46.5	2	65	36 (100%)	0 (0%)	-5.3	23.3	-24.5	-8	14	-59	51
NBMI100mg (n=13)	Visit day 1	13 (100%)	0 (0%)	41.1	14.04	31	40	51	21	64									
	Follow-up day 15	13 (100%)	0 (0%)	41.3	12.71	32	43	51	22	65	13 (100%)	0 (0%)	0.2	19.79	-10	-3	9	-33	44
	Follow-up day 45	13 (100%)	0 (0%)	36.5	17.28	28	41	46	2	61	13 (100%)	0 (0%)	-4.5	25.19	-18	-12	14	-59	34
NBMI300mg (n=11)	Visit day 1	11 (100%)	0 (0%)	39	16.74	28	37	51	11	65									
	Follow-up day 15	11 (100%)	0 (0%)	38.2	13.37	30	36	46	20	64	11 (100%)	0 (0%)	-0.8	19.41	-15	-6	19	-30	36
	Follow-up day 45	11 (100%)	0 (0%)	36.7	18.95	20	41	47	8	65	11 (100%)	0 (0%)	-2.3	24.47	-25	-7	14	-29	51
Placebo (n=12)	Visit day 1	12 (100%)	0 (0%)	42.7	10	36	41	50.5	26	59									
	Follow-up day 15	12 (100%)	0 (0%)	33.6	14.98	23.5	31	43	11	65	12 (100%)	0 (0%)	-9.1	15.61	-19	-9.5	-2.5	-32	29
	Follow-up day 45	12 (100%)	0 (0%)	33.8	17.26	20.5	39.5	47.5	3	54	12 (100%)	0 (0%)	-8.9	21.61	-31	-8	13.5	-40	18

Executed script: \\SDS-SERVER2\SAS$\SDS3\Projects\STATISTICS DRUG DEV\NBMI4011 - CTI002\Analyser\Script\SAS\T_cat001.sas, on: 23MAY2016:14:00, by: maria.lidsell
Version of source data: nbmi_neu.sas7bdat, last modified: 24FEB16:23:13, status: Final

Table DE5.14.1 Change from baseline of NMB, Sway test, Mean-Sway (Full analysis set)

		Result									Change from baseline (Visit day 1)								
**Treatment**	**Visit**	**n**	**nmiss**	**Mean**	**SD**	**Q1**	**Median**	**Q3**	**Min**	**Max**	**n**	**nmiss**	**Mean**	**SD**	**Q1**	**Median**	**Q3**	**Min**	**Max**
Total (n=36)	Visit day 1	36 (100%)	0 (0%)	4.5	1.49	3.9	4.4	5.3	0.7	7.3									
	Follow-up day 15	36 (100%)	0 (0%)	6.1	10.62	3.3	4.5	5.6	0.7	67	36 (100%)	0 (0%)	1.6	10.67	-0.9	0.2	1	-5.6	62.2
	Follow-up day 45	36 (100%)	0 (0%)	5.2	2.39	3.9	5.15	5.85	0.5	14.4	36 (100%)	0 (0%)	0.7	2.11	-0.75	0.3	1.7	-3.9	8.2
NBMI100mg (n=13)	Visit day 1	13 (100%)	0 (0%)	4.5	1.18	3.9	4.4	4.8	2.8	6.6									
	Follow-up day 15	13 (100%)	0 (0%)	8.6	17.64	3	3.7	4.7	0.7	67	13 (100%)	0 (0%)	4	17.56	-0.8	0.1	0.3	-4.9	62.2
	Follow-up day 45	13 (100%)	0 (0%)	5.3	3.44	3.2	4.5	6	0.5	14.4	13 (100%)	0 (0%)	0.7	2.77	-0.7	0.3	1.6	-3.9	8.2
NBMI300mg (n=11)	Visit day 1	11 (100%)	0 (0%)	4.5	1.53	3.9	4.9	5.2	0.7	6.7									
	Follow-up day 15	11 (100%)	0 (0%)	5.2	2.29	4	5.4	6.5	0.9	8.7	11 (100%)	0 (0%)	0.7	3.15	-1	0.2	1.7	-4.1	8
	Follow-up day 45	11 (100%)	0 (0%)	4.8	1.73	3.9	4.7	5.2	2.2	9.1	11 (100%)	0 (0%)	0.3	1.98	-1.4	0.2	1.3	-2	4.2
Placebo (n=12)	Visit day 1	12 (100%)	0 (0%)	4.5	1.85	3.65	4.25	5.85	0.7	7.3									
	Follow-up day 15	12 (100%)	0 (0%)	4.4	1.96	3.9	4.55	5.4	0.9	8.1	12 (100%)	0 (0%)	0	2.54	-1.1	0.5	1.45	-5.6	4
	Follow-up day 45	12 (100%)	0 (0%)	5.4	1.54	5	5.5	6.2	1.3	7.3	12 (100%)	0 (0%)	0.9	1.42	0.05	0.55	2.45	-1.5	2.7

Executed script: \\SDS-SERVER2\SAS$\SDS3\Projects\STATISTICS DRUG DEV\NBMI4011 - CTI002\Analyser\Script\SAS\T_cat001.sas, on: 23MAY2016:14:00, by: maria.lidsell
Version of source data: nbmi_neu.sas7bdat, last modified: 24FEB16:23:13, status: Final

Table DE5.15.1 Change from baseline of NMB, Sway test, Transversal Sway (Full analysis set)

		Result									Change from baseline (Visit day 1)								
**Treatment**	**Visit**	**n**	**nmiss**	**Mean**	**SD**	**Q1**	**Median**	**Q3**	**Min**	**Max**	**n**	**nmiss**	**Mean**	**SD**	**Q1**	**Median**	**Q3**	**Min**	**Max**
Total (n=36)	Visit day 1	36 (100%)	0 (0%)	2.4	0.96	1.8	2.45	3	0.3	4.3									
	Follow-up day 15	36 (100%)	0 (0%)	2.3	1.03	1.65	2.3	2.9	0.4	4.5	36 (100%)	0 (0%)	0	1.29	-0.6	-0.05	0.55	-3.4	2.6
	Follow-up day 45	36 (100%)	0 (0%)	2.8	1.65	1.9	2.2	3.4	0.3	9.5	36 (100%)	0 (0%)	0.4	1.5	-0.55	0.4	1	-2.1	6.4
NBMI100mg (n=13)	Visit day 1	13 (100%)	0 (0%)	2.4	0.85	1.6	2.4	2.8	1.1	4.3									
	Follow-up day 15	13 (100%)	0 (0%)	2.2	1.17	1.6	2	2.7	0.4	4.4	13 (100%)	0 (0%)	-0.1	1.34	-0.7	-0.1	0.2	-3.4	2.2
	Follow-up day 45	13 (100%)	0 (0%)	3.1	2.34	1.9	2.1	3.9	0.3	9.5	13 (100%)	0 (0%)	0.8	2.04	-0.4	0.4	0.6	-1.2	6.4
NBMI300mg (n=11)	Visit day 1	11 (100%)	0 (0%)	2.5	1.01	1.8	2.6	3.1	0.4	4									
	Follow-up day 15	11 (100%)	0 (0%)	2.5	0.93	1.7	2.8	3.1	0.6	3.7	11 (100%)	0 (0%)	0.1	1.26	-0.3	0.2	0.5	-2	2.6
	Follow-up day 45	11 (100%)	0 (0%)	2.4	1.15	1.8	2.2	3.1	1.2	5.2	11 (100%)	0 (0%)	0	1.35	-0.9	-0.3	0.6	-2.1	2.6
Placebo (n=12)	Visit day 1	12 (100%)	0 (0%)	2.3	1.09	1.8	2.1	3	0.3	4									
	Follow-up day 15	12 (100%)	0 (0%)	2.3	1.03	1.9	2.25	2.5	0.5	4.5	12 (100%)	0 (0%)	0	1.37	-0.6	-0.05	0.85	-3.1	2.2
	Follow-up day 45	12 (100%)	0 (0%)	2.7	1.09	2.15	2.7	3.4	0.7	4.5	12 (100%)	0 (0%)	0.4	0.77	-0.15	0.45	1.05	-0.9	1.4

Executed script: \\SDS-SERVER2\SAS$\SDS3\Projects\STATISTICS DRUG DEV\NBMI4011 - CTI002\Analyser\Script\SAS\T_cat001.sas, on: 23MAY2016:14:00, by: maria.lidsell
Version of source data: nbmi_neu.sas7bdat, last modified: 24FEB16:23:13, status: Final

Table DE5.16.1 Change from baseline of NMB, Sway test, Sagittal Sway (Full analysis set)

		Result									Change from baseline (Visit day 1)								
**Treatment**	**Visit**	**n**	**nmiss**	**Mean**	**SD**	**Q1**	**Median**	**Q3**	**Min**	**Max**	**n**	**nmiss**	**Mean**	**SD**	**Q1**	**Median**	**Q3**	**Min**	**Max**
Total (n=36)	Visit day 1	36 (100%)	0 (0%)	3.3	1.13	2.75	3.2	3.85	0.5	5.4									
	Follow-up day 15	36 (100%)	0 (0%)	3.3	1.71	2.35	3.2	4.4	0.5	7.5	36 (100%)	0 (0%)	0	2.12	-1.1	0.2	0.9	-4	7
	Follow-up day 45	36 (100%)	0 (0%)	3.7	1.71	2.5	3.6	4.4	0.3	9.4	36 (100%)	0 (0%)	0.4	1.69	-0.6	0.4	1.4	-3.6	4.6
NBMI100mg (n=13)	Visit day 1	13 (100%)	0 (0%)	3.3	0.99	2.5	3.1	3.7	2.1	5.4									
	Follow-up day 15	13 (100%)	0 (0%)	2.7	1.36	2	2.7	3.8	0.5	5	13 (100%)	0 (0%)	-0.6	1.31	-0.6	-0.4	0.2	-3.1	0.9
	Follow-up day 45	13 (100%)	0 (0%)	3.7	2.26	2.3	3.5	4.1	0.3	9.4	13 (100%)	0 (0%)	0.3	1.83	-0.2	0.4	1	-3.6	4.6
NBMI300mg (n=11)	Visit day 1	11 (100%)	0 (0%)	3.3	1.18	3.1	3.2	3.8	0.5	5.3									
	Follow-up day 15	11 (100%)	0 (0%)	4	2.05	2.4	4.1	4.9	0.6	7.5	11 (100%)	0 (0%)	0.7	2.9	-1.5	0.4	2.3	-3.2	7
	Follow-up day 45	11 (100%)	0 (0%)	3.6	1.31	2.8	3.6	4.2	1.4	6.4	11 (100%)	0 (0%)	0.4	1.66	-1.7	0.7	1.8	-1.9	2.8
Placebo (n=12)	Visit day 1	12 (100%)	0 (0%)	3.4	1.32	2.6	3.4	4.45	0.6	5.3									
	Follow-up day 15	12 (100%)	0 (0%)	3.3	1.62	2.8	3.4	4.1	0.6	5.9	12 (100%)	0 (0%)	0	1.96	-1	0.35	0.9	-4	2.9
	Follow-up day 45	12 (100%)	0 (0%)	3.8	1.45	2.95	4.15	4.65	0.9	6	12 (100%)	0 (0%)	0.5	1.72	-0.5	0.35	2.15	-2.7	2.8

Executed script: \\SDS-SERVER2\SAS$\SDS3\Projects\STATISTICS DRUG DEV\NBMI4011 - CTI002\Analyser\Script\SAS\T_cat001.sas, on: 23MAY2016:14:00, by: maria.lidsell
Version of source data: nbmi_neu.sas7bdat, last modified: 24FEB16:23:13, status: Final

Table DE5.17.1 Change from baseline of NMB, Sway test, Sway Area (Full analysis set)

		Result									Change from baseline (Visit day 1)								
**Treatment**	**Visit**	**n**	**nmiss**	**Mean**	**SD**	**Q1**	**Median**	**Q3**	**Min**	**Max**	**n**	**nmiss**	**Mean**	**SD**	**Q1**	**Median**	**Q3**	**Min**	**Max**
Total (n=36)	Visit day 1	36 (100%)	0 (0%)	187.8	126.56	91	167	235.5	3	677									
	Follow-up day 15	36 (100%)	0 (0%)	179.8	122.94	96	155.5	240	4	508	36 (100%)	0 (0%)	-8	126.33	-48	0	71.5	-414	207
	Follow-up day 45	36 (100%)	0 (0%)	215.4	132.72	124.5	196.5	283	2	521	36 (100%)	0 (0%)	27.6	121.75	-24	17	120.5	-419	285
NBMI100mg (n=13)	Visit day 1	13 (100%)	0 (0%)	181.6	105.76	89	161	226	79	433									
	Follow-up day 15	13 (100%)	0 (0%)	153	105.93	86	141	214	4	346	13 (100%)	0 (0%)	-28.6	124.98	-29	-5	22	-414	97
	Follow-up day 45	13 (100%)	0 (0%)	212.8	164.48	102	147	300	2	514	13 (100%)	0 (0%)	31.2	91.03	-24	17	68	-87	207
NBMI300mg (n=11)	Visit day 1	11 (100%)	0 (0%)	184.9	99.44	106	179	241	5	338									
	Follow-up day 15	11 (100%)	0 (0%)	221.5	134.96	95	212	383	9	418	11 (100%)	0 (0%)	36.6	131.69	-46	62	146	-226	207
	Follow-up day 45	11 (100%)	0 (0%)	211.7	136.82	132	152	256	56	521	11 (100%)	0 (0%)	26.8	125.91	-83	-22	127	-140	285
Placebo (n=12)	Visit day 1	12 (100%)	0 (0%)	197.2	172.12	85.5	166.5	225.5	3	677									
	Follow-up day 15	12 (100%)	0 (0%)	170.7	128.88	127.5	144.5	211.5	8	508	12 (100%)	0 (0%)	-26.5	122.93	-111.5	-2.5	61.5	-282	143
	Follow-up day 45	12 (100%)	0 (0%)	221.7	97.21	188.5	241.5	283	13	350	12 (100%)	0 (0%)	24.5	153.9	-3.5	50.5	126.5	-419	142

Executed script: \\SDS-SERVER2\SAS$\SDS3\Projects\STATISTICS DRUG DEV\NBMI4011 - CTI002\Analyser\Script\SAS\T_cat001.sas, on: 23MAY2016:14:00, by: maria.lidsell
Version of source data: nbmi_neu.sas7bdat, last modified: 24FEB16:23:13, status: Final

Table DE5.18.1 Change from baseline of NMB, Sway test, Sway Velocity (Full analysis set)

		Result									Change from baseline (Visit day 1)								
**Treatment**	**Visit**	**n**	**nmiss**	**Mean**	**SD**	**Q1**	**Median**	**Q3**	**Min**	**Max**	**n**	**nmiss**	**Mean**	**SD**	**Q1**	**Median**	**Q3**	**Min**	**Max**
Total (n=36)	Visit day 1	36 (100%)	0 (0%)	7.7	2.91	6.05	7.45	9.1	1	16.2									
	Follow-up day 15	36 (100%)	0 (0%)	7.3	2.77	5.85	7.65	9.4	0.9	12.2	36 (100%)	0 (0%)	-0.4	3.46	-0.6	0.3	1.35	-12.9	5.5
	Follow-up day 45	36 (100%)	0 (0%)	8.2	2.69	6.8	8.3	9.75	1.3	16	36 (100%)	0 (0%)	0.5	2	-0.35	0.55	1.75	-5.9	6.5
NBMI100mg (n=13)	Visit day 1	13 (100%)	0 (0%)	7.7	2.94	5.7	7	8.2	5.2	16.2									
	Follow-up day 15	13 (100%)	0 (0%)	6.8	2.6	5.9	6.8	9	0.9	9.9	13 (100%)	0 (0%)	-0.9	3.93	-0.4	0.1	0.9	-12.9	2.4
	Follow-up day 45	13 (100%)	0 (0%)	7.6	3.32	6.1	7.3	8.4	1.3	16	13 (100%)	0 (0%)	0	2.03	-0.5	0.6	1.2	-5.9	1.8
NBMI300mg (n=11)	Visit day 1	11 (100%)	0 (0%)	8	3.19	5.9	8.2	9.2	1	14									
	Follow-up day 15	11 (100%)	0 (0%)	8.1	2.83	6.4	8.8	10.3	1.9	12.2	11 (100%)	0 (0%)	0.1	3.01	-0.6	0.5	1.4	-7.1	5.5
	Follow-up day 45	11 (100%)	0 (0%)	8.7	1.87	7.5	8	10.8	6.1	11.7	11 (100%)	0 (0%)	0.7	2.45	-1.5	0.1	2.1	-2.3	6.5
Placebo (n=12)	Visit day 1	12 (100%)	0 (0%)	7.4	2.85	6.45	7.75	9.35	1	11.4									
	Follow-up day 15	12 (100%)	0 (0%)	7	2.93	5.1	7.45	9.55	1.9	10.4	12 (100%)	0 (0%)	-0.4	3.51	-1.85	0.45	1.85	-7.4	4.8
	Follow-up day 45	12 (100%)	0 (0%)	8.3	2.69	8	9.25	9.85	1.8	10.9	12 (100%)	0 (0%)	0.9	1.47	0.25	1.2	1.9	-2.1	2.8

Executed script: \\SDS-SERVER2\SAS$\SDS3\Projects\STATISTICS DRUG DEV\NBMI4011 - CTI002\Analyser\Script\SAS\T_cat001.sas, on: 23MAY2016:14:00, by: maria.lidsell
Version of source data: nbmi_neu.sas7bdat, last modified: 24FEB16:23:13, status: Final

Table DE5.19.1 Change from baseline of NMB, Sway test, Sway Intensity (Full analysis set)

		Result									Change from baseline (Visit day 1)								
**Treatment**	**Visit**	**n**	**nmiss**	**Mean**	**SD**	**Q1**	**Median**	**Q3**	**Min**	**Max**	**n**	**nmiss**	**Mean**	**SD**	**Q1**	**Median**	**Q3**	**Min**	**Max**
Total (n=36)	Visit day 1	36 (100%)	0 (0%)	3.7	1.26	3.05	3.45	4.35	0.7	7									
	Follow-up day 15	36 (100%)	0 (0%)	3.6	1.45	3.05	3.85	4.4	0.7	6.8	36 (100%)	0 (0%)	-0.1	1.76	-0.9	0.3	0.95	-5.5	3.2
	Follow-up day 45	36 (100%)	0 (0%)	4.9	5.49	3.25	4.3	4.95	0.5	36	36 (100%)	0 (0%)	1.2	5.32	-0.15	0.3	0.95	-2.4	31.6
NBMI100mg (n=13)	Visit day 1	13 (100%)	0 (0%)	3.8	1.15	3	3.5	4.2	2.6	6.7									
	Follow-up day 15	13 (100%)	0 (0%)	3.4	1.4	2.8	3.8	4.1	0.7	5.9	13 (100%)	0 (0%)	-0.3	1.83	-0.9	0.4	0.6	-5.5	1.4
	Follow-up day 45	13 (100%)	0 (0%)	3.9	1.54	3.2	3.6	5.1	0.5	6.3	13 (100%)	0 (0%)	0.1	1.16	-0.4	0	0.3	-2.1	2.5
NBMI300mg (n=11)	Visit day 1	11 (100%)	0 (0%)	3.8	1.29	3.3	4.2	4.6	0.7	5.6									
	Follow-up day 15	11 (100%)	0 (0%)	4	1.68	3.1	3.9	4.6	1.1	6.8	11 (100%)	0 (0%)	0.2	1.81	-0.9	0.1	2.2	-3.2	2.6
	Follow-up day 45	11 (100%)	0 (0%)	4.3	1.31	3.3	4.2	5	2.7	7.3	11 (100%)	0 (0%)	0.5	1.1	-0.1	0.2	0.8	-0.9	2.8
Placebo (n=12)	Visit day 1	12 (100%)	0 (0%)	3.5	1.44	2.9	3.35	3.85	0.7	7									
	Follow-up day 15	12 (100%)	0 (0%)	3.5	1.35	3.3	3.8	4.35	0.7	5.2	12 (100%)	0 (0%)	0	1.76	-1.25	0.45	1.05	-2.7	3.2
	Follow-up day 45	12 (100%)	0 (0%)	6.7	9.31	3.8	4.55	4.75	1.1	36	12 (100%)	0 (0%)	3.2	9.02	0.55	0.85	1.3	-2.4	31.6

Executed script: \\SDS-SERVER2\SAS$\SDS3\Projects\STATISTICS DRUG DEV\NBMI4011 - CTI002\Analyser\Script\SAS\T_cat001.sas, on: 23MAY2016:14:00, by: maria.lidsell
Version of source data: nbmi_neu.sas7bdat, last modified: 24FEB16:23:13, status: Final

Table DE5.20.1 Change from baseline of NMB, Rhythmic test slow P/S, Right hand, Average Time (Full analysis set)

		Result									Change from baseline (Visit day 1)								
**Treatment**	**Visit**	**n**	**nmiss**	**Mean**	**SD**	**Q1**	**Median**	**Q3**	**Min**	**Max**	**n**	**nmiss**	**Mean**	**SD**	**Q1**	**Median**	**Q3**	**Min**	**Max**
Total (n=36)	Visit day 1	36 (100%)	0 (0%)	-0.1	0.11	-0.224	-0.0885	-0.005	-0.25	0.034									
	Follow-up day 15	36 (100%)	0 (0%)	0	0.08	-0.063	-0.025	0.01	-0.244	0.109	36 (100%)	0 (0%)	0.1	0.14	-0.032	0.0635	0.2135	-0.258	0.328
	Follow-up day 45	36 (100%)	0 (0%)	0	0.11	-0.113	-0.037	0.0445	-0.25	0.155	36 (100%)	0 (0%)	0.1	0.14	-0.019	0.053	0.154	-0.26	0.291
NBMI100mg (n=13)	Visit day 1	13 (100%)	0 (0%)	-0.1	0.1	-0.179	-0.05	-0.008	-0.25	0.026									
	Follow-up day 15	13 (100%)	0 (0%)	-0.1	0.07	-0.087	-0.043	-0.032	-0.212	0.076	13 (100%)	0 (0%)	0	0.13	-0.056	-0.031	0.133	-0.13	0.269
	Follow-up day 45	13 (100%)	0 (0%)	0	0.12	-0.049	-0.002	0.083	-0.231	0.155	13 (100%)	0 (0%)	0.1	0.15	-0.014	0.09	0.168	-0.223	0.291
NBMI300mg (n=11)	Visit day 1	11 (100%)	0 (0%)	-0.1	0.12	-0.236	-0.142	0.003	-0.25	0.022									
	Follow-up day 15	11 (100%)	0 (0%)	0	0.07	-0.024	-0.009	0.028	-0.135	0.109	11 (100%)	0 (0%)	0.1	0.13	-0.017	0.059	0.251	-0.044	0.3
	Follow-up day 45	11 (100%)	0 (0%)	-0.1	0.1	-0.145	-0.089	0.042	-0.25	0.047	11 (100%)	0 (0%)	0	0.13	-0.108	0.034	0.117	-0.114	0.27
Placebo (n=12)	Visit day 1	12 (100%)	0 (0%)	-0.1	0.11	-0.2335	-0.129	-0.05	-0.25	0.034									
	Follow-up day 15	12 (100%)	0 (0%)	0	0.08	-0.0415	-0.0225	-0.0095	-0.244	0.078	12 (100%)	0 (0%)	0.1	0.17	0.036	0.112	0.2135	-0.258	0.328
	Follow-up day 45	12 (100%)	0 (0%)	-0.1	0.11	-0.1075	-0.035	0.0245	-0.247	0.074	12 (100%)	0 (0%)	0.1	0.15	-0.0055	0.0845	0.1665	-0.26	0.278

Executed script: \\SDS-SERVER2\SAS$\SDS3\Projects\STATISTICS DRUG DEV\NBMI4011 - CTI002\Analyser\Script\SAS\T_cat001.sas, on: 23MAY2016:14:00, by: maria.lidsell
Version of source data: nbmi_neu.sas7bdat, last modified: 24FEB16:23:13, status: Final

Table DE5.21.1 Change from baseline of NMB, Rhythmic test slow P/S, Right hand, Standard Deviation (Full analysis set)

		Result									Change from baseline (Visit day 1)								
**Treatment**	**Visit**	**n**	**nmiss**	**Mean**	**SD**	**Q1**	**Median**	**Q3**	**Min**	**Max**	**n**	**nmiss**	**Mean**	**SD**	**Q1**	**Median**	**Q3**	**Min**	**Max**
Total (n=36)	Visit day 1	36 (100%)	0 (0%)	0.1	0.04	0.033	0.0425	0.076	0	0.185									
	Follow-up day 15	36 (100%)	0 (0%)	0.1	0.04	0.039	0.0525	0.084	0.008	0.166	36 (100%)	0 (0%)	0	0.06	-0.031	0.0075	0.033	-0.165	0.143
	Follow-up day 45	36 (100%)	0 (0%)	0.1	0.05	0.039	0.059	0.0875	0	0.185	36 (100%)	0 (0%)	0	0.06	-0.008	0.0135	0.0555	-0.151	0.137
NBMI100mg (n=13)	Visit day 1	13 (100%)	0 (0%)	0.1	0.05	0.04	0.05	0.086	0	0.185									
	Follow-up day 15	13 (100%)	0 (0%)	0.1	0.05	0.038	0.042	0.099	0.02	0.166	13 (100%)	0 (0%)	0	0.07	-0.033	-0.001	0.037	-0.165	0.116
	Follow-up day 45	13 (100%)	0 (0%)	0.1	0.05	0.039	0.066	0.101	0.028	0.185	13 (100%)	0 (0%)	0	0.07	-0.025	0.024	0.061	-0.151	0.099
NBMI300mg (n=11)	Visit day 1	11 (100%)	0 (0%)	0.1	0.04	0.027	0.05	0.087	0	0.12									
	Follow-up day 15	11 (100%)	0 (0%)	0.1	0.04	0.042	0.063	0.078	0.027	0.162	11 (100%)	0 (0%)	0	0.06	-0.03	0.007	0.02	-0.076	0.143
	Follow-up day 45	11 (100%)	0 (0%)	0.1	0.03	0.039	0.055	0.088	0	0.123	11 (100%)	0 (0%)	0	0.04	-0.004	0.008	0.037	-0.071	0.07
Placebo (n=12)	Visit day 1	12 (100%)	0 (0%)	0	0.03	0.0195	0.0355	0.0535	0	0.105									
	Follow-up day 15	12 (100%)	0 (0%)	0.1	0.03	0.0335	0.0525	0.0775	0.008	0.103	12 (100%)	0 (0%)	0	0.04	-0.021	0.0225	0.037	-0.051	0.098
	Follow-up day 45	12 (100%)	0 (0%)	0.1	0.05	0.0375	0.054	0.0695	0.004	0.18	12 (100%)	0 (0%)	0	0.06	-0.003	0.0105	0.0565	-0.084	0.137

Executed script: \\SDS-SERVER2\SAS$\SDS3\Projects\STATISTICS DRUG DEV\NBMI4011 - CTI002\Analyser\Script\SAS\T_cat001.sas, on: 23MAY2016:14:00, by: maria.lidsell
Version of source data: nbmi_neu.sas7bdat, last modified: 24FEB16:23:13, status: Final

Table DE5.22.1 Change from baseline of NMB, Rhythmic test slow P/S, Right hand, P Index (Full analysis set)

		**Result**									**Change from baseline (Visit day 1)**								
**Treatment**	**Visit**	**n**	**nmiss**	**Mean**	**SD**	**Q1**	**Median**	**Q3**	**Min**	**Max**	**n**	**nmiss**	**Mean**	**SD**	**Q1**	**Median**	**Q3**	**Min**	**Max**
Total (n=36)	Visit day 1	36 (100%)	0 (0%)	118.3	56.18	69	144.5	157	3	183									
	Follow-up day 15	36 (100%)	0 (0%)	107.4	54.52	56	126	149.5	5	179	36 (100%)	0 (0%)	-10.9	82.5	-53.5	-16	38	-166	168
	Follow-up day 45	36 (100%)	0 (0%)	106.4	53.1	62	118.5	151.5	4	183	36 (100%)	0 (0%)	-11.9	71.65	-64.5	-15	20	-137	153
NBMI100mg (n=13)	Visit day 1	13 (100%)	0 (0%)	102.2	60.19	52	131	149	3	183									
	Follow-up day 15	13 (100%)	0 (0%)	106.1	61.79	36	145	151	5	171	13 (100%)	0 (0%)	3.9	92.13	-31	1	74	-137	168
	Follow-up day 45	13 (100%)	0 (0%)	94.6	53.58	53	91	150	13	164	13 (100%)	0 (0%)	-7.5	79.41	-58	-27	44	-115	153
NBMI300mg (n=11)	Visit day 1	11 (100%)	0 (0%)	116.2	58.29	50	132	164	20	183									
	Follow-up day 15	11 (100%)	0 (0%)	100.4	52.91	66	99	145	6	164	11 (100%)	0 (0%)	-15.8	85.11	-50	-13	40	-166	122
	Follow-up day 45	11 (100%)	0 (0%)	101.9	55.74	50	120	150	18	183	11 (100%)	0 (0%)	-14.3	68.11	-63	-13	6	-125	124
Placebo (n=12)	Visit day 1	12 (100%)	0 (0%)	137.8	47.7	121	154.5	170	30	183									
	Follow-up day 15	12 (100%)	0 (0%)	115.3	51.27	69.5	126	156.5	32	179	12 (100%)	0 (0%)	-22.5	73.34	-58	-28.5	21	-149	99
	Follow-up day 45	12 (100%)	0 (0%)	123.3	50.18	93	132.5	162.5	4	181	12 (100%)	0 (0%)	-14.4	72.14	-70.5	-13	16.5	-137	140

Executed script: \\SDS-SERVER2\SAS$\SDS3\Projects\STATISTICS DRUG DEV\NBMI4011 - CTI002\Analyser\Script\SAS\T_cat001.sas, on: 23MAY2016:14:00, by: maria.lidsell
Version of source data: nbmi_neu.sas7bdat, last modified: 24FEB16:23:13, status: Final

Table DE5.23.1 Change from baseline of NMB, Rhythmic test slow P/S, Left hand, Average Time (Full analysis set)

		Result									Change from baseline (Visit day 1)								
**Treatment**	**Visit**	**n**	**nmiss**	**Mean**	**SD**	**Q1**	**Median**	**Q3**	**Min**	**Max**	**n**	**nmiss**	**Mean**	**SD**	**Q1**	**Median**	**Q3**	**Min**	**Max**
Total (n=36)	Visit day 1	36 (100%)	0 (0%)	-0.1	0.09	-0.2055	-0.1275	-0.031	-0.25	0.019									
	Follow-up day 15	36 (100%)	0 (0%)	-0.1	0.08	-0.106	-0.0355	-0.003	-0.232	0.108	36 (100%)	0 (0%)	0.1	0.12	-0.0255	0.0415	0.1725	-0.227	0.358
	Follow-up day 45	36 (100%)	0 (0%)	-0.1	0.1	-0.092	-0.045	-0.005	-0.25	0.186	36 (100%)	0 (0%)	0.1	0.14	-0.0385	0.0555	0.174	-0.226	0.338
NBMI100mg (n=13)	Visit day 1	13 (100%)	0 (0%)	-0.1	0.07	-0.141	-0.071	-0.054	-0.236	-0.006									
	Follow-up day 15	13 (100%)	0 (0%)	0	0.09	-0.117	-0.02	0.018	-0.221	0.087	13 (100%)	0 (0%)	0.1	0.08	0.017	0.042	0.076	-0.064	0.228
	Follow-up day 45	13 (100%)	0 (0%)	0	0.1	-0.055	0.007	0.027	-0.25	0.186	13 (100%)	0 (0%)	0.1	0.12	0.036	0.078	0.203	-0.124	0.256
NBMI300mg (n=11)	Visit day 1	11 (100%)	0 (0%)	-0.1	0.12	-0.25	-0.037	-0.014	-0.25	0.019									
	Follow-up day 15	11 (100%)	0 (0%)	0	0.07	-0.075	-0.039	-0.013	-0.174	0.108	11 (100%)	0 (0%)	0.1	0.15	-0.038	-0.011	0.237	-0.056	0.358
	Follow-up day 45	11 (100%)	0 (0%)	-0.1	0.09	-0.146	-0.071	-0.031	-0.25	0.088	11 (100%)	0 (0%)	0	0.17	-0.102	-0.036	0.198	-0.226	0.338
Placebo (n=12)	Visit day 1	12 (100%)	0 (0%)	-0.1	0.08	-0.2055	-0.1375	-0.055	-0.228	-0.005									
	Follow-up day 15	12 (100%)	0 (0%)	-0.1	0.08	-0.139	-0.0365	-0.003	-0.232	0.006	12 (100%)	0 (0%)	0.1	0.14	-0.029	0.0915	0.1835	-0.227	0.211
	Follow-up day 45	12 (100%)	0 (0%)	-0.1	0.08	-0.111	-0.056	-0.0305	-0.247	0.019	12 (100%)	0 (0%)	0	0.12	-0.032	0.0305	0.1485	-0.219	0.231

Executed script: \\SDS-SERVER2\SAS$\SDS3\Projects\STATISTICS DRUG DEV\NBMI4011 - CTI002\Analyser\Script\SAS\T_cat001.sas, on: 23MAY2016:14:00, by: maria.lidsell
Version of source data: nbmi_neu.sas7bdat, last modified: 24FEB16:23:13, status: Final

Table DE5.24.1 Change from baseline of NMB, Rhythmic test slow P/S, Left hand, Standard Deviation (Full analysis set)

		Result									Change from baseline (Visit day 1)								
**Treatment**	**Visit**	**n**	**nmiss**	**Mean**	**SD**	**Q1**	**Median**	**Q3**	**Min**	**Max**	**n**	**nmiss**	**Mean**	**SD**	**Q1**	**Median**	**Q3**	**Min**	**Max**
Total (n=36)	Visit day 1	36 (100%)	0 (0%)	0.1	0.04	0.032	0.0545	0.0815	0	0.175									
	Follow-up day 15	36 (100%)	0 (0%)	0.1	0.04	0.033	0.0485	0.078	0.021	0.194	36 (100%)	0 (0%)	0	0.05	-0.0175	0	0.022	-0.134	0.099
	Follow-up day 45	36 (100%)	0 (0%)	0.1	0.04	0.0375	0.05	0.077	0	0.214	36 (100%)	0 (0%)	0	0.05	-0.0305	0.001	0.027	-0.124	0.17
NBMI100mg (n=13)	Visit day 1	13 (100%)	0 (0%)	0.1	0.05	0.054	0.069	0.124	0.029	0.175									
	Follow-up day 15	13 (100%)	0 (0%)	0.1	0.05	0.046	0.056	0.083	0.028	0.194	13 (100%)	0 (0%)	0	0.06	-0.026	0.001	0.02	-0.134	0.087
	Follow-up day 45	13 (100%)	0 (0%)	0.1	0.04	0.047	0.051	0.087	0	0.146	13 (100%)	0 (0%)	0	0.05	-0.049	-0.006	0.014	-0.124	0.055
NBMI300mg (n=11)	Visit day 1	11 (100%)	0 (0%)	0	0.02	0	0.032	0.052	0	0.07									
	Follow-up day 15	11 (100%)	0 (0%)	0.1	0.04	0.029	0.043	0.079	0.021	0.143	11 (100%)	0 (0%)	0	0.04	-0.021	0.009	0.06	-0.026	0.099
	Follow-up day 45	11 (100%)	0 (0%)	0.1	0.06	0.03	0.045	0.107	0	0.214	11 (100%)	0 (0%)	0	0.06	0.001	0.04	0.051	-0.052	0.17
Placebo (n=12)	Visit day 1	12 (100%)	0 (0%)	0.1	0.03	0.0365	0.0675	0.085	0.024	0.121									
	Follow-up day 15	12 (100%)	0 (0%)	0.1	0.03	0.033	0.05	0.0755	0.027	0.107	12 (100%)	0 (0%)	0	0.02	-0.0135	-0.003	0.0055	-0.053	0.024
	Follow-up day 45	12 (100%)	0 (0%)	0	0.02	0.0365	0.0465	0.066	0.006	0.091	12 (100%)	0 (0%)	0	0.03	-0.034	-0.012	0.0045	-0.079	0.045

Executed script: \\SDS-SERVER2\SAS$\SDS3\Projects\STATISTICS DRUG DEV\NBMI4011 - CTI002\Analyser\Script\SAS\T_cat001.sas, on: 23MAY2016:14:00, by: maria.lidsell
Version of source data: nbmi_neu.sas7bdat, last modified: 24FEB16:23:13, status: Final

Table DE5.25.1 Change from baseline of NMB, Rhythmic test slow P/S, Left hand, P Index (Full analysis set)

		**Result**									**Change from baseline (Visit day 1)**								
**Treatment**	**Visit**	**n**	**nmiss**	**Mean**	**SD**	**Q1**	**Median**	**Q3**	**Min**	**Max**	**n**	**nmiss**	**Mean**	**SD**	**Q1**	**Median**	**Q3**	**Min**	**Max**
Total (n=36)	Visit day 1	36 (100%)	0 (0%)	99.4	50.23	58.5	105.5	142	8	171									
	Follow-up day 15	36 (100%)	0 (0%)	100.5	44.82	62.5	116.5	140.5	5	154	36 (100%)	0 (0%)	1.2	51.75	-29.5	1.5	28	-113	121
	Follow-up day 45	36 (100%)	0 (0%)	101.8	46.4	65	113.5	134.5	3	171	36 (100%)	0 (0%)	2.5	58.07	-32	-2.5	41	-122	115
NBMI100mg (n=13)	Visit day 1	13 (100%)	0 (0%)	74.5	51.11	23	77	107	8	145									
	Follow-up day 15	13 (100%)	0 (0%)	89.5	47.75	56	102	122	5	147	13 (100%)	0 (0%)	15	54.9	-29	0	40	-40	121
	Follow-up day 45	13 (100%)	0 (0%)	95.8	45.66	53	111	120	14	171	13 (100%)	0 (0%)	21.3	59.87	-14	5	55	-89	115
NBMI300mg (n=11)	Visit day 1	11 (100%)	0 (0%)	136.1	31.79	111	142	171	76	171									
	Follow-up day 15	11 (100%)	0 (0%)	109.5	46.23	61	126	145	16	154	11 (100%)	0 (0%)	-26.6	56.39	-77	-11	30	-113	43
	Follow-up day 45	11 (100%)	0 (0%)	98.5	59.39	33	122	145	3	171	11 (100%)	0 (0%)	-37.6	49.16	-67	-40	-2	-122	60
Placebo (n=12)	Visit day 1	12 (100%)	0 (0%)	92.7	46.14	54	80.5	136	25	152									
	Follow-up day 15	12 (100%)	0 (0%)	104.3	41.5	67	113.5	140.5	34	148	12 (100%)	0 (0%)	11.7	34.96	-6.5	5.5	14.5	-44	87
	Follow-up day 45	12 (100%)	0 (0%)	111.5	34.82	83	119.5	136	48	167	12 (100%)	0 (0%)	18.8	47.97	-6.5	18	53.5	-76	102

Executed script: \\SDS-SERVER2\SAS$\SDS3\Projects\STATISTICS DRUG DEV\NBMI4011 - CTI002\Analyser\Script\SAS\T_cat001.sas, on: 23MAY2016:14:00, by: maria.lidsell
Version of source data: nbmi_neu.sas7bdat, last modified: 24FEB16:23:13, status: Final

Table DE5.26.1 Change from baseline of NMB, Rhythmic test fast P/S, Right hand, Average Time (Full analysis set)

		Result									Change from baseline (Visit day 1)								
**Treatment**	**Visit**	**n**	**nmiss**	**Mean**	**SD**	**Q1**	**Median**	**Q3**	**Min**	**Max**	**n**	**nmiss**	**Mean**	**SD**	**Q1**	**Median**	**Q3**	**Min**	**Max**
Total (n=36)	Visit day 1	36 (100%)	0 (0%)	-0.1	0.07	-0.1645	-0.094	-0.069	-0.245	0.033									
	Follow-up day 15	36 (100%)	0 (0%)	-0.1	0.06	-0.101	-0.029	-0.007	-0.192	0.037	36 (100%)	0 (0%)	0.1	0.08	0.001	0.0545	0.114	-0.133	0.206
	Follow-up day 45	36 (100%)	0 (0%)	-0.1	0.08	-0.168	-0.0725	-0.022	-0.25	0.011	36 (100%)	0 (0%)	0	0.1	-0.0385	0.0015	0.0985	-0.203	0.189
NBMI100mg (n=13)	Visit day 1	13 (100%)	0 (0%)	-0.1	0.06	-0.151	-0.097	-0.073	-0.207	0.012									
	Follow-up day 15	13 (100%)	0 (0%)	-0.1	0.08	-0.132	-0.023	-0.002	-0.189	0.037	13 (100%)	0 (0%)	0	0.08	0.006	0.033	0.098	-0.133	0.172
	Follow-up day 45	13 (100%)	0 (0%)	-0.1	0.09	-0.189	-0.051	-0.011	-0.25	0.011	13 (100%)	0 (0%)	0	0.11	-0.034	0.033	0.108	-0.171	0.156
NBMI300mg (n=11)	Visit day 1	11 (100%)	0 (0%)	-0.1	0.09	-0.19	-0.095	-0.045	-0.245	0.033									
	Follow-up day 15	11 (100%)	0 (0%)	0	0.05	-0.08	-0.031	-0.003	-0.157	0.021	11 (100%)	0 (0%)	0.1	0.08	-0.012	0.083	0.152	-0.042	0.191
	Follow-up day 45	11 (100%)	0 (0%)	-0.1	0.09	-0.183	-0.119	-0.027	-0.25	-0.004	11 (100%)	0 (0%)	0	0.1	-0.065	-0.019	0.089	-0.188	0.163
Placebo (n=12)	Visit day 1	12 (100%)	0 (0%)	-0.1	0.07	-0.161	-0.0915	-0.069	-0.228	-0.006									
	Follow-up day 15	12 (100%)	0 (0%)	-0.1	0.06	-0.1015	-0.029	-0.0115	-0.192	0.017	12 (100%)	0 (0%)	0.1	0.09	0.0025	0.049	0.1125	-0.102	0.206
	Follow-up day 45	12 (100%)	0 (0%)	-0.1	0.07	-0.1215	-0.0665	-0.0285	-0.222	-0.009	12 (100%)	0 (0%)	0	0.1	-0.024	0.032	0.0945	-0.203	0.189

Executed script: \\SDS-SERVER2\SAS$\SDS3\Projects\STATISTICS DRUG DEV\NBMI4011 - CTI002\Analyser\Script\SAS\T_cat001.sas, on: 23MAY2016:14:00, by: maria.lidsell
Version of source data: nbmi_neu.sas7bdat, last modified: 24FEB16:23:13, status: Final

Table DE5.27.1 Change from baseline of NMB, Rythmic test fast P/S, Right hand, Standard Deviation (Full analysis set)

		Result									Change from baseline (Visit day 1)								
**Treatment**	**Visit**	**n**	**nmiss**	**Mean**	**SD**	**Q1**	**Median**	**Q3**	**Min**	**Max**	**n**	**nmiss**	**Mean**	**SD**	**Q1**	**Median**	**Q3**	**Min**	**Max**
Total (n=36)	Visit day 1	36 (100%)	0 (0%)	0.1	0.04	0.0645	0.1095	0.13	0.014	0.144									
	Follow-up day 15	36 (100%)	0 (0%)	0.1	0.04	0.031	0.0725	0.1085	0.013	0.137	36 (100%)	0 (0%)	0	0.05	-0.0575	-0.0225	0.011	-0.105	0.081
	Follow-up day 45	36 (100%)	0 (0%)	0.1	0.04	0.0235	0.075	0.1085	0	0.142	36 (100%)	0 (0%)	0	0.05	-0.0685	-0.0225	0.0045	-0.124	0.081
NBMI100mg (n=13)	Visit day 1	13 (100%)	0 (0%)	0.1	0.04	0.05	0.11	0.131	0.026	0.144									
	Follow-up day 15	13 (100%)	0 (0%)	0.1	0.04	0.052	0.086	0.113	0.013	0.136	13 (100%)	0 (0%)	0	0.05	-0.036	-0.003	0.021	-0.105	0.081
	Follow-up day 45	13 (100%)	0 (0%)	0.1	0.04	0.025	0.08	0.096	0.002	0.121	13 (100%)	0 (0%)	0	0.06	-0.065	-0.024	0.013	-0.12	0.081
NBMI300mg (n=11)	Visit day 1	11 (100%)	0 (0%)	0.1	0.04	0.059	0.097	0.129	0.021	0.141									
	Follow-up day 15	11 (100%)	0 (0%)	0.1	0.04	0.024	0.068	0.106	0.015	0.137	11 (100%)	0 (0%)	0	0.05	-0.061	-0.023	0.032	-0.094	0.064
	Follow-up day 45	11 (100%)	0 (0%)	0.1	0.05	0.019	0.063	0.113	0	0.142	11 (100%)	0 (0%)	0	0.05	-0.072	-0.021	0.002	-0.097	0.054
Placebo (n=12)	Visit day 1	12 (100%)	0 (0%)	0.1	0.04	0.0735	0.11	0.1245	0.014	0.139									
	Follow-up day 15	12 (100%)	0 (0%)	0.1	0.04	0.025	0.047	0.096	0.015	0.137	12 (100%)	0 (0%)	0	0.04	-0.066	-0.023	0.002	-0.104	0.01
	Follow-up day 45	12 (100%)	0 (0%)	0.1	0.04	0.028	0.076	0.1115	0.01	0.132	12 (100%)	0 (0%)	0	0.05	-0.065	-0.015	0.004	-0.124	0.062

Executed script: \\SDS-SERVER2\SAS$\SDS3\Projects\STATISTICS DRUG DEV\NBMI4011 - CTI002\Analyser\Script\SAS\T_cat001.sas, on: 23MAY2016:14:00, by: maria.lidsell
Version of source data: nbmi_neu.sas7bdat, last modified: 24FEB16:23:13, status: Final

Table DE5.28.1 Change from baseline of NMB, Rhythmic test fast P/S, Right hand, P Index (Full analysis set)

		Result									Change from baseline (Visit day 1)								
**Treatment**	**Visit**	**n**	**nmiss**	**Mean**	**SD**	**Q1**	**Median**	**Q3**	**Min**	**Max**	**n**	**nmiss**	**Mean**	**SD**	**Q1**	**Median**	**Q3**	**Min**	**Max**
Total (n=36)	Visit day 1	36 (100%)	0 (0%)	30.2	50.91	1	3	25	1	158									
	Follow-up day 15	36 (100%)	0 (0%)	50.3	58.59	3	17	109	1	160	36 (100%)	0 (0%)	20.1	64.05	-2.5	2	34	-102	154
	Follow-up day 45	36 (100%)	0 (0%)	55.2	67.13	3	15	135	1	178	36 (100%)	0 (0%)	25	75.93	-0.5	7	58.5	-124	173
NBMI100mg (n=13)	Visit day 1	13 (100%)	0 (0%)	31.3	48.82	1	3	45	1	127									
	Follow-up day 15	13 (100%)	0 (0%)	33.7	50.83	3	9	42	1	160	13 (100%)	0 (0%)	2.4	67.1	-36	0	7	-102	154
	Follow-up day 45	13 (100%)	0 (0%)	52.4	67.34	6	12	131	2	175	13 (100%)	0 (0%)	21.1	85.2	-3	9	37	-124	173
NBMI300mg (n=11)	Visit day 1	11 (100%)	0 (0%)	31.8	55.18	1	5	31	1	143									
	Follow-up day 15	11 (100%)	0 (0%)	50.6	62.32	4	21	133	1	156	11 (100%)	0 (0%)	18.8	70.33	-10	3	41	-100	142
	Follow-up day 45	11 (100%)	0 (0%)	61.6	73.32	3	25	146	1	178	11 (100%)	0 (0%)	29.8	77.71	0	11	80	-108	173
Placebo (n=12)	Visit day 1	12 (100%)	0 (0%)	27.6	53.55	1.5	3.5	16	1	158									
	Follow-up day 15	12 (100%)	0 (0%)	68.1	62.48	6.5	60.5	129.5	1	156	12 (100%)	0 (0%)	40.5	53.09	0	10.5	93	-2	134
	Follow-up day 45	12 (100%)	0 (0%)	52.3	66.69	3	14.5	116.5	1	165	12 (100%)	0 (0%)	24.8	69.97	-0.5	5.5	58	-105	155

Executed script: \\SDS-SERVER2\SAS$\SDS3\Projects\STATISTICS DRUG DEV\NBMI4011 - CTI002\Analyser\Script\SAS\T_cat001.sas, on: 23MAY2016:14:00, by: maria.lidsell
Version of source data: nbmi_neu.sas7bdat, last modified: 24FEB16:23:13, status: Final

Table DE5.29.1 Change from baseline of NMB, Rhythmic test fast P/S, Left hand, Average Time (Full analysis set)

		Result									Change from baseline (Visit day 1)								
**Treatment**	**Visit**	**n**	**nmiss**	**Mean**	**SD**	**Q1**	**Median**	**Q3**	**Min**	**Max**	**n**	**nmiss**	**Mean**	**SD**	**Q1**	**Median**	**Q3**	**Min**	**Max**
Total (n=36)	Visit day 1	36 (100%)	0 (0%)	-0.1	0.07	-0.1355	-0.087	-0.03	-0.25	0.017									
	Follow-up day 15	36 (100%)	0 (0%)	-0.1	0.06	-0.1015	-0.034	-0.0075	-0.233	0.033	36 (100%)	0 (0%)	0	0.09	-0.018	0.0325	0.0975	-0.097	0.261
	Follow-up day 45	36 (100%)	0 (0%)	-0.1	0.08	-0.124	-0.0875	-0.0235	-0.25	0.014	36 (100%)	0 (0%)	0	0.11	-0.0545	-0.0055	0.0715	-0.229	0.23
NBMI100mg (n=13)	Visit day 1	13 (100%)	0 (0%)	-0.1	0.06	-0.135	-0.109	-0.081	-0.166	0.017									
	Follow-up day 15	13 (100%)	0 (0%)	-0.1	0.08	-0.104	-0.043	-0.026	-0.233	0.016	13 (100%)	0 (0%)	0	0.07	-0.014	0.026	0.083	-0.068	0.142
	Follow-up day 45	13 (100%)	0 (0%)	-0.1	0.08	-0.126	-0.079	-0.043	-0.25	0.002	13 (100%)	0 (0%)	0	0.08	-0.051	-0.011	0.06	-0.146	0.137
NBMI300mg (n=11)	Visit day 1	11 (100%)	0 (0%)	-0.1	0.09	-0.199	-0.088	-0.02	-0.244	0.009									
	Follow-up day 15	11 (100%)	0 (0%)	0	0.05	-0.101	-0.033	0.005	-0.105	0.033	11 (100%)	0 (0%)	0.1	0.09	0.002	0.064	0.128	-0.084	0.204
	Follow-up day 45	11 (100%)	0 (0%)	-0.1	0.09	-0.205	-0.094	-0.013	-0.25	0.014	11 (100%)	0 (0%)	0	0.14	-0.104	-0.007	0.137	-0.229	0.229
Placebo (n=12)	Visit day 1	12 (100%)	0 (0%)	-0.1	0.08	-0.107	-0.081	-0.03	-0.25	0.008									
	Follow-up day 15	12 (100%)	0 (0%)	-0.1	0.06	-0.0765	-0.034	-0.019	-0.2	0.012	12 (100%)	0 (0%)	0	0.1	-0.0305	0.0295	0.075	-0.097	0.261
	Follow-up day 45	12 (100%)	0 (0%)	-0.1	0.07	-0.119	-0.094	-0.018	-0.249	-0.013	12 (100%)	0 (0%)	0	0.11	-0.039	0.0015	0.0715	-0.216	0.23

Executed script: \\SDS-SERVER2\SAS$\SDS3\Projects\STATISTICS DRUG DEV\NBMI4011 - CTI002\Analyser\Script\SAS\T_cat001.sas, on: 23MAY2016:14:00, by: maria.lidsell
Version of source data: nbmi_neu.sas7bdat, last modified: 24FEB16:23:13, status: Final

Table DE5.30.1 Change from baseline of NMB, Rhythmic test fast P/S, Left hand, Standard Deviation (Full analysis set)

		**Result**									**Change from baseline (Visit day 1)**								
**Treatment**	**Visit**	**n**	**nmiss**	**Mean**	**SD**	**Q1**	**Median**	**Q3**	**Min**	**Max**	**n**	**nmiss**	**Mean**	**SD**	**Q1**	**Median**	**Q3**	**Min**	**Max**
Total (n=36)	Visit day 1	36 (100%)	0 (0%)	0.1	0.05	0.046	0.095	0.128	0	0.151									
	Follow-up day 15	36 (100%)	0 (0%)	0.1	0.04	0.025	0.064	0.108	0.012	0.136	36 (100%)	0 (0%)	0	0.06	-0.0665	-0.0035	0.0235	-0.129	0.1
	Follow-up day 45	36 (100%)	0 (0%)	0.1	0.05	0.021	0.059	0.112	0	0.155	36 (100%)	0 (0%)	0	0.05	-0.051	-0.0155	0.0025	-0.12	0.1
NBMI100mg (n=13)	Visit day 1	13 (100%)	0 (0%)	0.1	0.03	0.103	0.12	0.131	0.019	0.138									
	Follow-up day 15	13 (100%)	0 (0%)	0.1	0.04	0.03	0.066	0.11	0.017	0.135	13 (100%)	0 (0%)	0	0.04	-0.071	-0.036	-0.003	-0.108	0.018
	Follow-up day 45	13 (100%)	0 (0%)	0.1	0.04	0.031	0.084	0.11	0	0.133	13 (100%)	0 (0%)	0	0.05	-0.053	-0.028	-0.002	-0.12	0.057
NBMI300mg (n=11)	Visit day 1	11 (100%)	0 (0%)	0.1	0.04	0.025	0.077	0.095	0.021	0.121									
	Follow-up day 15	11 (100%)	0 (0%)	0.1	0.05	0.019	0.062	0.123	0.015	0.136	11 (100%)	0 (0%)	0	0.06	-0.062	0.009	0.032	-0.094	0.1
	Follow-up day 45	11 (100%)	0 (0%)	0.1	0.05	0.015	0.05	0.112	0	0.155	11 (100%)	0 (0%)	0	0.06	-0.07	-0.006	0.014	-0.095	0.078
Placebo (n=12)	Visit day 1	12 (100%)	0 (0%)	0.1	0.06	0.021	0.089	0.132	0	0.151									
	Follow-up day 15	12 (100%)	0 (0%)	0.1	0.04	0.0245	0.0595	0.0975	0.012	0.119	12 (100%)	0 (0%)	0	0.07	-0.07	0.0005	0.028	-0.129	0.07
	Follow-up day 45	12 (100%)	0 (0%)	0.1	0.04	0.025	0.056	0.113	0.003	0.127	12 (100%)	0 (0%)	0	0.05	-0.041	-0.0225	0.0155	-0.086	0.1

Executed script: \\SDS-SERVER2\SAS$\SDS3\Projects\STATISTICS DRUG DEV\NBMI4011 - CTI002\Analyser\Script\SAS\T_cat001.sas, on: 23MAY2016:14:00, by: maria.lidsell
Version of source data: nbmi_neu.sas7bdat, last modified: 24FEB16:23:13, status: Final

Table DE5.31.1 Change from baseline of NMB, Rhythmic test fast P/S, Left hand, P Index (Full analysis set)

		Result									Change from baseline (Visit day 1)								
**Treatment**	**Visit**	**n**	**nmiss**	**Mean**	**SD**	**Q1**	**Median**	**Q3**	**Min**	**Max**	**n**	**nmiss**	**Mean**	**SD**	**Q1**	**Median**	**Q3**	**Min**	**Max**
Total (n=36)	Visit day 1	36 (100%)	0 (0%)	47.9	56.28	7	18.5	84	4	167									
	Follow-up day 15	36 (100%)	0 (0%)	68.7	58.02	13	46.5	131.5	6	153	36 (100%)	0 (0%)	20.8	69.61	-10	3.5	82	-131	134
	Follow-up day 45	36 (100%)	0 (0%)	69.8	60.45	11	54	139.5	3	167	36 (100%)	0 (0%)	21.9	64.23	-0.5	8	52	-141	158
NBMI100mg (n=13)	Visit day 1	13 (100%)	0 (0%)	23.3	38.32	6	9	15	5	143									
	Follow-up day 15	13 (100%)	0 (0%)	65.9	57.26	12	44	121	6	147	13 (100%)	0 (0%)	42.6	48.43	1	32	86	-5	129
	Follow-up day 45	13 (100%)	0 (0%)	56.5	61.33	12	26	117	6	167	13 (100%)	0 (0%)	33.2	71.36	1	7	94	-110	158
NBMI300mg (n=11)	Visit day 1	11 (100%)	0 (0%)	57.3	53.1	18	31	132	9	140									
	Follow-up day 15	11 (100%)	0 (0%)	65.7	62.24	8	49	142	6	149	11 (100%)	0 (0%)	8.5	83.2	-45	-2	118	-131	127
	Follow-up day 45	11 (100%)	0 (0%)	84.4	61.91	11	69	149	3	167	11 (100%)	0 (0%)	27.1	64.45	-28	9	59	-43	149
Placebo (n=12)	Visit day 1	12 (100%)	0 (0%)	65.9	68.96	6	22.5	138	4	167									
	Follow-up day 15	12 (100%)	0 (0%)	74.5	59.66	17.5	59	133	9	153	12 (100%)	0 (0%)	8.6	75.43	-30	-1	50.5	-104	134
	Follow-up day 45	12 (100%)	0 (0%)	71	60.13	11	60.5	129.5	7	164	12 (100%)	0 (0%)	5.1	57.53	-9.5	12.5	47	-141	75

Executed script: \\SDS-SERVER2\SAS$\SDS3\Projects\STATISTICS DRUG DEV\NBMI4011 - CTI002\Analyser\Script\SAS\T_cat001.sas, on: 23MAY2016:14:00, by: maria.lidsell
Version of source data: nbmi_neu.sas7bdat, last modified: 24FEB16:23:13, status: Final

Table DE5.32.1 Change from baseline of NMB, Rhythmic test slow - Finger tapping, Right hand, Average Time (Full analysis set)

		**Result**									**Change from baseline (Visit day 1)**								
**Treatment**	**Visit**	**n**	**nmiss**	**Mean**	**SD**	**Q1**	**Median**	**Q3**	**Min**	**Max**	**n**	**nmiss**	**Mean**	**SD**	**Q1**	**Median**	**Q3**	**Min**	**Max**
Total (n=36)	Visit day 1	36 (100%)	0 (0%)	-0.1	0.09	-0.148	-0.061	-0.0155	-0.25	0.077									
	Follow-up day 15	36 (100%)	0 (0%)	-0.1	0.07	-0.0965	-0.058	-0.0125	-0.239	0.107	36 (100%)	0 (0%)	0	0.09	-0.026	0.008	0.0835	-0.166	0.207
	Follow-up day 45	36 (100%)	0 (0%)	-0.1	0.1	-0.098	-0.0335	-0.0025	-0.25	0.177	36 (100%)	0 (0%)	0	0.13	-0.05	0.0285	0.1115	-0.281	0.265
NBMI100mg (n=13)	Visit day 1	13 (100%)	0 (0%)	-0.1	0.07	-0.156	-0.087	-0.048	-0.23	-0.007									
	Follow-up day 15	13 (100%)	0 (0%)	-0.1	0.08	-0.145	-0.068	-0.029	-0.239	0.016	13 (100%)	0 (0%)	0	0.09	-0.022	0.006	0.054	-0.155	0.207
	Follow-up day 45	13 (100%)	0 (0%)	0	0.13	-0.1	-0.023	0.015	-0.25	0.177	13 (100%)	0 (0%)	0.1	0.15	0.031	0.044	0.179	-0.243	0.265
NBMI300mg (n=11)	Visit day 1	11 (100%)	0 (0%)	-0.1	0.1	-0.14	-0.028	0.014	-0.25	0.035									
	Follow-up day 15	11 (100%)	0 (0%)	0	0.07	-0.059	-0.017	0	-0.157	0.107	11 (100%)	0 (0%)	0	0.09	-0.043	0.048	0.14	-0.091	0.191
	Follow-up day 45	11 (100%)	0 (0%)	-0.1	0.11	-0.212	-0.031	-0.003	-0.25	0.064	11 (100%)	0 (0%)	0	0.14	-0.066	0.008	0.07	-0.281	0.204
Placebo (n=12)	Visit day 1	12 (100%)	0 (0%)	-0.1	0.1	-0.154	-0.041	0.0065	-0.202	0.077									
	Follow-up day 15	12 (100%)	0 (0%)	-0.1	0.06	-0.097	-0.072	-0.0135	-0.112	0.073	12 (100%)	0 (0%)	0	0.1	-0.026	0.008	0.0835	-0.166	0.18
	Follow-up day 45	12 (100%)	0 (0%)	0	0.03	-0.0635	-0.0375	-0.0195	-0.096	0.011	12 (100%)	0 (0%)	0	0.09	-0.0545	0.0195	0.0965	-0.123	0.163

Executed script: \\SDS-SERVER2\SAS$\SDS3\Projects\STATISTICS DRUG DEV\NBMI4011 - CTI002\Analyser\Script\SAS\T_cat001.sas, on: 23MAY2016:14:00, by: maria.lidsell
Version of source data: nbmi_neu.sas7bdat, last modified: 24FEB16:23:13, status: Final

Table DE5.33.1 Change from baseline of NMB, Rhythmic test slow - Finger tapping, Right hand, Standard Deviation (Full analysis set)

		Result									Change from baseline (Visit day 1)								
**Treatment**	**Visit**	**n**	**nmiss**	**Mean**	**SD**	**Q1**	**Median**	**Q3**	**Min**	**Max**	**n**	**nmiss**	**Mean**	**SD**	**Q1**	**Median**	**Q3**	**Min**	**Max**
Total (n=36)	Visit day 1	36 (100%)	0 (0%)	0.1	0.05	0.0315	0.059	0.094	0	0.196									
	Follow-up day 15	36 (100%)	0 (0%)	0.1	0.04	0.038	0.044	0.085	0.025	0.193	36 (100%)	0 (0%)	0	0.04	-0.02	0.0005	0.022	-0.155	0.059
	Follow-up day 45	36 (100%)	0 (0%)	0.1	0.04	0.0435	0.058	0.0815	0	0.139	36 (100%)	0 (0%)	0	0.06	-0.0295	-0.0015	0.025	-0.168	0.112
NBMI100mg (n=13)	Visit day 1	13 (100%)	0 (0%)	0.1	0.06	0.05	0.076	0.151	0.031	0.196									
	Follow-up day 15	13 (100%)	0 (0%)	0.1	0.05	0.04	0.044	0.094	0.025	0.193	13 (100%)	0 (0%)	0	0.06	-0.036	-0.017	-0.001	-0.155	0.042
	Follow-up day 45	13 (100%)	0 (0%)	0.1	0.04	0.037	0.048	0.075	0	0.126	13 (100%)	0 (0%)	0	0.06	-0.076	-0.025	0.002	-0.168	0.026
NBMI300mg (n=11)	Visit day 1	11 (100%)	0 (0%)	0.1	0.05	0.02	0.04	0.086	0	0.131									
	Follow-up day 15	11 (100%)	0 (0%)	0.1	0.04	0.035	0.041	0.054	0.028	0.163	11 (100%)	0 (0%)	0	0.03	-0.021	0.005	0.032	-0.07	0.041
	Follow-up day 45	11 (100%)	0 (0%)	0.1	0.03	0.047	0.061	0.081	0	0.128	11 (100%)	0 (0%)	0	0.05	-0.006	0	0.061	-0.086	0.079
Placebo (n=12)	Visit day 1	12 (100%)	0 (0%)	0.1	0.03	0.0305	0.0585	0.0725	0.026	0.095									
	Follow-up day 15	12 (100%)	0 (0%)	0.1	0.03	0.042	0.064	0.094	0.028	0.121	12 (100%)	0 (0%)	0	0.03	-0.0005	0.0095	0.033	-0.032	0.059
	Follow-up day 45	12 (100%)	0 (0%)	0.1	0.04	0.0485	0.066	0.0955	0.034	0.139	12 (100%)	0 (0%)	0	0.05	-0.0165	0.008	0.0525	-0.061	0.112

Executed script: \\SDS-SERVER2\SAS$\SDS3\Projects\STATISTICS DRUG DEV\NBMI4011 - CTI002\Analyser\Script\SAS\T_cat001.sas, on: 23MAY2016:14:00, by: maria.lidsell
Version of source data: nbmi_neu.sas7bdat, last modified: 24FEB16:23:13, status: Final

Table DE5.34.1 Change from baseline of NMB, Rhythmic test slow - Finger tapping, Right hand, P Index (Full analysis set)

		Result									Change from baseline (Visit day 1)								
**Treatment**	**Visit**	**n**	**nmiss**	**Mean**	**SD**	**Q1**	**Median**	**Q3**	**Min**	**Max**	**n**	**nmiss**	**Mean**	**SD**	**Q1**	**Median**	**Q3**	**Min**	**Max**
Total (n=36)	Visit day 1	36 (100%)	0 (0%)	86.1	55.66	35.5	85.5	142	3	173									
	Follow-up day 15	36 (100%)	0 (0%)	93	48.19	45.5	119.5	130	3	150	36 (100%)	0 (0%)	6.9	47.46	-20	-1.5	35.5	-95	123
	Follow-up day 45	36 (100%)	0 (0%)	86.8	48	49.5	88	122	12	173	36 (100%)	0 (0%)	0.7	78.16	-47	0.5	31.5	-137	167
NBMI100mg (n=13)	Visit day 1	13 (100%)	0 (0%)	61.4	53.21	9	57	108	3	143									
	Follow-up day 15	13 (100%)	0 (0%)	92	52.97	36	120	127	3	150	13 (100%)	0 (0%)	30.6	48.03	0	21	42	-33	123
	Follow-up day 45	13 (100%)	0 (0%)	100	52.26	58	113	132	16	173	13 (100%)	0 (0%)	38.6	70.37	-7	11	98	-53	167
NBMI300mg (n=11)	Visit day 1	11 (100%)	0 (0%)	103.9	63.1	44	127	156	14	173									
	Follow-up day 15	11 (100%)	0 (0%)	108.8	43.51	99	126	137	6	147	11 (100%)	0 (0%)	4.9	40.4	-22	-6	45	-47	83
	Follow-up day 45	11 (100%)	0 (0%)	86.2	45.7	50	81	113	15	173	11 (100%)	0 (0%)	-17.7	80.46	-98	0	11	-120	129
Placebo (n=12)	Visit day 1	12 (100%)	0 (0%)	96.4	44.67	62	87	143.5	35	150									
	Follow-up day 15	12 (100%)	0 (0%)	79.6	46.46	36.5	76.5	125	18	147	12 (100%)	0 (0%)	-16.8	43.41	-44	-15.5	1.5	-95	64
	Follow-up day 45	12 (100%)	0 (0%)	73	45.14	37	72.5	110	12	138	12 (100%)	0 (0%)	-23.4	74.01	-82.5	-14	27.5	-137	103

Executed script: \\SDS-SERVER2\SAS$\SDS3\Projects\STATISTICS DRUG DEV\NBMI4011 - CTI002\Analyser\Script\SAS\T_cat001.sas, on: 23MAY2016:14:00, by: maria.lidsell
Version of source data: nbmi_neu.sas7bdat, last modified: 24FEB16:23:13, status: Final

Table DE5.35.1 Change from baseline of NMB, Rhythmic test slow - Finger tapping, Left hand, Average Time (Full analysis set)

		Result									Change from baseline (Visit day 1)								
**Treatment**	**Visit**	**n**	**nmiss**	**Mean**	**SD**	**Q1**	**Median**	**Q3**	**Min**	**Max**	**n**	**nmiss**	**Mean**	**SD**	**Q1**	**Median**	**Q3**	**Min**	**Max**
Total (n=36)	Visit day 1	36 (100%)	0 (0%)	-1.3	7.15	-0.1845	-0.0645	-0.013	-43	0.068									
	Follow-up day 15	36 (100%)	0 (0%)	-1.2	6.99	-0.1165	-0.0565	-0.0135	-42	0.04	36 (100%)	0 (0%)	0.1	10.15	-0.0205	0.0345	0.0795	-41.991	42.914
	Follow-up day 45	36 (100%)	0 (0%)	-0.1	0.1	-0.1415	-0.0375	-0.0065	-0.25	0.191	36 (100%)	0 (0%)	1.2	7.13	-0.015	0.017	0.1015	-0.218	42.775
NBMI100mg (n=13)	Visit day 1	13 (100%)	0 (0%)	-3.4	11.9	-0.195	-0.09	-0.035	-43	0.012									
	Follow-up day 15	13 (100%)	0 (0%)	-0.1	0.08	-0.086	-0.063	-0.022	-0.248	0.022	13 (100%)	0 (0%)	3.3	11.89	-0.028	0.045	0.088	-0.075	42.914
	Follow-up day 45	13 (100%)	0 (0%)	-0.1	0.1	-0.151	-0.046	-0.006	-0.225	0.135	13 (100%)	0 (0%)	3.3	11.85	-0.007	0.053	0.189	-0.114	42.775
NBMI300mg (n=11)	Visit day 1	11 (100%)	0 (0%)	-0.1	0.11	-0.219	-0.046	0.008	-0.25	0.024									
	Follow-up day 15	11 (100%)	0 (0%)	0	0.06	-0.019	-0.01	0.001	-0.148	0.04	11 (100%)	0 (0%)	0.1	0.09	-0.018	0.051	0.107	-0.027	0.242
	Follow-up day 45	11 (100%)	0 (0%)	-0.1	0.13	-0.21	-0.116	-0.007	-0.25	0.191	11 (100%)	0 (0%)	0	0.17	-0.177	0.006	0.087	-0.218	0.373
Placebo (n=12)	Visit day 1	12 (100%)	0 (0%)	-0.1	0.08	-0.128	-0.0445	-0.011	-0.206	0.068									
	Follow-up day 15	12 (100%)	0 (0%)	-3.6	12.1	-0.128	-0.098	-0.041	-42	0.027	12 (100%)	0 (0%)	-3.5	12.12	-0.071	0.0015	0.042	-41.991	0.112
	Follow-up day 45	12 (100%)	0 (0%)	0	0.06	-0.065	-0.025	-0.0005	-0.215	0.017	12 (100%)	0 (0%)	0	0.09	0.0045	0.0205	0.078	-0.165	0.175

Executed script: \\SDS-SERVER2\SAS$\SDS3\Projects\STATISTICS DRUG DEV\NBMI4011 - CTI002\Analyser\Script\SAS\T_cat001.sas, on: 23MAY2016:14:00, by: maria.lidsell
Version of source data: nbmi_neu.sas7bdat, last modified: 24FEB16:23:13, status: Final

Table DE5.36.1 Change from baseline of NMB, Rhythmic test slow - Finger tapping, Left hand, Standard Deviation (Full analysis set)

		Result									Change from baseline (Visit day 1)								
Treatment	Visit	n	nmiss	Mean	SD	Q1	Median	Q3	Min	Max	n	nmiss	Mean	SD	Q1	Median	Q3	Min	Max
Total (n=36)	Visit day 1	36 (100%)	0 (0%)	0.1	0.04	0.039	0.0505	0.0905	0	0.165									
	Follow-up day 15	36 (100%)	0 (0%)	0.1	0.04	0.037	0.054	0.085	0.005	0.163	36 (100%)	0 (0%)	0	0.06	-0.0155	-0.0035	0.023	-0.126	0.103
	Follow-up day 45	36 (100%)	0 (0%)	0.1	0.03	0.0345	0.063	0.0885	0	0.142	36 (100%)	0 (0%)	0	0.05	-0.0335	0.001	0.034	-0.124	0.087
NBMI100mg (n=13)	Visit day 1	13 (100%)	0 (0%)	0.1	0.05	0.042	0.059	0.131	0.019	0.165									
	Follow-up day 15	13 (100%)	0 (0%)	0.1	0.05	0.044	0.05	0.086	0.005	0.153	13 (100%)	0 (0%)	0	0.07	-0.065	0.001	0.019	-0.126	0.103
	Follow-up day 45	13 (100%)	0 (0%)	0.1	0.03	0.037	0.07	0.104	0.035	0.142	13 (100%)	0 (0%)	0	0.06	-0.043	-0.004	0.025	-0.124	0.086
NBMI300mg (n=11)	Visit day 1	11 (100%)	0 (0%)	0	0.04	0.021	0.038	0.082	0	0.111									
	Follow-up day 15	11 (100%)	0 (0%)	0.1	0.03	0.035	0.068	0.091	0.022	0.126	11 (100%)	0 (0%)	0	0.06	-0.014	-0.001	0.084	-0.084	0.095
	Follow-up day 45	11 (100%)	0 (0%)	0	0.03	0.025	0.046	0.078	0	0.091	11 (100%)	0 (0%)	0	0.04	-0.025	0	0.053	-0.082	0.06
Placebo (n=12)	Visit day 1	12 (100%)	0 (0%)	0.1	0.03	0.0425	0.0545	0.073	0.03	0.143									
	Follow-up day 15	12 (100%)	0 (0%)	0.1	0.04	0.037	0.054	0.0655	0.023	0.163	12 (100%)	0 (0%)	0	0.04	-0.022	-0.007	0.0005	-0.053	0.099
	Follow-up day 45	12 (100%)	0 (0%)	0.1	0.03	0.033	0.0635	0.089	0.026	0.133	12 (100%)	0 (0%)	0	0.05	-0.0345	0.013	0.034	-0.098	0.087

Executed script: \\SDS-SERVER2\SAS$\SDS3\Projects\STATISTICS DRUG DEV\NBMI4011 - CTI002\Analyser\Script\SAS\T_cat001.sas, on: 23MAY2016:14:00, by: maria.lidsell
Version of source data: nbmi_neu.sas7bdat, last modified: 24FEB16:23:13, status: Final

Table DE5.37.1 Change from baseline of NMB, Rhythmic test slow - Finger tapping, Left hand, P Index (Full analysis set)

		Result									Change from baseline (Visit day 1)								
**Treatment**	**Visit**	**n**	**nmiss**	**Mean**	**SD**	**Q1**	**Median**	**Q3**	**Min**	**Max**	**n**	**nmiss**	**Mean**	**SD**	**Q1**	**Median**	**Q3**	**Min**	**Max**
Total (n=36)	Visit day 1	36 (100%)	0 (0%)	82	63.22	14	86	130	0	188									
	Follow-up day 15	36 (100%)	0 (0%)	79.8	59.05	18	71.5	135.5	1	185	36 (100%)	0 (0%)	-2.3	80.18	-30	10.5	32.5	-176	183
	Follow-up day 45	36 (100%)	0 (0%)	80.6	65.87	17.5	67	145	1	188	36 (100%)	0 (0%)	-1.4	89	-70.5	-2	55	-165	168
NBMI100mg (n=13)	Visit day 1	13 (100%)	0 (0%)	66.2	65.4	2	57	121	0	172									
	Follow-up day 15	13 (100%)	0 (0%)	76.8	59.38	17	89	114	1	185	13 (100%)	0 (0%)	10.7	81.88	-16	-1	35	-105	183
	Follow-up day 45	13 (100%)	0 (0%)	68.7	63.39	7	37	138	1	155	13 (100%)	0 (0%)	2.5	96.1	-65	5	21	-165	155
NBMI300mg (n=11)	Visit day 1	11 (100%)	0 (0%)	108.6	70.26	20	133	169	6	188									
	Follow-up day 15	11 (100%)	0 (0%)	75.3	65.99	13	38	143	3	168	11 (100%)	0 (0%)	-33.4	104.93	-148	0	18	-176	154
	Follow-up day 45	11 (100%)	0 (0%)	101.6	70.96	25	106	164	14	188	11 (100%)	0 (0%)	-7	94.51	-113	0	56	-138	168
Placebo (n=12)	Visit day 1	12 (100%)	0 (0%)	74.8	49.99	32.5	70.5	118.5	1	153									
	Follow-up day 15	12 (100%)	0 (0%)	87	56.58	45	71.5	137	1	166	12 (100%)	0 (0%)	12.3	41.69	-0.5	13.5	40.5	-79	63
	Follow-up day 45	12 (100%)	0 (0%)	74.3	64.74	15.5	49.5	147	2	162	12 (100%)	0 (0%)	-0.5	83.42	-72	-19.5	72	-103	142

Executed script: \\SDS-SERVER2\SAS$\SDS3\Projects\STATISTICS DRUG DEV\NBMI4011 - CTI002\Analyser\Script\SAS\T_cat001.sas, on: 23MAY2016:14:00, by: maria.lidsell
Version of source data: nbmi_neu.sas7bdat, last modified: 24FEB16:23:13, status: Final

Table DE5.38.1 Change from baseline of NMB, Rhythmic test fast - Finger tapping, Right hand, Average Time (Full analysis set)

		Result									Change from baseline (Visit day 1)								
**Treatment**	**Visit**	**n**	**nmiss**	**Mean**	**SD**	**Q1**	**Median**	**Q3**	**Min**	**Max**	**n**	**nmiss**	**Mean**	**SD**	**Q1**	**Median**	**Q3**	**Min**	**Max**
Total (n=36)	Visit day 1	36 (100%)	0 (0%)	-0.1	0.08	-0.15	-0.0865	-0.0295	-0.249	0.046									
	Follow-up day 15	36 (100%)	0 (0%)	-0.1	0.07	-0.1315	-0.0925	-0.033	-0.243	0.012	36 (100%)	0 (0%)	0	0.09	-0.0555	0	0.041	-0.241	0.154
	Follow-up day 45	36 (100%)	0 (0%)	-0.1	0.08	-0.158	-0.066	-0.036	-0.25	0.015	36 (100%)	0 (0%)	0	0.1	-0.0795	-0.0015	0.076	-0.239	0.179
NBMI100mg (n=13)	Visit day 1	13 (100%)	0 (0%)	-0.1	0.08	-0.157	-0.101	-0.047	-0.242	0.03									
	Follow-up day 15	13 (100%)	0 (0%)	-0.1	0.08	-0.169	-0.091	-0.036	-0.241	-0.005	13 (100%)	0 (0%)	0	0.09	-0.035	0.013	0.05	-0.241	0.121
	Follow-up day 45	13 (100%)	0 (0%)	-0.1	0.08	-0.164	-0.089	-0.052	-0.237	0.004	13 (100%)	0 (0%)	0	0.1	-0.08	-0.012	0.074	-0.158	0.179
NBMI300mg (n=11)	Visit day 1	11 (100%)	0 (0%)	-0.1	0.09	-0.182	-0.058	-0.011	-0.249	0.011									
	Follow-up day 15	11 (100%)	0 (0%)	-0.1	0.08	-0.114	-0.096	-0.013	-0.243	0.012	11 (100%)	0 (0%)	0	0.09	-0.056	0.016	0.068	-0.156	0.154
	Follow-up day 45	11 (100%)	0 (0%)	-0.1	0.09	-0.218	-0.093	-0.029	-0.25	-0.006	11 (100%)	0 (0%)	0	0.12	-0.087	0.009	0.072	-0.239	0.137
Placebo (n=12)	Visit day 1	12 (100%)	0 (0%)	-0.1	0.07	-0.123	-0.087	-0.027	-0.208	0.046									
	Follow-up day 15	12 (100%)	0 (0%)	-0.1	0.04	-0.1315	-0.102	-0.0735	-0.172	-0.028	12 (100%)	0 (0%)	0	0.08	-0.0705	-0.0055	0.01	-0.191	0.117
	Follow-up day 45	12 (100%)	0 (0%)	-0.1	0.06	-0.0785	-0.0495	-0.036	-0.192	0.015	12 (100%)	0 (0%)	0	0.1	-0.0515	0.005	0.102	-0.166	0.157

Executed script: \\SDS-SERVER2\SAS$\SDS3\Projects\STATISTICS DRUG DEV\NBMI4011 - CTI002\Analyser\Script\SAS\T_cat001.sas, on: 23MAY2016:14:00, by: maria.lidsell
Version of source data: nbmi_neu.sas7bdat, last modified: 24FEB16:23:13, status: Final

Table DE5.39.1 Change from baseline of NMB, Rhythmic test fast - Finger tapping, Right hand, Standard Deviation (Full analysis set)

		Result									Change from baseline (Visit day 1)								
**Treatment**	**Visit**	**n**	**nmiss**	**Mean**	**SD**	**Q1**	**Median**	**Q3**	**Min**	**Max**	**n**	**nmiss**	**Mean**	**SD**	**Q1**	**Median**	**Q3**	**Min**	**Max**
Total (n=36)	Visit day 1	36 (100%)	0 (0%)	0.1	0.03	0.02	0.0475	0.083	0.002	0.113									
	Follow-up day 15	36 (100%)	0 (0%)	0.1	0.04	0.022	0.0455	0.0955	0.01	0.146	36 (100%)	0 (0%)	0	0.05	-0.024	-0.001	0.035	-0.097	0.115
	Follow-up day 45	36 (100%)	0 (0%)	0	0.03	0.018	0.0405	0.0795	0	0.116	36 (100%)	0 (0%)	0	0.04	-0.0365	-0.0005	0.0285	-0.079	0.085
NBMI100mg (n=13)	Visit day 1	13 (100%)	0 (0%)	0.1	0.03	0.04	0.069	0.09	0.015	0.113									
	Follow-up day 15	13 (100%)	0 (0%)	0	0.04	0.025	0.04	0.055	0.011	0.123	13 (100%)	0 (0%)	0	0.04	-0.048	-0.004	0.012	-0.077	0.033
	Follow-up day 45	13 (100%)	0 (0%)	0.1	0.04	0.022	0.058	0.092	0.012	0.116	13 (100%)	0 (0%)	0	0.05	-0.041	0	0.025	-0.079	0.076
NBMI300mg (n=11)	Visit day 1	11 (100%)	0 (0%)	0	0.04	0.018	0.039	0.082	0.002	0.111									
	Follow-up day 15	11 (100%)	0 (0%)	0.1	0.05	0.014	0.026	0.101	0.012	0.146	11 (100%)	0 (0%)	0	0.05	-0.031	-0.003	0.052	-0.097	0.099
	Follow-up day 45	11 (100%)	0 (0%)	0	0.04	0.013	0.026	0.074	0	0.104	11 (100%)	0 (0%)	0	0.04	-0.03	-0.008	0.02	-0.06	0.072
Placebo (n=12)	Visit day 1	12 (100%)	0 (0%)	0	0.03	0.02	0.033	0.071	0.013	0.097									
	Follow-up day 15	12 (100%)	0 (0%)	0.1	0.04	0.0305	0.0805	0.0995	0.01	0.135	12 (100%)	0 (0%)	0	0.04	-0.002	0.021	0.0505	-0.028	0.115
	Follow-up day 45	12 (100%)	0 (0%)	0	0.03	0.018	0.03	0.0715	0.013	0.105	12 (100%)	0 (0%)	0	0.05	-0.0365	-0.001	0.038	-0.077	0.085

Executed script: \\SDS-SERVER2\SAS$\SDS3\Projects\STATISTICS DRUG DEV\NBMI4011 - CTI002\Analyser\Script\SAS\T_cat001.sas, on: 23MAY2016:14:00, by: maria.lidsell
Version of source data: nbmi_neu.sas7bdat, last modified: 24FEB16:23:13, status: Final

Table DE5.40.1 Change from baseline of NMB, Rythmic test fast - Finger tapping, Right hand, P Index (Full analysis set)

		Result									Change from baseline (Visit day 1)								
**Treatment**	**Visit**	**n**	**nmiss**	**Mean**	**SD**	**Q1**	**Median**	**Q3**	**Min**	**Max**	**n**	**nmiss**	**Mean**	**SD**	**Q1**	**Median**	**Q3**	**Min**	**Max**
Total (n=36)	Visit day 1	36 (100%)	0 (0%)	49	57.68	2	18.5	111.5	0	174									
	Follow-up day 15	36 (100%)	0 (0%)	53.8	59.36	1	22.5	100	0	154	36 (100%)	0 (0%)	4.8	63.49	-5.5	1.5	28.5	-173	143
	Follow-up day 45	36 (100%)	0 (0%)	60	62.7	2	29	124	0	177	36 (100%)	0 (0%)	11.1	77.8	-25	0.5	64.5	-171	167
NBMI100mg (n=13)	Visit day 1	13 (100%)	0 (0%)	30.5	48.36	1	4	31	0	138									
	Follow-up day 15	13 (100%)	0 (0%)	54.9	58.47	11	31	83	0	152	13 (100%)	0 (0%)	24.4	50.08	-1	9	38	-58	143
	Follow-up day 45	13 (100%)	0 (0%)	44.6	58.95	1	9	100	0	148	13 (100%)	0 (0%)	14.1	59.51	-15	-1	25	-71	147
NBMI300mg (n=11)	Visit day 1	11 (100%)	0 (0%)	54.6	65.27	2	33	122	0	174									
	Follow-up day 15	11 (100%)	0 (0%)	71.6	61.1	1	75	143	0	149	11 (100%)	0 (0%)	17	79.04	-5	18	65	-173	143
	Follow-up day 45	11 (100%)	0 (0%)	73.1	71.09	3	75	144	0	177	11 (100%)	0 (0%)	18.5	97.46	-47	1	111	-171	167
Placebo (n=12)	Visit day 1	12 (100%)	0 (0%)	63.8	59.06	4.5	52.5	113.5	1	147									
	Follow-up day 15	12 (100%)	0 (0%)	36.3	58.6	1	2.5	60	0	154	12 (100%)	0 (0%)	-27.5	51.68	-68	-1	2.5	-114	25
	Follow-up day 45	12 (100%)	0 (0%)	64.8	60.38	4	64.5	122	0	146	12 (100%)	0 (0%)	1	81.15	-67	3	65	-113	119

Executed script: \\SDS-SERVER2\SAS$\SDS3\Projects\STATISTICS DRUG DEV\NBMI4011 - CTI002\Analyser\Script\SAS\T_cat001.sas, on: 23MAY2016:14:00, by: maria.lidsell
Version of source data: nbmi_neu.sas7bdat, last modified: 24FEB16:23:13, status: Final

Table DE5.41.1 Change from baseline of NMB, Rhythmic test fast - Finger tapping, Left hand, Average Time (Full analysis set)

		Result									Change from baseline (Visit day 1)								
**Treatment**	**Visit**	**n**	**nmiss**	**Mean**	**SD**	**Q1**	**Median**	**Q3**	**Min**	**Max**	**n**	**nmiss**	**Mean**	**SD**	**Q1**	**Median**	**Q3**	**Min**	**Max**
Total (n=36)	Visit day 1	36 (100%)	0 (0%)	-0.1	0.08	-0.145	-0.0835	-0.0355	-0.25	0.007									
	Follow-up day 15	36 (100%)	0 (0%)	-0.1	0.08	-0.1355	-0.055	-0.0275	-0.245	0.015	36 (100%)	0 (0%)	0	0.08	-0.0225	0.0085	0.065	-0.196	0.162
	Follow-up day 45	36 (100%)	0 (0%)	-0.8	4.32	-0.1335	-0.062	-0.0315	-26	-0.009	36 (100%)	0 (0%)	-0.7	4.34	-0.036	0.0235	0.0835	-26.005	0.15
NBMI100mg (n=13)	Visit day 1	13 (100%)	0 (0%)	-0.1	0.08	-0.156	-0.094	-0.044	-0.25	0.007									
	Follow-up day 15	13 (100%)	0 (0%)	-0.1	0.09	-0.194	-0.074	-0.026	-0.245	0.009	13 (100%)	0 (0%)	0	0.08	-0.002	0.018	0.063	-0.196	0.12
	Follow-up day 45	13 (100%)	0 (0%)	-0.1	0.08	-0.14	-0.064	-0.029	-0.245	-0.009	13 (100%)	0 (0%)	0	0.11	-0.016	0.032	0.102	-0.187	0.148
NBMI300mg (n=11)	Visit day 1	11 (100%)	0 (0%)	-0.1	0.09	-0.161	-0.058	-0.01	-0.248	0.005									
	Follow-up day 15	11 (100%)	0 (0%)	-0.1	0.08	-0.128	-0.074	-0.029	-0.245	0.015	11 (100%)	0 (0%)	0	0.08	-0.025	0.01	0.067	-0.103	0.148
	Follow-up day 45	11 (100%)	0 (0%)	-2.5	7.81	-0.204	-0.132	-0.055	-26	-0.018	11 (100%)	0 (0%)	-2.4	7.84	-0.146	-0.019	0.038	-26.005	0.15
Placebo (n=12)	Visit day 1	12 (100%)	0 (0%)	-0.1	0.06	-0.107	-0.0745	-0.0315	-0.215	-0.02									
	Follow-up day 15	12 (100%)	0 (0%)	-0.1	0.07	-0.094	-0.052	-0.028	-0.218	-0.003	12 (100%)	0 (0%)	0	0.08	-0.0525	0.003	0.0625	-0.113	0.162
	Follow-up day 45	12 (100%)	0 (0%)	-0.1	0.03	-0.0625	-0.048	-0.03	-0.131	-0.02	12 (100%)	0 (0%)	0	0.06	-0.0095	0.029	0.0575	-0.098	0.123

Executed script: \\SDS-SERVER2\SAS$\SDS3\Projects\STATISTICS DRUG DEV\NBMI4011 - CTI002\Analyser\Script\SAS\T_cat001.sas, on: 23MAY2016:14:00, by: maria.lidsell
Version of source data: nbmi_neu.sas7bdat, last modified: 24FEB16:23:13, status: Final

Table DE5.42.1 Change from baseline of NMB, Rhythmic test fast - Finger tapping, Left hand, Standard Deviation (Full analysis set)

		**Result**									**Change from baseline (Visit day 1)**								
**Treatment**	**Visit**	**n**	**nmiss**	**Mean**	**SD**	**Q1**	**Median**	**Q3**	**Min**	**Max**	**n**	**nmiss**	**Mean**	**SD**	**Q1**	**Median**	**Q3**	**Min**	**Max**
Total (n=36)	Visit day 1	36 (100%)	0 (0%)	0.1	0.04	0.024	0.0585	0.0825	0	0.13									
	Follow-up day 15	36 (100%)	0 (0%)	0.1	0.04	0.0145	0.054	0.082	0.011	0.126	36 (100%)	0 (0%)	0	0.03	-0.014	-0.0025	0.0045	-0.101	0.063
	Follow-up day 45	36 (100%)	0 (0%)	0.1	0.04	0.021	0.0445	0.0845	0.011	0.136	36 (100%)	0 (0%)	0	0.05	-0.0465	-0.001	0.0315	-0.107	0.086
NBMI100mg (n=13)	Visit day 1	13 (100%)	0 (0%)	0.1	0.04	0.027	0.069	0.08	0	0.127									
	Follow-up day 15	13 (100%)	0 (0%)	0.1	0.04	0.016	0.056	0.09	0.012	0.115	13 (100%)	0 (0%)	0	0.04	-0.014	-0.001	0.016	-0.068	0.054
	Follow-up day 45	13 (100%)	0 (0%)	0.1	0.04	0.026	0.048	0.082	0.013	0.115	13 (100%)	0 (0%)	0	0.06	-0.065	-0.003	0.041	-0.101	0.082
NBMI300mg (n=11)	Visit day 1	11 (100%)	0 (0%)	0.1	0.04	0.015	0.053	0.082	0.008	0.13									
	Follow-up day 15	11 (100%)	0 (0%)	0.1	0.04	0.015	0.052	0.084	0.012	0.126	11 (100%)	0 (0%)	0	0.03	-0.014	-0.003	0.006	-0.046	0.063
	Follow-up day 45	11 (100%)	0 (0%)	0.1	0.04	0.03	0.078	0.098	0.011	0.136	11 (100%)	0 (0%)	0	0.04	-0.013	0.006	0.045	-0.042	0.086
Placebo (n=12)	Visit day 1	12 (100%)	0 (0%)	0.1	0.04	0.024	0.0495	0.087	0.01	0.13									
	Follow-up day 15	12 (100%)	0 (0%)	0	0.03	0.013	0.044	0.075	0.011	0.087	12 (100%)	0 (0%)	0	0.04	-0.0135	-0.003	-0.001	-0.101	0.057
	Follow-up day 45	12 (100%)	0 (0%)	0	0.03	0.016	0.0255	0.053	0.013	0.105	12 (100%)	0 (0%)	0	0.05	-0.051	-0.009	0.005	-0.107	0.061

Executed script: \\SDS-SERVER2\SAS$\SDS3\Projects\STATISTICS DRUG DEV\NBMI4011 - CTI002\Analyser\Script\SAS\T_cat001.sas, on: 23MAY2016:14:00, by: maria.lidsell
Version of source data: nbmi_neu.sas7bdat, last modified: 24FEB16:23:13, status: Final

Table DE5.43.1 Change from baseline of NMB, Rhythmic test fast - Finger tapping, Left hand, P Index (Full analysis set)

		Result									Change from baseline (Visit day 1)								
**Treatment**	**Visit**	**n**	**nmiss**	**Mean**	**SD**	**Q1**	**Median**	**Q3**	**Min**	**Max**	**n**	**nmiss**	**Mean**	**SD**	**Q1**	**Median**	**Q3**	**Min**	**Max**
Total (n=36)	Visit day 1	36 (100%)	0 (0%)	59.5	56.95	12	30	111	2	165									
	Follow-up day 15	36 (100%)	0 (0%)	68.4	60.04	12	36	137.5	2	145	36 (100%)	0 (0%)	8.9	54.73	-5.5	2	20.5	-133	137
	Follow-up day 45	36 (100%)	0 (0%)	65.2	54.15	11	51.5	120.5	2	146	36 (100%)	0 (0%)	5.7	77.72	-37	0	74	-153	127
NBMI100mg (n=13)	Visit day 1	13 (100%)	0 (0%)	49.8	55.8	13	20	100	2	165									
	Follow-up day 15	13 (100%)	0 (0%)	64.2	62.62	9	33	135	3	143	13 (100%)	0 (0%)	14.4	55.56	-15	1	22	-54	130
	Follow-up day 45	13 (100%)	0 (0%)	61.8	52	12	45	103	3	141	13 (100%)	0 (0%)	12	90.79	-38	0	88	-153	127
NBMI300mg (n=11)	Visit day 1	11 (100%)	0 (0%)	67.6	61.98	12	37	138	2	151									
	Follow-up day 15	11 (100%)	0 (0%)	67.1	60.86	11	39	136	2	143	11 (100%)	0 (0%)	-0.5	62.11	-15	2	19	-133	93
	Follow-up day 45	11 (100%)	0 (0%)	43.1	52.35	7	14	90	2	146	11 (100%)	0 (0%)	-24.5	75.68	-81	-3	8	-144	88
Placebo (n=12)	Visit day 1	12 (100%)	0 (0%)	62.5	56.95	10.5	49.5	110	2	148									
	Follow-up day 15	12 (100%)	0 (0%)	74.1	61.37	16	65.5	141.5	10	145	12 (100%)	0 (0%)	11.6	50.25	1	6	18.5	-92	137
	Follow-up day 45	12 (100%)	0 (0%)	89.2	52.54	40.5	106	134	5	143	12 (100%)	0 (0%)	26.7	59.8	-9.5	23	74	-93	114

Executed script: \\SDS-SERVER2\SAS$\SDS3\Projects\STATISTICS DRUG DEV\NBMI4011 - CTI002\Analyser\Script\SAS\T_cat001.sas, on: 23MAY2016:14:00, by: maria.lidsell
Version of source data: nbmi_neu.sas7bdat, last modified: 24FEB16:23:13, status: Final

Table DE5.44.1 Change from baseline of NMB, Max. Frequency test P/S, Right hand, Max Frequency (Full analysis set)

		Result									Change from baseline (Visit day 1)								
**Treatment**	**Visit**	**n**	**nmiss**	**Mean**	**SD**	**Q1**	**Median**	**Q3**	**Min**	**Max**	**n**	**nmiss**	**Mean**	**SD**	**Q1**	**Median**	**Q3**	**Min**	**Max**
Total (n=36)	Visit day 1	36 (100%)	0 (0%)	5.7	1.31	4.9	5.5	7.1	3.3	7.5									
	Follow-up day 15	36 (100%)	0 (0%)	6.1	1.36	5	6.55	7.5	2.9	7.5	36 (100%)	0 (0%)	0.4	1.46	-0.45	0.05	1.1	-2.8	4.2
	Follow-up day 45	36 (100%)	0 (0%)	6.4	1.16	5.45	6.65	7.5	3.8	7.5	36 (100%)	0 (0%)	0.7	1.57	-0.3	0.3	1.5	-2	4.2
NBMI100mg (n=13)	Visit day 1	13 (100%)	0 (0%)	5.5	1.24	4.7	5.2	6.3	3.3	7.5									
	Follow-up day 15	13 (100%)	0 (0%)	5.7	1.54	4.6	5.7	7	2.9	7.5	13 (100%)	0 (0%)	0.2	1.34	-0.5	0	1.1	-2.1	2.7
	Follow-up day 45	13 (100%)	0 (0%)	6.2	1.08	5.4	6	7.5	4.4	7.5	13 (100%)	0 (0%)	0.8	1.55	0	0.6	1.5	-2	4.2
NBMI300mg (n=11)	Visit day 1	11 (100%)	0 (0%)	6.2	1.47	5.3	6.7	7.5	3.3	7.5									
	Follow-up day 15	11 (100%)	0 (0%)	6.4	1.27	4.8	6.7	7.5	4.5	7.5	11 (100%)	0 (0%)	0.2	1.78	-0.8	0	0.8	-2.8	4.2
	Follow-up day 45	11 (100%)	0 (0%)	7	0.79	6	7.5	7.5	5.5	7.5	11 (100%)	0 (0%)	0.8	1.72	0	0.4	2.1	-2	4.2
Placebo (n=12)	Visit day 1	12 (100%)	0 (0%)	5.6	1.21	5	5.35	6.2	3.6	7.5									
	Follow-up day 15	12 (100%)	0 (0%)	6.3	1.23	5.4	6.55	7.5	3.8	7.5	12 (100%)	0 (0%)	0.7	1.32	0.1	0.6	1.2	-2.1	3.3
	Follow-up day 45	12 (100%)	0 (0%)	6.1	1.39	5	6.05	7.5	3.8	7.5	12 (100%)	0 (0%)	0.5	1.58	-0.8	0	1.85	-1.2	3.6

Executed script: \\SDS-SERVER2\SAS$\SDS3\Projects\STATISTICS DRUG DEV\NBMI4011 - CTI002\Analyser\Script\SAS\T_cat001.sas, on: 23MAY2016:14:00, by: maria.lidsell
Version of source data: nbmi_neu.sas7bdat, last modified: 24FEB16:23:13, status: Final

Table DE5.45.1 Change from baseline of NMB, Max. Frequency test P/S, Right hand, MF Index (Full analysis set)

		Result									Change from baseline (Visit day 1)								
**Treatment**	**Visit**	**n**	**nmiss**	**Mean**	**SD**	**Q1**	**Median**	**Q3**	**Min**	**Max**	**n**	**nmiss**	**Mean**	**SD**	**Q1**	**Median**	**Q3**	**Min**	**Max**
Total (n=36)	Visit day 1	36 (100%)	0 (0%)	135.4	54.76	103.5	145	187.5	23	192									
	Follow-up day 15	36 (100%)	0 (0%)	147.2	55.72	111	179.5	192	14	192	36 (100%)	0 (0%)	11.8	66.84	-10	3	43.5	-130	169
	Follow-up day 45	36 (100%)	0 (0%)	161.1	43.36	142.5	181.5	192	34	192	36 (100%)	0 (0%)	25.7	66.95	-8	11.5	64	-115	169
NBMI100mg (n=13)	Visit day 1	13 (100%)	0 (0%)	125	55.88	85	128	175	23	192									
	Follow-up day 15	13 (100%)	0 (0%)	131.8	66.2	76	153	187	14	192	13 (100%)	0 (0%)	6.8	63.96	-30	0	36	-127	102
	Follow-up day 45	13 (100%)	0 (0%)	157.7	40.9	140	166	192	62	192	13 (100%)	0 (0%)	32.7	73.06	0	14	97	-115	169
NBMI300mg (n=11)	Visit day 1	11 (100%)	0 (0%)	151	56.47	134	183	192	23	192									
	Follow-up day 15	11 (100%)	0 (0%)	155.5	49.49	94	183	192	69	192	11 (100%)	0 (0%)	4.5	76.45	-51	0	9	-107	169
	Follow-up day 45	11 (100%)	0 (0%)	182.2	16.3	166	192	192	145	192	11 (100%)	0 (0%)	31.2	61.44	0	9	52	-47	169
Placebo (n=12)	Visit day 1	12 (100%)	0 (0%)	132.3	53.41	113	136.5	171.5	30	192									
	Follow-up day 15	12 (100%)	0 (0%)	156.3	49.31	138.5	179.5	192	34	192	12 (100%)	0 (0%)	24	64.76	6	15	53	-130	156
	Follow-up day 45	12 (100%)	0 (0%)	145.4	56.48	109.5	165	192	34	192	12 (100%)	0 (0%)	13.2	68.89	-33.5	0	64	-83	153

Executed script: \\SDS-SERVER2\SAS$\SDS3\Projects\STATISTICS DRUG DEV\NBMI4011 - CTI002\Analyser\Script\SAS\T_cat001.sas, on: 23MAY2016:14:00, by: maria.lidsell
Version of source data: nbmi_neu.sas7bdat, last modified: 24FEB16:23:13, status: Final

Table DE5.46.1 Change from baseline of NMB, Max. Frequency test P/S, Left hand, Max Frequency (Full analysis set)

		Result									Change from baseline (Visit day 1)								
**Treatment**	**Visit**	**n**	**nmiss**	**Mean**	**SD**	**Q1**	**Median**	**Q3**	**Min**	**Max**	**n**	**nmiss**	**Mean**	**SD**	**Q1**	**Median**	**Q3**	**Min**	**Max**
Total (n=36)	Visit day 1	36 (100%)	0 (0%)	6.2	6.47	3.8	5.1	6.45	3.2	43									
	Follow-up day 15	36 (100%)	0 (0%)	6	1.46	4.95	6	7.5	3.3	7.5	36 (100%)	0 (0%)	-0.3	6.93	-0.2	0.7	1.85	-39.4	4.3
	Follow-up day 45	35 (97.22%)	1 (2.78%)	6	1.22	5.1	5.8	7.5	3.3	7.5	35 (97.22%)	1 (2.78%)	-0.3	6.65	-0.1	0.6	1.6	-37.5	4.3
NBMI100mg (n=13)	Visit day 1	13 (100%)	0 (0%)	7.5	10.73	3.6	4.8	5.1	3.2	43									
	Follow-up day 15	13 (100%)	0 (0%)	5.6	1.43	4.3	5.4	7.2	3.6	7.5	13 (100%)	0 (0%)	-1.8	11.36	0.2	0.9	1.8	-39.4	4.3
	Follow-up day 45	12 (92.31%)	1 (7.69%)	5.5	1.02	4.75	5.15	6	4.4	7.5	12 (92.31%)	1 (7.69%)	-2.3	11.19	-0.35	0.2	1.4	-37.5	4.3
NBMI300mg (n=11)	Visit day 1	11 (100%)	0 (0%)	5.9	1.43	4.9	5.7	7.5	3.3	7.5									
	Follow-up day 15	11 (100%)	0 (0%)	5.7	1.68	3.9	5.2	7.5	3.3	7.5	11 (100%)	0 (0%)	-0.2	1.63	-1.3	-0.4	1.7	-2.5	2.4
	Follow-up day 45	11 (100%)	0 (0%)	6.7	0.97	5.6	7.5	7.5	5.1	7.5	11 (100%)	0 (0%)	0.8	1.36	0	0.6	1.8	-1.9	3
Placebo (n=12)	Visit day 1	12 (100%)	0 (0%)	5.2	1.65	3.7	4.6	6.9	3.5	7.5									
	Follow-up day 15	12 (100%)	0 (0%)	6.6	1.14	5.6	7.4	7.5	4.5	7.5	12 (100%)	0 (0%)	1.4	1.96	0.15	0.9	3.3	-2.3	4
	Follow-up day 45	12 (100%)	0 (0%)	6	1.39	5	6	7.5	3.3	7.5	12 (100%)	0 (0%)	0.8	1.73	-0.1	0.75	1.8	-2.4	4

Executed script: \\SDS-SERVER2\SAS$\SDS3\Projects\STATISTICS DRUG DEV\NBMI4011 - CTI002\Analyser\Script\SAS\T_cat001.sas, on: 23MAY2016:14:00, by: maria.lidsell
Version of source data: nbmi_neu.sas7bdat, last modified: 24FEB16:23:13, status: Final

Table DE5.47.1 Change from baseline of NMB, Max. Frequency test P/S, Left hand, MF Index (Full analysis set)

		Result									Change from baseline (Visit day 1)								
**Treatment**	**Visit**	**n**	**nmiss**	**Mean**	**SD**	**Q1**	**Median**	**Q3**	**Min**	**Max**	**n**	**nmiss**	**Mean**	**SD**	**Q1**	**Median**	**Q3**	**Min**	**Max**
Total (n=36)	Visit day 1	36 (100%)	0 (0%)	116.4	66.61	40	132	182.5	22	195									
	Follow-up day 15	36 (100%)	0 (0%)	148.6	57.33	129	176	195	26	195	36 (100%)	0 (0%)	32.2	67.76	-5	22	67	-85	173
	Follow-up day 45	36 (100%)	0 (0%)	154.1	48.75	132	167.5	195	4	195	36 (100%)	0 (0%)	37.7	65.64	-1.5	12.5	86	-100	173
NBMI100mg (n=13)	Visit day 1	13 (100%)	0 (0%)	91.5	59.7	35	86	139	22	180									
	Follow-up day 15	13 (100%)	0 (0%)	135.5	60.18	68	155	193	35	195	13 (100%)	0 (0%)	44.1	58.03	15	34	54	-33	173
	Follow-up day 45	13 (100%)	0 (0%)	129.9	53.16	107	139	159	4	195	13 (100%)	0 (0%)	38.5	80.18	-18	13	91	-100	173
NBMI300mg (n=11)	Visit day 1	11 (100%)	0 (0%)	151.9	54.66	125	166	195	26	195									
	Follow-up day 15	11 (100%)	0 (0%)	136	68.33	46	145	195	26	195	11 (100%)	0 (0%)	-15.9	44.39	-50	-2	29	-85	56
	Follow-up day 45	11 (100%)	0 (0%)	181.5	18.97	163	195	195	139	195	11 (100%)	0 (0%)	29.5	47.39	0	12	56	-32	113
Placebo (n=12)	Visit day 1	12 (100%)	0 (0%)	111	74.06	37.5	96.5	190	30	195									
	Follow-up day 15	12 (100%)	0 (0%)	174.4	34.3	160.5	194.5	195	86	195	12 (100%)	0 (0%)	63.4	74.83	1	62	133.5	-50	165
	Follow-up day 45	12 (100%)	0 (0%)	155.3	52.06	132	176	195	26	195	12 (100%)	0 (0%)	44.3	67.38	-6	26	84.5	-56	165

Executed script: \\SDS-SERVER2\SAS$\SDS3\Projects\STATISTICS DRUG DEV\NBMI4011 - CTI002\Analyser\Script\SAS\T_cat001.sas, on: 23MAY2016:14:00, by: maria.lidsell
Version of source data: nbmi_neu.sas7bdat, last modified: 24FEB16:23:13, status: Final

Table DE5.48.1 Change from baseline of NMB, Max. Frequency test - Finger tapping, Right hand, Max Frequency (Full analysis set)

		Result									Change from baseline (Visit day 1)								
**Treatment**	**Visit**	**n**	**nmiss**	**Mean**	**SD**	**Q1**	**Median**	**Q3**	**Min**	**Max**	**n**	**nmiss**	**Mean**	**SD**	**Q1**	**Median**	**Q3**	**Min**	**Max**
Total (n=36)	Visit day 1	36 (100%)	0 (0%)	7.8	0.96	8.1	8.1	8.1	3.3	8.1									
	Follow-up day 15	36 (100%)	0 (0%)	8	1.93	8.1	8.1	8.1	5.3	18.1	36 (100%)	0 (0%)	0.2	2.09	0	0	0	-2.8	10
	Follow-up day 45	35 (97.22%)	1 (2.78%)	8	0.58	8.1	8.1	8.1	5	8.1	35 (97.22%)	1 (2.78%)	0.1	1	0	0	0	-3.1	4.8
NBMI100mg (n=13)	Visit day 1	13 (100%)	0 (0%)	7.9	0.86	8.1	8.1	8.1	5	8.1									
	Follow-up day 15	13 (100%)	0 (0%)	7.7	0.88	8.1	8.1	8.1	5.3	8.1	13 (100%)	0 (0%)	-0.2	1.32	0	0	0	-2.8	3.1
	Follow-up day 45	12 (92.31%)	1 (7.69%)	7.8	0.89	8.1	8.1	8.1	5	8.1	12 (92.31%)	1 (7.69%)	-0.3	0.89	0	0	0	-3.1	0
NBMI300mg (n=11)	Visit day 1	11 (100%)	0 (0%)	8	0.36	8.1	8.1	8.1	6.9	8.1									
	Follow-up day 15	11 (100%)	0 (0%)	7.9	0.75	8.1	8.1	8.1	5.6	8.1	11 (100%)	0 (0%)	-0.1	0.39	0	0	0	-1.3	0
	Follow-up day 45	11 (100%)	0 (0%)	8.1	0.03	8.1	8.1	8.1	8	8.1	11 (100%)	0 (0%)	0.1	0.33	0	0	0	0	1.1
Placebo (n=12)	Visit day 1	12 (100%)	0 (0%)	7.6	1.4	8.1	8.1	8.1	3.3	8.1									
	Follow-up day 15	12 (100%)	0 (0%)	8.4	3.18	7.35	8.1	8.1	5.6	18.1	12 (100%)	0 (0%)	0.8	3.34	-0.65	0	0	-2	10
	Follow-up day 45	12 (100%)	0 (0%)	8	0.46	8.1	8.1	8.1	6.5	8.1	12 (100%)	0 (0%)	0.4	1.4	0	0	0	-0.4	4.8

Executed script: \\SDS-SERVER2\SAS$\SDS3\Projects\STATISTICS DRUG DEV\NBMI4011 - CTI002\Analyser\Script\SAS\T_cat001.sas, on: 23MAY2016:14:00, by: maria.lidsell
Version of source data: nbmi_neu.sas7bdat, last modified: 24FEB16:23:13, status: Final

Table DE5.49.1 Change from baseline of NMB, Max. Frequency test - Finger tapping, Right hand, MF Index (Full analysis set)

		Result									Change from baseline (Visit day 1)								
**Treatment**	**Visit**	**n**	**nmiss**	**Mean**	**SD**	**Q1**	**Median**	**Q3**	**Min**	**Max**	**n**	**nmiss**	**Mean**	**SD**	**Q1**	**Median**	**Q3**	**Min**	**Max**
Total (n=36)	Visit day 1	36 (100%)	0 (0%)	186.8	36.39	196	196	196	11	196									
	Follow-up day 15	36 (100%)	0 (0%)	184.9	33.88	196	196	196	11	196	36 (100%)	0 (0%)	-1.9	50.91	0	0	0	-185	185
	Follow-up day 45	36 (100%)	0 (0%)	186.6	37.74	196	196	196	2	196	36 (100%)	0 (0%)	-0.2	39.47	0	0	0	-122	185
NBMI100mg (n=13)	Visit day 1	13 (100%)	0 (0%)	186.6	33.84	196	196	196	74	196									
	Follow-up day 15	13 (100%)	0 (0%)	179.4	50.97	196	196	196	11	196	13 (100%)	0 (0%)	-7.2	63.88	0	0	0	-185	122
	Follow-up day 45	13 (100%)	0 (0%)	171.7	61.13	196	196	196	2	196	13 (100%)	0 (0%)	-14.9	37.83	0	0	0	-122	0
NBMI300mg (n=11)	Visit day 1	11 (100%)	0 (0%)	194.9	3.62	196	196	196	184	196									
	Follow-up day 15	11 (100%)	0 (0%)	190.1	19.27	196	196	196	132	196	11 (100%)	0 (0%)	-4.8	15.65	0	0	0	-52	0
	Follow-up day 45	11 (100%)	0 (0%)	195.9	0.3	196	196	196	195	196	11 (100%)	0 (0%)	1	3.32	0	0	0	0	11
Placebo (n=12)	Visit day 1	12 (100%)	0 (0%)	179.5	53.2	196	196	196	11	196									
	Follow-up day 15	12 (100%)	0 (0%)	186.1	20.39	186.5	196	196	132	196	12 (100%)	0 (0%)	6.6	58.75	-9.5	0	0	-51	185
	Follow-up day 45	12 (100%)	0 (0%)	194.1	6.64	196	196	196	173	196	12 (100%)	0 (0%)	14.6	53.74	0	0	0	-10	185

Executed script: \\SDS-SERVER2\SAS$\SDS3\Projects\STATISTICS DRUG DEV\NBMI4011 - CTI002\Analyser\Script\SAS\T_cat001.sas, on: 23MAY2016:14:00, by: maria.lidsell
Version of source data: nbmi_neu.sas7bdat, last modified: 24FEB16:23:13, status: Final

Table DE5.50.1 Change from baseline of NMB, Max. Frequency test - Finger tapping, Left hand, Max Frequency (Full analysis set)

		Result									Change from baseline (Visit day 1)								
**Treatment**	**Visit**	**n**	**nmiss**	**Mean**	**SD**	**Q1**	**Median**	**Q3**	**Min**	**Max**	**n**	**nmiss**	**Mean**	**SD**	**Q1**	**Median**	**Q3**	**Min**	**Max**
Total (n=36)	Visit day 1	36 (100%)	0 (0%)	7.8	0.84	8.1	8.1	8.1	4.8	8.1									
	Follow-up day 15	36 (100%)	0 (0%)	7.8	0.7	8.1	8.1	8.1	4.9	8.1	36 (100%)	0 (0%)	0.1	1.17	0	0	0	-3.2	3.3
	Follow-up day 45	36 (100%)	0 (0%)	7.9	0.94	8.1	8.1	8.1	3.1	8.1	36 (100%)	0 (0%)	0.1	1.19	0	0	0	-3.8	3.3
NBMI100mg (n=13)	Visit day 1	13 (100%)	0 (0%)	7.6	1.12	8.1	8.1	8.1	4.8	8.1									
	Follow-up day 15	13 (100%)	0 (0%)	7.8	0.92	8.1	8.1	8.1	4.9	8.1	13 (100%)	0 (0%)	0.1	1.56	0	0	0	-3.2	3.3
	Follow-up day 45	13 (100%)	0 (0%)	8	0.23	8.1	8.1	8.1	7.3	8.1	13 (100%)	0 (0%)	0.4	1.18	0	0	0	-0.8	3.3
NBMI300mg (n=11)	Visit day 1	11 (100%)	0 (0%)	8	0.29	8.1	8.1	8.1	7.2	8.1									
	Follow-up day 15	11 (100%)	0 (0%)	8.1	0.09	8.1	8.1	8.1	7.8	8.1	11 (100%)	0 (0%)	0.1	0.21	0	0	0	0	0.6
	Follow-up day 45	11 (100%)	0 (0%)	7.9	0.81	8.1	8.1	8.1	5.4	8.1	11 (100%)	0 (0%)	-0.1	0.9	0	0	0	-2.7	0.9
Placebo (n=12)	Visit day 1	12 (100%)	0 (0%)	7.7	0.86	7.55	8.1	8.1	5.3	8.1									
	Follow-up day 15	12 (100%)	0 (0%)	7.7	0.73	7.5	8.1	8.1	6.1	8.1	12 (100%)	0 (0%)	0	1.28	-0.6	0	0.55	-2	2.8
	Follow-up day 45	12 (100%)	0 (0%)	7.7	1.44	8.1	8.1	8.1	3.1	8.1	12 (100%)	0 (0%)	0	1.46	0	0	0.05	-3.8	2.8

Executed script: \\SDS-SERVER2\SAS$\SDS3\Projects\STATISTICS DRUG DEV\NBMI4011 - CTI002\Analyser\Script\SAS\T_cat001.sas, on: 23MAY2016:14:00, by: maria.lidsell
Version of source data: nbmi_neu.sas7bdat, last modified: 24FEB16:23:13, status: Final

Table DE5.51.1 Change from baseline of NMB, Max. Frequency test - Finger tapping, Left hand, MF Index (Full analysis set)

		Result									Change from baseline (Visit day 1)								
**Treatment**	**Visit**	**n**	**nmiss**	**Mean**	**SD**	**Q1**	**Median**	**Q3**	**Min**	**Max**	**n**	**nmiss**	**Mean**	**SD**	**Q1**	**Median**	**Q3**	**Min**	**Max**
Total (n=36)	Visit day 1	36 (100%)	0 (0%)	189.2	25.46	197	197	197	70	197									
	Follow-up day 15	36 (100%)	0 (0%)	192.1	19.97	197	197	197	80	197	36 (100%)	0 (0%)	2.9	33.56	0	0	0	-117	127
	Follow-up day 45	36 (100%)	0 (0%)	189.9	32.63	197	197	197	10	197	36 (100%)	0 (0%)	0.7	41.48	0	0	0	-177	127
NBMI100mg (n=13)	Visit day 1	13 (100%)	0 (0%)	182.8	37.39	197	197	197	70	197									
	Follow-up day 15	13 (100%)	0 (0%)	187.3	32.34	197	197	197	80	197	13 (100%)	0 (0%)	4.5	52.35	0	0	0	-117	127
	Follow-up day 45	13 (100%)	0 (0%)	196.5	1.39	197	197	197	192	197	13 (100%)	0 (0%)	13.7	37.6	0	0	0	-5	127
NBMI300mg (n=11)	Visit day 1	11 (100%)	0 (0%)	196.3	1.85	197	197	197	191	197									
	Follow-up day 15	11 (100%)	0 (0%)	196.9	0.3	197	197	197	196	197	11 (100%)	0 (0%)	0.6	1.57	0	0	0	0	5
	Follow-up day 45	11 (100%)	0 (0%)	191.2	19.3	197	197	197	133	197	11 (100%)	0 (0%)	-5.1	19.62	0	0	0	-64	6
Placebo (n=12)	Visit day 1	12 (100%)	0 (0%)	189.5	20.9	193.5	197	197	124	197									
	Follow-up day 15	12 (100%)	0 (0%)	192.8	8.76	192.5	197	197	169	197	12 (100%)	0 (0%)	3.3	24.14	-4.5	0	3.5	-28	73
	Follow-up day 45	12 (100%)	0 (0%)	181.4	53.98	197	197	197	10	197	12 (100%)	0 (0%)	-8.1	57.15	0	0	0	-177	73

Executed script: \\SDS-SERVER2\SAS$\SDS3\Projects\STATISTICS DRUG DEV\NBMI4011 - CTI002\Analyser\Script\SAS\T_cat001.sas, on: 23MAY2016:14:00, by: maria.lidsell
Version of source data: nbmi_neu.sas7bdat, last modified: 24FEB16:23:13, status: Final

Table DE5.52.1 Change from baseline of NMB, Reaction time, Right hand, Average Time (Full analysis set)

		Result									Change from baseline (Visit day 1)								
**Treatment**	**Visit**	**n**	**nmiss**	**Mean**	**SD**	**Q1**	**Median**	**Q3**	**Min**	**Max**	**n**	**nmiss**	**Mean**	**SD**	**Q1**	**Median**	**Q3**	**Min**	**Max**
Total (n=36)	Visit day 1	36 (100%)	0 (0%)	0.3	0.06	0.2235	0.2445	0.259	0.205	0.552									
	Follow-up day 15	36 (100%)	0 (0%)	0.2	0.04	0.224	0.236	0.266	0.18	0.354	36 (100%)	0 (0%)	0	0.06	-0.024	-0.009	0.0245	-0.29	0.065
	Follow-up day 45	36 (100%)	0 (0%)	0.3	0.03	0.2325	0.252	0.2745	0.194	0.366	36 (100%)	0 (0%)	0	0.07	-0.017	0.0035	0.03	-0.334	0.116
NBMI100mg (n=13)	Visit day 1	13 (100%)	0 (0%)	0.3	0.09	0.23	0.25	0.281	0.205	0.552									
	Follow-up day 15	13 (100%)	0 (0%)	0.3	0.05	0.23	0.254	0.313	0.183	0.354	13 (100%)	0 (0%)	0	0.09	-0.022	0.014	0.055	-0.29	0.065
	Follow-up day 45	13 (100%)	0 (0%)	0.3	0.04	0.235	0.253	0.277	0.218	0.366	13 (100%)	0 (0%)	0	0.11	-0.014	-0.008	0.026	-0.334	0.116
NBMI300mg (n=11)	Visit day 1	11 (100%)	0 (0%)	0.2	0.04	0.215	0.238	0.245	0.21	0.363									
	Follow-up day 15	11 (100%)	0 (0%)	0.2	0.03	0.224	0.254	0.28	0.183	0.296	11 (100%)	0 (0%)	0	0.04	-0.021	0.01	0.033	-0.067	0.065
	Follow-up day 45	11 (100%)	0 (0%)	0.3	0.03	0.242	0.252	0.272	0.194	0.284	11 (100%)	0 (0%)	0	0.04	-0.016	0.018	0.035	-0.083	0.046
Placebo (n=12)	Visit day 1	12 (100%)	0 (0%)	0.2	0.03	0.224	0.255	0.2575	0.208	0.291									
	Follow-up day 15	12 (100%)	0 (0%)	0.2	0.02	0.213	0.231	0.2345	0.18	0.26	12 (100%)	0 (0%)	0	0.03	-0.035	-0.0205	-0.0065	-0.078	0.024
	Follow-up day 45	12 (100%)	0 (0%)	0.2	0.03	0.212	0.246	0.274	0.203	0.296	12 (100%)	0 (0%)	0	0.03	-0.024	0	0.018	-0.045	0.061

Executed script: \\SDS-SERVER2\SAS$\SDS3\Projects\STATISTICS DRUG DEV\NBMI4011 - CTI002\Analyser\Script\SAS\T_cat001.sas, on: 23MAY2016:14:00, by: maria.lidsell
Version of source data: nbmi_neu.sas7bdat, last modified: 24FEB16:23:13, status: Final

Table DE5.53.1 Change from baseline of NMB, Reaction time, Right hand, Standard Deviation (Full analysis set)

		Result									Change from baseline (Visit day 1)								
**Treatment**	**Visit**	**n**	**nmiss**	**Mean**	**SD**	**Q1**	**Median**	**Q3**	**Min**	**Max**	**n**	**nmiss**	**Mean**	**SD**	**Q1**	**Median**	**Q3**	**Min**	**Max**
Total (n=36)	Visit day 1	36 (100%)	0 (0%)	0.1	0.05	0.0355	0.041	0.054	0.02	0.332									
	Follow-up day 15	36 (100%)	0 (0%)	0.1	0.04	0.0295	0.038	0.0625	0.011	0.173	36 (100%)	0 (0%)	0	0.06	-0.012	-0.004	0.011	-0.264	0.139
	Follow-up day 45	36 (100%)	0 (0%)	0.1	0.05	0.0305	0.0385	0.0665	0.014	0.313	36 (100%)	0 (0%)	0	0.07	-0.0125	-0.0015	0.016	-0.297	0.261
NBMI100mg (n=13)	Visit day 1	13 (100%)	0 (0%)	0.1	0.08	0.034	0.039	0.052	0.023	0.332									
	Follow-up day 15	13 (100%)	0 (0%)	0.1	0.05	0.034	0.055	0.106	0.011	0.173	13 (100%)	0 (0%)	0	0.09	-0.003	0.004	0.051	-0.264	0.139
	Follow-up day 45	13 (100%)	0 (0%)	0.1	0.08	0.033	0.035	0.047	0.014	0.313	13 (100%)	0 (0%)	0	0.12	-0.01	-0.002	0.016	-0.297	0.261
NBMI300mg (n=11)	Visit day 1	11 (100%)	0 (0%)	0	0.02	0.029	0.039	0.047	0.02	0.096									
	Follow-up day 15	11 (100%)	0 (0%)	0	0.04	0.024	0.029	0.077	0.017	0.164	11 (100%)	0 (0%)	0	0.03	-0.02	-0.009	0.006	-0.023	0.068
	Follow-up day 45	11 (100%)	0 (0%)	0	0.03	0.03	0.038	0.057	0.02	0.113	11 (100%)	0 (0%)	0	0.04	-0.018	-0.009	0.024	-0.058	0.082
Placebo (n=12)	Visit day 1	12 (100%)	0 (0%)	0.1	0.02	0.04	0.05	0.068	0.03	0.095									
	Follow-up day 15	12 (100%)	0 (0%)	0	0.01	0.032	0.0455	0.0505	0.016	0.06	12 (100%)	0 (0%)	0	0.02	-0.0205	-0.0095	-0.004	-0.079	0.022
	Follow-up day 45	12 (100%)	0 (0%)	0.1	0.03	0.0275	0.06	0.079	0.018	0.109	12 (100%)	0 (0%)	0	0.02	-0.0115	0.001	0.0165	-0.031	0.037

Executed script: \\SDS-SERVER2\SAS$\SDS3\Projects\STATISTICS DRUG DEV\NBMI4011 - CTI002\Analyser\Script\SAS\T_cat001.sas, on: 23MAY2016:14:00, by: maria.lidsell
Version of source data: nbmi_neu.sas7bdat, last modified: 24FEB16:23:13, status: Final

Table DE5.54.1 Change from baseline of NMB, Reaction time, Right hand, RT Index (Full analysis set)

		Result									Change from baseline (Visit day 1)								
**Treatment**	**Visit**	**n**	**nmiss**	**Mean**	**SD**	**Q1**	**Median**	**Q3**	**Min**	**Max**	**n**	**nmiss**	**Mean**	**SD**	**Q1**	**Median**	**Q3**	**Min**	**Max**
Total (n=36)	Visit day 1	36 (100%)	0 (0%)	49.3	34.32	27	40.5	73	0	118									
	Follow-up day 15	36 (100%)	0 (0%)	54.7	43.96	22.5	52	72	2	159	36 (100%)	0 (0%)	5.4	42.97	-24	10.5	27	-76	131
	Follow-up day 45	36 (100%)	0 (0%)	43.2	34.57	17.5	33	56.5	1	139	36 (100%)	0 (0%)	-6.1	43.68	-33	-2.5	13.5	-85	84
NBMI100mg (n=13)	Visit day 1	13 (100%)	0 (0%)	44.5	37.73	15	34	60	0	118									
	Follow-up day 15	13 (100%)	0 (0%)	41.2	43.75	6	31	61	2	155	13 (100%)	0 (0%)	-3.2	32.26	-28	-2	21	-56	37
	Follow-up day 45	13 (100%)	0 (0%)	34.1	24.6	16	32	52	1	84	13 (100%)	0 (0%)	-10.4	45.05	-33	2	13	-85	84
NBMI300mg (n=11)	Visit day 1	11 (100%)	0 (0%)	55.5	32.06	40	49	91	1	104									
	Follow-up day 15	11 (100%)	0 (0%)	47.2	42.51	15	31	72	10	155	11 (100%)	0 (0%)	-8.4	48.75	-42	-10	12	-76	102
	Follow-up day 45	11 (100%)	0 (0%)	41.5	35.98	19	33	44	14	139	11 (100%)	0 (0%)	-14	33.43	-33	-12	14	-74	35
Placebo (n=12)	Visit day 1	12 (100%)	0 (0%)	48.8	34.55	28.5	30	72	11	111									
	Follow-up day 15	12 (100%)	0 (0%)	76.2	40.55	54	59.5	98	26	159	12 (100%)	0 (0%)	27.3	42.16	12.5	25.5	37	-53	131
	Follow-up day 45	12 (100%)	0 (0%)	54.5	41.54	18	39	100.5	10	122	12 (100%)	0 (0%)	5.7	51.05	-30.5	1	52.5	-70	75

Executed script: \\SDS-SERVER2\SAS$\SDS3\Projects\STATISTICS DRUG DEV\NBMI4011 - CTI002\Analyser\Script\SAS\T_cat001.sas, on: 23MAY2016:14:00, by: maria.lidsell
Version of source data: nbmi_neu.sas7bdat, last modified: 24FEB16:23:13, status: Final

Table DE5.55.1 Change from baseline of NMB, Reaction time, Left hand, Average Time (Full analysis set)

		Result									Change from baseline (Visit day 1)								
**Treatment**	**Visit**	**n**	**nmiss**	**Mean**	**SD**	**Q1**	**Median**	**Q3**	**Min**	**Max**	**n**	**nmiss**	**Mean**	**SD**	**Q1**	**Median**	**Q3**	**Min**	**Max**
Total (n=36)	Visit day 1	36 (100%)	0 (0%)	0.2	0.03	0.226	0.242	0.2625	0.181	0.366									
	Follow-up day 15	36 (100%)	0 (0%)	0.3	0.07	0.2315	0.2495	0.279	0.02	0.514	36 (100%)	0 (0%)	0	0.07	-0.0235	0.0065	0.0485	-0.207	0.29
	Follow-up day 45	36 (100%)	0 (0%)	0.3	0.04	0.234	0.2575	0.2975	0.198	0.388	36 (100%)	0 (0%)	0	0.05	-0.0135	0.0125	0.055	-0.075	0.128
NBMI100mg (n=13)	Visit day 1	13 (100%)	0 (0%)	0.2	0.03	0.224	0.244	0.26	0.183	0.299									
	Follow-up day 15	13 (100%)	0 (0%)	0.3	0.08	0.241	0.277	0.298	0.207	0.514	13 (100%)	0 (0%)	0	0.09	-0.004	0.013	0.053	-0.063	0.29
	Follow-up day 45	13 (100%)	0 (0%)	0.3	0.04	0.238	0.266	0.281	0.215	0.388	13 (100%)	0 (0%)	0	0.06	-0.007	0.016	0.066	-0.075	0.128
NBMI300mg (n=11)	Visit day 1	11 (100%)	0 (0%)	0.3	0.04	0.231	0.246	0.269	0.21	0.366									
	Follow-up day 15	11 (100%)	0 (0%)	0.3	0.03	0.239	0.265	0.273	0.197	0.3	11 (100%)	0 (0%)	0	0.05	-0.025	0.015	0.048	-0.085	0.061
	Follow-up day 45	11 (100%)	0 (0%)	0.3	0.05	0.23	0.238	0.305	0.217	0.331	11 (100%)	0 (0%)	0	0.05	-0.029	0.01	0.049	-0.047	0.098
Placebo (n=12)	Visit day 1	12 (100%)	0 (0%)	0.2	0.03	0.219	0.236	0.2585	0.181	0.284									
	Follow-up day 15	12 (100%)	0 (0%)	0.2	0.07	0.227	0.2325	0.2385	0.02	0.334	12 (100%)	0 (0%)	0	0.07	-0.0235	-0.007	0.018	-0.207	0.054
	Follow-up day 45	12 (100%)	0 (0%)	0.3	0.05	0.23	0.2455	0.2875	0.198	0.36	12 (100%)	0 (0%)	0	0.05	-0.0075	0.0125	0.0565	-0.043	0.123

Executed script: \\SDS-SERVER2\SAS$\SDS3\Projects\STATISTICS DRUG DEV\NBMI4011 - CTI002\Analyser\Script\SAS\T_cat001.sas, on: 23MAY2016:14:00, by: maria.lidsell
Version of source data: nbmi_neu.sas7bdat, last modified: 24FEB16:23:13, status: Final

Table DE5.56.1 Change from baseline of NMB, Reaction time, Left hand, Standard Deviation (Full analysis set)

		Result									Change from baseline (Visit day 1)								
**Treatment**	**Visit**	**n**	**nmiss**	**Mean**	**SD**	**Q1**	**Median**	**Q3**	**Min**	**Max**	**n**	**nmiss**	**Mean**	**SD**	**Q1**	**Median**	**Q3**	**Min**	**Max**
Total (n=36)	Visit day 1	36 (100%)	0 (0%)	0	0.02	0.026	0.0335	0.048	0.016	0.103									
	Follow-up day 15	36 (100%)	0 (0%)	0.1	0.07	0.0315	0.043	0.0525	0.018	0.43	36 (100%)	0 (0%)	0	0.07	-0.002	0.007	0.0215	-0.054	0.387
	Follow-up day 45	36 (100%)	0 (0%)	0.1	0.04	0.0345	0.045	0.0685	0.014	0.199	36 (100%)	0 (0%)	0	0.05	-0.0065	0.011	0.0285	-0.053	0.173
NBMI100mg (n=13)	Visit day 1	13 (100%)	0 (0%)	0	0.02	0.026	0.03	0.041	0.022	0.079									
	Follow-up day 15	13 (100%)	0 (0%)	0.1	0.11	0.045	0.051	0.067	0.018	0.43	13 (100%)	0 (0%)	0	0.11	-0.001	0.025	0.029	-0.033	0.387
	Follow-up day 45	13 (100%)	0 (0%)	0.1	0.05	0.031	0.04	0.065	0.014	0.199	13 (100%)	0 (0%)	0	0.06	-0.016	0.009	0.028	-0.048	0.173
NBMI300mg (n=11)	Visit day 1	11 (100%)	0 (0%)	0	0.03	0.023	0.032	0.05	0.02	0.103									
	Follow-up day 15	11 (100%)	0 (0%)	0	0.04	0.027	0.037	0.05	0.018	0.137	11 (100%)	0 (0%)	0	0.04	-0.002	0.005	0.013	-0.054	0.113
	Follow-up day 45	11 (100%)	0 (0%)	0	0.02	0.036	0.045	0.072	0.022	0.082	11 (100%)	0 (0%)	0	0.03	-0.004	0.013	0.024	-0.053	0.058
Placebo (n=12)	Visit day 1	12 (100%)	0 (0%)	0	0.02	0.0305	0.0355	0.05	0.016	0.073									
	Follow-up day 15	12 (100%)	0 (0%)	0	0.02	0.031	0.036	0.047	0.027	0.094	12 (100%)	0 (0%)	0	0.01	-0.0065	0.001	0.0115	-0.02	0.021
	Follow-up day 45	12 (100%)	0 (0%)	0.1	0.04	0.033	0.0495	0.0755	0.024	0.177	12 (100%)	0 (0%)	0	0.04	-0.005	0.0095	0.033	-0.018	0.143

Executed script: \\SDS-SERVER2\SAS$\SDS3\Projects\STATISTICS DRUG DEV\NBMI4011 - CTI002\Analyser\Script\SAS\T_cat001.sas, on: 23MAY2016:14:00, by: maria.lidsell
Version of source data: nbmi_neu.sas7bdat, last modified: 24FEB16:23:13, status: Final

Table DE5.57.1 Change from baseline of NMB, Reaction time, Left hand, RT Index (Full analysis set)

		Result									Change from baseline (Visit day 1)								
**Treatment**	**Visit**	**n**	**nmiss**	**Mean**	**SD**	**Q1**	**Median**	**Q3**	**Min**	**Max**	**n**	**nmiss**	**Mean**	**SD**	**Q1**	**Median**	**Q3**	**Min**	**Max**
Total (n=36)	Visit day 1	36 (100%)	0 (0%)	52.6	38.63	24.5	43.5	68.5	1	158									
	Follow-up day 15	36 (100%)	0 (0%)	43.4	35.4	15.5	35	58	0	145	36 (100%)	0 (0%)	-9.2	49.53	-34	-4	18	-147	113
	Follow-up day 45	36 (100%)	0 (0%)	36.5	31.59	9.5	28.5	54.5	1	131	36 (100%)	0 (0%)	-16.1	47.39	-40	-14.5	6.5	-140	86
NBMI100mg (n=13)	Visit day 1	13 (100%)	0 (0%)	51.8	39.71	27	42	71	9	155									
	Follow-up day 15	13 (100%)	0 (0%)	32.8	34.07	9	16	45	0	112	13 (100%)	0 (0%)	-18.9	58.57	-45	-10	8	-147	85
	Follow-up day 45	13 (100%)	0 (0%)	30.1	25.22	15	22	48	1	92	13 (100%)	0 (0%)	-21.7	51.43	-33	-14	6	-140	81
NBMI300mg (n=11)	Visit day 1	11 (100%)	0 (0%)	42.5	29.73	21	39	59	1	107									
	Follow-up day 15	11 (100%)	0 (0%)	38.7	36.17	18	23	47	9	134	11 (100%)	0 (0%)	-3.7	52.07	-33	-12	23	-88	113
	Follow-up day 45	11 (100%)	0 (0%)	37.8	31.62	8	49	61	4	87	11 (100%)	0 (0%)	-4.6	35.93	-34	-5	25	-55	54
Placebo (n=12)	Visit day 1	12 (100%)	0 (0%)	62.8	44.82	27	51	85	14	158									
	Follow-up day 15	12 (100%)	0 (0%)	59.2	33.29	47.5	56.5	66	3	145	12 (100%)	0 (0%)	-3.6	37.7	-12	10.5	18	-97	33
	Follow-up day 45	12 (100%)	0 (0%)	42.3	38.52	13	39.5	62	2	131	12 (100%)	0 (0%)	-20.4	53.82	-55.5	-18.5	6	-118	86

Executed script: \\SDS-SERVER2\SAS$\SDS3\Projects\STATISTICS DRUG DEV\NBMI4011 - CTI002\Analyser\Script\SAS\T_cat001.sas, on: 23MAY2016:14:00, by: maria.lidsell
Version of source data: nbmi_neu.sas7bdat, last modified: 24FEB16:23:13, status: Final

Table DE6.1.1 Change from usual of Total Fatigue Score Sum (Full analysis set)

		Result									Change from usual*								
**Treatment**	**Visit**	**n**	**nmiss**	**Mean**	**SD**	**Q1**	**Median**	**Q3**	**Min**	**Max**	**n**	**nmiss**	**Mean**	**SD**	**Q1**	**Median**	**Q3**	**Min**	**Max**
Total (n=36)	Visit day 2	36 (100%)	0 (0%)	13	0.17	13	13	13	12	13	36 (100%)	0 (0%)	0	0.17	0	0	0	-1	0
	Visit day 3	36 (100%)	0 (0%)	11.3	3.05	10.5	13	13	3	15	36 (100%)	0 (0%)	-1.7	3.05	-2.5	0	0	-10	2
	Visit day 4	36 (100%)	0 (0%)	11.3	2.73	10	13	13	3	14	36 (100%)	0 (0%)	-1.7	2.73	-3	0	0	-10	1
	Visit day 5	36 (100%)	0 (0%)	10.9	2.94	9	12	13	0	13	36 (100%)	0 (0%)	-2.1	2.94	-4	-1	0	-13	0
	Visit day 6	36 (100%)	0 (0%)	10.4	3.67	9	12	13	0	14	36 (100%)	0 (0%)	-2.6	3.67	-4	-1	0	-13	1
	Visit day 7	36 (100%)	0 (0%)	9.8	3.91	7	12	13	0	15	36 (100%)	0 (0%)	-3.2	3.91	-6	-1	0	-13	2
	Visit day 8	36 (100%)	0 (0%)	9.1	3.91	6	10	13	0	13	36 (100%)	0 (0%)	-3.9	3.91	-7	-3	0	-13	0
	Visit day 9	36 (100%)	0 (0%)	10	3.48	7	11	13	3	16	36 (100%)	0 (0%)	-3	3.48	-6	-2	0	-10	3
	Visit day 10	36 (100%)	0 (0%)	9.9	3.62	9	11	13	0	14	36 (100%)	0 (0%)	-3.1	3.62	-4	-2	0	-13	1
	Visit day 11	36 (100%)	0 (0%)	9.7	3.11	7.5	11	12.5	0	13	36 (100%)	0 (0%)	-3.3	3.11	-5.5	-2	-0.5	-13	0
	Visit day 12	36 (100%)	0 (0%)	9.8	3.38	8	10	13	0	13	36 (100%)	0 (0%)	-3.2	3.38	-5	-3	0	-13	0
	Visit day 13	36 (100%)	0 (0%)	8.3	4.63	4	9.5	13	0	14	36 (100%)	0 (0%)	-4.7	4.63	-9	-3.5	0	-13	1
	Follow-up day 15	36 (100%)	0 (0%)	7.8	4.64	4.5	9.5	11.5	0	13	36 (100%)	0 (0%)	-5.3	4.64	-8.5	-3.5	-1.5	-13	0
	Follow-up day 45	36 (100%)	0 (0%)	7.7	4.62	5	8	12	0	14	36 (100%)	0 (0%)	-5.3	4.62	-8	-5	-1	-13	1
NBMI100mg (n=13)	Visit day 2	13 (100%)	0 (0%)	13	0	13	13	13	13	13	13 (100%)	0 (0%)	0	0	0	0	0	0	0
	Visit day 3	13 (100%)	0 (0%)	10.6	3.57	8	13	13	4	13	13 (100%)	0 (0%)	-2.4	3.57	-5	0	0	-9	0
	Visit day 4	13 (100%)	0 (0%)	11.5	1.76	10	13	13	9	13	13 (100%)	0 (0%)	-1.5	1.76	-3	0	0	-4	0
	Visit day 5	13 (100%)	0 (0%)	10.3	3.61	9	10	13	0	13	13 (100%)	0 (0%)	-2.7	3.61	-4	-3	0	-13	0
	Visit day 6	13 (100%)	0 (0%)	9.5	4.37	7	11	13	0	13	13 (100%)	0 (0%)	-3.5	4.37	-6	-2	0	-13	0
	Visit day 7	13 (100%)	0 (0%)	10.2	3.95	9	12	13	0	13	13 (100%)	0 (0%)	-2.8	3.95	-4	-1	0	-13	0
	Visit day 8	13 (100%)	0 (0%)	9.1	4.37	6	11	13	2	13	13 (100%)	0 (0%)	-3.9	4.37	-7	-2	0	-11	0
	Visit day 9	13 (100%)	0 (0%)	10.5	3.64	9	11	13	3	15	13 (100%)	0 (0%)	-2.5	3.64	-4	-2	0	-10	2
	Visit day 10	13 (100%)	0 (0%)	9.7	4.48	9	11	13	0	14	13 (100%)	0 (0%)	-3.3	4.48	-4	-2	0	-13	1
	Visit day 11	13 (100%)	0 (0%)	10.2	2.24	8	11	12	7	13	13 (100%)	0 (0%)	-2.8	2.24	-5	-2	-1	-6	0
	Visit day 12	13 (100%)	0 (0%)	10.4	2.02	9	10	13	8	13	13 (100%)	0 (0%)	-2.6	2.02	-4	-3	0	-5	0

		Result									Change from usual*								
**Treatment**	**Visit**	**n**	**nmiss**	**Mean**	**SD**	**Q1**	**Median**	**Q3**	**Min**	**Max**	**n**	**nmiss**	**Mean**	**SD**	**Q1**	**Median**	**Q3**	**Min**	**Max**
	Visit day 13	13 (100%)	0 (0%)	8.8	4.67	8	9	13	0	14	13 (100%)	0 (0%)	-4.2	4.67	-5	-4	0	-13	1
	Follow-up day 15	13 (100%)	0 (0%)	7.2	4.95	4	8	11	0	13	13 (100%)	0 (0%)	-5.8	4.95	-9	-5	-2	-13	0
	Follow-up day 45	13 (100%)	0 (0%)	8	5	4	8	12	0	14	13 (100%)	0 (0%)	-5	5	-9	-5	-1	-13	1
NBMI300mg (n=11)	Visit day 2	11 (100%)	0 (0%)	12.9	0.3	13	13	13	12	13	11 (100%)	0 (0%)	-0.1	0.3	0	0	0	-1	0
	Visit day 3	11 (100%)	0 (0%)	11.6	1.96	10	13	13	7	13	11 (100%)	0 (0%)	-1.4	1.96	-3	0	0	-6	0
	Visit day 4	11 (100%)	0 (0%)	10.7	3.93	10	13	13	3	13	11 (100%)	0 (0%)	-2.3	3.93	-3	0	0	-10	0
	Visit day 5	11 (100%)	0 (0%)	11.4	1.69	10	12	13	8	13	11 (100%)	0 (0%)	-1.6	1.69	-3	-1	0	-5	0
	Visit day 6	11 (100%)	0 (0%)	10.8	3.63	9	12	13	1	14	11 (100%)	0 (0%)	-2.2	3.63	-4	-1	0	-12	1
	Visit day 7	11 (100%)	0 (0%)	9.2	3.92	7	11	12	0	13	11 (100%)	0 (0%)	-3.8	3.92	-6	-2	-1	-13	0
	Visit day 8	11 (100%)	0 (0%)	9.1	3.3	6	10	12	3	13	11 (100%)	0 (0%)	-3.9	3.3	-7	-3	-1	-10	0
	Visit day 9	11 (100%)	0 (0%)	9.5	4.01	6	11	12	3	16	11 (100%)	0 (0%)	-3.5	4.01	-7	-2	-1	-10	3
	Visit day 10	11 (100%)	0 (0%)	9.5	2.84	7	9	12	5	13	11 (100%)	0 (0%)	-3.5	2.84	-6	-4	-1	-8	0
	Visit day 11	11 (100%)	0 (0%)	8.2	3.82	6	9	11	0	12	11 (100%)	0 (0%)	-4.8	3.82	-7	-4	-2	-13	-1
	Visit day 12	11 (100%)	0 (0%)	9	4.4	7	11	12	0	13	11 (100%)	0 (0%)	-4	4.4	-6	-2	-1	-13	0
	Visit day 13	11 (100%)	0 (0%)	6.5	4.76	2	8	11	0	12	11 (100%)	0 (0%)	-6.5	4.76	-11	-5	-2	-13	-1
	Follow-up day 15	11 (100%)	0 (0%)	6.3	4.63	2	7	10	0	12	11 (100%)	0 (0%)	-6.7	4.63	-11	-6	-3	-13	-1
	Follow-up day 45	11 (100%)	0 (0%)	5.8	4.6	0	7	9	0	13	11 (100%)	0 (0%)	-7.2	4.6	-13	-6	-4	-13	0
Placebo (n=12)	Visit day 2	12 (100%)	0 (0%)	13	0	13	13	13	13	13	12 (100%)	0 (0%)	0	0	0	0	0	0	0
	Visit day 3	12 (100%)	0 (0%)	11.8	3.35	13	13	13	3	15	12 (100%)	0 (0%)	-1.2	3.35	0	0	0	-10	2
	Visit day 4	12 (100%)	0 (0%)	11.5	2.43	10.5	13	13	6	14	12 (100%)	0 (0%)	-1.5	2.43	-2.5	0	0	-7	1
	Visit day 5	12 (100%)	0 (0%)	11.1	3.18	9.5	13	13	4	13	12 (100%)	0 (0%)	-1.9	3.18	-3.5	0	0	-9	0
	Visit day 6	12 (100%)	0 (0%)	11	2.92	9.5	13	13	5	13	12 (100%)	0 (0%)	-2	2.92	-3.5	0	0	-8	0
	Visit day 7	12 (100%)	0 (0%)	10	4.13	6.5	12.5	13	2	15	12 (100%)	0 (0%)	-3	4.13	-6.5	-0.5	0	-11	2
	Visit day 8	12 (100%)	0 (0%)	9.3	4.22	6	10	13	0	13	12 (100%)	0 (0%)	-3.8	4.22	-7	-3	0	-13	0
	Visit day 9	12 (100%)	0 (0%)	10.1	3	8.5	10.5	13	4	13	12 (100%)	0 (0%)	-2.9	3	-4.5	-2.5	0	-9	0
	Visit day 10	12 (100%)	0 (0%)	10.3	3.47	9	11	13	1	13	12 (100%)	0 (0%)	-2.7	3.47	-4	-2	0	-12	0
	Visit day 11	12 (100%)	0 (0%)	10.6	2.94	8	12.5	13	6	13	12 (100%)	0 (0%)	-2.4	2.94	-5	-0.5	0	-7	0

		Result									Change from usual*								
**Treatment**	**Visit**	**n**	**nmiss**	**Mean**	**SD**	**Q1**	**Median**	**Q3**	**Min**	**Max**	**n**	**nmiss**	**Mean**	**SD**	**Q1**	**Median**	**Q3**	**Min**	**Max**
	Visit day 12	12 (100%)	0 (0%)	10	3.62	6.5	11.5	13	3	13	12 (100%)	0 (0%)	-3	3.62	-6.5	-1.5	0	-10	0
	Visit day 13	12 (100%)	0 (0%)	9.3	4.41	6.5	10.5	13	0	13	12 (100%)	0 (0%)	-3.8	4.41	-6.5	-2.5	0	-13	0
	Follow-up day 15	12 (100%)	0 (0%)	9.7	3.98	7	11	13	0	13	12 (100%)	0 (0%)	-3.3	3.98	-6	-2	0	-13	0
	Follow-up day 45	12 (100%)	0 (0%)	9.1	3.94	6.5	9.5	12.5	0	13	12 (100%)	0 (0%)	-3.9	3.94	-6.5	-3.5	-0.5	-13	0

Executed script: \\SDS-SERVER2\SAS$\SDS3\Projects\STATISTICS DRUG DEV\NBMI4011 - CTI002\Analyser\Script\SAS\T_fs002.sas, on: 23MAY2016:14:00, by: maria.lidsell
Version of source data: nbmi_ph3.sas7bdat, last modified: 24FEB16:23:13, status: Final
*) Change for physical and mental fatigue score is defined as difference from 1=same as usual.

Table DE6.2.1 Change from usual of Physical Fatigue Score, Total (Full analysis set)

		Result									Change from usual*								
**Treatment**	**Visit**	**n**	**nmiss**	**Mean**	**SD**	**Q1**	**Median**	**Q3**	**Min**	**Max**	**n**	**nmiss**	**Mean**	**SD**	**Q1**	**Median**	**Q3**	**Min**	**Max**
Total (n=36)	Visit day 2	36 (100%)	0 (0%)	8	0.17	8	8	8	7	8	36 (100%)	0 (0%)	0	0.17	0	0	0	-1	0
	Visit day 3	36 (100%)	0 (0%)	6.8	2.24	5.5	8	8	0	10	36 (100%)	0 (0%)	-1.2	2.24	-2.5	0	0	-8	2
	Visit day 4	36 (100%)	0 (0%)	6.7	2.07	6	8	8	0	9	36 (100%)	0 (0%)	-1.3	2.07	-2	0	0	-8	1
	Visit day 5	36 (100%)	0 (0%)	6.4	2.09	5	7.5	8	0	8	36 (100%)	0 (0%)	-1.6	2.09	-3	-0.5	0	-8	0
	Visit day 6	36 (100%)	0 (0%)	6.1	2.33	5	7	8	0	8	36 (100%)	0 (0%)	-1.9	2.33	-3	-1	0	-8	0
	Visit day 7	36 (100%)	0 (0%)	5.8	2.62	4	7	8	0	10	36 (100%)	0 (0%)	-2.3	2.62	-4	-1	0	-8	2
	Visit day 8	36 (100%)	0 (0%)	5.3	2.47	3	5	8	0	8	36 (100%)	0 (0%)	-2.7	2.47	-5	-3	0	-8	0
	Visit day 9	36 (100%)	0 (0%)	5.9	2.41	4	6	8	1	11	36 (100%)	0 (0%)	-2.1	2.41	-4	-2	0	-7	3
	Visit day 10	36 (100%)	0 (0%)	5.6	2.52	4	6	8	0	9	36 (100%)	0 (0%)	-2.4	2.52	-4	-2	0	-8	1
	Visit day 11	36 (100%)	0 (0%)	5.4	2.34	4	6	8	0	8	36 (100%)	0 (0%)	-2.6	2.34	-4	-2	0	-8	0
	Visit day 12	36 (100%)	0 (0%)	5.8	2.21	5	6	8	0	8	36 (100%)	0 (0%)	-2.2	2.21	-3	-2	0	-8	0
	Visit day 13	36 (100%)	0 (0%)	4.6	3	1.5	5	8	0	9	36 (100%)	0 (0%)	-3.4	3	-6.5	-3	0	-8	1
	Follow-up day 15	36 (100%)	0 (0%)	4.4	2.8	2.5	5	6.5	0	8	36 (100%)	0 (0%)	-3.6	2.8	-5.5	-3	-1.5	-8	0
	Follow-up day 45	36 (100%)	0 (0%)	4.4	2.98	2.5	4	7	0	9	36 (100%)	0 (0%)	-3.6	2.98	-5.5	-4	-1	-8	1
NBMI100mg (n=13)	Visit day 2	13 (100%)	0 (0%)	8	0	8	8	8	8	8	13 (100%)	0 (0%)	0	0	0	0	0	0	0
	Visit day 3	13 (100%)	0 (0%)	6.4	2.47	5	8	8	1	8	13 (100%)	0 (0%)	-1.6	2.47	-3	0	0	-7	0
	Visit day 4	13 (100%)	0 (0%)	6.8	1.48	6	8	8	4	8	13 (100%)	0 (0%)	-1.2	1.48	-2	0	0	-4	0
	Visit day 5	13 (100%)	0 (0%)	6.2	2.38	5	7	8	0	8	13 (100%)	0 (0%)	-1.8	2.38	-3	-1	0	-8	0
	Visit day 6	13 (100%)	0 (0%)	5.8	2.59	5	7	8	0	8	13 (100%)	0 (0%)	-2.2	2.59	-3	-1	0	-8	0
	Visit day 7	13 (100%)	0 (0%)	5.9	2.6	5	7	8	0	8	13 (100%)	0 (0%)	-2.1	2.6	-3	-1	0	-8	0
	Visit day 8	13 (100%)	0 (0%)	5.3	2.69	2	6	8	1	8	13 (100%)	0 (0%)	-2.7	2.69	-6	-2	0	-7	0
	Visit day 9	13 (100%)	0 (0%)	6.2	2.35	5	6	8	2	10	13 (100%)	0 (0%)	-1.8	2.35	-3	-2	0	-6	2
	Visit day 10	13 (100%)	0 (0%)	5.5	2.93	4	6	8	0	9	13 (100%)	0 (0%)	-2.5	2.93	-4	-2	0	-8	1
	Visit day 11	13 (100%)	0 (0%)	5.8	1.86	5	6	8	2	8	13 (100%)	0 (0%)	-2.2	1.86	-3	-2	0	-6	0
	Visit day 12	13 (100%)	0 (0%)	6.1	1.5	5	5	8	4	8	13 (100%)	0 (0%)	-1.9	1.5	-3	-3	0	-4	0

		Result									Change from usual*								
**Treatment**	**Visit**	**n**	**nmiss**	**Mean**	**SD**	**Q1**	**Median**	**Q3**	**Min**	**Max**	**n**	**nmiss**	**Mean**	**SD**	**Q1**	**Median**	**Q3**	**Min**	**Max**
	Visit day 13	13 (100%)	0 (0%)	5.1	2.96	4	5	8	0	9	13 (100%)	0 (0%)	-2.9	2.96	-4	-3	0	-8	1
	Follow-up day 15	13 (100%)	0 (0%)	4.2	3	2	4	6	0	8	13 (100%)	0 (0%)	-3.8	3	-6	-4	-2	-8	0
	Follow-up day 45	13 (100%)	0 (0%)	4.3	3.33	2	4	7	0	9	13 (100%)	0 (0%)	-3.7	3.33	-6	-4	-1	-8	1
NBMI300mg (n=11)	Visit day 2	11 (100%)	0 (0%)	7.9	0.3	8	8	8	7	8	11 (100%)	0 (0%)	-0.1	0.3	0	0	0	-1	0
	Visit day 3	11 (100%)	0 (0%)	6.8	1.54	5	8	8	4	8	11 (100%)	0 (0%)	-1.2	1.54	-3	0	0	-4	0
	Visit day 4	11 (100%)	0 (0%)	6.2	2.96	5	8	8	0	8	11 (100%)	0 (0%)	-1.8	2.96	-3	0	0	-8	0
	Visit day 5	11 (100%)	0 (0%)	6.5	1.57	5	7	8	4	8	11 (100%)	0 (0%)	-1.5	1.57	-3	-1	0	-4	0
	Visit day 6	11 (100%)	0 (0%)	6.2	2.44	5	7	8	0	8	11 (100%)	0 (0%)	-1.8	2.44	-3	-1	0	-8	0
	Visit day 7	11 (100%)	0 (0%)	5.2	2.64	3	6	7	0	8	11 (100%)	0 (0%)	-2.8	2.64	-5	-2	-1	-8	0
	Visit day 8	11 (100%)	0 (0%)	5.2	2.27	3	5	7	2	8	11 (100%)	0 (0%)	-2.8	2.27	-5	-3	-1	-6	0
	Visit day 9	11 (100%)	0 (0%)	5.5	2.94	3	7	7	1	11	11 (100%)	0 (0%)	-2.5	2.94	-5	-1	-1	-7	3
	Visit day 10	11 (100%)	0 (0%)	5.1	2.26	3	5	7	2	8	11 (100%)	0 (0%)	-2.9	2.26	-5	-3	-1	-6	0
	Visit day 11	11 (100%)	0 (0%)	4.2	2.6	2	5	7	0	7	11 (100%)	0 (0%)	-3.8	2.6	-6	-3	-1	-8	-1
	Visit day 12	11 (100%)	0 (0%)	5.2	2.86	2	6	7	0	8	11 (100%)	0 (0%)	-2.8	2.86	-6	-2	-1	-8	0
	Visit day 13	11 (100%)	0 (0%)	3.2	2.89	0	3	6	0	7	11 (100%)	0 (0%)	-4.8	2.89	-8	-5	-2	-8	-1
	Follow-up day 15	11 (100%)	0 (0%)	3.5	2.66	1	3	6	0	7	11 (100%)	0 (0%)	-4.5	2.66	-7	-5	-2	-8	-1
	Follow-up day 45	11 (100%)	0 (0%)	3.1	2.7	0	3	5	0	8	11 (100%)	0 (0%)	-4.9	2.7	-8	-5	-3	-8	0
Placebo (n=12)	Visit day 2	12 (100%)	0 (0%)	8	0	8	8	8	8	8	12 (100%)	0 (0%)	0	0	0	0	0	0	0
	Visit day 3	12 (100%)	0 (0%)	7.2	2.62	8	8	8	0	10	12 (100%)	0 (0%)	-0.8	2.62	0	0	0	-8	2
	Visit day 4	12 (100%)	0 (0%)	7.1	1.68	6.5	8	8	3	9	12 (100%)	0 (0%)	-0.9	1.68	-1.5	0	0	-5	1
	Visit day 5	12 (100%)	0 (0%)	6.5	2.32	4.5	8	8	2	8	12 (100%)	0 (0%)	-1.5	2.32	-3.5	0	0	-6	0
	Visit day 6	12 (100%)	0 (0%)	6.5	2.07	5	8	8	2	8	12 (100%)	0 (0%)	-1.5	2.07	-3	0	0	-6	0
	Visit day 7	12 (100%)	0 (0%)	6.1	2.78	3.5	7.5	8	1	10	12 (100%)	0 (0%)	-1.9	2.78	-4.5	-0.5	0	-7	2
	Visit day 8	12 (100%)	0 (0%)	5.4	2.61	4	5	8	0	8	12 (100%)	0 (0%)	-2.6	2.61	-4	-3	0	-8	0
	Visit day 9	12 (100%)	0 (0%)	5.8	2.08	4.5	6	8	2	8	12 (100%)	0 (0%)	-2.2	2.08	-3.5	-2	0	-6	0
	Visit day 10	12 (100%)	0 (0%)	6	2.41	5	6.5	8	0	8	12 (100%)	0 (0%)	-2	2.41	-3	-1.5	0	-8	0
	Visit day 11	12 (100%)	0 (0%)	6.2	2.25	4.5	7.5	8	2	8	12 (100%)	0 (0%)	-1.8	2.25	-3.5	-0.5	0	-6	0

Treatment	Visit	Result n	nmiss	Mean	SD	Q1	Median	Q3	Min	Max	Change from usual* n	nmiss	Mean	SD	Q1	Median	Q3	Min	Max
	Visit day 12	12 (100%)	0 (0%)	6	2.26	4.5	6.5	8	2	8	12 (100%)	0 (0%)	-2	2.26	-3.5	-1.5	0	-6	0
	Visit day 13	12 (100%)	0 (0%)	5.3	2.93	3	6	8	0	8	12 (100%)	0 (0%)	-2.7	2.93	-5	-2	0	-8	0
	Follow-up day 15	12 (100%)	0 (0%)	5.6	2.5	4	6	8	0	8	12 (100%)	0 (0%)	-2.4	2.5	-4	-2	0	-8	0
	Follow-up day 45	12 (100%)	0 (0%)	5.6	2.5	4	6	8	0	8	12 (100%)	0 (0%)	-2.4	2.5	-4	-2	0	-8	0

Executed script: \\SDS-SERVER2\SAS$\SDS3\Projects\STATISTICS DRUG DEV\NBMI4011 - CTI002\Analyser\Script\SAS\T_fs002.sas, on: 23MAY2016:14:00, by: maria.lidsell
Version of source data: nbmi_ph3.sas7bdat, last modified: 24FEB16:23:13, status: Final
*) Change for physical and mental fatigue score is defined as difference from 1=same as usual.

Table DE6.3.1 Change from usual of Mental Fatigue Score, Total (Full analysis set)

		Result									Change from usual*								
**Treatment**	**Visit**	**n**	**nmiss**	**Mean**	**SD**	**Q1**	**Median**	**Q3**	**Min**	**Max**	**n**	**nmiss**	**Mean**	**SD**	**Q1**	**Median**	**Q3**	**Min**	**Max**
Total (n=36)	Visit day 2	36 (100%)	0 (0%)	5	0	5	5	5	5	5	36 (100%)	0 (0%)	0	0	0	0	0	0	0
	Visit day 3	36 (100%)	0 (0%)	4.6	0.94	5	5	5	2	5	36 (100%)	0 (0%)	-0.4	0.94	0	0	0	-3	0
	Visit day 4	36 (100%)	0 (0%)	4.6	0.97	5	5	5	1	5	36 (100%)	0 (0%)	-0.4	0.97	0	0	0	-4	0
	Visit day 5	36 (100%)	0 (0%)	4.5	1.08	4.5	5	5	0	5	36 (100%)	0 (0%)	-0.5	1.08	-0.5	0	0	-5	0
	Visit day 6	36 (100%)	0 (0%)	4.3	1.48	4	5	5	0	6	36 (100%)	0 (0%)	-0.8	1.48	-1	0	0	-5	1
	Visit day 7	36 (100%)	0 (0%)	4.1	1.47	3	5	5	0	5	36 (100%)	0 (0%)	-0.9	1.47	-2	0	0	-5	0
	Visit day 8	36 (100%)	0 (0%)	3.8	1.65	3	5	5	0	5	36 (100%)	0 (0%)	-1.2	1.65	-2	0	0	-5	0
	Visit day 9	36 (100%)	0 (0%)	4.1	1.25	3	5	5	1	5	36 (100%)	0 (0%)	-0.9	1.25	-2	0	0	-4	0
	Visit day 10	36 (100%)	0 (0%)	4.3	1.33	4	5	5	0	5	36 (100%)	0 (0%)	-0.7	1.33	-1	0	0	-5	0
	Visit day 11	36 (100%)	0 (0%)	4.3	1.16	4	5	5	0	5	36 (100%)	0 (0%)	-0.7	1.16	-1	0	0	-5	0
	Visit day 12	36 (100%)	0 (0%)	4.1	1.39	3	5	5	0	5	36 (100%)	0 (0%)	-0.9	1.39	-2	0	0	-5	0
	Visit day 13	36 (100%)	0 (0%)	3.7	1.82	2.5	5	5	0	5	36 (100%)	0 (0%)	-1.3	1.82	-2.5	0	0	-5	0
	Follow-up day 15	36 (100%)	0 (0%)	3.3	1.91	2	4	5	0	5	36 (100%)	0 (0%)	-1.7	1.91	-3	-1	0	-5	0
	Follow-up day 45	36 (100%)	0 (0%)	3.3	1.91	2	4	5	0	6	36 (100%)	0 (0%)	-1.7	1.91	-3	-1	0	-5	1
NBMI100mg (n=13)	Visit day 2	13 (100%)	0 (0%)	5	0	5	5	5	5	5	13 (100%)	0 (0%)	0	0	0	0	0	0	0
	Visit day 3	13 (100%)	0 (0%)	4.2	1.24	3	5	5	2	5	13 (100%)	0 (0%)	-0.8	1.24	-2	0	0	-3	0
	Visit day 4	13 (100%)	0 (0%)	4.8	0.6	5	5	5	3	5	13 (100%)	0 (0%)	-0.2	0.6	0	0	0	-2	0
	Visit day 5	13 (100%)	0 (0%)	4.2	1.46	4	5	5	0	5	13 (100%)	0 (0%)	-0.8	1.46	-1	0	0	-5	0
	Visit day 6	13 (100%)	0 (0%)	3.7	1.89	3	5	5	0	5	13 (100%)	0 (0%)	-1.3	1.89	-2	0	0	-5	0
	Visit day 7	13 (100%)	0 (0%)	4.2	1.48	4	5	5	0	5	13 (100%)	0 (0%)	-0.8	1.48	-1	0	0	-5	0
	Visit day 8	13 (100%)	0 (0%)	3.8	1.83	4	5	5	0	5	13 (100%)	0 (0%)	-1.2	1.83	-1	0	0	-5	0
	Visit day 9	13 (100%)	0 (0%)	4.2	1.48	4	5	5	1	5	13 (100%)	0 (0%)	-0.8	1.48	-1	0	0	-4	0
	Visit day 10	13 (100%)	0 (0%)	4.2	1.68	4	5	5	0	5	13 (100%)	0 (0%)	-0.8	1.68	-1	0	0	-5	0
	Visit day 11	13 (100%)	0 (0%)	4.4	0.96	4	5	5	2	5	13 (100%)	0 (0%)	-0.6	0.96	-1	0	0	-3	0
	Visit day 12	13 (100%)	0 (0%)	4.3	0.85	4	5	5	3	5	13 (100%)	0 (0%)	-0.7	0.85	-1	0	0	-2	0

		Result									Change from usual*								
**Treatment**	**Visit**	**n**	**nmiss**	**Mean**	**SD**	**Q1**	**Median**	**Q3**	**Min**	**Max**	**n**	**nmiss**	**Mean**	**SD**	**Q1**	**Median**	**Q3**	**Min**	**Max**
	Visit day 13	13 (100%)	0 (0%)	3.8	1.92	3	5	5	0	5	13 (100%)	0 (0%)	-1.2	1.92	-2	0	0	-5	0
	Follow-up day 15	13 (100%)	0 (0%)	3	2	2	4	5	0	5	13 (100%)	0 (0%)	-2	2	-3	-1	0	-5	0
	Follow-up day 45	13 (100%)	0 (0%)	3.7	1.89	2	5	5	0	6	13 (100%)	0 (0%)	-1.3	1.89	-3	0	0	-5	1
NBMI300mg (n=11)	Visit day 2	11 (100%)	0 (0%)	5	0	5	5	5	5	5	11 (100%)	0 (0%)	0	0	0	0	0	0	0
	Visit day 3	11 (100%)	0 (0%)	4.8	0.6	5	5	5	3	5	11 (100%)	0 (0%)	-0.2	0.6	0	0	0	-2	0
	Visit day 4	11 (100%)	0 (0%)	4.5	1.04	5	5	5	2	5	11 (100%)	0 (0%)	-0.5	1.04	0	0	0	-3	0
	Visit day 5	11 (100%)	0 (0%)	4.8	0.4	5	5	5	4	5	11 (100%)	0 (0%)	-0.2	0.4	0	0	0	-1	0
	Visit day 6	11 (100%)	0 (0%)	4.6	1.29	5	5	5	1	6	11 (100%)	0 (0%)	-0.4	1.29	0	0	0	-4	1
	Visit day 7	11 (100%)	0 (0%)	4	1.55	3	5	5	0	5	11 (100%)	0 (0%)	-1	1.55	-2	0	0	-5	0
	Visit day 8	11 (100%)	0 (0%)	3.9	1.3	3	4	5	1	5	11 (100%)	0 (0%)	-1.1	1.3	-2	-1	0	-4	0
	Visit day 9	11 (100%)	0 (0%)	3.9	1.14	3	4	5	2	5	11 (100%)	0 (0%)	-1.1	1.14	-2	-1	0	-3	0
	Visit day 10	11 (100%)	0 (0%)	4.5	1.04	4	5	5	2	5	11 (100%)	0 (0%)	-0.5	1.04	-1	0	0	-3	0
	Visit day 11	11 (100%)	0 (0%)	4	1.61	4	5	5	0	5	11 (100%)	0 (0%)	-1	1.61	-1	0	0	-5	0
	Visit day 12	11 (100%)	0 (0%)	3.8	1.78	3	5	5	0	5	11 (100%)	0 (0%)	-1.2	1.78	-2	0	0	-5	0
	Visit day 13	11 (100%)	0 (0%)	3.4	2.01	1	5	5	0	5	11 (100%)	0 (0%)	-1.6	2.01	-4	0	0	-5	0
	Follow-up day 15	11 (100%)	0 (0%)	2.8	2.04	1	4	5	0	5	11 (100%)	0 (0%)	-2.2	2.04	-4	-1	0	-5	0
	Follow-up day 45	11 (100%)	0 (0%)	2.7	2.05	0	4	4	0	5	11 (100%)	0 (0%)	-2.3	2.05	-5	-1	-1	-5	0
Placebo (n=12)	Visit day 2	12 (100%)	0 (0%)	5	0	5	5	5	5	5	12 (100%)	0 (0%)	0	0	0	0	0	0	0
	Visit day 3	12 (100%)	0 (0%)	4.7	0.78	5	5	5	3	5	12 (100%)	0 (0%)	-0.3	0.78	0	0	0	-2	0
	Visit day 4	12 (100%)	0 (0%)	4.4	1.24	4.5	5	5	1	5	12 (100%)	0 (0%)	-0.6	1.24	-0.5	0	0	-4	0
	Visit day 5	12 (100%)	0 (0%)	4.6	1	5	5	5	2	5	12 (100%)	0 (0%)	-0.4	1	0	0	0	-3	0
	Visit day 6	12 (100%)	0 (0%)	4.5	1	4.5	5	5	2	5	12 (100%)	0 (0%)	-0.5	1	-0.5	0	0	-3	0
	Visit day 7	12 (100%)	0 (0%)	3.9	1.51	2.5	5	5	1	5	12 (100%)	0 (0%)	-1.1	1.51	-2.5	0	0	-4	0
	Visit day 8	12 (100%)	0 (0%)	3.8	1.85	2.5	5	5	0	5	12 (100%)	0 (0%)	-1.2	1.85	-2.5	0	0	-5	0
	Visit day 9	12 (100%)	0 (0%)	4.3	1.14	3	5	5	2	5	12 (100%)	0 (0%)	-0.8	1.14	-2	0	0	-3	0
	Visit day 10	12 (100%)	0 (0%)	4.3	1.23	4	5	5	1	5	12 (100%)	0 (0%)	-0.7	1.23	-1	0	0	-4	0
	Visit day 11	12 (100%)	0 (0%)	4.4	0.9	3.5	5	5	3	5	12 (100%)	0 (0%)	-0.6	0.9	-1.5	0	0	-2	0

		Result									Change from usual*								
**Treatment**	**Visit**	**n**	**nmiss**	**Mean**	**SD**	**Q1**	**Median**	**Q3**	**Min**	**Max**	**n**	**nmiss**	**Mean**	**SD**	**Q1**	**Median**	**Q3**	**Min**	**Max**
	Visit day 12	12 (100%)	0 (0%)	4	1.54	2.5	5	5	1	5	12 (100%)	0 (0%)	-1	1.54	-2.5	0	0	-4	0
	Visit day 13	12 (100%)	0 (0%)	3.9	1.62	3	5	5	0	5	12 (100%)	0 (0%)	-1.1	1.62	-2	0	0	-5	0
	Follow-up day 15	12 (100%)	0 (0%)	4.1	1.56	3	5	5	0	5	12 (100%)	0 (0%)	-0.9	1.56	-2	0	0	-5	0
	Follow-up day 45	12 (100%)	0 (0%)	3.5	1.83	2	4.5	5	0	5	12 (100%)	0 (0%)	-1.5	1.83	-3	-0.5	0	-5	0

Executed script: \\SDS-SERVER2\SAS$\SDS3\Projects\STATISTICS DRUG DEV\NBMI4011 - CTI002\Analyser\Script\SAS\T_fs002.sas, on: 23MAY2016:14:00, by: maria.lidsell
Version of source data: nbmi_ph3.sas7bdat, last modified: 24FEB16:23:13, status: Final
*) Change for physical and mental fatigue score is defined as difference from 1=same as usual.

Table DE7.1.1 Categorical summary of change from usual of Physical and mental fatigue score item - Physical Fatigue Score, tired (Full analysis set)

Treatment	Visit	Result* n	much better than usual	better than usual	same as usual	worse than usual	Change from usual** n	-1	0	1
Total (n=36)	Visit day 2	36 (100%)			36 (100%)		36 (100%)		36 (100%)	
	Visit day 3	36 (100%)	2 (5.56%)	6 (16.67%)	28 (77.78%)		36 (100%)	8 (22.22%)	28 (77.78%)	
	Visit day 4	36 (100%)		8 (22.22%)	28 (77.78%)		36 (100%)	8 (22.22%)	28 (77.78%)	
	Visit day 5	36 (100%)	2 (5.56%)	8 (22.22%)	26 (72.22%)		36 (100%)	10 (27.78%)	26 (72.22%)	
	Visit day 6	36 (100%)	1 (2.78%)	7 (19.44%)	28 (77.78%)		36 (100%)	8 (22.22%)	28 (77.78%)	
	Visit day 7	36 (100%)	1 (2.78%)	9 (25%)	24 (66.67%)	2 (5.56%)	36 (100%)	10 (27.78%)	24 (66.67%)	2 (5.56%)
	Visit day 8	36 (100%)	3 (8.33%)	12 (33.33%)	21 (58.33%)		36 (100%)	15 (41.67%)	21 (58.33%)	
	Visit day 9	36 (100%)	1 (2.78%)	11 (30.56%)	24 (66.67%)		36 (100%)	12 (33.33%)	24 (66.67%)	
	Visit day 10	36 (100%)	3 (8.33%)	10 (27.78%)	23 (63.89%)		36 (100%)	13 (36.11%)	23 (63.89%)	
	Visit day 11	36 (100%)	3 (8.33%)	7 (19.44%)	26 (72.22%)		36 (100%)	10 (27.78%)	26 (72.22%)	
	Visit day 12	36 (100%)	3 (8.33%)	8 (22.22%)	25 (69.44%)		36 (100%)	11 (30.56%)	25 (69.44%)	
	Visit day 13	36 (100%)	8 (22.22%)	9 (25%)	19 (52.78%)		36 (100%)	17 (47.22%)	19 (52.78%)	
	Follow-up day 15	36 (100%)	7 (19.44%)	15 (41.67%)	14 (38.89%)		36 (100%)	22 (61.11%)	14 (38.89%)	
	Follow-up day 45	36 (100%)	5 (13.89%)	15 (41.67%)	16 (44.44%)		36 (100%)	20 (55.56%)	16 (44.44%)	
NBMI100mg (n=13)	Visit day 2	13 (100%)			13 (100%)		13 (100%)		13 (100%)	
	Visit day 3	13 (100%)	1 (7.69%)	2 (15.38%)	10 (76.92%)		13 (100%)	3 (23.08%)	10 (76.92%)	
	Visit day 4	13 (100%)		2 (15.38%)	11 (84.62%)		13 (100%)	2 (15.38%)	11 (84.62%)	
	Visit day 5	13 (100%)	1 (7.69%)	2 (15.38%)	10 (76.92%)		13 (100%)	3 (23.08%)	10 (76.92%)	
	Visit day 6	13 (100%)	1 (7.69%)	1 (7.69%)	11 (84.62%)		13 (100%)	2 (15.38%)	11 (84.62%)	
	Visit day 7	13 (100%)		2 (15.38%)	11 (84.62%)		13 (100%)	2 (15.38%)	11 (84.62%)	
	Visit day 8	13 (100%)	1 (7.69%)	3 (23.08%)	9 (69.23%)		13 (100%)	4 (30.77%)	9 (69.23%)	
	Visit day 9	13 (100%)		2 (15.38%)	11 (84.62%)		13 (100%)	2 (15.38%)	11 (84.62%)	
	Visit day 10	13 (100%)	2 (15.38%)	2 (15.38%)	9 (69.23%)		13 (100%)	4 (30.77%)	9 (69.23%)	
	Visit day 11	13 (100%)	1 (7.69%)	1 (7.69%)	11 (84.62%)		13 (100%)	2 (15.38%)	11 (84.62%)	

		Result*					Change from usual**			
**Treatment**	**Visit**	**n**	**much better than usual**	**better than usual**	**same as usual**	**worse than usual**	**n**	**-1**	**0**	**1**
	Visit day 12	13 (100%)	1 (7.69%)	1 (7.69%)	11 (84.62%)		13 (100%)	2 (15.38%)	11 (84.62%)	
	Visit day 13	13 (100%)	4 (30.77%)	2 (15.38%)	7 (53.85%)		13 (100%)	6 (46.15%)	7 (53.85%)	
	Follow-up day 15	13 (100%)	5 (38.46%)	3 (23.08%)	5 (38.46%)		13 (100%)	8 (61.54%)	5 (38.46%)	
	Follow-up day 45	13 (100%)	2 (15.38%)	4 (30.77%)	7 (53.85%)		13 (100%)	6 (46.15%)	7 (53.85%)	
NBMI300mg (n=11)	Visit day 2	11 (100%)			11 (100%)		11 (100%)		11 (100%)	
	Visit day 3	11 (100%)	1 (9.09%)	2 (18.18%)	8 (72.73%)		11 (100%)	3 (27.27%)	8 (72.73%)	
	Visit day 4	11 (100%)		3 (27.27%)	8 (72.73%)		11 (100%)	3 (27.27%)	8 (72.73%)	
	Visit day 5	11 (100%)		3 (27.27%)	8 (72.73%)		11 (100%)	3 (27.27%)	8 (72.73%)	
	Visit day 6	11 (100%)		1 (9.09%)	10 (90.91%)		11 (100%)	1 (9.09%)	10 (90.91%)	
	Visit day 7	11 (100%)	1 (9.09%)	3 (27.27%)	6 (54.55%)	1 (9.09%)	11 (100%)	4 (36.36%)	6 (54.55%)	1 (9.09%)
	Visit day 8	11 (100%)		5 (45.45%)	6 (54.55%)		11 (100%)	5 (45.45%)	6 (54.55%)	
	Visit day 9	11 (100%)	1 (9.09%)	4 (36.36%)	6 (54.55%)		11 (100%)	5 (45.45%)	6 (54.55%)	
	Visit day 10	11 (100%)	1 (9.09%)	4 (36.36%)	6 (54.55%)		11 (100%)	5 (45.45%)	6 (54.55%)	
	Visit day 11	11 (100%)	2 (18.18%)	3 (27.27%)	6 (54.55%)		11 (100%)	5 (45.45%)	6 (54.55%)	
	Visit day 12	11 (100%)	1 (9.09%)	3 (27.27%)	7 (63.64%)		11 (100%)	4 (36.36%)	7 (63.64%)	
	Visit day 13	11 (100%)	3 (27.27%)	3 (27.27%)	5 (45.45%)		11 (100%)	6 (54.55%)	5 (45.45%)	
	Follow-up day 15	11 (100%)	2 (18.18%)	6 (54.55%)	3 (27.27%)		11 (100%)	8 (72.73%)	3 (27.27%)	
	Follow-up day 45	11 (100%)	1 (9.09%)	7 (63.64%)	3 (27.27%)		11 (100%)	8 (72.73%)	3 (27.27%)	
Placebo (n=12)	Visit day 2	12 (100%)			12 (100%)		12 (100%)		12 (100%)	
	Visit day 3	12 (100%)		2 (16.67%)	10 (83.33%)		12 (100%)	2 (16.67%)	10 (83.33%)	
	Visit day 4	12 (100%)		3 (25%)	9 (75%)		12 (100%)	3 (25%)	9 (75%)	
	Visit day 5	12 (100%)	1 (8.33%)	3 (25%)	8 (66.67%)		12 (100%)	4 (33.33%)	8 (66.67%)	
	Visit day 6	12 (100%)		5 (41.67%)	7 (58.33%)		12 (100%)	5 (41.67%)	7 (58.33%)	
	Visit day 7	12 (100%)		4 (33.33%)	7 (58.33%)	1 (8.33%)	12 (100%)	4 (33.33%)	7 (58.33%)	1 (8.33%)
	Visit day 8	12 (100%)	2 (16.67%)	4 (33.33%)	6 (50%)		12 (100%)	6 (50%)	6 (50%)	
	Visit day 9	12 (100%)		5 (41.67%)	7 (58.33%)		12 (100%)	5 (41.67%)	7 (58.33%)	

Treatment	Visit	Result* n	much better than usual	better than usual	same as usual	worse than usual	Change from usual** n	-1	0	1
	Visit day 10	12 (100%)		4 (33.33%)	8 (66.67%)		12 (100%)	4 (33.33%)	8 (66.67%)	
	Visit day 11	12 (100%)		3 (25%)	9 (75%)		12 (100%)	3 (25%)	9 (75%)	
	Visit day 12	12 (100%)	1 (8.33%)	4 (33.33%)	7 (58.33%)		12 (100%)	5 (41.67%)	7 (58.33%)	
	Visit day 13	12 (100%)	1 (8.33%)	4 (33.33%)	7 (58.33%)		12 (100%)	5 (41.67%)	7 (58.33%)	
	Follow-up day 15	12 (100%)		6 (50%)	6 (50%)		12 (100%)	6 (50%)	6 (50%)	
	Follow-up day 45	12 (100%)	2 (16.67%)	4 (33.33%)	6 (50%)		12 (100%)	6 (50%)	6 (50%)	

Executed script: \\SDS-SERVER2\SAS$\SDS3\Projects\STATISTICS DRUG DEV\NBMI4011 - CTI002\Analyser\Script\SAS\T_fs003.sas, on: 23MAY2016:14:00, by: maria.lidsell
Version of source data: nbmi_ph3.sas7bdat, last modified: 24FEB16:23:13, status: Final
*) Scores are counted according to: much better than usual=0, better than usual=0, same as usual=1, worse than usual=2, much worse than usual=3.
**) Change for physical and mental fatigue score is defined as difference from 1=same as usual.

Table DE7.2.1 Categorical summary of change from usual of Physical and mental fatigue score item - Physical Fatigue Score, need to rest (Full analysis set)

		Result*					Change from usual**			
**Treatment**	**Visit**	**n**	**much better than usual**	**better than usual**	**same as usual**	**worse than usual**	**n**	**-1**	**0**	**1**
Total (n=36)	Visit day 2	36 (100%)		1 (2.78%)	35 (97.22%)		36 (100%)	1 (2.78%)	35 (97.22%)	
	Visit day 3	36 (100%)		7 (19.44%)	28 (77.78%)	1 (2.78%)	36 (100%)	7 (19.44%)	28 (77.78%)	1 (2.78%)
	Visit day 4	36 (100%)		8 (22.22%)	28 (77.78%)		36 (100%)	8 (22.22%)	28 (77.78%)	
	Visit day 5	36 (100%)		5 (13.89%)	31 (86.11%)		36 (100%)	5 (13.89%)	31 (86.11%)	
	Visit day 6	36 (100%)	1 (2.78%)	6 (16.67%)	29 (80.56%)		36 (100%)	7 (19.44%)	29 (80.56%)	
	Visit day 7	36 (100%)	1 (2.78%)	11 (30.56%)	23 (63.89%)	1 (2.78%)	36 (100%)	12 (33.33%)	23 (63.89%)	1 (2.78%)
	Visit day 8	36 (100%)	2 (5.56%)	9 (25%)	25 (69.44%)		36 (100%)	11 (30.56%)	25 (69.44%)	
	Visit day 9	36 (100%)	1 (2.78%)	9 (25%)	25 (69.44%)	1 (2.78%)	36 (100%)	10 (27.78%)	25 (69.44%)	1 (2.78%)
	Visit day 10	36 (100%)	1 (2.78%)	7 (19.44%)	28 (77.78%)		36 (100%)	8 (22.22%)	28 (77.78%)	
	Visit day 11	36 (100%)	2 (5.56%)	5 (13.89%)	29 (80.56%)		36 (100%)	7 (19.44%)	29 (80.56%)	
	Visit day 12	36 (100%)	2 (5.56%)	6 (16.67%)	28 (77.78%)		36 (100%)	8 (22.22%)	28 (77.78%)	
	Visit day 13	36 (100%)	4 (11.11%)	11 (30.56%)	21 (58.33%)		36 (100%)	15 (41.67%)	21 (58.33%)	
	Follow-up day 15	36 (100%)	4 (11.11%)	9 (25%)	23 (63.89%)		36 (100%)	13 (36.11%)	23 (63.89%)	
	Follow-up day 45	36 (100%)	5 (13.89%)	8 (22.22%)	21 (58.33%)	2 (5.56%)	36 (100%)	13 (36.11%)	21 (58.33%)	2 (5.56%)
NBMI100mg (n=13)	Visit day 2	13 (100%)			13 (100%)		13 (100%)		13 (100%)	
	Visit day 3	13 (100%)		3 (23.08%)	10 (76.92%)		13 (100%)	3 (23.08%)	10 (76.92%)	
	Visit day 4	13 (100%)		2 (15.38%)	11 (84.62%)		13 (100%)	2 (15.38%)	11 (84.62%)	
	Visit day 5	13 (100%)		3 (23.08%)	10 (76.92%)		13 (100%)	3 (23.08%)	10 (76.92%)	
	Visit day 6	13 (100%)	1 (7.69%)	2 (15.38%)	10 (76.92%)		13 (100%)	3 (23.08%)	10 (76.92%)	
	Visit day 7	13 (100%)		3 (23.08%)	10 (76.92%)		13 (100%)	3 (23.08%)	10 (76.92%)	
	Visit day 8	13 (100%)	1 (7.69%)	3 (23.08%)	9 (69.23%)		13 (100%)	4 (30.77%)	9 (69.23%)	
	Visit day 9	13 (100%)		3 (23.08%)	9 (69.23%)	1 (7.69%)	13 (100%)	3 (23.08%)	9 (69.23%)	1 (7.69%)
	Visit day 10	13 (100%)	1 (7.69%)	2 (15.38%)	10 (76.92%)		13 (100%)	3 (23.08%)	10 (76.92%)	

		Result*					Change from usual**			
**Treatment**	**Visit**	**n**	**much better than usual**	**better than usual**	**same as usual**	**worse than usual**	**n**	**-1**	**0**	**1**
	Visit day 11	13 (100%)	1 (7.69%)	1 (7.69%)	11 (84.62%)		13 (100%)	2 (15.38%)	11 (84.62%)	
	Visit day 12	13 (100%)	1 (7.69%)	1 (7.69%)	11 (84.62%)		13 (100%)	2 (15.38%)	11 (84.62%)	
	Visit day 13	13 (100%)	1 (7.69%)	3 (23.08%)	9 (69.23%)		13 (100%)	4 (30.77%)	9 (69.23%)	
	Follow-up day 15	13 (100%)	3 (23.08%)	2 (15.38%)	8 (61.54%)		13 (100%)	5 (38.46%)	8 (61.54%)	
	Follow-up day 45	13 (100%)	2 (15.38%)	3 (23.08%)	6 (46.15%)	2 (15.38%)	13 (100%)	5 (38.46%)	6 (46.15%)	2 (15.38%)
NBMI300mg (n=11)	Visit day 2	11 (100%)		1 (9.09%)	10 (90.91%)		11 (100%)	1 (9.09%)	10 (90.91%)	
	Visit day 3	11 (100%)		3 (27.27%)	8 (72.73%)		11 (100%)	3 (27.27%)	8 (72.73%)	
	Visit day 4	11 (100%)		4 (36.36%)	7 (63.64%)		11 (100%)	4 (36.36%)	7 (63.64%)	
	Visit day 5	11 (100%)		1 (9.09%)	10 (90.91%)		11 (100%)	1 (9.09%)	10 (90.91%)	
	Visit day 6	11 (100%)		2 (18.18%)	9 (81.82%)		11 (100%)	2 (18.18%)	9 (81.82%)	
	Visit day 7	11 (100%)	1 (9.09%)	3 (27.27%)	7 (63.64%)		11 (100%)	4 (36.36%)	7 (63.64%)	
	Visit day 8	11 (100%)		3 (27.27%)	8 (72.73%)		11 (100%)	3 (27.27%)	8 (72.73%)	
	Visit day 9	11 (100%)		4 (36.36%)	7 (63.64%)		11 (100%)	4 (36.36%)	7 (63.64%)	
	Visit day 10	11 (100%)		3 (27.27%)	8 (72.73%)		11 (100%)	3 (27.27%)	8 (72.73%)	
	Visit day 11	11 (100%)	1 (9.09%)	3 (27.27%)	7 (63.64%)		11 (100%)	4 (36.36%)	7 (63.64%)	
	Visit day 12	11 (100%)	1 (9.09%)	2 (18.18%)	8 (72.73%)		11 (100%)	3 (27.27%)	8 (72.73%)	
	Visit day 13	11 (100%)	2 (18.18%)	4 (36.36%)	5 (45.45%)		11 (100%)	6 (54.55%)	5 (45.45%)	
	Follow-up day 15	11 (100%)		5 (45.45%)	6 (54.55%)		11 (100%)	5 (45.45%)	6 (54.55%)	
	Follow-up day 45	11 (100%)	2 (18.18%)	4 (36.36%)	5 (45.45%)		11 (100%)	6 (54.55%)	5 (45.45%)	
Placebo (n=12)	Visit day 2	12 (100%)			12 (100%)		12 (100%)		12 (100%)	
	Visit day 3	12 (100%)		1 (8.33%)	10 (83.33%)	1 (8.33%)	12 (100%)	1 (8.33%)	10 (83.33%)	1 (8.33%)
	Visit day 4	12 (100%)		2 (16.67%)	10 (83.33%)		12 (100%)	2 (16.67%)	10 (83.33%)	
	Visit day 5	12 (100%)		1 (8.33%)	11 (91.67%)		12 (100%)	1 (8.33%)	11 (91.67%)	
	Visit day 6	12 (100%)		2 (16.67%)	10 (83.33%)		12 (100%)	2 (16.67%)	10 (83.33%)	
	Visit day 7	12 (100%)		5 (41.67%)	6 (50%)	1 (8.33%)	12 (100%)	5 (41.67%)	6 (50%)	1 (8.33%)
	Visit day 8	12 (100%)	1 (8.33%)	3 (25%)	8 (66.67%)		12 (100%)	4 (33.33%)	8 (66.67%)	

		Result*					Change from usual**			
**Treatment**	**Visit**	**n**	**much better than usual**	**better than usual**	**same as usual**	**worse than usual**	**n**	**-1**	**0**	**1**
	Visit day 9	12 (100%)	1 (8.33%)	2 (16.67%)	9 (75%)		12 (100%)	3 (25%)	9 (75%)	
	Visit day 10	12 (100%)		2 (16.67%)	10 (83.33%)		12 (100%)	2 (16.67%)	10 (83.33%)	
	Visit day 11	12 (100%)		1 (8.33%)	11 (91.67%)		12 (100%)	1 (8.33%)	11 (91.67%)	
	Visit day 12	12 (100%)		3 (25%)	9 (75%)		12 (100%)	3 (25%)	9 (75%)	
	Visit day 13	12 (100%)	1 (8.33%)	4 (33.33%)	7 (58.33%)		12 (100%)	5 (41.67%)	7 (58.33%)	
	Follow-up day 15	12 (100%)	1 (8.33%)	2 (16.67%)	9 (75%)		12 (100%)	3 (25%)	9 (75%)	
	Follow-up day 45	12 (100%)	1 (8.33%)	1 (8.33%)	10 (83.33%)		12 (100%)	2 (16.67%)	10 (83.33%)	

Executed script: \\SDS-SERVER2\SAS$\SDS3\Projects\STATISTICS DRUG DEV\NBMI4011 - CTI002\Analyser\Script\SAS\T_fs003.sas, on: 23MAY2016:14:00, by: maria.lidsell
Version of source data: nbmi_ph3.sas7bdat, last modified: 24FEB16:23:13, status: Final
*) Scores are counted according to: much better than usual=0, better than usual=0, same as usual=1, worse than usual=2, much worse than usual=3.
**) Change for physical and mental fatigue score is defined as difference from 1=same as usual.

Table DE7.3.1 Categorical summary of change from usual of Physical and mental fatigue score item - Physical Fatigue Score, feel sleepy or drowsy (Full analysis set)

		**Result***					**Change from usual****			
**Treatment**	**Visit**	**n**	**much better than usual**	**better than usual**	**same as usual**	**worse than usual**	**n**	**-1**	**0**	**1**
Total (n=36)	Visit day 2	36 (100%)			36 (100%)		36 (100%)		36 (100%)	
	Visit day 3	36 (100%)		4 (11.11%)	31 (86.11%)	1 (2.78%)	36 (100%)	4 (11.11%)	31 (86.11%)	1 (2.78%)
	Visit day 4	36 (100%)		4 (11.11%)	30 (83.33%)	2 (5.56%)	36 (100%)	4 (11.11%)	30 (83.33%)	2 (5.56%)
	Visit day 5	36 (100%)	1 (2.78%)	4 (11.11%)	30 (83.33%)	1 (2.78%)	36 (100%)	5 (13.89%)	30 (83.33%)	1 (2.78%)
	Visit day 6	36 (100%)		7 (19.44%)	29 (80.56%)		36 (100%)	7 (19.44%)	29 (80.56%)	
	Visit day 7	36 (100%)	1 (2.78%)	8 (22.22%)	27 (75%)		36 (100%)	9 (25%)	27 (75%)	
	Visit day 8	36 (100%)	3 (8.33%)	7 (19.44%)	26 (72.22%)		36 (100%)	10 (27.78%)	26 (72.22%)	
	Visit day 9	36 (100%)	2 (5.56%)	7 (19.44%)	26 (72.22%)	1 (2.78%)	36 (100%)	9 (25%)	26 (72.22%)	1 (2.78%)
	Visit day 10	36 (100%)	2 (5.56%)	8 (22.22%)	24 (66.67%)	2 (5.56%)	36 (100%)	10 (27.78%)	24 (66.67%)	2 (5.56%)
	Visit day 11	36 (100%)	4 (11.11%)	8 (22.22%)	24 (66.67%)		36 (100%)	12 (33.33%)	24 (66.67%)	
	Visit day 12	36 (100%)	3 (8.33%)	8 (22.22%)	25 (69.44%)		36 (100%)	11 (30.56%)	25 (69.44%)	
	Visit day 13	36 (100%)	6 (16.67%)	10 (27.78%)	19 (52.78%)	1 (2.78%)	36 (100%)	16 (44.44%)	19 (52.78%)	1 (2.78%)
	Follow-up day 15	36 (100%)	4 (11.11%)	12 (33.33%)	20 (55.56%)		36 (100%)	16 (44.44%)	20 (55.56%)	
	Follow-up day 45	36 (100%)	6 (16.67%)	13 (36.11%)	17 (47.22%)		36 (100%)	19 (52.78%)	17 (47.22%)	
NBMI100mg (n=13)	Visit day 2	13 (100%)			13 (100%)		13 (100%)		13 (100%)	
	Visit day 3	13 (100%)		2 (15.38%)	11 (84.62%)		13 (100%)	2 (15.38%)	11 (84.62%)	
	Visit day 4	13 (100%)		1 (7.69%)	12 (92.31%)		13 (100%)	1 (7.69%)	12 (92.31%)	
	Visit day 5	13 (100%)	1 (7.69%)	1 (7.69%)	10 (76.92%)	1 (7.69%)	13 (100%)	2 (15.38%)	10 (76.92%)	1 (7.69%)
	Visit day 6	13 (100%)		2 (15.38%)	11 (84.62%)		13 (100%)	2 (15.38%)	11 (84.62%)	
	Visit day 7	13 (100%)		2 (15.38%)	11 (84.62%)		13 (100%)	2 (15.38%)	11 (84.62%)	
	Visit day 8	13 (100%)		3 (23.08%)	10 (76.92%)		13 (100%)	3 (23.08%)	10 (76.92%)	
	Visit day 9	13 (100%)	1 (7.69%)	2 (15.38%)	9 (69.23%)	1 (7.69%)	13 (100%)	3 (23.08%)	9 (69.23%)	1 (7.69%)
	Visit day 10	13 (100%)	1 (7.69%)	3 (23.08%)	8 (61.54%)	1 (7.69%)	13 (100%)	4 (30.77%)	8 (61.54%)	1 (7.69%)

Treatment	Visit	Result* n	much better than usual	better than usual	same as usual	worse than usual	Change from usual** n	-1	0	1
	Visit day 11	13 (100%)	2 (15.38%)	2 (15.38%)	9 (69.23%)		13 (100%)	4 (30.77%)	9 (69.23%)	
	Visit day 12	13 (100%)	1 (7.69%)	3 (23.08%)	9 (69.23%)		13 (100%)	4 (30.77%)	9 (69.23%)	
	Visit day 13	13 (100%)	2 (15.38%)	3 (23.08%)	7 (53.85%)	1 (7.69%)	13 (100%)	5 (38.46%)	7 (53.85%)	1 (7.69%)
	Follow-up day 15	13 (100%)	3 (23.08%)	3 (23.08%)	7 (53.85%)		13 (100%)	6 (46.15%)	7 (53.85%)	
	Follow-up day 45	13 (100%)	1 (7.69%)	4 (30.77%)	8 (61.54%)		13 (100%)	5 (38.46%)	8 (61.54%)	
NBMI300mg (n=11)	Visit day 2	11 (100%)			11 (100%)		11 (100%)		11 (100%)	
	Visit day 3	11 (100%)		1 (9.09%)	10 (90.91%)		11 (100%)	1 (9.09%)	10 (90.91%)	
	Visit day 4	11 (100%)		2 (18.18%)	8 (72.73%)	1 (9.09%)	11 (100%)	2 (18.18%)	8 (72.73%)	1 (9.09%)
	Visit day 5	11 (100%)		1 (9.09%)	10 (90.91%)		11 (100%)	1 (9.09%)	10 (90.91%)	
	Visit day 6	11 (100%)		4 (36.36%)	7 (63.64%)		11 (100%)	4 (36.36%)	7 (63.64%)	
	Visit day 7	11 (100%)	1 (9.09%)	4 (36.36%)	6 (54.55%)		11 (100%)	5 (45.45%)	6 (54.55%)	
	Visit day 8	11 (100%)	1 (9.09%)	3 (27.27%)	7 (63.64%)		11 (100%)	4 (36.36%)	7 (63.64%)	
	Visit day 9	11 (100%)	1 (9.09%)	4 (36.36%)	6 (54.55%)		11 (100%)	5 (45.45%)	6 (54.55%)	
	Visit day 10	11 (100%)	1 (9.09%)	4 (36.36%)	5 (45.45%)	1 (9.09%)	11 (100%)	5 (45.45%)	5 (45.45%)	1 (9.09%)
	Visit day 11	11 (100%)	2 (18.18%)	3 (27.27%)	6 (54.55%)		11 (100%)	5 (45.45%)	6 (54.55%)	
	Visit day 12	11 (100%)	2 (18.18%)	2 (18.18%)	7 (63.64%)		11 (100%)	4 (36.36%)	7 (63.64%)	
	Visit day 13	11 (100%)	3 (27.27%)	4 (36.36%)	4 (36.36%)		11 (100%)	7 (63.64%)	4 (36.36%)	
	Follow-up day 15	11 (100%)	1 (9.09%)	6 (54.55%)	4 (36.36%)		11 (100%)	7 (63.64%)	4 (36.36%)	
	Follow-up day 45	11 (100%)	4 (36.36%)	5 (45.45%)	2 (18.18%)		11 (100%)	9 (81.82%)	2 (18.18%)	
Placebo (n=12)	Visit day 2	12 (100%)			12 (100%)		12 (100%)		12 (100%)	
	Visit day 3	12 (100%)		1 (8.33%)	10 (83.33%)	1 (8.33%)	12 (100%)	1 (8.33%)	10 (83.33%)	1 (8.33%)
	Visit day 4	12 (100%)		1 (8.33%)	10 (83.33%)	1 (8.33%)	12 (100%)	1 (8.33%)	10 (83.33%)	1 (8.33%)
	Visit day 5	12 (100%)		2 (16.67%)	10 (83.33%)		12 (100%)	2 (16.67%)	10 (83.33%)	
	Visit day 6	12 (100%)		1 (8.33%)	11 (91.67%)		12 (100%)	1 (8.33%)	11 (91.67%)	
	Visit day 7	12 (100%)		2 (16.67%)	10 (83.33%)		12 (100%)	2 (16.67%)	10 (83.33%)	
	Visit day 8	12 (100%)	2 (16.67%)	1 (8.33%)	9 (75%)		12 (100%)	3 (25%)	9 (75%)	

Treatment	Visit	Result* n	much better than usual	better than usual	same as usual	worse than usual	Change from usual** n	-1	0	1
	Visit day 9	12 (100%)		1 (8.33%)	11 (91.67%)		12 (100%)	1 (8.33%)	11 (91.67%)	
	Visit day 10	12 (100%)		1 (8.33%)	11 (91.67%)		12 (100%)	1 (8.33%)	11 (91.67%)	
	Visit day 11	12 (100%)		3 (25%)	9 (75%)		12 (100%)	3 (25%)	9 (75%)	
	Visit day 12	12 (100%)		3 (25%)	9 (75%)		12 (100%)	3 (25%)	9 (75%)	
	Visit day 13	12 (100%)	1 (8.33%)	3 (25%)	8 (66.67%)		12 (100%)	4 (33.33%)	8 (66.67%)	
	Follow-up day 15	12 (100%)		3 (25%)	9 (75%)		12 (100%)	3 (25%)	9 (75%)	
	Follow-up day 45	12 (100%)	1 (8.33%)	4 (33.33%)	7 (58.33%)		12 (100%)	5 (41.67%)	7 (58.33%)	

Executed script: \\SDS-SERVER2\SAS$\SDS3\Projects\STATISTICS DRUG DEV\NBMI4011 - CTI002\Analyser\Script\SAS\T_fs003.sas, on: 23MAY2016:14:00, by: maria.lidsell
Version of source data: nbmi_ph3.sas7bdat, last modified: 24FEB16:23:13, status: Final
*) Scores are counted according to: much better than usual=0, better than usual=0, same as usual=1, worse than usual=2, much worse than usual=3.
**) Change for physical and mental fatigue score is defined as difference from 1=same as usual.

Table DE7.4.1 Categorical summary of change from usual of Physical and mental fatigue score item - Physical Fatigue Score, no longer start anything (Full analysis set)

		Result*					Change from usual**			
**Treatment**	**Visit**	**n**	**much better than usual**	**better than usual**	**same as usual**	**worse than usual**	**n**	**-1**	**0**	**1**
Total (n=36)	Visit day 2	36 (100%)			36 (100%)		36 (100%)		36 (100%)	
	Visit day 3	36 (100%)		3 (8.33%)	33 (91.67%)		36 (100%)	3 (8.33%)	33 (91.67%)	
	Visit day 4	36 (100%)		2 (5.56%)	34 (94.44%)		36 (100%)	2 (5.56%)	34 (94.44%)	
	Visit day 5	36 (100%)		2 (5.56%)	34 (94.44%)		36 (100%)	2 (5.56%)	34 (94.44%)	
	Visit day 6	36 (100%)		5 (13.89%)	31 (86.11%)		36 (100%)	5 (13.89%)	31 (86.11%)	
	Visit day 7	36 (100%)	1 (2.78%)	5 (13.89%)	30 (83.33%)		36 (100%)	6 (16.67%)	30 (83.33%)	
	Visit day 8	36 (100%)	2 (5.56%)	3 (8.33%)	31 (86.11%)		36 (100%)	5 (13.89%)	31 (86.11%)	
	Visit day 9	36 (100%)		3 (8.33%)	33 (91.67%)		36 (100%)	3 (8.33%)	33 (91.67%)	
	Visit day 10	36 (100%)		5 (13.89%)	31 (86.11%)		36 (100%)	5 (13.89%)	31 (86.11%)	
	Visit day 11	36 (100%)	1 (2.78%)	3 (8.33%)	32 (88.89%)		36 (100%)	4 (11.11%)	32 (88.89%)	
	Visit day 12	36 (100%)	2 (5.56%)		34 (94.44%)		36 (100%)	2 (5.56%)	34 (94.44%)	
	Visit day 13	36 (100%)	4 (11.11%)	5 (13.89%)	27 (75%)		36 (100%)	9 (25%)	27 (75%)	
	Follow-up day 15	36 (100%)	4 (11.11%)	4 (11.11%)	28 (77.78%)		36 (100%)	8 (22.22%)	28 (77.78%)	
	Follow-up day 45	36 (100%)	4 (11.11%)	8 (22.22%)	24 (66.67%)		36 (100%)	12 (33.33%)	24 (66.67%)	
NBMI100mg (n=13)	Visit day 2	13 (100%)			13 (100%)		13 (100%)		13 (100%)	
	Visit day 3	13 (100%)		2 (15.38%)	11 (84.62%)		13 (100%)	2 (15.38%)	11 (84.62%)	
	Visit day 4	13 (100%)			13 (100%)		13 (100%)		13 (100%)	
	Visit day 5	13 (100%)		1 (7.69%)	12 (92.31%)		13 (100%)	1 (7.69%)	12 (92.31%)	
	Visit day 6	13 (100%)		2 (15.38%)	11 (84.62%)		13 (100%)	2 (15.38%)	11 (84.62%)	
	Visit day 7	13 (100%)		3 (23.08%)	10 (76.92%)		13 (100%)	3 (23.08%)	10 (76.92%)	
	Visit day 8	13 (100%)		2 (15.38%)	11 (84.62%)		13 (100%)	2 (15.38%)	11 (84.62%)	
	Visit day 9	13 (100%)			13 (100%)		13 (100%)		13 (100%)	
	Visit day 10	13 (100%)		2 (15.38%)	11 (84.62%)		13 (100%)	2 (15.38%)	11 (84.62%)	

Treatment	Visit	Result* n	much better than usual	better than usual	same as usual	worse than usual	Change from usual** n	-1	0	1
	Visit day 11	13 (100%)			13 (100%)		13 (100%)		13 (100%)	
	Visit day 12	13 (100%)			13 (100%)		13 (100%)		13 (100%)	
	Visit day 13	13 (100%)	1 (7.69%)	1 (7.69%)	11 (84.62%)		13 (100%)	2 (15.38%)	11 (84.62%)	
	Follow-up day 15	13 (100%)	2 (15.38%)	1 (7.69%)	10 (76.92%)		13 (100%)	3 (23.08%)	10 (76.92%)	
	Follow-up day 45	13 (100%)	2 (15.38%)	1 (7.69%)	10 (76.92%)		13 (100%)	3 (23.08%)	10 (76.92%)	
NBMI300mg (n=11)	Visit day 2	11 (100%)			11 (100%)		11 (100%)		11 (100%)	
	Visit day 3	11 (100%)			11 (100%)		11 (100%)		11 (100%)	
	Visit day 4	11 (100%)		2 (18.18%)	9 (81.82%)		11 (100%)	2 (18.18%)	9 (81.82%)	
	Visit day 5	11 (100%)			11 (100%)		11 (100%)		11 (100%)	
	Visit day 6	11 (100%)		1 (9.09%)	10 (90.91%)		11 (100%)	1 (9.09%)	10 (90.91%)	
	Visit day 7	11 (100%)	1 (9.09%)	1 (9.09%)	9 (81.82%)		11 (100%)	2 (18.18%)	9 (81.82%)	
	Visit day 8	11 (100%)			11 (100%)		11 (100%)		11 (100%)	
	Visit day 9	11 (100%)		2 (18.18%)	9 (81.82%)		11 (100%)	2 (18.18%)	9 (81.82%)	
	Visit day 10	11 (100%)		2 (18.18%)	9 (81.82%)		11 (100%)	2 (18.18%)	9 (81.82%)	
	Visit day 11	11 (100%)	1 (9.09%)	2 (18.18%)	8 (72.73%)		11 (100%)	3 (27.27%)	8 (72.73%)	
	Visit day 12	11 (100%)	2 (18.18%)		9 (81.82%)		11 (100%)	2 (18.18%)	9 (81.82%)	
	Visit day 13	11 (100%)	2 (18.18%)	3 (27.27%)	6 (54.55%)		11 (100%)	5 (45.45%)	6 (54.55%)	
	Follow-up day 15	11 (100%)	2 (18.18%)	2 (18.18%)	7 (63.64%)		11 (100%)	4 (36.36%)	7 (63.64%)	
	Follow-up day 45	11 (100%)	1 (9.09%)	5 (45.45%)	5 (45.45%)		11 (100%)	6 (54.55%)	5 (45.45%)	
Placebo (n=12)	Visit day 2	12 (100%)			12 (100%)		12 (100%)		12 (100%)	
	Visit day 3	12 (100%)		1 (8.33%)	11 (91.67%)		12 (100%)	1 (8.33%)	11 (91.67%)	
	Visit day 4	12 (100%)			12 (100%)		12 (100%)		12 (100%)	
	Visit day 5	12 (100%)		1 (8.33%)	11 (91.67%)		12 (100%)	1 (8.33%)	11 (91.67%)	
	Visit day 6	12 (100%)		2 (16.67%)	10 (83.33%)		12 (100%)	2 (16.67%)	10 (83.33%)	
	Visit day 7	12 (100%)		1 (8.33%)	11 (91.67%)		12 (100%)	1 (8.33%)	11 (91.67%)	
	Visit day 8	12 (100%)	2 (16.67%)	1 (8.33%)	9 (75%)		12 (100%)	3 (25%)	9 (75%)	

		Result*					Change from usual**			
**Treatment**	**Visit**	**n**	**much better than usual**	**better than usual**	**same as usual**	**worse than usual**	**n**	**-1**	**0**	**1**
	Visit day 9	12 (100%)		1 (8.33%)	11 (91.67%)		12 (100%)	1 (8.33%)	11 (91.67%)	
	Visit day 10	12 (100%)		1 (8.33%)	11 (91.67%)		12 (100%)	1 (8.33%)	11 (91.67%)	
	Visit day 11	12 (100%)		1 (8.33%)	11 (91.67%)		12 (100%)	1 (8.33%)	11 (91.67%)	
	Visit day 12	12 (100%)			12 (100%)		12 (100%)		12 (100%)	
	Visit day 13	12 (100%)	1 (8.33%)	1 (8.33%)	10 (83.33%)		12 (100%)	2 (16.67%)	10 (83.33%)	
	Follow-up day 15	12 (100%)		1 (8.33%)	11 (91.67%)		12 (100%)	1 (8.33%)	11 (91.67%)	
	Follow-up day 45	12 (100%)	1 (8.33%)	2 (16.67%)	9 (75%)		12 (100%)	3 (25%)	9 (75%)	

Executed script: \\SDS-SERVER2\SAS$\SDS3\Projects\STATISTICS DRUG DEV\NBMI4011 - CTI002\Analyser\Script\SAS\T_fs003.sas, on: 23MAY2016:14:00, by: maria.lidsell
Version of source data: nbmi_ph3.sas7bdat, last modified: 24FEB16:23:13, status: Final
*) Scores are counted according to: much better than usual=0, better than usual=0, same as usual=1, worse than usual=2, much worse than usual=3.
**) Change for physical and mental fatigue score is defined as difference from 1=same as usual.

Table DE7.5.1 Categorical summary of change from usual of Physical and mental fatigue score item - Physical Fatigue Score, always lack energy (Full analysis set)

		Result*					Change from usual**			
**Treatment**	**Visit**	**n**	**much better than usual**	**better than usual**	**same as usual**	**worse than usual**	**n**	**-1**	**0**	**1**
Total (n=36)	Visit day 2	36 (100%)			36 (100%)		36 (100%)		36 (100%)	
	Visit day 3	36 (100%)	1 (2.78%)	11 (30.56%)	24 (66.67%)		36 (100%)	12 (33.33%)	24 (66.67%)	
	Visit day 4	36 (100%)	5 (13.89%)	8 (22.22%)	23 (63.89%)		36 (100%)	13 (36.11%)	23 (63.89%)	
	Visit day 5	36 (100%)	6 (16.67%)	8 (22.22%)	22 (61.11%)		36 (100%)	14 (38.89%)	22 (61.11%)	
	Visit day 6	36 (100%)	7 (19.44%)	10 (27.78%)	19 (52.78%)		36 (100%)	17 (47.22%)	19 (52.78%)	
	Visit day 7	36 (100%)	4 (11.11%)	15 (41.67%)	17 (47.22%)		36 (100%)	19 (52.78%)	17 (47.22%)	
	Visit day 8	36 (100%)	7 (19.44%)	14 (38.89%)	15 (41.67%)		36 (100%)	21 (58.33%)	15 (41.67%)	
	Visit day 9	36 (100%)	6 (16.67%)	15 (41.67%)	14 (38.89%)	1 (2.78%)	36 (100%)	21 (58.33%)	14 (38.89%)	1 (2.78%)
	Visit day 10	36 (100%)	9 (25%)	12 (33.33%)	15 (41.67%)		36 (100%)	21 (58.33%)	15 (41.67%)	
	Visit day 11	36 (100%)	13 (36.11%)	11 (30.56%)	12 (33.33%)		36 (100%)	24 (66.67%)	12 (33.33%)	
	Visit day 12	36 (100%)	11 (30.56%)	9 (25%)	16 (44.44%)		36 (100%)	20 (55.56%)	16 (44.44%)	
	Visit day 13	36 (100%)	13 (36.11%)	12 (33.33%)	11 (30.56%)		36 (100%)	25 (69.44%)	11 (30.56%)	
	Follow-up day 15	36 (100%)	12 (33.33%)	15 (41.67%)	9 (25%)		36 (100%)	27 (75%)	9 (25%)	
	Follow-up day 45	36 (100%)	10 (27.78%)	17 (47.22%)	8 (22.22%)	1 (2.78%)	36 (100%)	27 (75%)	8 (22.22%)	1 (2.78%)
NBMI100mg (n=13)	Visit day 2	13 (100%)			13 (100%)		13 (100%)		13 (100%)	
	Visit day 3	13 (100%)		5 (38.46%)	8 (61.54%)		13 (100%)	5 (38.46%)	8 (61.54%)	
	Visit day 4	13 (100%)	4 (30.77%)	2 (15.38%)	7 (53.85%)		13 (100%)	6 (46.15%)	7 (53.85%)	
	Visit day 5	13 (100%)	3 (23.08%)	3 (23.08%)	7 (53.85%)		13 (100%)	6 (46.15%)	7 (53.85%)	
	Visit day 6	13 (100%)	5 (38.46%)	2 (15.38%)	6 (46.15%)		13 (100%)	7 (53.85%)	6 (46.15%)	
	Visit day 7	13 (100%)	2 (15.38%)	4 (30.77%)	7 (53.85%)		13 (100%)	6 (46.15%)	7 (53.85%)	
	Visit day 8	13 (100%)	3 (23.08%)	3 (23.08%)	7 (53.85%)		13 (100%)	6 (46.15%)	7 (53.85%)	
	Visit day 9	13 (100%)	1 (7.69%)	6 (46.15%)	6 (46.15%)		13 (100%)	7 (53.85%)	6 (46.15%)	
	Visit day 10	13 (100%)	3 (23.08%)	4 (30.77%)	6 (46.15%)		13 (100%)	7 (53.85%)	6 (46.15%)	

Treatment	Visit	Result* n	much better than usual	better than usual	same as usual	worse than usual	Change from usual** n	-1	0	1
	Visit day 11	13 (100%)	6 (46.15%)	2 (15.38%)	5 (38.46%)		13 (100%)	8 (61.54%)	5 (38.46%)	
	Visit day 12	13 (100%)	5 (38.46%)	1 (7.69%)	7 (53.85%)		13 (100%)	6 (46.15%)	7 (53.85%)	
	Visit day 13	13 (100%)	4 (30.77%)	4 (30.77%)	5 (38.46%)		13 (100%)	8 (61.54%)	5 (38.46%)	
	Follow-up day 15	13 (100%)	5 (38.46%)	3 (23.08%)	5 (38.46%)		13 (100%)	8 (61.54%)	5 (38.46%)	
	Follow-up day 45	13 (100%)	6 (46.15%)	5 (38.46%)	1 (7.69%)	1 (7.69%)	13 (100%)	11 (84.62%)	1 (7.69%)	1 (7.69%)
NBMI300mg (n=11)	Visit day 2	11 (100%)			11 (100%)		11 (100%)		11 (100%)	
	Visit day 3	11 (100%)	1 (9.09%)	4 (36.36%)	6 (54.55%)		11 (100%)	5 (45.45%)	6 (54.55%)	
	Visit day 4	11 (100%)		4 (36.36%)	7 (63.64%)		11 (100%)	4 (36.36%)	7 (63.64%)	
	Visit day 5	11 (100%)	2 (18.18%)	3 (27.27%)	6 (54.55%)		11 (100%)	5 (45.45%)	6 (54.55%)	
	Visit day 6	11 (100%)	2 (18.18%)	4 (36.36%)	5 (45.45%)		11 (100%)	6 (54.55%)	5 (45.45%)	
	Visit day 7	11 (100%)	2 (18.18%)	6 (54.55%)	3 (27.27%)		11 (100%)	8 (72.73%)	3 (27.27%)	
	Visit day 8	11 (100%)	2 (18.18%)	6 (54.55%)	3 (27.27%)		11 (100%)	8 (72.73%)	3 (27.27%)	
	Visit day 9	11 (100%)	2 (18.18%)	5 (45.45%)	3 (27.27%)	1 (9.09%)	11 (100%)	7 (63.64%)	3 (27.27%)	1 (9.09%)
	Visit day 10	11 (100%)	5 (45.45%)	2 (18.18%)	4 (36.36%)		11 (100%)	7 (63.64%)	4 (36.36%)	
	Visit day 11	11 (100%)	6 (54.55%)	4 (36.36%)	1 (9.09%)		11 (100%)	10 (90.91%)	1 (9.09%)	
	Visit day 12	11 (100%)	4 (36.36%)	4 (36.36%)	3 (27.27%)		11 (100%)	8 (72.73%)	3 (27.27%)	
	Visit day 13	11 (100%)	6 (54.55%)	4 (36.36%)	1 (9.09%)		11 (100%)	10 (90.91%)	1 (9.09%)	
	Follow-up day 15	11 (100%)	4 (36.36%)	6 (54.55%)	1 (9.09%)		11 (100%)	10 (90.91%)	1 (9.09%)	
	Follow-up day 45	11 (100%)	3 (27.27%)	6 (54.55%)	2 (18.18%)		11 (100%)	9 (81.82%)	2 (18.18%)	
Placebo (n=12)	Visit day 2	12 (100%)			12 (100%)		12 (100%)		12 (100%)	
	Visit day 3	12 (100%)		2 (16.67%)	10 (83.33%)		12 (100%)	2 (16.67%)	10 (83.33%)	
	Visit day 4	12 (100%)	1 (8.33%)	2 (16.67%)	9 (75%)		12 (100%)	3 (25%)	9 (75%)	
	Visit day 5	12 (100%)	1 (8.33%)	2 (16.67%)	9 (75%)		12 (100%)	3 (25%)	9 (75%)	
	Visit day 6	12 (100%)		4 (33.33%)	8 (66.67%)		12 (100%)	4 (33.33%)	8 (66.67%)	
	Visit day 7	12 (100%)		5 (41.67%)	7 (58.33%)		12 (100%)	5 (41.67%)	7 (58.33%)	
	Visit day 8	12 (100%)	2 (16.67%)	5 (41.67%)	5 (41.67%)		12 (100%)	7 (58.33%)	5 (41.67%)	

		Result*					Change from usual**			
**Treatment**	**Visit**	**n**	**much better than usual**	**better than usual**	**same as usual**	**worse than usual**	**n**	**-1**	**0**	**1**
	Visit day 9	12 (100%)	3 (25%)	4 (33.33%)	5 (41.67%)		12 (100%)	7 (58.33%)	5 (41.67%)	
	Visit day 10	12 (100%)	1 (8.33%)	6 (50%)	5 (41.67%)		12 (100%)	7 (58.33%)	5 (41.67%)	
	Visit day 11	12 (100%)	1 (8.33%)	5 (41.67%)	6 (50%)		12 (100%)	6 (50%)	6 (50%)	
	Visit day 12	12 (100%)	2 (16.67%)	4 (33.33%)	6 (50%)		12 (100%)	6 (50%)	6 (50%)	
	Visit day 13	12 (100%)	3 (25%)	4 (33.33%)	5 (41.67%)		12 (100%)	7 (58.33%)	5 (41.67%)	
	Follow-up day 15	12 (100%)	3 (25%)	6 (50%)	3 (25%)		12 (100%)	9 (75%)	3 (25%)	
	Follow-up day 45	12 (100%)	1 (8.33%)	6 (50%)	5 (41.67%)		12 (100%)	7 (58.33%)	5 (41.67%)	

Executed script: \\SDS-SERVER2\SAS$\SDS3\Projects\STATISTICS DRUG DEV\NBMI4011 - CTI002\Analyser\Script\SAS\T_fs003.sas, on: 23MAY2016:14:00, by: maria.lidsell
Version of source data: nbmi_ph3.sas7bdat, last modified: 24FEB16:23:13, status: Final
*) Scores are counted according to: much better than usual=0, better than usual=0, same as usual=1, worse than usual=2, much worse than usual=3.
**) Change for physical and mental fatigue score is defined as difference from 1=same as usual.

Table DE7.6.1 Categorical summary of change from usual of Physical and mental fatigue score item - Physical Fatigue Score, have less strength in your muscles (Full analysis set)

		Result*					Change from usual**			
**Treatment**	**Visit**	**n**	**much better than usual**	**better than usual**	**same as usual**	**worse than usual**	**n**	**-1**	**0**	**1**
Total (n=36)	Visit day 2	36 (100%)			36 (100%)		36 (100%)		36 (100%)	
	Visit day 3	36 (100%)		3 (8.33%)	33 (91.67%)		36 (100%)	3 (8.33%)	33 (91.67%)	
	Visit day 4	36 (100%)	4 (11.11%)	3 (8.33%)	29 (80.56%)		36 (100%)	7 (19.44%)	29 (80.56%)	
	Visit day 5	36 (100%)	5 (13.89%)	8 (22.22%)	23 (63.89%)		36 (100%)	13 (36.11%)	23 (63.89%)	
	Visit day 6	36 (100%)	5 (13.89%)	7 (19.44%)	24 (66.67%)		36 (100%)	12 (33.33%)	24 (66.67%)	
	Visit day 7	36 (100%)	3 (8.33%)	8 (22.22%)	25 (69.44%)		36 (100%)	11 (30.56%)	25 (69.44%)	
	Visit day 8	36 (100%)	5 (13.89%)	8 (22.22%)	23 (63.89%)		36 (100%)	13 (36.11%)	23 (63.89%)	
	Visit day 9	36 (100%)	4 (11.11%)	7 (19.44%)	24 (66.67%)	1 (2.78%)	36 (100%)	11 (30.56%)	24 (66.67%)	1 (2.78%)
	Visit day 10	36 (100%)	4 (11.11%)	13 (36.11%)	19 (52.78%)		36 (100%)	17 (47.22%)	19 (52.78%)	
	Visit day 11	36 (100%)	9 (25%)	10 (27.78%)	17 (47.22%)		36 (100%)	19 (52.78%)	17 (47.22%)	
	Visit day 12	36 (100%)	8 (22.22%)	3 (8.33%)	25 (69.44%)		36 (100%)	11 (30.56%)	25 (69.44%)	
	Visit day 13	36 (100%)	11 (30.56%)	4 (11.11%)	21 (58.33%)		36 (100%)	15 (41.67%)	21 (58.33%)	
	Follow-up day 15	36 (100%)	8 (22.22%)	9 (25%)	19 (52.78%)		36 (100%)	17 (47.22%)	19 (52.78%)	
	Follow-up day 45	36 (100%)	3 (8.33%)	16 (44.44%)	16 (44.44%)	1 (2.78%)	36 (100%)	19 (52.78%)	16 (44.44%)	1 (2.78%)
NBMI100mg (n=13)	Visit day 2	13 (100%)			13 (100%)		13 (100%)		13 (100%)	
	Visit day 3	13 (100%)		2 (15.38%)	11 (84.62%)		13 (100%)	2 (15.38%)	11 (84.62%)	
	Visit day 4	13 (100%)	3 (23.08%)	1 (7.69%)	9 (69.23%)		13 (100%)	4 (30.77%)	9 (69.23%)	
	Visit day 5	13 (100%)	3 (23.08%)	3 (23.08%)	7 (53.85%)		13 (100%)	6 (46.15%)	7 (53.85%)	
	Visit day 6	13 (100%)	4 (30.77%)	2 (15.38%)	7 (53.85%)		13 (100%)	6 (46.15%)	7 (53.85%)	
	Visit day 7	13 (100%)	2 (15.38%)	2 (15.38%)	9 (69.23%)		13 (100%)	4 (30.77%)	9 (69.23%)	
	Visit day 8	13 (100%)	3 (23.08%)	3 (23.08%)	7 (53.85%)		13 (100%)	6 (46.15%)	7 (53.85%)	
	Visit day 9	13 (100%)	1 (7.69%)	5 (38.46%)	7 (53.85%)		13 (100%)	6 (46.15%)	7 (53.85%)	
	Visit day 10	13 (100%)	3 (23.08%)	3 (23.08%)	7 (53.85%)		13 (100%)	6 (46.15%)	7 (53.85%)	

Treatment	Visit	Result* n	much better than usual	better than usual	same as usual	worse than usual	Change from usual** n	-1	0	1
	Visit day 11	13 (100%)	4 (30.77%)	4 (30.77%)	5 (38.46%)		13 (100%)	8 (61.54%)	5 (38.46%)	
	Visit day 12	13 (100%)	5 (38.46%)	1 (7.69%)	7 (53.85%)		13 (100%)	6 (46.15%)	7 (53.85%)	
	Visit day 13	13 (100%)	5 (38.46%)	1 (7.69%)	7 (53.85%)		13 (100%)	6 (46.15%)	7 (53.85%)	
	Follow-up day 15	13 (100%)	6 (46.15%)	1 (7.69%)	6 (46.15%)		13 (100%)	7 (53.85%)	6 (46.15%)	
	Follow-up day 45	13 (100%)	2 (15.38%)	7 (53.85%)	3 (23.08%)	1 (7.69%)	13 (100%)	9 (69.23%)	3 (23.08%)	1 (7.69%)
NBMI300mg (n=11)	Visit day 2	11 (100%)			11 (100%)		11 (100%)		11 (100%)	
	Visit day 3	11 (100%)			11 (100%)		11 (100%)		11 (100%)	
	Visit day 4	11 (100%)		1 (9.09%)	10 (90.91%)		11 (100%)	1 (9.09%)	10 (90.91%)	
	Visit day 5	11 (100%)		4 (36.36%)	7 (63.64%)		11 (100%)	4 (36.36%)	7 (63.64%)	
	Visit day 6	11 (100%)		3 (27.27%)	8 (72.73%)		11 (100%)	3 (27.27%)	8 (72.73%)	
	Visit day 7	11 (100%)	1 (9.09%)	2 (18.18%)	8 (72.73%)		11 (100%)	3 (27.27%)	8 (72.73%)	
	Visit day 8	11 (100%)	1 (9.09%)	4 (36.36%)	6 (54.55%)		11 (100%)	5 (45.45%)	6 (54.55%)	
	Visit day 9	11 (100%)	1 (9.09%)	1 (9.09%)	8 (72.73%)	1 (9.09%)	11 (100%)	2 (18.18%)	8 (72.73%)	1 (9.09%)
	Visit day 10	11 (100%)	1 (9.09%)	5 (45.45%)	5 (45.45%)		11 (100%)	6 (54.55%)	5 (45.45%)	
	Visit day 11	11 (100%)	4 (36.36%)	3 (27.27%)	4 (36.36%)		11 (100%)	7 (63.64%)	4 (36.36%)	
	Visit day 12	11 (100%)	2 (18.18%)	2 (18.18%)	7 (63.64%)		11 (100%)	4 (36.36%)	7 (63.64%)	
	Visit day 13	11 (100%)	4 (36.36%)	3 (27.27%)	4 (36.36%)		11 (100%)	7 (63.64%)	4 (36.36%)	
	Follow-up day 15	11 (100%)	1 (9.09%)	5 (45.45%)	5 (45.45%)		11 (100%)	6 (54.55%)	5 (45.45%)	
	Follow-up day 45	11 (100%)		7 (63.64%)	4 (36.36%)		11 (100%)	7 (63.64%)	4 (36.36%)	
Placebo (n=12)	Visit day 2	12 (100%)			12 (100%)		12 (100%)		12 (100%)	
	Visit day 3	12 (100%)		1 (8.33%)	11 (91.67%)		12 (100%)	1 (8.33%)	11 (91.67%)	
	Visit day 4	12 (100%)	1 (8.33%)	1 (8.33%)	10 (83.33%)		12 (100%)	2 (16.67%)	10 (83.33%)	
	Visit day 5	12 (100%)	2 (16.67%)	1 (8.33%)	9 (75%)		12 (100%)	3 (25%)	9 (75%)	
	Visit day 6	12 (100%)	1 (8.33%)	2 (16.67%)	9 (75%)		12 (100%)	3 (25%)	9 (75%)	
	Visit day 7	12 (100%)		4 (33.33%)	8 (66.67%)		12 (100%)	4 (33.33%)	8 (66.67%)	
	Visit day 8	12 (100%)	1 (8.33%)	1 (8.33%)	10 (83.33%)		12 (100%)	2 (16.67%)	10 (83.33%)	

Treatment	Visit	Result* n	much better than usual	better than usual	same as usual	worse than usual	Change from usual** n	-1	0	1
	Visit day 9	12 (100%)	2 (16.67%)	1 (8.33%)	9 (75%)		12 (100%)	3 (25%)	9 (75%)	
	Visit day 10	12 (100%)		5 (41.67%)	7 (58.33%)		12 (100%)	5 (41.67%)	7 (58.33%)	
	Visit day 11	12 (100%)	1 (8.33%)	3 (25%)	8 (66.67%)		12 (100%)	4 (33.33%)	8 (66.67%)	
	Visit day 12	12 (100%)	1 (8.33%)		11 (91.67%)		12 (100%)	1 (8.33%)	11 (91.67%)	
	Visit day 13	12 (100%)	2 (16.67%)		10 (83.33%)		12 (100%)	2 (16.67%)	10 (83.33%)	
	Follow-up day 15	12 (100%)	1 (8.33%)	3 (25%)	8 (66.67%)		12 (100%)	4 (33.33%)	8 (66.67%)	
	Follow-up day 45	12 (100%)	1 (8.33%)	2 (16.67%)	9 (75%)		12 (100%)	3 (25%)	9 (75%)	

Executed script: \\SDS-SERVER2\SAS$\SDS3\Projects\STATISTICS DRUG DEV\NBMI4011 - CTI002\Analyser\Script\SAS\T_fs003.sas, on: 23MAY2016:14:00, by: maria.lidsell
Version of source data: nbmi_ph3.sas7bdat, last modified: 24FEB16:23:13, status: Final
*) Scores are counted according to: much better than usual=0, better than usual=0, same as usual=1, worse than usual=2, much worse than usual=3.
**) Change for physical and mental fatigue score is defined as difference from 1=same as usual.

Table DE7.7.1 Categorical summary of change from usual of Physical and mental fatigue score item - Physical Fatigue Score, feel weak (Full analysis set)

		**Result***					**Change from usual****			
**Treatment**	**Visit**	**n**	**much better than usual**	**better than usual**	**same as usual**	**worse than usual**	**n**	**-1**	**0**	**1**
Total (n=36)	Visit day 2	36 (100%)			36 (100%)		36 (100%)		36 (100%)	
	Visit day 3	36 (100%)		6 (16.67%)	30 (83.33%)		36 (100%)	6 (16.67%)	30 (83.33%)	
	Visit day 4	36 (100%)		5 (13.89%)	31 (86.11%)		36 (100%)	5 (13.89%)	31 (86.11%)	
	Visit day 5	36 (100%)	2 (5.56%)	6 (16.67%)	28 (77.78%)		36 (100%)	8 (22.22%)	28 (77.78%)	
	Visit day 6	36 (100%)	2 (5.56%)	6 (16.67%)	28 (77.78%)		36 (100%)	8 (22.22%)	28 (77.78%)	
	Visit day 7	36 (100%)	2 (5.56%)	11 (30.56%)	23 (63.89%)		36 (100%)	13 (36.11%)	23 (63.89%)	
	Visit day 8	36 (100%)	4 (11.11%)	15 (41.67%)	17 (47.22%)		36 (100%)	19 (52.78%)	17 (47.22%)	
	Visit day 9	36 (100%)	2 (5.56%)	12 (33.33%)	21 (58.33%)	1 (2.78%)	36 (100%)	14 (38.89%)	21 (58.33%)	1 (2.78%)
	Visit day 10	36 (100%)	4 (11.11%)	9 (25%)	23 (63.89%)		36 (100%)	13 (36.11%)	23 (63.89%)	
	Visit day 11	36 (100%)	5 (13.89%)	10 (27.78%)	21 (58.33%)		36 (100%)	15 (41.67%)	21 (58.33%)	
	Visit day 12	36 (100%)	4 (11.11%)	11 (30.56%)	21 (58.33%)		36 (100%)	15 (41.67%)	21 (58.33%)	
	Visit day 13	36 (100%)	9 (25%)	10 (27.78%)	17 (47.22%)		36 (100%)	19 (52.78%)	17 (47.22%)	
	Follow-up day 15	36 (100%)	3 (8.33%)	16 (44.44%)	17 (47.22%)		36 (100%)	19 (52.78%)	17 (47.22%)	
	Follow-up day 45	36 (100%)	4 (11.11%)	13 (36.11%)	19 (52.78%)		36 (100%)	17 (47.22%)	19 (52.78%)	
NBMI100mg (n=13)	Visit day 2	13 (100%)			13 (100%)		13 (100%)		13 (100%)	
	Visit day 3	13 (100%)		3 (23.08%)	10 (76.92%)		13 (100%)	3 (23.08%)	10 (76.92%)	
	Visit day 4	13 (100%)		1 (7.69%)	12 (92.31%)		13 (100%)	1 (7.69%)	12 (92.31%)	
	Visit day 5	13 (100%)	1 (7.69%)	2 (15.38%)	10 (76.92%)		13 (100%)	3 (23.08%)	10 (76.92%)	
	Visit day 6	13 (100%)	2 (15.38%)	3 (23.08%)	8 (61.54%)		13 (100%)	5 (38.46%)	8 (61.54%)	
	Visit day 7	13 (100%)	1 (7.69%)	4 (30.77%)	8 (61.54%)		13 (100%)	5 (38.46%)	8 (61.54%)	
	Visit day 8	13 (100%)	3 (23.08%)	5 (38.46%)	5 (38.46%)		13 (100%)	8 (61.54%)	5 (38.46%)	
	Visit day 9	13 (100%)	1 (7.69%)	3 (23.08%)	9 (69.23%)		13 (100%)	4 (30.77%)	9 (69.23%)	
	Visit day 10	13 (100%)	3 (23.08%)	2 (15.38%)	8 (61.54%)		13 (100%)	5 (38.46%)	8 (61.54%)	

		Result*					Change from usual**			
Treatment	Visit	n	much better than usual	better than usual	same as usual	worse than usual	n	-1	0	1
	Visit day 11	13 (100%)	2 (15.38%)	2 (15.38%)	9 (69.23%)		13 (100%)	4 (30.77%)	9 (69.23%)	
	Visit day 12	13 (100%)	2 (15.38%)	3 (23.08%)	8 (61.54%)		13 (100%)	5 (38.46%)	8 (61.54%)	
	Visit day 13	13 (100%)	4 (30.77%)	2 (15.38%)	7 (53.85%)		13 (100%)	6 (46.15%)	7 (53.85%)	
	Follow-up day 15	13 (100%)	3 (23.08%)	6 (46.15%)	4 (30.77%)		13 (100%)	9 (69.23%)	4 (30.77%)	
	Follow-up day 45	13 (100%)	2 (15.38%)	7 (53.85%)	4 (30.77%)		13 (100%)	9 (69.23%)	4 (30.77%)	
NBMI300mg (n=11)	Visit day 2	11 (100%)			11 (100%)		11 (100%)		11 (100%)	
	Visit day 3	11 (100%)		1 (9.09%)	10 (90.91%)		11 (100%)	1 (9.09%)	10 (90.91%)	
	Visit day 4	11 (100%)		3 (27.27%)	8 (72.73%)		11 (100%)	3 (27.27%)	8 (72.73%)	
	Visit day 5	11 (100%)	1 (9.09%)	1 (9.09%)	9 (81.82%)		11 (100%)	2 (18.18%)	9 (81.82%)	
	Visit day 6	11 (100%)		2 (18.18%)	9 (81.82%)		11 (100%)	2 (18.18%)	9 (81.82%)	
	Visit day 7	11 (100%)	1 (9.09%)	4 (36.36%)	6 (54.55%)		11 (100%)	5 (45.45%)	6 (54.55%)	
	Visit day 8	11 (100%)	1 (9.09%)	5 (45.45%)	5 (45.45%)		11 (100%)	6 (54.55%)	5 (45.45%)	
	Visit day 9	11 (100%)		4 (36.36%)	6 (54.55%)	1 (9.09%)	11 (100%)	4 (36.36%)	6 (54.55%)	1 (9.09%)
	Visit day 10	11 (100%)	1 (9.09%)	4 (36.36%)	6 (54.55%)		11 (100%)	5 (45.45%)	6 (54.55%)	
	Visit day 11	11 (100%)	3 (27.27%)	4 (36.36%)	4 (36.36%)		11 (100%)	7 (63.64%)	4 (36.36%)	
	Visit day 12	11 (100%)	1 (9.09%)	4 (36.36%)	6 (54.55%)		11 (100%)	5 (45.45%)	6 (54.55%)	
	Visit day 13	11 (100%)	4 (36.36%)	4 (36.36%)	3 (27.27%)		11 (100%)	8 (72.73%)	3 (27.27%)	
	Follow-up day 15	11 (100%)		7 (63.64%)	4 (36.36%)		11 (100%)	7 (63.64%)	4 (36.36%)	
	Follow-up day 45	11 (100%)	1 (9.09%)	5 (45.45%)	5 (45.45%)		11 (100%)	6 (54.55%)	5 (45.45%)	
Placebo (n=12)	Visit day 2	12 (100%)			12 (100%)		12 (100%)		12 (100%)	
	Visit day 3	12 (100%)		2 (16.67%)	10 (83.33%)		12 (100%)	2 (16.67%)	10 (83.33%)	
	Visit day 4	12 (100%)		1 (8.33%)	11 (91.67%)		12 (100%)	1 (8.33%)	11 (91.67%)	
	Visit day 5	12 (100%)		3 (25%)	9 (75%)		12 (100%)	3 (25%)	9 (75%)	
	Visit day 6	12 (100%)		1 (8.33%)	11 (91.67%)		12 (100%)	1 (8.33%)	11 (91.67%)	
	Visit day 7	12 (100%)		3 (25%)	9 (75%)		12 (100%)	3 (25%)	9 (75%)	
	Visit day 8	12 (100%)		5 (41.67%)	7 (58.33%)		12 (100%)	5 (41.67%)	7 (58.33%)	

		Result*					Change from usual**			
Treatment	Visit	n	much better than usual	better than usual	same as usual	worse than usual	n	-1	0	1
	Visit day 9	12 (100%)	1 (8.33%)	5 (41.67%)	6 (50%)		12 (100%)	6 (50%)	6 (50%)	
	Visit day 10	12 (100%)		3 (25%)	9 (75%)		12 (100%)	3 (25%)	9 (75%)	
	Visit day 11	12 (100%)		4 (33.33%)	8 (66.67%)		12 (100%)	4 (33.33%)	8 (66.67%)	
	Visit day 12	12 (100%)	1 (8.33%)	4 (33.33%)	7 (58.33%)		12 (100%)	5 (41.67%)	7 (58.33%)	
	Visit day 13	12 (100%)	1 (8.33%)	4 (33.33%)	7 (58.33%)		12 (100%)	5 (41.67%)	7 (58.33%)	
	Follow-up day 15	12 (100%)		3 (25%)	9 (75%)		12 (100%)	3 (25%)	9 (75%)	
	Follow-up day 45	12 (100%)	1 (8.33%)	1 (8.33%)	10 (83.33%)		12 (100%)	2 (16.67%)	10 (83.33%)	

Executed script: \\SDS-SERVER2\SAS$\SDS3\Projects\STATISTICS DRUG DEV\NBMI4011 - CTI002\Analyser\Script\SAS\T_fs003.sas, on: 23MAY2016:14:00, by: maria.lidsell
Version of source data: nbmi_ph3.sas7bdat, last modified: 24FEB16:23:13, status: Final
*) Scores are counted according to: much better than usual=0, better than usual=0, same as usual=1, worse than usual=2, much worse than usual=3.
**) Change for physical and mental fatigue score is defined as difference from 1=same as usual.

Table DE7.8.1 Categorical summary of change from usual of Physical and mental fatigue score item - Physical Fatigue Score, start things without difficulties, but get weak as you go on (Full analysis set)

		Result*					Change from usual**			
**Treatment**	**Visit**	**n**	**much better than usual**	**better than usual**	**same as usual**	**worse than usual**	**n**	**-1**	**0**	**1**
Total (n=36)	Visit day 2	36 (100%)			36 (100%)		36 (100%)		36 (100%)	
	Visit day 3	36 (100%)		3 (8.33%)	33 (91.67%)		36 (100%)	3 (8.33%)	33 (91.67%)	
	Visit day 4	36 (100%)		2 (5.56%)	34 (94.44%)		36 (100%)	2 (5.56%)	34 (94.44%)	
	Visit day 5	36 (100%)		2 (5.56%)	34 (94.44%)		36 (100%)	2 (5.56%)	34 (94.44%)	
	Visit day 6	36 (100%)		3 (8.33%)	33 (91.67%)		36 (100%)	3 (8.33%)	33 (91.67%)	
	Visit day 7	36 (100%)	1 (2.78%)	3 (8.33%)	32 (88.89%)		36 (100%)	4 (11.11%)	32 (88.89%)	
	Visit day 8	36 (100%)		3 (8.33%)	33 (91.67%)		36 (100%)	3 (8.33%)	33 (91.67%)	
	Visit day 9	36 (100%)		1 (2.78%)	35 (97.22%)		36 (100%)	1 (2.78%)	35 (97.22%)	
	Visit day 10	36 (100%)		3 (8.33%)	33 (91.67%)		36 (100%)	3 (8.33%)	33 (91.67%)	
	Visit day 11	36 (100%)		1 (2.78%)	35 (97.22%)		36 (100%)	1 (2.78%)	35 (97.22%)	
	Visit day 12	36 (100%)	1 (2.78%)	1 (2.78%)	34 (94.44%)		36 (100%)	2 (5.56%)	34 (94.44%)	
	Visit day 13	36 (100%)	5 (13.89%)	3 (8.33%)	28 (77.78%)		36 (100%)	8 (22.22%)	28 (77.78%)	
	Follow-up day 15	36 (100%)	2 (5.56%)	5 (13.89%)	28 (77.78%)	1 (2.78%)	36 (100%)	7 (19.44%)	28 (77.78%)	1 (2.78%)
	Follow-up day 45	36 (100%)	1 (2.78%)	8 (22.22%)	26 (72.22%)	1 (2.78%)	36 (100%)	9 (25%)	26 (72.22%)	1 (2.78%)
NBMI100mg (n=13)	Visit day 2	13 (100%)			13 (100%)		13 (100%)		13 (100%)	
	Visit day 3	13 (100%)		1 (7.69%)	12 (92.31%)		13 (100%)	1 (7.69%)	12 (92.31%)	
	Visit day 4	13 (100%)			13 (100%)		13 (100%)		13 (100%)	
	Visit day 5	13 (100%)		1 (7.69%)	12 (92.31%)		13 (100%)	1 (7.69%)	12 (92.31%)	
	Visit day 6	13 (100%)		2 (15.38%)	11 (84.62%)		13 (100%)	2 (15.38%)	11 (84.62%)	
	Visit day 7	13 (100%)		2 (15.38%)	11 (84.62%)		13 (100%)	2 (15.38%)	11 (84.62%)	
	Visit day 8	13 (100%)		2 (15.38%)	11 (84.62%)		13 (100%)	2 (15.38%)	11 (84.62%)	
	Visit day 9	13 (100%)			13 (100%)		13 (100%)		13 (100%)	
	Visit day 10	13 (100%)		2 (15.38%)	11 (84.62%)		13 (100%)	2 (15.38%)	11 (84.62%)	

Treatment	Visit	Result* n	much better than usual	better than usual	same as usual	worse than usual	Change from usual** n	-1	0	1
	Visit day 11	13 (100%)			13 (100%)		13 (100%)		13 (100%)	
	Visit day 12	13 (100%)			13 (100%)		13 (100%)		13 (100%)	
	Visit day 13	13 (100%)	2 (15.38%)		11 (84.62%)		13 (100%)	2 (15.38%)	11 (84.62%)	
	Follow-up day 15	13 (100%)	2 (15.38%)	1 (7.69%)	10 (76.92%)		13 (100%)	3 (23.08%)	10 (76.92%)	
	Follow-up day 45	13 (100%)	1 (7.69%)	4 (30.77%)	7 (53.85%)	1 (7.69%)	13 (100%)	5 (38.46%)	7 (53.85%)	1 (7.69%)
NBMI300mg (n=11)	Visit day 2	11 (100%)			11 (100%)		11 (100%)		11 (100%)	
	Visit day 3	11 (100%)			11 (100%)		11 (100%)		11 (100%)	
	Visit day 4	11 (100%)		2 (18.18%)	9 (81.82%)		11 (100%)	2 (18.18%)	9 (81.82%)	
	Visit day 5	11 (100%)			11 (100%)		11 (100%)		11 (100%)	
	Visit day 6	11 (100%)		1 (9.09%)	10 (90.91%)		11 (100%)	1 (9.09%)	10 (90.91%)	
	Visit day 7	11 (100%)	1 (9.09%)		10 (90.91%)		11 (100%)	1 (9.09%)	10 (90.91%)	
	Visit day 8	11 (100%)			11 (100%)		11 (100%)		11 (100%)	
	Visit day 9	11 (100%)		1 (9.09%)	10 (90.91%)		11 (100%)	1 (9.09%)	10 (90.91%)	
	Visit day 10	11 (100%)			11 (100%)		11 (100%)		11 (100%)	
	Visit day 11	11 (100%)		1 (9.09%)	10 (90.91%)		11 (100%)	1 (9.09%)	10 (90.91%)	
	Visit day 12	11 (100%)	1 (9.09%)		10 (90.91%)		11 (100%)	1 (9.09%)	10 (90.91%)	
	Visit day 13	11 (100%)	2 (18.18%)	2 (18.18%)	7 (63.64%)		11 (100%)	4 (36.36%)	7 (63.64%)	
	Follow-up day 15	11 (100%)		3 (27.27%)	8 (72.73%)		11 (100%)	3 (27.27%)	8 (72.73%)	
	Follow-up day 45	11 (100%)		3 (27.27%)	8 (72.73%)		11 (100%)	3 (27.27%)	8 (72.73%)	
Placebo (n=12)	Visit day 2	12 (100%)			12 (100%)		12 (100%)		12 (100%)	
	Visit day 3	12 (100%)		2 (16.67%)	10 (83.33%)		12 (100%)	2 (16.67%)	10 (83.33%)	
	Visit day 4	12 (100%)			12 (100%)		12 (100%)		12 (100%)	
	Visit day 5	12 (100%)		1 (8.33%)	11 (91.67%)		12 (100%)	1 (8.33%)	11 (91.67%)	
	Visit day 6	12 (100%)			12 (100%)		12 (100%)		12 (100%)	
	Visit day 7	12 (100%)		1 (8.33%)	11 (91.67%)		12 (100%)	1 (8.33%)	11 (91.67%)	
	Visit day 8	12 (100%)		1 (8.33%)	11 (91.67%)		12 (100%)	1 (8.33%)	11 (91.67%)	

		Result*					Change from usual**			
**Treatment**	**Visit**	**n**	**much better than usual**	**better than usual**	**same as usual**	**worse than usual**	**n**	**-1**	**0**	**1**
	Visit day 9	12 (100%)			12 (100%)		12 (100%)		12 (100%)	
	Visit day 10	12 (100%)		1 (8.33%)	11 (91.67%)		12 (100%)	1 (8.33%)	11 (91.67%)	
	Visit day 11	12 (100%)			12 (100%)		12 (100%)		12 (100%)	
	Visit day 12	12 (100%)		1 (8.33%)	11 (91.67%)		12 (100%)	1 (8.33%)	11 (91.67%)	
	Visit day 13	12 (100%)	1 (8.33%)	1 (8.33%)	10 (83.33%)		12 (100%)	2 (16.67%)	10 (83.33%)	
	Follow-up day 15	12 (100%)		1 (8.33%)	10 (83.33%)	1 (8.33%)	12 (100%)	1 (8.33%)	10 (83.33%)	1 (8.33%)
	Follow-up day 45	12 (100%)		1 (8.33%)	11 (91.67%)		12 (100%)	1 (8.33%)	11 (91.67%)	

Executed script: \\SDS-SERVER2\SAS$\SDS3\Projects\STATISTICS DRUG DEV\NBMI4011 - CTI002\Analyser\Script\SAS\T_fs003.sas, on: 23MAY2016:14:00, by: maria.lidsell
Version of source data: nbmi_ph3.sas7bdat, last modified: 24FEB16:23:13, status: Final
*) Scores are counted according to: much better than usual=0, better than usual=0, same as usual=1, worse than usual=2, much worse than usual=3.
**) Change for physical and mental fatigue score is defined as difference from 1=same as usual.

Table DE7.9.1 Categorical summary of change from usual of Physical and mental fatigue score item - Mental Fatigue Score, problems concentrating (Full analysis set)

		Result*					Change from usual**			
**Treatment**	**Visit**	**n**	**much better than usual**	**better than usual**	**same as usual**	**worse than usual**	**n**	**-1**	**0**	**1**
Total (n=36)	Visit day 2	36 (100%)			36 (100%)		36 (100%)		36 (100%)	
	Visit day 3	36 (100%)		7 (19.44%)	29 (80.56%)		36 (100%)	7 (19.44%)	29 (80.56%)	
	Visit day 4	36 (100%)		5 (13.89%)	31 (86.11%)		36 (100%)	5 (13.89%)	31 (86.11%)	
	Visit day 5	36 (100%)	1 (2.78%)	5 (13.89%)	30 (83.33%)		36 (100%)	6 (16.67%)	30 (83.33%)	
	Visit day 6	36 (100%)		8 (22.22%)	28 (77.78%)		36 (100%)	8 (22.22%)	28 (77.78%)	
	Visit day 7	36 (100%)	1 (2.78%)	10 (27.78%)	25 (69.44%)		36 (100%)	11 (30.56%)	25 (69.44%)	
	Visit day 8	36 (100%)	2 (5.56%)	13 (36.11%)	21 (58.33%)		36 (100%)	15 (41.67%)	21 (58.33%)	
	Visit day 9	36 (100%)		11 (30.56%)	25 (69.44%)		36 (100%)	11 (30.56%)	25 (69.44%)	
	Visit day 10	36 (100%)	2 (5.56%)	6 (16.67%)	28 (77.78%)		36 (100%)	8 (22.22%)	28 (77.78%)	
	Visit day 11	36 (100%)	2 (5.56%)	8 (22.22%)	26 (72.22%)		36 (100%)	10 (27.78%)	26 (72.22%)	
	Visit day 12	36 (100%)	1 (2.78%)	9 (25%)	26 (72.22%)		36 (100%)	10 (27.78%)	26 (72.22%)	
	Visit day 13	36 (100%)	5 (13.89%)	7 (19.44%)	24 (66.67%)		36 (100%)	12 (33.33%)	24 (66.67%)	
	Follow-up day 15	36 (100%)	3 (8.33%)	13 (36.11%)	20 (55.56%)		36 (100%)	16 (44.44%)	20 (55.56%)	
	Follow-up day 45	36 (100%)	3 (8.33%)	13 (36.11%)	20 (55.56%)		36 (100%)	16 (44.44%)	20 (55.56%)	
NBMI100mg (n=13)	Visit day 2	13 (100%)			13 (100%)		13 (100%)		13 (100%)	
	Visit day 3	13 (100%)		4 (30.77%)	9 (69.23%)		13 (100%)	4 (30.77%)	9 (69.23%)	
	Visit day 4	13 (100%)		1 (7.69%)	12 (92.31%)		13 (100%)	1 (7.69%)	12 (92.31%)	
	Visit day 5	13 (100%)		3 (23.08%)	10 (76.92%)		13 (100%)	3 (23.08%)	10 (76.92%)	
	Visit day 6	13 (100%)		4 (30.77%)	9 (69.23%)		13 (100%)	4 (30.77%)	9 (69.23%)	
	Visit day 7	13 (100%)		3 (23.08%)	10 (76.92%)		13 (100%)	3 (23.08%)	10 (76.92%)	
	Visit day 8	13 (100%)		5 (38.46%)	8 (61.54%)		13 (100%)	5 (38.46%)	8 (61.54%)	
	Visit day 9	13 (100%)		2 (15.38%)	11 (84.62%)		13 (100%)	2 (15.38%)	11 (84.62%)	
	Visit day 10	13 (100%)	1 (7.69%)	1 (7.69%)	11 (84.62%)		13 (100%)	2 (15.38%)	11 (84.62%)	

		Result*					Change from usual**			
**Treatment**	**Visit**	**n**	**much better than usual**	**better than usual**	**same as usual**	**worse than usual**	**n**	**-1**	**0**	**1**
	Visit day 11	13 (100%)		3 (23.08%)	10 (76.92%)		13 (100%)	3 (23.08%)	10 (76.92%)	
	Visit day 12	13 (100%)		2 (15.38%)	11 (84.62%)		13 (100%)	2 (15.38%)	11 (84.62%)	
	Visit day 13	13 (100%)	2 (15.38%)	1 (7.69%)	10 (76.92%)		13 (100%)	3 (23.08%)	10 (76.92%)	
	Follow-up day 15	13 (100%)	3 (23.08%)	3 (23.08%)	7 (53.85%)		13 (100%)	6 (46.15%)	7 (53.85%)	
	Follow-up day 45	13 (100%)	1 (7.69%)	4 (30.77%)	8 (61.54%)		13 (100%)	5 (38.46%)	8 (61.54%)	
NBMI300mg (n=11)	Visit day 2	11 (100%)			11 (100%)		11 (100%)		11 (100%)	
	Visit day 3	11 (100%)		1 (9.09%)	10 (90.91%)		11 (100%)	1 (9.09%)	10 (90.91%)	
	Visit day 4	11 (100%)		2 (18.18%)	9 (81.82%)		11 (100%)	2 (18.18%)	9 (81.82%)	
	Visit day 5	11 (100%)	1 (9.09%)		10 (90.91%)		11 (100%)	1 (9.09%)	10 (90.91%)	
	Visit day 6	11 (100%)		1 (9.09%)	10 (90.91%)		11 (100%)	1 (9.09%)	10 (90.91%)	
	Visit day 7	11 (100%)	1 (9.09%)	2 (18.18%)	8 (72.73%)		11 (100%)	3 (27.27%)	8 (72.73%)	
	Visit day 8	11 (100%)		6 (54.55%)	5 (45.45%)		11 (100%)	6 (54.55%)	5 (45.45%)	
	Visit day 9	11 (100%)		6 (54.55%)	5 (45.45%)		11 (100%)	6 (54.55%)	5 (45.45%)	
	Visit day 10	11 (100%)	1 (9.09%)	2 (18.18%)	8 (72.73%)		11 (100%)	3 (27.27%)	8 (72.73%)	
	Visit day 11	11 (100%)	2 (18.18%)	2 (18.18%)	7 (63.64%)		11 (100%)	4 (36.36%)	7 (63.64%)	
	Visit day 12	11 (100%)	1 (9.09%)	3 (27.27%)	7 (63.64%)		11 (100%)	4 (36.36%)	7 (63.64%)	
	Visit day 13	11 (100%)	2 (18.18%)	3 (27.27%)	6 (54.55%)		11 (100%)	5 (45.45%)	6 (54.55%)	
	Follow-up day 15	11 (100%)		7 (63.64%)	4 (36.36%)		11 (100%)	7 (63.64%)	4 (36.36%)	
	Follow-up day 45	11 (100%)	1 (9.09%)	7 (63.64%)	3 (27.27%)		11 (100%)	8 (72.73%)	3 (27.27%)	
Placebo (n=12)	Visit day 2	12 (100%)			12 (100%)		12 (100%)		12 (100%)	
	Visit day 3	12 (100%)		2 (16.67%)	10 (83.33%)		12 (100%)	2 (16.67%)	10 (83.33%)	
	Visit day 4	12 (100%)		2 (16.67%)	10 (83.33%)		12 (100%)	2 (16.67%)	10 (83.33%)	
	Visit day 5	12 (100%)		2 (16.67%)	10 (83.33%)		12 (100%)	2 (16.67%)	10 (83.33%)	
	Visit day 6	12 (100%)		3 (25%)	9 (75%)		12 (100%)	3 (25%)	9 (75%)	
	Visit day 7	12 (100%)		5 (41.67%)	7 (58.33%)		12 (100%)	5 (41.67%)	7 (58.33%)	
	Visit day 8	12 (100%)	2 (16.67%)	2 (16.67%)	8 (66.67%)		12 (100%)	4 (33.33%)	8 (66.67%)	

		Result*					Change from usual**			
Treatment	Visit	n	much better than usual	better than usual	same as usual	worse than usual	n	-1	0	1
	Visit day 9	12 (100%)		3 (25%)	9 (75%)		12 (100%)	3 (25%)	9 (75%)	
	Visit day 10	12 (100%)		3 (25%)	9 (75%)		12 (100%)	3 (25%)	9 (75%)	
	Visit day 11	12 (100%)		3 (25%)	9 (75%)		12 (100%)	3 (25%)	9 (75%)	
	Visit day 12	12 (100%)		4 (33.33%)	8 (66.67%)		12 (100%)	4 (33.33%)	8 (66.67%)	
	Visit day 13	12 (100%)	1 (8.33%)	3 (25%)	8 (66.67%)		12 (100%)	4 (33.33%)	8 (66.67%)	
	Follow-up day 15	12 (100%)		3 (25%)	9 (75%)		12 (100%)	3 (25%)	9 (75%)	
	Follow-up day 45	12 (100%)	1 (8.33%)	2 (16.67%)	9 (75%)		12 (100%)	3 (25%)	9 (75%)	

Executed script: \\SDS-SERVER2\SAS$\SDS3\Projects\STATISTICS DRUG DEV\NBMI4011 - CTI002\Analyser\Script\SAS\T_fs003.sas, on: 23MAY2016:14:00, by: maria.lidsell
Version of source data: nbmi_ph3.sas7bdat, last modified: 24FEB16:23:13, status: Final
*) Scores are counted according to: much better than usual=0, better than usual=0, same as usual=1, worse than usual=2, much worse than usual=3.
**) Change for physical and mental fatigue score is defined as difference from 1=same as usual.

Table DE7.10.1 Categorical summary of change from usual of Physical and mental fatigue score item - Mental Fatigue Score, problems thinking clearly (Full analysis set)

		Result*					Change from usual**			
**Treatment**	**Visit**	**n**	**much better than usual**	**better than usual**	**same as usual**	**worse than usual**	**n**	**-1**	**0**	**1**
Total (n=36)	Visit day 2	36 (100%)			36 (100%)		36 (100%)		36 (100%)	
	Visit day 3	36 (100%)		4 (11.11%)	32 (88.89%)		36 (100%)	4 (11.11%)	32 (88.89%)	
	Visit day 4	36 (100%)		3 (8.33%)	33 (91.67%)		36 (100%)	3 (8.33%)	33 (91.67%)	
	Visit day 5	36 (100%)		3 (8.33%)	33 (91.67%)		36 (100%)	3 (8.33%)	33 (91.67%)	
	Visit day 6	36 (100%)		3 (8.33%)	33 (91.67%)		36 (100%)	3 (8.33%)	33 (91.67%)	
	Visit day 7	36 (100%)	1 (2.78%)	5 (13.89%)	30 (83.33%)		36 (100%)	6 (16.67%)	30 (83.33%)	
	Visit day 8	36 (100%)	2 (5.56%)	6 (16.67%)	28 (77.78%)		36 (100%)	8 (22.22%)	28 (77.78%)	
	Visit day 9	36 (100%)		10 (27.78%)	26 (72.22%)		36 (100%)	10 (27.78%)	26 (72.22%)	
	Visit day 10	36 (100%)		4 (11.11%)	32 (88.89%)		36 (100%)	4 (11.11%)	32 (88.89%)	
	Visit day 11	36 (100%)	1 (2.78%)	2 (5.56%)	33 (91.67%)		36 (100%)	3 (8.33%)	33 (91.67%)	
	Visit day 12	36 (100%)	1 (2.78%)	6 (16.67%)	29 (80.56%)		36 (100%)	7 (19.44%)	29 (80.56%)	
	Visit day 13	36 (100%)	5 (13.89%)	5 (13.89%)	26 (72.22%)		36 (100%)	10 (27.78%)	26 (72.22%)	
	Follow-up day 15	36 (100%)	3 (8.33%)	10 (27.78%)	23 (63.89%)		36 (100%)	13 (36.11%)	23 (63.89%)	
	Follow-up day 45	36 (100%)	4 (11.11%)	8 (22.22%)	24 (66.67%)		36 (100%)	12 (33.33%)	24 (66.67%)	
NBMI100mg (n=13)	Visit day 2	13 (100%)			13 (100%)		13 (100%)		13 (100%)	
	Visit day 3	13 (100%)		2 (15.38%)	11 (84.62%)		13 (100%)	2 (15.38%)	11 (84.62%)	
	Visit day 4	13 (100%)			13 (100%)		13 (100%)		13 (100%)	
	Visit day 5	13 (100%)		2 (15.38%)	11 (84.62%)		13 (100%)	2 (15.38%)	11 (84.62%)	
	Visit day 6	13 (100%)		2 (15.38%)	11 (84.62%)		13 (100%)	2 (15.38%)	11 (84.62%)	
	Visit day 7	13 (100%)		2 (15.38%)	11 (84.62%)		13 (100%)	2 (15.38%)	11 (84.62%)	
	Visit day 8	13 (100%)		2 (15.38%)	11 (84.62%)		13 (100%)	2 (15.38%)	11 (84.62%)	
	Visit day 9	13 (100%)		3 (23.08%)	10 (76.92%)		13 (100%)	3 (23.08%)	10 (76.92%)	
	Visit day 10	13 (100%)		2 (15.38%)	11 (84.62%)		13 (100%)	2 (15.38%)	11 (84.62%)	

		Result*					Change from usual**			
**Treatment**	**Visit**	**n**	**much better than usual**	**better than usual**	**same as usual**	**worse than usual**	**n**	**-1**	**0**	**1**
	Visit day 11	13 (100%)			13 (100%)		13 (100%)		13 (100%)	
	Visit day 12	13 (100%)		1 (7.69%)	12 (92.31%)		13 (100%)	1 (7.69%)	12 (92.31%)	
	Visit day 13	13 (100%)	2 (15.38%)		11 (84.62%)		13 (100%)	2 (15.38%)	11 (84.62%)	
	Follow-up day 15	13 (100%)	2 (15.38%)	3 (23.08%)	8 (61.54%)		13 (100%)	5 (38.46%)	8 (61.54%)	
	Follow-up day 45	13 (100%)	1 (7.69%)	2 (15.38%)	10 (76.92%)		13 (100%)	3 (23.08%)	10 (76.92%)	
NBMI300mg (n=11)	Visit day 2	11 (100%)			11 (100%)		11 (100%)		11 (100%)	
	Visit day 3	11 (100%)		1 (9.09%)	10 (90.91%)		11 (100%)	1 (9.09%)	10 (90.91%)	
	Visit day 4	11 (100%)		2 (18.18%)	9 (81.82%)		11 (100%)	2 (18.18%)	9 (81.82%)	
	Visit day 5	11 (100%)			11 (100%)		11 (100%)		11 (100%)	
	Visit day 6	11 (100%)		1 (9.09%)	10 (90.91%)		11 (100%)	1 (9.09%)	10 (90.91%)	
	Visit day 7	11 (100%)	1 (9.09%)	1 (9.09%)	9 (81.82%)		11 (100%)	2 (18.18%)	9 (81.82%)	
	Visit day 8	11 (100%)		3 (27.27%)	8 (72.73%)		11 (100%)	3 (27.27%)	8 (72.73%)	
	Visit day 9	11 (100%)		4 (36.36%)	7 (63.64%)		11 (100%)	4 (36.36%)	7 (63.64%)	
	Visit day 10	11 (100%)		1 (9.09%)	10 (90.91%)		11 (100%)	1 (9.09%)	10 (90.91%)	
	Visit day 11	11 (100%)	1 (9.09%)	1 (9.09%)	9 (81.82%)		11 (100%)	2 (18.18%)	9 (81.82%)	
	Visit day 12	11 (100%)	1 (9.09%)	2 (18.18%)	8 (72.73%)		11 (100%)	3 (27.27%)	8 (72.73%)	
	Visit day 13	11 (100%)	2 (18.18%)	3 (27.27%)	6 (54.55%)		11 (100%)	5 (45.45%)	6 (54.55%)	
	Follow-up day 15	11 (100%)	1 (9.09%)	4 (36.36%)	6 (54.55%)		11 (100%)	5 (45.45%)	6 (54.55%)	
	Follow-up day 45	11 (100%)	2 (18.18%)	3 (27.27%)	6 (54.55%)		11 (100%)	5 (45.45%)	6 (54.55%)	
Placebo (n=12)	Visit day 2	12 (100%)			12 (100%)		12 (100%)		12 (100%)	
	Visit day 3	12 (100%)		1 (8.33%)	11 (91.67%)		12 (100%)	1 (8.33%)	11 (91.67%)	
	Visit day 4	12 (100%)		1 (8.33%)	11 (91.67%)		12 (100%)	1 (8.33%)	11 (91.67%)	
	Visit day 5	12 (100%)		1 (8.33%)	11 (91.67%)		12 (100%)	1 (8.33%)	11 (91.67%)	
	Visit day 6	12 (100%)			12 (100%)		12 (100%)		12 (100%)	
	Visit day 7	12 (100%)		2 (16.67%)	10 (83.33%)		12 (100%)	2 (16.67%)	10 (83.33%)	
	Visit day 8	12 (100%)	2 (16.67%)	1 (8.33%)	9 (75%)		12 (100%)	3 (25%)	9 (75%)	

		Result*					Change from usual**			
Treatment	Visit	n	much better than usual	better than usual	same as usual	worse than usual	n	-1	0	1
	Visit day 9	12 (100%)		3 (25%)	9 (75%)		12 (100%)	3 (25%)	9 (75%)	
	Visit day 10	12 (100%)		1 (8.33%)	11 (91.67%)		12 (100%)	1 (8.33%)	11 (91.67%)	
	Visit day 11	12 (100%)		1 (8.33%)	11 (91.67%)		12 (100%)	1 (8.33%)	11 (91.67%)	
	Visit day 12	12 (100%)		3 (25%)	9 (75%)		12 (100%)	3 (25%)	9 (75%)	
	Visit day 13	12 (100%)	1 (8.33%)	2 (16.67%)	9 (75%)		12 (100%)	3 (25%)	9 (75%)	
	Follow-up day 15	12 (100%)		3 (25%)	9 (75%)		12 (100%)	3 (25%)	9 (75%)	
	Follow-up day 45	12 (100%)	1 (8.33%)	3 (25%)	8 (66.67%)		12 (100%)	4 (33.33%)	8 (66.67%)	

Executed script: \\SDS-SERVER2\SAS$\SDS3\Projects\STATISTICS DRUG DEV\NBMI4011 - CTI002\Analyser\Script\SAS\T_fs003.sas, on: 23MAY2016:14:00, by: maria.lidsell
Version of source data: nbmi_ph3.sas7bdat, last modified: 24FEB16:23:13, status: Final
*) Scores are counted according to: much better than usual=0, better than usual=0, same as usual=1, worse than usual=2, much worse than usual=3.
**) Change for physical and mental fatigue score is defined as difference from 1=same as usual.

Table DE7.11.1 Categorical summary of change from usual of Physical and mental fatigue score item - Mental Fatigue Score, problems to find correct words when you speak (Full analysis set)

		Result*					Change from usual**			
**Treatment**	**Visit**	**n**	**much better than usual**	**better than usual**	**same as usual**	**worse than usual**	**n**	**-1**	**0**	**1**
Total (n=36)	Visit day 2	36 (100%)			36 (100%)		36 (100%)		36 (100%)	
	Visit day 3	36 (100%)			36 (100%)		36 (100%)		36 (100%)	
	Visit day 4	36 (100%)		1 (2.78%)	35 (97.22%)		36 (100%)	1 (2.78%)	35 (97.22%)	
	Visit day 5	36 (100%)		2 (5.56%)	34 (94.44%)		36 (100%)	2 (5.56%)	34 (94.44%)	
	Visit day 6	36 (100%)		2 (5.56%)	34 (94.44%)		36 (100%)	2 (5.56%)	34 (94.44%)	
	Visit day 7	36 (100%)		4 (11.11%)	32 (88.89%)		36 (100%)	4 (11.11%)	32 (88.89%)	
	Visit day 8	36 (100%)	1 (2.78%)	4 (11.11%)	31 (86.11%)		36 (100%)	5 (13.89%)	31 (86.11%)	
	Visit day 9	36 (100%)			36 (100%)		36 (100%)		36 (100%)	
	Visit day 10	36 (100%)		1 (2.78%)	35 (97.22%)		36 (100%)	1 (2.78%)	35 (97.22%)	
	Visit day 11	36 (100%)		1 (2.78%)	35 (97.22%)		36 (100%)	1 (2.78%)	35 (97.22%)	
	Visit day 12	36 (100%)	1 (2.78%)	1 (2.78%)	34 (94.44%)		36 (100%)	2 (5.56%)	34 (94.44%)	
	Visit day 13	36 (100%)	2 (5.56%)	6 (16.67%)	28 (77.78%)		36 (100%)	8 (22.22%)	28 (77.78%)	
	Follow-up day 15	36 (100%)	3 (8.33%)	5 (13.89%)	28 (77.78%)		36 (100%)	8 (22.22%)	28 (77.78%)	
	Follow-up day 45	36 (100%)	1 (2.78%)	8 (22.22%)	27 (75%)		36 (100%)	9 (25%)	27 (75%)	
NBMI100mg (n=13)	Visit day 2	13 (100%)			13 (100%)		13 (100%)		13 (100%)	
	Visit day 3	13 (100%)			13 (100%)		13 (100%)		13 (100%)	
	Visit day 4	13 (100%)			13 (100%)		13 (100%)		13 (100%)	
	Visit day 5	13 (100%)		2 (15.38%)	11 (84.62%)		13 (100%)	2 (15.38%)	11 (84.62%)	
	Visit day 6	13 (100%)		2 (15.38%)	11 (84.62%)		13 (100%)	2 (15.38%)	11 (84.62%)	
	Visit day 7	13 (100%)		1 (7.69%)	12 (92.31%)		13 (100%)	1 (7.69%)	12 (92.31%)	
	Visit day 8	13 (100%)		2 (15.38%)	11 (84.62%)		13 (100%)	2 (15.38%)	11 (84.62%)	
	Visit day 9	13 (100%)			13 (100%)		13 (100%)		13 (100%)	
	Visit day 10	13 (100%)		1 (7.69%)	12 (92.31%)		13 (100%)	1 (7.69%)	12 (92.31%)	

		Result*					Change from usual**			
**Treatment**	**Visit**	**n**	**much better than usual**	**better than usual**	**same as usual**	**worse than usual**	**n**	**-1**	**0**	**1**
	Visit day 11	13 (100%)			13 (100%)		13 (100%)		13 (100%)	
	Visit day 12	13 (100%)			13 (100%)		13 (100%)		13 (100%)	
	Visit day 13	13 (100%)	1 (7.69%)	2 (15.38%)	10 (76.92%)		13 (100%)	3 (23.08%)	10 (76.92%)	
	Follow-up day 15	13 (100%)	3 (23.08%)	1 (7.69%)	9 (69.23%)		13 (100%)	4 (30.77%)	9 (69.23%)	
	Follow-up day 45	13 (100%)	1 (7.69%)	1 (7.69%)	11 (84.62%)		13 (100%)	2 (15.38%)	11 (84.62%)	
NBMI300mg (n=11)	Visit day 2	11 (100%)			11 (100%)		11 (100%)		11 (100%)	
	Visit day 3	11 (100%)			11 (100%)		11 (100%)		11 (100%)	
	Visit day 4	11 (100%)			11 (100%)		11 (100%)		11 (100%)	
	Visit day 5	11 (100%)			11 (100%)		11 (100%)		11 (100%)	
	Visit day 6	11 (100%)			11 (100%)		11 (100%)		11 (100%)	
	Visit day 7	11 (100%)		2 (18.18%)	9 (81.82%)		11 (100%)	2 (18.18%)	9 (81.82%)	
	Visit day 8	11 (100%)		1 (9.09%)	10 (90.91%)		11 (100%)	1 (9.09%)	10 (90.91%)	
	Visit day 9	11 (100%)			11 (100%)		11 (100%)		11 (100%)	
	Visit day 10	11 (100%)			11 (100%)		11 (100%)		11 (100%)	
	Visit day 11	11 (100%)		1 (9.09%)	10 (90.91%)		11 (100%)	1 (9.09%)	10 (90.91%)	
	Visit day 12	11 (100%)	1 (9.09%)	1 (9.09%)	9 (81.82%)		11 (100%)	2 (18.18%)	9 (81.82%)	
	Visit day 13	11 (100%)	1 (9.09%)	2 (18.18%)	8 (72.73%)		11 (100%)	3 (27.27%)	8 (72.73%)	
	Follow-up day 15	11 (100%)		3 (27.27%)	8 (72.73%)		11 (100%)	3 (27.27%)	8 (72.73%)	
	Follow-up day 45	11 (100%)		4 (36.36%)	7 (63.64%)		11 (100%)	4 (36.36%)	7 (63.64%)	
Placebo (n=12)	Visit day 2	12 (100%)			12 (100%)		12 (100%)		12 (100%)	
	Visit day 3	12 (100%)			12 (100%)		12 (100%)		12 (100%)	
	Visit day 4	12 (100%)		1 (8.33%)	11 (91.67%)		12 (100%)	1 (8.33%)	11 (91.67%)	
	Visit day 5	12 (100%)			12 (100%)		12 (100%)		12 (100%)	
	Visit day 6	12 (100%)			12 (100%)		12 (100%)		12 (100%)	
	Visit day 7	12 (100%)		1 (8.33%)	11 (91.67%)		12 (100%)	1 (8.33%)	11 (91.67%)	
	Visit day 8	12 (100%)	1 (8.33%)	1 (8.33%)	10 (83.33%)		12 (100%)	2 (16.67%)	10 (83.33%)	

		Result*					Change from usual**			
**Treatment**	**Visit**	**n**	**much better than usual**	**better than usual**	**same as usual**	**worse than usual**	**n**	**-1**	**0**	**1**
	Visit day 9	12 (100%)			12 (100%)		12 (100%)		12 (100%)	
	Visit day 10	12 (100%)			12 (100%)		12 (100%)		12 (100%)	
	Visit day 11	12 (100%)			12 (100%)		12 (100%)		12 (100%)	
	Visit day 12	12 (100%)			12 (100%)		12 (100%)		12 (100%)	
	Visit day 13	12 (100%)		2 (16.67%)	10 (83.33%)		12 (100%)	2 (16.67%)	10 (83.33%)	
	Follow-up day 15	12 (100%)		1 (8.33%)	11 (91.67%)		12 (100%)	1 (8.33%)	11 (91.67%)	
	Follow-up day 45	12 (100%)		3 (25%)	9 (75%)		12 (100%)	3 (25%)	9 (75%)	

Executed script: \\SDS-SERVER2\SAS$\SDS3\Projects\STATISTICS DRUG DEV\NBMI4011 - CTI002\Analyser\Script\SAS\T_fs003.sas, on: 23MAY2016:14:00, by: maria.lidsell
Version of source data: nbmi_ph3.sas7bdat, last modified: 24FEB16:23:13, status: Final
*) Scores are counted according to: much better than usual=0, better than usual=0, same as usual=1, worse than usual=2, much worse than usual=3.
**) Change for physical and mental fatigue score is defined as difference from 1=same as usual.

Table DE7.12.1 Categorical summary of change from usual of Physical and mental fatigue score item - Mental Fatigue Score, problems with eyestrain (Full analysis set)

		Result*					Change from usual**			
**Treatment**	**Visit**	**n**	**much better than usual**	**better than usual**	**same as usual**	**worse than usual**	**n**	**-1**	**0**	**1**
Total (n=36)	Visit day 2	36 (100%)			36 (100%)		36 (100%)		36 (100%)	
	Visit day 3	36 (100%)		1 (2.78%)	35 (97.22%)		36 (100%)	1 (2.78%)	35 (97.22%)	
	Visit day 4	36 (100%)		1 (2.78%)	35 (97.22%)		36 (100%)	1 (2.78%)	35 (97.22%)	
	Visit day 5	36 (100%)	3 (8.33%)	1 (2.78%)	31 (86.11%)	1 (2.78%)	36 (100%)	4 (11.11%)	31 (86.11%)	1 (2.78%)
	Visit day 6	36 (100%)	4 (11.11%)	4 (11.11%)	27 (75%)	1 (2.78%)	36 (100%)	8 (22.22%)	27 (75%)	1 (2.78%)
	Visit day 7	36 (100%)	3 (8.33%)	3 (8.33%)	29 (80.56%)	1 (2.78%)	36 (100%)	6 (16.67%)	29 (80.56%)	1 (2.78%)
	Visit day 8	36 (100%)	4 (11.11%)	3 (8.33%)	28 (77.78%)	1 (2.78%)	36 (100%)	7 (19.44%)	28 (77.78%)	1 (2.78%)
	Visit day 9	36 (100%)	1 (2.78%)	2 (5.56%)	33 (91.67%)		36 (100%)	3 (8.33%)	33 (91.67%)	
	Visit day 10	36 (100%)	3 (8.33%)	3 (8.33%)	30 (83.33%)		36 (100%)	6 (16.67%)	30 (83.33%)	
	Visit day 11	36 (100%)	2 (5.56%)	3 (8.33%)	31 (86.11%)		36 (100%)	5 (13.89%)	31 (86.11%)	
	Visit day 12	36 (100%)	3 (8.33%)	4 (11.11%)	29 (80.56%)		36 (100%)	7 (19.44%)	29 (80.56%)	
	Visit day 13	36 (100%)	4 (11.11%)	4 (11.11%)	28 (77.78%)		36 (100%)	8 (22.22%)	28 (77.78%)	
	Follow-up day 15	36 (100%)	5 (13.89%)	7 (19.44%)	24 (66.67%)		36 (100%)	12 (33.33%)	24 (66.67%)	
	Follow-up day 45	36 (100%)	4 (11.11%)	7 (19.44%)	24 (66.67%)	1 (2.78%)	36 (100%)	11 (30.56%)	24 (66.67%)	1 (2.78%)
NBMI100mg (n=13)	Visit day 2	13 (100%)			13 (100%)		13 (100%)		13 (100%)	
	Visit day 3	13 (100%)		1 (7.69%)	12 (92.31%)		13 (100%)	1 (7.69%)	12 (92.31%)	
	Visit day 4	13 (100%)			13 (100%)		13 (100%)		13 (100%)	
	Visit day 5	13 (100%)	2 (15.38%)		10 (76.92%)	1 (7.69%)	13 (100%)	2 (15.38%)	10 (76.92%)	1 (7.69%)
	Visit day 6	13 (100%)	4 (30.77%)	1 (7.69%)	8 (61.54%)		13 (100%)	5 (38.46%)	8 (61.54%)	
	Visit day 7	13 (100%)	2 (15.38%)	1 (7.69%)	9 (69.23%)	1 (7.69%)	13 (100%)	3 (23.08%)	9 (69.23%)	1 (7.69%)
	Visit day 8	13 (100%)	3 (23.08%)	2 (15.38%)	7 (53.85%)	1 (7.69%)	13 (100%)	5 (38.46%)	7 (53.85%)	1 (7.69%)
	Visit day 9	13 (100%)	1 (7.69%)	2 (15.38%)	10 (76.92%)		13 (100%)	3 (23.08%)	10 (76.92%)	
	Visit day 10	13 (100%)	2 (15.38%)	2 (15.38%)	9 (69.23%)		13 (100%)	4 (30.77%)	9 (69.23%)	

		Result*					Change from usual**			
**Treatment**	**Visit**	**n**	**much better than usual**	**better than usual**	**same as usual**	**worse than usual**	**n**	**-1**	**0**	**1**
	Visit day 11	13 (100%)	2 (15.38%)	2 (15.38%)	9 (69.23%)		13 (100%)	4 (30.77%)	9 (69.23%)	
	Visit day 12	13 (100%)	2 (15.38%)	2 (15.38%)	9 (69.23%)		13 (100%)	4 (30.77%)	9 (69.23%)	
	Visit day 13	13 (100%)	3 (23.08%)	2 (15.38%)	8 (61.54%)		13 (100%)	5 (38.46%)	8 (61.54%)	
	Follow-up day 15	13 (100%)	4 (30.77%)	2 (15.38%)	7 (53.85%)		13 (100%)	6 (46.15%)	7 (53.85%)	
	Follow-up day 45	13 (100%)	2 (15.38%)	3 (23.08%)	7 (53.85%)	1 (7.69%)	13 (100%)	5 (38.46%)	7 (53.85%)	1 (7.69%)
NBMI300mg (n=11)	Visit day 2	11 (100%)			11 (100%)		11 (100%)		11 (100%)	
	Visit day 3	11 (100%)			11 (100%)		11 (100%)		11 (100%)	
	Visit day 4	11 (100%)			11 (100%)		11 (100%)		11 (100%)	
	Visit day 5	11 (100%)		1 (9.09%)	10 (90.91%)		11 (100%)	1 (9.09%)	10 (90.91%)	
	Visit day 6	11 (100%)		2 (18.18%)	8 (72.73%)	1 (9.09%)	11 (100%)	2 (18.18%)	8 (72.73%)	1 (9.09%)
	Visit day 7	11 (100%)	1 (9.09%)	1 (9.09%)	9 (81.82%)		11 (100%)	2 (18.18%)	9 (81.82%)	
	Visit day 8	11 (100%)			11 (100%)		11 (100%)		11 (100%)	
	Visit day 9	11 (100%)			11 (100%)		11 (100%)		11 (100%)	
	Visit day 10	11 (100%)			11 (100%)		11 (100%)		11 (100%)	
	Visit day 11	11 (100%)		1 (9.09%)	10 (90.91%)		11 (100%)	1 (9.09%)	10 (90.91%)	
	Visit day 12	11 (100%)	1 (9.09%)		10 (90.91%)		11 (100%)	1 (9.09%)	10 (90.91%)	
	Visit day 13	11 (100%)	1 (9.09%)		10 (90.91%)		11 (100%)	1 (9.09%)	10 (90.91%)	
	Follow-up day 15	11 (100%)	1 (9.09%)	4 (36.36%)	6 (54.55%)		11 (100%)	5 (45.45%)	6 (54.55%)	
	Follow-up day 45	11 (100%)	2 (18.18%)	2 (18.18%)	7 (63.64%)		11 (100%)	4 (36.36%)	7 (63.64%)	
Placebo (n=12)	Visit day 2	12 (100%)			12 (100%)		12 (100%)		12 (100%)	
	Visit day 3	12 (100%)			12 (100%)		12 (100%)		12 (100%)	
	Visit day 4	12 (100%)		1 (8.33%)	11 (91.67%)		12 (100%)	1 (8.33%)	11 (91.67%)	
	Visit day 5	12 (100%)	1 (8.33%)		11 (91.67%)		12 (100%)	1 (8.33%)	11 (91.67%)	
	Visit day 6	12 (100%)		1 (8.33%)	11 (91.67%)		12 (100%)	1 (8.33%)	11 (91.67%)	
	Visit day 7	12 (100%)		1 (8.33%)	11 (91.67%)		12 (100%)	1 (8.33%)	11 (91.67%)	
	Visit day 8	12 (100%)	1 (8.33%)	1 (8.33%)	10 (83.33%)		12 (100%)	2 (16.67%)	10 (83.33%)	

		Result*					Change from usual**			
**Treatment**	**Visit**	**n**	**much better than usual**	**better than usual**	**same as usual**	**worse than usual**	**n**	**-1**	**0**	**1**
	Visit day 9	12 (100%)			12 (100%)		12 (100%)		12 (100%)	
	Visit day 10	12 (100%)	1 (8.33%)	1 (8.33%)	10 (83.33%)		12 (100%)	2 (16.67%)	10 (83.33%)	
	Visit day 11	12 (100%)			12 (100%)		12 (100%)		12 (100%)	
	Visit day 12	12 (100%)		2 (16.67%)	10 (83.33%)		12 (100%)	2 (16.67%)	10 (83.33%)	
	Visit day 13	12 (100%)		2 (16.67%)	10 (83.33%)		12 (100%)	2 (16.67%)	10 (83.33%)	
	Follow-up day 15	12 (100%)		1 (8.33%)	11 (91.67%)		12 (100%)	1 (8.33%)	11 (91.67%)	
	Follow-up day 45	12 (100%)		2 (16.67%)	10 (83.33%)		12 (100%)	2 (16.67%)	10 (83.33%)	

Executed script: \\SDS-SERVER2\SAS$\SDS3\Projects\STATISTICS DRUG DEV\NBMI4011 - CTI002\Analyser\Script\SAS\T_fs003.sas, on: 23MAY2016:14:00, by: maria.lidsell
Version of source data: nbmi_ph3.sas7bdat, last modified: 24FEB16:23:13, status: Final
*) Scores are counted according to: much better than usual=0, better than usual=0, same as usual=1, worse than usual=2, much worse than usual=3.
**) Change for physical and mental fatigue score is defined as difference from 1=same as usual.

Table DE7.13.1 Categorical summary of change from usual of Physical and mental fatigue score item - Mental Fatigue Score, problems with memory (Full analysis set)

Treatment	Visit	Result* n	much better than usual	better than usual	same as usual	worse than usual	Change from usual** n	-1	0	1
Total (n=36)	Visit day 2	36 (100%)			36 (100%)		36 (100%)		36 (100%)	
	Visit day 3	36 (100%)		4 (11.11%)	32 (88.89%)		36 (100%)	4 (11.11%)	32 (88.89%)	
	Visit day 4	36 (100%)		5 (13.89%)	31 (86.11%)		36 (100%)	5 (13.89%)	31 (86.11%)	
	Visit day 5	36 (100%)		4 (11.11%)	32 (88.89%)		36 (100%)	4 (11.11%)	32 (88.89%)	
	Visit day 6	36 (100%)	1 (2.78%)	6 (16.67%)	29 (80.56%)		36 (100%)	7 (19.44%)	29 (80.56%)	
	Visit day 7	36 (100%)	1 (2.78%)	7 (19.44%)	28 (77.78%)		36 (100%)	8 (22.22%)	28 (77.78%)	
	Visit day 8	36 (100%)	1 (2.78%)	7 (19.44%)	28 (77.78%)		36 (100%)	8 (22.22%)	28 (77.78%)	
	Visit day 9	36 (100%)		7 (19.44%)	29 (80.56%)		36 (100%)	7 (19.44%)	29 (80.56%)	
	Visit day 10	36 (100%)		6 (16.67%)	30 (83.33%)		36 (100%)	6 (16.67%)	30 (83.33%)	
	Visit day 11	36 (100%)	1 (2.78%)	6 (16.67%)	29 (80.56%)		36 (100%)	7 (19.44%)	29 (80.56%)	
	Visit day 12	36 (100%)	1 (2.78%)	7 (19.44%)	28 (77.78%)		36 (100%)	8 (22.22%)	28 (77.78%)	
	Visit day 13	36 (100%)	2 (5.56%)	7 (19.44%)	27 (75%)		36 (100%)	9 (25%)	27 (75%)	
	Follow-up day 15	36 (100%)	2 (5.56%)	10 (27.78%)	24 (66.67%)		36 (100%)	12 (33.33%)	24 (66.67%)	
	Follow-up day 45	36 (100%)	3 (8.33%)	10 (27.78%)	23 (63.89%)		36 (100%)	13 (36.11%)	23 (63.89%)	
NBMI100mg (n=13)	Visit day 2	13 (100%)			13 (100%)		13 (100%)		13 (100%)	
	Visit day 3	13 (100%)		3 (23.08%)	10 (76.92%)		13 (100%)	3 (23.08%)	10 (76.92%)	
	Visit day 4	13 (100%)		2 (15.38%)	11 (84.62%)		13 (100%)	2 (15.38%)	11 (84.62%)	
	Visit day 5	13 (100%)		3 (23.08%)	10 (76.92%)		13 (100%)	3 (23.08%)	10 (76.92%)	
	Visit day 6	13 (100%)	1 (7.69%)	3 (23.08%)	9 (69.23%)		13 (100%)	4 (30.77%)	9 (69.23%)	
	Visit day 7	13 (100%)		2 (15.38%)	11 (84.62%)		13 (100%)	2 (15.38%)	11 (84.62%)	
	Visit day 8	13 (100%)		3 (23.08%)	10 (76.92%)		13 (100%)	3 (23.08%)	10 (76.92%)	
	Visit day 9	13 (100%)		2 (15.38%)	11 (84.62%)		13 (100%)	2 (15.38%)	11 (84.62%)	
	Visit day 10	13 (100%)		2 (15.38%)	11 (84.62%)		13 (100%)	2 (15.38%)	11 (84.62%)	

		Result*					Change from usual**			
**Treatment**	**Visit**	**n**	**much better than usual**	**better than usual**	**same as usual**	**worse than usual**	**n**	**-1**	**0**	**1**
	Visit day 11	13 (100%)		1 (7.69%)	12 (92.31%)		13 (100%)	1 (7.69%)	12 (92.31%)	
	Visit day 12	13 (100%)		2 (15.38%)	11 (84.62%)		13 (100%)	2 (15.38%)	11 (84.62%)	
	Visit day 13	13 (100%)	1 (7.69%)	2 (15.38%)	10 (76.92%)		13 (100%)	3 (23.08%)	10 (76.92%)	
	Follow-up day 15	13 (100%)	2 (15.38%)	3 (23.08%)	8 (61.54%)		13 (100%)	5 (38.46%)	8 (61.54%)	
	Follow-up day 45	13 (100%)	1 (7.69%)	2 (15.38%)	10 (76.92%)		13 (100%)	3 (23.08%)	10 (76.92%)	
NBMI300mg (n=11)	Visit day 2	11 (100%)			11 (100%)		11 (100%)		11 (100%)	
	Visit day 3	11 (100%)			11 (100%)		11 (100%)		11 (100%)	
	Visit day 4	11 (100%)		1 (9.09%)	10 (90.91%)		11 (100%)	1 (9.09%)	10 (90.91%)	
	Visit day 5	11 (100%)			11 (100%)		11 (100%)		11 (100%)	
	Visit day 6	11 (100%)		1 (9.09%)	10 (90.91%)		11 (100%)	1 (9.09%)	10 (90.91%)	
	Visit day 7	11 (100%)	1 (9.09%)	1 (9.09%)	9 (81.82%)		11 (100%)	2 (18.18%)	9 (81.82%)	
	Visit day 8	11 (100%)		2 (18.18%)	9 (81.82%)		11 (100%)	2 (18.18%)	9 (81.82%)	
	Visit day 9	11 (100%)		2 (18.18%)	9 (81.82%)		11 (100%)	2 (18.18%)	9 (81.82%)	
	Visit day 10	11 (100%)		2 (18.18%)	9 (81.82%)		11 (100%)	2 (18.18%)	9 (81.82%)	
	Visit day 11	11 (100%)	1 (9.09%)	2 (18.18%)	8 (72.73%)		11 (100%)	3 (27.27%)	8 (72.73%)	
	Visit day 12	11 (100%)	1 (9.09%)	2 (18.18%)	8 (72.73%)		11 (100%)	3 (27.27%)	8 (72.73%)	
	Visit day 13	11 (100%)		4 (36.36%)	7 (63.64%)		11 (100%)	4 (36.36%)	7 (63.64%)	
	Follow-up day 15	11 (100%)		4 (36.36%)	7 (63.64%)		11 (100%)	4 (36.36%)	7 (63.64%)	
	Follow-up day 45	11 (100%)	1 (9.09%)	3 (27.27%)	7 (63.64%)		11 (100%)	4 (36.36%)	7 (63.64%)	
Placebo (n=12)	Visit day 2	12 (100%)			12 (100%)		12 (100%)		12 (100%)	
	Visit day 3	12 (100%)		1 (8.33%)	11 (91.67%)		12 (100%)	1 (8.33%)	11 (91.67%)	
	Visit day 4	12 (100%)		2 (16.67%)	10 (83.33%)		12 (100%)	2 (16.67%)	10 (83.33%)	
	Visit day 5	12 (100%)		1 (8.33%)	11 (91.67%)		12 (100%)	1 (8.33%)	11 (91.67%)	
	Visit day 6	12 (100%)		2 (16.67%)	10 (83.33%)		12 (100%)	2 (16.67%)	10 (83.33%)	
	Visit day 7	12 (100%)		4 (33.33%)	8 (66.67%)		12 (100%)	4 (33.33%)	8 (66.67%)	
	Visit day 8	12 (100%)	1 (8.33%)	2 (16.67%)	9 (75%)		12 (100%)	3 (25%)	9 (75%)	

Treatment	Visit	Result* n	much better than usual	better than usual	same as usual	worse than usual	Change from usual** n	-1	0	1
	Visit day 9	12 (100%)		3 (25%)	9 (75%)		12 (100%)	3 (25%)	9 (75%)	
	Visit day 10	12 (100%)		2 (16.67%)	10 (83.33%)		12 (100%)	2 (16.67%)	10 (83.33%)	
	Visit day 11	12 (100%)		3 (25%)	9 (75%)		12 (100%)	3 (25%)	9 (75%)	
	Visit day 12	12 (100%)		3 (25%)	9 (75%)		12 (100%)	3 (25%)	9 (75%)	
	Visit day 13	12 (100%)	1 (8.33%)	1 (8.33%)	10 (83.33%)		12 (100%)	2 (16.67%)	10 (83.33%)	
	Follow-up day 15	12 (100%)		3 (25%)	9 (75%)		12 (100%)	3 (25%)	9 (75%)	
	Follow-up day 45	12 (100%)	1 (8.33%)	5 (41.67%)	6 (50%)		12 (100%)	6 (50%)	6 (50%)	

Executed script: \\SDS-SERVER2\SAS$\SDS3\Projects\STATISTICS DRUG DEV\NBMI4011 - CTI002\Analyser\Script\SAS\T_fs003.sas, on: 23MAY2016:14:00, by: maria.lidsell
Version of source data: nbmi_ph3.sas7bdat, last modified: 24FEB16:23:13, status: Final
*) Scores are counted according to: much better than usual=0, better than usual=0, same as usual=1, worse than usual=2, much worse than usual=3.
**) Change for physical and mental fatigue score is defined as difference from 1=same as usual.

Table DE8.1 Categorical summary of change from baseline of finger to nose test (Full analysis set)

		**Result***				**Change** from baseline (Visit day 1)**				
**Treatment**	**Visit**	**n**	**ABSENT**	**SLIGHT**	**MODERATE**	**n**	**-1**	**0**	**1**	**2**
Total (n=36)	Visit day 1	36 (100%)	22 (61.11%)	14 (38.89%)						
	Follow-up day 15	36 (100%)	20 (55.56%)	15 (41.67%)	1 (2.78%)	36 (100%)	5 (13.89%)	23 (63.89%)	8 (22.22%)	
	Follow-up day 45	36 (100%)	15 (41.67%)	20 (55.56%)	1 (2.78%)	36 (100%)	5 (13.89%)	19 (52.78%)	11 (30.56%)	1 (2.78%)
NBMI100mg (n=13)	Visit day 1	13 (100%)	5 (38.46%)	8 (61.54%)						
	Follow-up day 15	13 (100%)	4 (30.77%)	8 (61.54%)	1 (7.69%)	13 (100%)	2 (15.38%)	7 (53.85%)	4 (30.77%)	
	Follow-up day 45	13 (100%)	5 (38.46%)	8 (61.54%)		13 (100%)	2 (15.38%)	9 (69.23%)	2 (15.38%)	
NBMI300mg (n=11)	Visit day 1	11 (100%)	8 (72.73%)	3 (27.27%)						
	Follow-up day 15	11 (100%)	8 (72.73%)	3 (27.27%)		11 (100%)	1 (9.09%)	9 (81.82%)	1 (9.09%)	
	Follow-up day 45	11 (100%)	4 (36.36%)	6 (54.55%)	1 (9.09%)	11 (100%)		7 (63.64%)	3 (27.27%)	1 (9.09%)
Placebo (n=12)	Visit day 1	12 (100%)	9 (75%)	3 (25%)						
	Follow-up day 15	12 (100%)	8 (66.67%)	4 (33.33%)		12 (100%)	2 (16.67%)	7 (58.33%)	3 (25%)	
	Follow-up day 45	12 (100%)	6 (50%)	6 (50%)		12 (100%)	3 (25%)	3 (25%)	6 (50%)	

Executed script: \\SDS-SERVER2\SAS$\SDS3\Projects\STATISTICS DRUG DEV\NBMI4011 - CTI002\Analyser\Script\SAS\T_tfn001.sas, on: 23MAY2016:14:00, by: maria.lidsell
Version of source data: nbmi_tre.sas7bdat, last modified: 24FEB16:23:14, status: Final
*) Results are scored as ABSENT=0, SLIGHT=1, MODERATE=2.
**) Change can be interpreted as negative number=better, 0=unchanged, positive number=worse.

Table DE9.1 Shift table of Baseline vs. post baseline of finger to nose test (Full analysis set)

			Post baseline score*			
**Visit**	**Treatment**	**Baseline score* (Visit day 1)**	**0**	**1**	**2**	**Total**
Follow-up day 15	Total (n=36)	0	15 (68.18%)	7 (31.82%)		22 (61.11%)
		1	5 (35.71%)	8 (57.14%)	1 (7.14%)	14 (38.89%)
		Total	20 (55.56%)	15 (41.67%)	1 (2.78%)	36 (100%)
	NBMI100mg (n=13)	0	2 (40%)	3 (60%)		5 (38.46%)
		1	2 (25%)	5 (62.5%)	1 (12.5%)	8 (61.54%)
		Total	4 (30.77%)	8 (61.54%)	1 (7.69%)	13 (100%)
	NBMI300mg (n=11)	0	7 (87.5%)	1 (12.5%)		8 (72.73%)
		1	1 (33.33%)	2 (66.67%)		3 (27.27%)
		Total	8 (72.73%)	3 (27.27%)		11 (100%)
	Placebo (n=12)	0	6 (66.67%)	3 (33.33%)		9 (75%)
		1	2 (66.67%)	1 (33.33%)		3 (25%)
		Total	8 (66.67%)	4 (33.33%)		12 (100%)
Follow-up day 45	Total (n=36)	0	10 (45.45%)	11 (50%)	1 (4.55%)	22 (61.11%)
		1	5 (35.71%)	9 (64.29%)		14 (38.89%)
		Total	15 (41.67%)	20 (55.56%)	1 (2.78%)	36 (100%)
	NBMI100mg (n=13)	0	3 (60%)	2 (40%)		5 (38.46%)
		1	2 (25%)	6 (75%)		8 (61.54%)
		Total	5 (38.46%)	8 (61.54%)		13 (100%)
	NBMI300mg (n=11)	0	4 (50%)	3 (37.5%)	1 (12.5%)	8 (72.73%)
		1		3 (100%)		3 (27.27%)
		Total	4 (36.36%)	6 (54.55%)	1 (9.09%)	11 (100%)
	Placebo (n=12)	0	3 (33.33%)	6 (66.67%)		9 (75%)
		1	3 (100%)			3 (25%)
		Total	6 (50%)	6 (50%)		12 (100%)

Executed script: \\SDS-SERVER2\SAS$\SDS3\Projects\STATISTICS DRUG DEV\NBMI4011 - CTI002\Analyser\Script\SAS\T_tfn002.sas, on: 23MAY2016:14:00, by: maria.lidsell
Version of source data: nbmi_tre.sas7bdat, last modified: 24FEB16:23:14, status: Final
*) Scores are 0=ABSENT, 1=SLIGHT, 2=MODERATE.

Table DE10.1.1 Change from baseline of Memory loss digit span test, Digit span backwards (Full analysis set)

		Result									Change from baseline (Visit day 1)								
**Treatment**	**Visit**	**n**	**nmiss**	**Mean**	**SD**	**Q1**	**Median**	**Q3**	**Min**	**Max**	**n**	**nmiss**	**Mean**	**SD**	**Q1**	**Median**	**Q3**	**Min**	**Max**
Total (n=36)	Visit day 1	17 (47.22%)	0 (0%)	3.3	0.92	2	4	4	2	4									
	Follow-up day 15	17 (47.22%)	0 (0%)	3.6	0.61	3	4	4	3	5	17 (47.22%)	0 (0%)	0.4	1	0	0	1	-1	2
	Follow-up day 45	17 (47.22%)	0 (0%)	4	1	3	4	5	2	6	17 (47.22%)	0 (0%)	0.7	1.05	0	1	1	-1	3
NBMI100mg (n=13)	Visit day 1	6 (46.15%)	0 (0%)	3.3	0.82	3	3.5	4	2	4									
	Follow-up day 15	6 (46.15%)	0 (0%)	3.8	0.75	3	4	4	3	5	6 (46.15%)	0 (0%)	0.5	0.84	0	0	1	0	2
	Follow-up day 45	6 (46.15%)	0 (0%)	4	0.63	4	4	4	3	5	6 (46.15%)	0 (0%)	0.7	0.52	0	1	1	0	1
NBMI300mg (n=11)	Visit day 1	5 (45.45%)	0 (0%)	3.2	1.1	2	4	4	2	4									
	Follow-up day 15	5 (45.45%)	0 (0%)	3.8	0.45	4	4	4	3	4	5 (45.45%)	0 (0%)	0.6	1.34	0	0	2	-1	2
	Follow-up day 45	5 (45.45%)	0 (0%)	4.6	0.89	4	4	5	4	6	5 (45.45%)	0 (0%)	1.4	1.34	0	2	2	0	3
Placebo (n=12)	Visit day 1	6 (50%)	0 (0%)	3.3	1.03	2	4	4	2	4									
	Follow-up day 15	6 (50%)	0 (0%)	3.3	0.52	3	3	4	3	4	6 (50%)	0 (0%)	0	0.89	-1	0	1	-1	1
	Follow-up day 45	6 (50%)	0 (0%)	3.5	1.22	3	3	5	2	5	6 (50%)	0 (0%)	0.2	0.98	-1	0.5	1	-1	1

Executed script: \\SDS-SERVER2\SAS$\SDS3\Projects\STATISTICS DRUG DEV\NBMI4011 - CTI002\Analyser\Script\SAS\T_mds001.sas, on: 23MAY2016:14:00, by: maria.lidsell
Version of source data: nbmi_mb1.sas7bdat, last modified: 24FEB16:23:13, status: Final

Table DE10.2.1 Change from baseline of Memory loss digit span test, Digit span forward (Full analysis set)

		Result									Change from baseline (Visit day 1)								
**Treatment**	**Visit**	**n**	**nmiss**	**Mean**	**SD**	**Q1**	**Median**	**Q3**	**Min**	**Max**	**n**	**nmiss**	**Mean**	**SD**	**Q1**	**Median**	**Q3**	**Min**	**Max**
Total (n=36)	Visit day 1	17 (47.22%)	0 (0%)	5	0.94	4	5	5	4	7									
	Follow-up day 15	17 (47.22%)	0 (0%)	5.5	1.07	5	5	6	4	8	17 (47.22%)	0 (0%)	0.5	1.12	0	1	1	-2	3
	Follow-up day 45	17 (47.22%)	0 (0%)	5.2	0.9	5	5	5	4	8	17 (47.22%)	0 (0%)	0.2	0.83	0	0	1	-2	1
NBMI100mg (n=13)	Visit day 1	6 (46.15%)	0 (0%)	4.8	1.17	4	4.5	5	4	7									
	Follow-up day 15	6 (46.15%)	0 (0%)	5.3	1.03	5	5	6	4	7	6 (46.15%)	0 (0%)	0.5	0.55	0	0.5	1	0	1
	Follow-up day 45	6 (46.15%)	0 (0%)	5.7	1.37	5	5.5	6	4	8	6 (46.15%)	0 (0%)	0.8	0.41	1	1	1	0	1
NBMI300mg (n=11)	Visit day 1	5 (45.45%)	0 (0%)	5.6	0.89	5	5	6	5	7									
	Follow-up day 15	5 (45.45%)	0 (0%)	5.8	1.3	5	5	6	5	8	5 (45.45%)	0 (0%)	0.2	1.92	-1	0	1	-2	3
	Follow-up day 45	5 (45.45%)	0 (0%)	5	0	5	5	5	5	5	5 (45.45%)	0 (0%)	-0.6	0.89	-1	0	0	-2	0
Placebo (n=12)	Visit day 1	6 (50%)	0 (0%)	4.7	0.52	4	5	5	4	5									
	Follow-up day 15	6 (50%)	0 (0%)	5.5	1.05	5	5.5	6	4	7	6 (50%)	0 (0%)	0.8	0.75	0	1	1	0	2
	Follow-up day 45	6 (50%)	0 (0%)	5	0.63	5	5	5	4	6	6 (50%)	0 (0%)	0.3	0.52	0	0	1	0	1

Executed script: \\SDS-SERVER2\SAS$\SDS3\Projects\STATISTICS DRUG DEV\NBMI4011 - CTI002\Analyser\Script\SAS\T_mds001.sas, on: 23MAY2016:14:00, by: maria.lidsell
Version of source data: nbmi_mb1.sas7bdat, last modified: 24FEB16:23:13, status: Final

Table DE11.1.1 Change from baseline of Plasma Hg (µg/L) (Full analysis set)

		**Result**									**Change from baseline (Visit day 1)**								
**Treatment**	**Visit**	**n**	**nmiss**	**Mean**	**SD**	**Q1**	**Median**	**Q3**	**Min**	**Max**	**n**	**nmiss**	**Mean**	**SD**	**Q1**	**Median**	**Q3**	**Min**	**Max**
Total (n=36)	Visit day 1	36 (100%)	0 (0%)	14.6	19.63	3.405	6.12	20	1.3	95.9									
	Follow-up day 15	36 (100%)	0 (0%)	11.4	13.23	2.985	5.97	17.4	1.46	64.4	36 (100%)	0 (0%)	-3.1	9.15	-2.815	-0.335	0.73	-35.7	7.8
	Follow-up day 45	35 (97.22%)	1 (2.78%)	14.9	20.91	2.72	6.3	17.6	0.971	90.1	35 (97.22%)	1 (2.78%)	0.1	18.29	-2.22	-0.25	2.75	-48.13	56.5
NBMI100mg (n=13)	Visit day 1	13 (100%)	0 (0%)	10.5	8.27	4.96	6.42	18.8	1.66	25.8									
	Follow-up day 15	13 (100%)	0 (0%)	10.2	9.24	3.28	7.98	11	2.56	33.6	13 (100%)	0 (0%)	-0.3	3.65	-2.4	-0.7	1.62	-7.8	7.8
	Follow-up day 45	13 (100%)	0 (0%)	12.4	12.84	3.5	9.3	14.5	1.57	42.2	13 (100%)	0 (0%)	1.9	6.79	-2.22	-0.22	5.28	-8	16.4
NBMI300mg (n=11)	Visit day 1	11 (100%)	0 (0%)	21.8	31.03	2.34	5.81	45.7	1.3	95.9									
	Follow-up day 15	11 (100%)	0 (0%)	13.6	18.27	2.5	6.78	19.6	1.72	64.4	11 (100%)	0 (0%)	-8.2	14.95	-26.1	-0.3	0.48	-35.7	2.87
	Follow-up day 45	10 (90.91%)	1 (9.09%)	13.5	15.2	2.72	6.365	21.7	1.84	49	10 (90.91%)	1 (9.09%)	-9.9	21.97	-24	-0.15	1.36	-48.13	13.9
Placebo (n=12)	Visit day 1	12 (100%)	0 (0%)	12.3	14.12	2.305	6.105	19.85	1.44	45.8									
	Follow-up day 15	12 (100%)	0 (0%)	10.7	12.38	2.35	5.085	17.7	1.46	42.9	12 (100%)	0 (0%)	-1.6	3.38	-3.44	-0.075	0.43	-9.4	1.8
	Follow-up day 45	12 (100%)	0 (0%)	18.7	30.96	2.31	5.865	12.665	0.971	90.1	12 (100%)	0 (0%)	6.4	21.25	-2.025	-0.32	2.24	-13.1	56.5

Executed script: \\SDS-SERVER2\SAS$\SDS3\Projects\STATISTICS DRUG DEV\NBMI4011 - CTI002\Analyser\Script\SAS\T_hgp001.sas, on: 23MAY2016:14:00, by: maria.lidsell
Version of source data: nbmi_bi3.sas7bdat, last modified: 24FEB16:23:13, status: Final
External source file: L1606555.csv, downloaded at: 2016-03-30 14:31

Table DE12.1.1 Change from baseline of Urine Hg (µg/L) (Full analysis set)

		Result									Change from baseline (Visit day 1)								
**Treatment**	**Visit**	**n**	**nmiss**	**Mean**	**SD**	**Q1**	**Median**	**Q3**	**Min**	**Max**	**n**	**nmiss**	**Mean**	**SD**	**Q1**	**Median**	**Q3**	**Min**	**Max**
Total (n=36)	Visit day 1	36 (100%)	0 (0%)	65.2	80.16	9.865	32.45	89.15	1.66	321									
	Follow-up day 15	36 (100%)	0 (0%)	39.9	42.3	9.155	26.25	55.55	1.33	161	36 (100%)	0 (0%)	-25.3	63.93	-42.5	-4.49	6.785	-240.9	76.1
	Follow-up day 45	35 (97.22%)	1 (2.78%)	45	60.53	4.75	25.6	63.2	1.31	275	35 (97.22%)	1 (2.78%)	-20.3	59.41	-25.7	-1.57	4.64	-246.9	49.28
NBMI100mg (n=13)	Visit day 1	13 (100%)	0 (0%)	56.3	42.4	29.9	39.1	73.9	12.6	160									
	Follow-up day 15	13 (100%)	0 (0%)	47.5	32.38	30.6	37.7	62.1	8.14	132	13 (100%)	0 (0%)	-8.8	50.89	-41.7	-6.36	24.1	-128.6	76.1
	Follow-up day 45	13 (100%)	0 (0%)	41.7	26.9	25.6	39.1	63	2.64	96.1	13 (100%)	0 (0%)	-14.7	40.39	-32.36	-1.5	11.8	-126.5	22.2
NBMI300mg (n=11)	Visit day 1	11 (100%)	0 (0%)	98.6	115.6	9.91	26.5	213	4.22	321									
	Follow-up day 15	11 (100%)	0 (0%)	33.1	41.09	2.84	18.5	59.6	2.1	131	11 (100%)	0 (0%)	-65.5	84.87	-153.4	-8.16	-2.25	-240.9	0.8
	Follow-up day 45	10 (90.91%)	1 (9.09%)	47.2	69.23	4.34	14.185	65.6	1.81	226	10 (90.91%)	1 (9.09%)	-55	87.74	-127.8	-6	-1.57	-246.9	6.8
Placebo (n=12)	Visit day 1	12 (100%)	0 (0%)	44.1	68.14	3.915	9.16	72.5	1.66	230									
	Follow-up day 15	12 (100%)	0 (0%)	38	53.8	7.175	13.9	35.8	1.33	161	12 (100%)	0 (0%)	-6.2	35.95	-20.16	2.71	9.58	-71.9	45.9
	Follow-up day 45	12 (100%)	0 (0%)	46.7	81.2	3.91	11.25	42.65	1.31	275	12 (100%)	0 (0%)	2.6	33.59	-6.025	0.12	22.77	-74.7	49.28

Executed script: \\SDS-SERVER2\SAS$\SDS3\Projects\STATISTICS DRUG DEV\NBMI4011 - CTI002\Analyser\Script\SAS\T_hgu002.sas, on: 23MAY2016:14:00, by: maria.lidsell
Version of source data: nbmi_bi3.sas7bdat, last modified: 24FEB16:23:13, status: Final
External source file: L1606554.csv, downloaded at: 2016-04-04 13:22

Table DE12.2.1 Change from baseline of Urine Hg (µg/g Creatinine) (Full analysis set)

		Result									Change from baseline (Visit day 1)								
**Treatment**	**Visit**	**n**	**nmiss**	**Mean**	**SD**	**Q1**	**Median**	**Q3**	**Min**	**Max**	**n**	**nmiss**	**Mean**	**SD**	**Q1**	**Median**	**Q3**	**Min**	**Max**
Total (n=36)	Visit day 1	36 (100%)	0 (0%)	127.4	180.07	17.41	55.02	167.23	2.63	773.2									
	Follow-up day 15	36 (100%)	0 (0%)	89.6	109.7	17.53	64.22	110.55	1.81	536.67	36 (100%)	0 (0%)	-37.8	147.31	-40.9	-4.31	15.87	-475.47	297.08
	Follow-up day 45	35 (97.22%)	1 (2.78%)	178.8	429.6	9.56	38.58	120.13	0.4	1883.33	35 (97.22%)	1 (2.78%)	50.8	353.07	-44.1	-6.54	11.68	-463.33	1557.8
NBMI100mg (n=13)	Visit day 1	13 (100%)	0 (0%)	84.9	59.6	31.82	66.67	107.76	12.39	200									
	Follow-up day 15	13 (100%)	0 (0%)	90.9	73.1	35.28	64.36	138.45	17.18	241	13 (100%)	0 (0%)	6	69.26	-39.29	8.21	38	-135.92	122.81
	Follow-up day 45	13 (100%)	0 (0%)	92.6	89.34	38.58	53.27	120.13	13.2	340.21	13 (100%)	0 (0%)	7.7	94	-28.86	-13.79	11.68	-140.18	232.45
NBMI300mg (n=11)	Visit day 1	11 (100%)	0 (0%)	199.1	271.33	15.07	62.35	411.54	7.58	773.2									
	Follow-up day 15	11 (100%)	0 (0%)	89.7	108.24	8.63	65.15	97.5	2.63	298	11 (100%)	0 (0%)	-109.3	175.9	-302	-24.6	-0.84	-475.47	79
	Follow-up day 45	10 (90.91%)	1 (9.09%)	275.5	587.17	5.42	34.79	136.67	2.93	1883.33	10 (90.91%)	1 (9.09%)	67.1	397.61	-53.19	-3.96	24.7	-463.33	1110.14
Placebo (n=12)	Visit day 1	12 (100%)	0 (0%)	107.8	157.07	9.59	18.74	208.06	2.63	488.5									
	Follow-up day 15	12 (100%)	0 (0%)	88.1	147.67	15.01	29.75	96.22	1.81	536.67	12 (100%)	0 (0%)	-19.7	167.14	-23.36	1.77	15.87	-407.88	297.08
	Follow-up day 45	12 (100%)	0 (0%)	191.6	512.04	7.25	9.86	73.26	0.4	1797.39	12 (100%)	0 (0%)	83.8	492.65	-79.87	-5.86	-1.43	-453.65	1557.8

Executed script: \\SDS-SERVER2\SAS$\SDS3\Projects\STATISTICS DRUG DEV\NBMI4011 - CTI002\Analyser\Script\SAS\T_hgu002.sas, on: 23MAY2016:14:00, by: maria.lidsell
Version of source data: nbmi_bi3.sas7bdat, last modified: 24FEB16:23:13, status: Final
External source file: L1606554.csv, downloaded at: 2016-04-04 13:22

Table E1.1 Statistical analysis (ANCOVA) of change from baseline of Mercury intoxication medical score sum (Full analysis set)

					Difference from Placebo		
**Visit**	**Treatment**	**n**	**LSmean**	**95% CI**	**LSmean**	**95% CI**	**P-value**
Follow-up day 15	NBMI100mg	13	-2.308823	(-3.017,-1.601)	0.189	(-0.904, 1.283)	0.7267
	NBMI300mg	11	-3.182585	(-3.939,-2.426)	-0.685	(-1.796, 0.427)	0.2186
	Placebo	12	-2.498073	(-3.270,-1.726)	.		.

Executed script: \\SDS-SERVER2\SAS$\SDS3\Projects\STATISTICS DRUG DEV\NBMI4011 - CTI002\Analyser\Script\SAS\A_ms001.sas, on: 17MAR2016:16:40, by: maria.lidsell
Version of source data: nbmi_ma2.sas7bdat, last modified: 24FEB16:22:13, status: Final

Table E2.1.1 Statistical analysis (Wilcoxons two-sample test) of change from baseline of Neuro-motoric test battery (CATSYS) Tremor intensity (Full analysis set)

							Comparison vs. Placebo	
**Hand**	**Visit**	**Treatment**	**n**	**Median**	**Mean Score**	**Sum of Scores**	**Statistic**	**P-value**
Right	Follow-up day 15	NBMI300mg	11	0.01	11.23	123.50	123.50	0.6212
		Placebo	12	0.01	12.71	152.50	.	.

Table E2.2.1 Statistical analysis (Wilcoxons two-sample test) of change from baseline of Neuro-motoric test battery (CATSYS) Tremor intensity (Full analysis set)

							Comparison vs. Placebo	
**Hand**	**Visit**	**Treatment**	**n**	**Median**	**Mean Score**	**Sum of Scores**	**Statistic**	**P-value**
Right	Follow-up day 15	NBMI100mg	13	0	11.73	152.50	172.50	0.3824
		Placebo	12	0.01	14.38	172.50	.	.

Table E2.3.1 Statistical analysis (Wilcoxons two-sample test) of change from baseline of Neuro-motoric test battery (CATSYS) Tremor intensity (Full analysis set)

							Comparison vs. Placebo	
**Hand**	**Visit**	**Treatment**	**n**	**Median**	**Mean Score**	**Sum of Scores**	**Statistic**	**P-value**
Left	Follow-up day 15	NBMI300mg	11	0.01	10.64	117.0	117.00	0.3690
		Placebo	12	0.01	13.25	159.0	.	.

Table E2.4.1 Statistical analysis (Wilcoxons two-sample test) of change from baseline of Neuro-motoric test battery (CATSYS) Tremor intensity (Full analysis set)

							Comparison vs. Placebo	
**Hand**	**Visit**	**Treatment**	**n**	**Median**	**Mean Score**	**Sum of Scores**	**Statistic**	**P-value**
Left	Follow-up day 15	NBMI100mg	13	-0.01	10.23	133.0	192.00	0.0527
		Placebo	12	0.01	16.00	192.0	.	.

Executed script: \\SDS-SERVER2\SAS$\SDS3\Projects\STATISTICS DRUG DEV\NBMI4011 - CTI002\Analyser\Script\SAS\A_cat001.sas, on: 17MAR2016:16:40, by: maria.lidsell
Version of source data: nbmi_neu.sas7bdat, last modified: 24FEB16:22:13, status: Final

Table E3.1.1 Statistical analysis (MMRM) of physical and mental fatigue sum scores - Total fatigue score sum (Full analysis set)

					Difference from Placebo		
**Visit**	**Treatment**	**n**	**LSmean**	**95% CI**	**LSmean**	**95% CI**	**P-value**
Follow-up day 15	NBMI100mg	13	-5.769	(-8.364,-3.175)	-2.436	(-5.815, 0.943)	0.1572
	NBMI300mg	11	-6.727	(-9.343,-4.112)	-3.394	(-6.789, 0.002)	0.0501
	Placebo	12	-3.333	(-5.499,-1.168)	.		.

Table E3.2.1 Statistical analysis (MMRM) of physical and mental fatigue sum scores - Physical fatigue score sum (Full analysis set)

					Difference from Placebo		
**Visit**	**Treatment**	**n**	**LSmean**	**95% CI**	**LSmean**	**95% CI**	**P-value**
Follow-up day 15	NBMI100mg	13	-3.769	(-5.343,-2.195)	-1.353	(-3.433, 0.728)	0.2019
	NBMI300mg	11	-4.545	(-6.049,-3.042)	-2.129	(-4.156,-0.102)	0.0396
	Placebo	12	-2.417	(-3.777,-1.057)	.		.

Table E3.3.1 Statistical analysis (MMRM) of physical and mental fatigue sum scores - Mental fatigue score sum (Full analysis set)

					Difference from Placebo		
**Visit**	**Treatment**	**n**	**LSmean**	**95% CI**	**LSmean**	**95% CI**	**P-value**
Follow-up day 15	NBMI100mg	13	-2.000	(-3.048,-0.952)	-1.083	(-2.432, 0.266)	0.1152
	NBMI300mg	11	-2.182	(-3.335,-1.029)	-1.265	(-2.698, 0.167)	0.0833
	Placebo	12	-0.917	(-1.767,-0.067)	.		.

Executed script: \\SDS-SERVER2\SAS$\SDS3\Projects\STATISTICS DRUG DEV\NBMI4011 - CTI002\Analyser\Script\SAS\A_fs001.sas, on: 17MAR2016:16:40, by: maria.lidsell
Version of source data: nbmi_ph3.sas7bdat, last modified: 24FEB16:22:13, status: Final

Table E4.1.1 Statistical analysis (Wilcoxons two sample test) of change from baseline of Tremor finger-to-nose test (Full analysis set)

						Comparison vs. Placebo	
Visit	Treatment	n	Median	Mean Score	Sum of Scores	Statistic	P-value
Follow-up day 15	NBMI100mg	13	0	13.35	173.50	151.50	0.9437
	Placebo	12	0	12.63	151.50	.	.

Table E4.2.1 Statistical analysis (Wilcoxons two sample test) of change from baseline of Tremor finger-to-nose test (Full analysis set)

						Comparison vs. Placebo	
Visit	Treatment	n	Median	Mean Score	Sum of Scores	Statistic	P-value
Follow-up day 15	NBMI300mg	11	0	11.55	127.0	127.00	0.8108
	Placebo	12	0	12.42	149.0	.	.

Executed script: \\SDS-SERVER2\SAS$\SDS3\Projects\STATISTICS DRUG DEV\NBMI4011 - CTI002\Analyser\Script\SAS\A_tfn001.sas, on: 17MAR2016:16:40, by: maria.lidsell
Version of source data: nbmi_tre.sas7bdat, last modified: 24FEB16:22:14, status: Final

Table E5.1.1 Statistical analysis (Wilcoxons two sample test) of change from baseline of plasma Hg (µg/L) (Full analysis set)

						Comparison vs. Placebo	
**Visit**	**Treatment**	**n**	**Median**	**Mean Score**	**Sum of Scores**	**Statistic**	**P-value**
Follow-up day 15	NBMI100mg	13	-0.7	13.81	179.50	145.50	0.5864
	Placebo	12	-0.075	12.13	145.50	.	.

Executed script: \\SDS-SERVER2\SAS$\SDS3\Projects\STATISTICS DRUG DEV\NBMI4011 - CTI002\Analyser\Script\SAS\A_hgp001.sas, on: 23MAY2016:12:30, by: maria.lidsell
Version of source data: nbmi_bi3.sas7bdat, last modified: 24FEB16:23:13, status: Final
External source file: L1606555.csv, downloaded at: 2016-03-30 14:31

Table E5.2.1 Statistical analysis (Wilcoxons two sample test) of change from baseline of plasma Hg (µg/L) (Full analysis set)

						Comparison vs. Placebo	
**Visit**	**Treatment**	**n**	**Median**	**Mean Score**	**Sum of Scores**	**Statistic**	**P-value**
Follow-up day 15	NBMI300mg	11	-0.3	11.55	127.0	127.00	0.7818
	Placebo	12	-0.075	12.42	149.0	.	.

Executed script: \\SDS-SERVER2\SAS$\SDS3\Projects\STATISTICS DRUG DEV\NBMI4011 - CTI002\Analyser\Script\SAS\A_hgp001.sas, on: 23MAY2016:12:30, by: maria.lidsell
Version of source data: nbmi_bi3.sas7bdat, last modified: 24FEB16:23:13, status: Final
External source file: L1606555.csv, downloaded at: 2016-03-30 14:31

Table E6.1.1 Statistical analysis (Wilcoxons two sample test) of change from baseline of urine Hg (µg/L) (Full analysis set)

						Comparison vs. Placebo	
**Visit**	**Treatment**	**n**	**Median**	**Mean Score**	**Sum of Scores**	**Statistic**	**P-value**
Follow-up day 15	NBMI100mg	13	-6.36	12.77	166.0	159.00	0.8918
	Placebo	12	2.71	13.25	159.0	.	.

Executed script: \\SDS-SERVER2\SAS$\SDS3\Projects\STATISTICS DRUG DEV\NBMI4011 - CTI002\Analyser\Script\SAS\A_hgu001.sas, on: 23MAY2016:12:30, by: maria.lidsell
Version of source data: nbmi_bi3.sas7bdat, last modified: 24FEB16:23:13, status: Final
External source file: L1606554.csv, downloaded at: 2016-04-04 13:22

Table E6.2.1 Statistical analysis (Wilcoxons two sample test) of change from baseline of urine Hg (µg/L) (Full analysis set)

						Comparison vs. Placebo	
**Visit**	**Treatment**	**n**	**Median**	**Mean Score**	**Sum of Scores**	**Statistic**	**P-value**
Follow-up day 15	NBMI300mg	11	-8.16	8.18	90.0	90.00	0.0106
	Placebo	12	2.71	15.50	186.0	.	.

Executed script: \\SDS-SERVER2\SAS$\SDS3\Projects\STATISTICS DRUG DEV\NBMI4011 - CTI002\Analyser\Script\SAS\A_hgu001.sas, on: 23MAY2016:12:30, by: maria.lidsell
Version of source data: nbmi_bi3.sas7bdat, last modified: 24FEB16:23:13, status: Final
External source file: L1606554.csv, downloaded at: 2016-04-04 13:22

Table E6.3.1 Statistical analysis (Wilcoxons two sample test) of change from baseline of urine Hg (µg/g Creatinine) (Full analysis set)

						Comparison vs. Placebo	
**Visit**	**Treatment**	**n**	**Median**	**Mean Score**	**Sum of Scores**	**Statistic**	**P-value**
Follow-up day 15	NBMI100mg	13	8.2053963	13.31	173.0	152.00	0.8490
	Placebo	12	1.7692093	12.67	152.0	.	.

Executed script: \\SDS-SERVER2\SAS$\SDS3\Projects\STATISTICS DRUG DEV\NBMI4011 - CTI002\Analyser\Script\SAS\A_hgu001.sas, on: 23MAY2016:12:30, by: maria.lidsell
Version of source data: nbmi_bi3.sas7bdat, last modified: 24FEB16:23:13, status: Final
External source file: L1606554.csv, downloaded at: 2016-04-04 13:22

Table E6.4.1 Statistical analysis (Wilcoxons two sample test) of change from baseline of urine Hg (µg/g Creatinine) (Full analysis set)

						Comparison vs. Placebo	
Visit	Treatment	n	Median	Mean Score	Sum of Scores	Statistic	P-value
Follow-up day 15	NBMI300mg	11	-24.59784	9.45	104.0	104.00	0.0905
	Placebo	12	1.7692093	14.33	172.0	.	.

Executed script: \\SDS-SERVER2\SAS$\SDS3\Projects\STATISTICS DRUG DEV\NBMI4011 - CTI002\Analyser\Script\SAS\A_hgu001.sas, on: 23MAY2016:12:30, by: maria.lidsell
Version of source data: nbmi_bi3.sas7bdat, last modified: 24FEB16:23:13, status: Final
External source file: L1606554.csv, downloaded at: 2016-04-04 13:22
